# Soil metaproteomics reveals an inter-kingdom stress response to the presence of black truffles

**DOI:** 10.1038/srep25773

**Published:** 2016-05-10

**Authors:** Elisa Zampieri, Marco Chiapello, Stefania Daghino, Paola Bonfante, Antonietta Mello

**Affiliations:** 1 Department of Life Sciences and Systems Biology, University of Torino, Viale P.A. Mattioli 25, I-10125 Torino, Italy; 2 Institute for Sustainable Plant Protection, CNR, Torino Unit, Viale P.A. Mattioli 25, I-10125 Torino, Italy

## Abstract

For some truffle species of the *Tuber* genus, the symbiotic phase is often associated with the presence of an area of scant vegetation, commonly known as the brûlé, around the host tree. Previous metagenomics studies have identified the microorganisms present inside and outside the brûlé of a *Tuber melanosporum* truffle-ground, but the molecular mechanisms that operate in this ecological niche remain to be clarified. To elucidate the metabolic pathways present in the brûlé, we conducted a metaproteomics analysis on the soil of a characterized truffle-ground and cross-referenced the resulting proteins with a database we constructed, incorporating the metagenomics data for the organisms previously identified in this soil. The soil inside the brûlé contained a larger number of proteins and, surprisingly, more proteins from plants, compared with the soil outside the brûlé. In addition, Fisher’s Exact Tests detected more biological processes inside the brûlé; these processes were related to responses to multiple types of stress. Thus, although the brûlé has a reduced diversity of plant and microbial species, the organisms in the brûlé show strong metabolic activity. Also, the combination of metagenomics and metaproteomics provides a powerful tool to reveal soil functioning.

Truffles, one of the world’s most prized and expensive foods, are ectomycorrhizal (EcM) fungi that have some unusual biological features. Examination of the genome sequence of the black truffle *Tuber melanosporum* revealed a relatively large genome (125 Mb), a heterothallic mating type system, and the absence of toxin-coding genes^1^. For some *Tuber* species such as *T. melanosporum* and *T. aestivum*^2^, development of the EcM symbiosis and production of the hypogeous fruiting bodies lead to the formation of a burnt area (commonly referred to by the French word brûlé), characterized by scarce vegetation around their symbiotic plants (Fig. 1). Several hypotheses have been proposed to explain this striking phenomenon: parasitism of *Tuber* on the non-host herbaceous plants^3^, competition for nutrients or water^4^, and phytotoxic effects of truffle metabolites and volatiles^2^,^5^,^6^,^7^,^8^. In initial attempts to understand the microbial ecology of the brûlé, two studies examined cultivable fungi to identify the effect of *Tuber* spp. on fungal biodiversity^9^,^10^. Later studies used metagenomics to compare the fungal composition inside and outside the brûlé in French truffle-ground soils at Cahors^11^,^12^. This analysis showed clear differences between the fungal communities, including lower fungal biodiversity inside the brûlé. *T. melanosporum* was the dominant fungus within the brûlé, but Basidiomycota, which are mostly EcM fungi, showed decreased abundance in the brûlé, indicating that *T. melanosporum* may compete with other EcM fungi.

Most herbaceous plants form symbioses with arbuscular mycorrhizal fungi (AMF), which belong to the phylum Glomeromycota^13^. Mello and colleagues examined whether the scant plant coverage in the brûlé reflects a change in AMF biodiversity, compared with the area outside the brûlé^14^. They found that the patchy herbaceous plants around a *T. melanosporum* host tree were extensively colonized by AMF, as were the plants outside the brûlé, and that AMF richness did not seem to be affected in the herbaceous plants inside the brûlé. In contrast to the AMF on the roots, examination of AMF diversity in the soil showed reduced species richness of AMF in the soil inside the brulé, compared with the soil outside the brûlé. However, members of Diversispora, Acaulospora, and Archaeospora were only found in the brûlé, in roots or soil, suggesting that this habitat specifically affected certain AMF taxa. Studies in the same truffle-ground, using denaturing gradient gel electrophoresis and DNA microarray analysis^15^, found that *T. melanosporum* also affected bacterial and archaeal communities. In particular, Firmicutes (*e.g., Bacillus*), several genera of Actinobacteria, and a few Cyanobacteria were more abundant inside the brûlé, whereas *Pseudomonas* and several genera within the family Flavobacteriaceae were more abundant outside the brûlé. These metagenomics studies clearly revealed the fungal, archaeal, and bacterial community composition inside and outside the brûlé, but they did not examine the interaction and role of the different organisms. Linking microbial community composition and ecological processes will improve our understanding of the functioning of soil microbial communities.

Metaproteomics is the study of all the proteins expressed by the organisms within an ecosystem at a specific time^16^,^17^. It can be used to unravel the microbial activity, metabolic pathways, and signal transduction involved in the soil biotic community^18^ and to identify organisms present in different environments^19^. Microbial metaproteomics has been used to examine different environments, i.e., soil^18^,^20^,^21^, sediments^22^,^23^, marine^24^,^25^, and freshwater systems^26^,^27^,^28^, confirming metaproteomics as a powerful tool to describe metabolic processes active in these environments^29^. Wang and colleagues applied metaproteomics to crop rhizospheres to elucidate some of the interactions between crops and microorganisms in soil and identify biological processes on-going in the environment and related to energy production, protein biosynthesis and turnover, defence machinery, and secondary metabolism^18^.

Given the promise of metaproteomics, the aim of this work was to use this tool on the same truffle-ground soils previously characterized by metagenomics^11^,^12^,^14^,^15^, to produce a protein dataset that can help to elucidate the active metabolic pathways in organisms inside and outside the brûlé. Soil samples collected for the previous investigations in a productive truffle-ground in Cahors, France were used for protein extraction and LC-MS/MS analyses. Thanks to the availability of sequenced genomes from 2682 Eukaryota and 58252 Prokaryota (http://www.ncbi.nlm.nih.gov/genome/browse/), including the black truffle *Tuber melanosporum*^1^, these proteins were categorized and assigned to the organisms living in the truffle-ground, allowing us to infer metabolic processes.

## Results

### Extraction methods and total number of identified proteins

**Figure 2:**
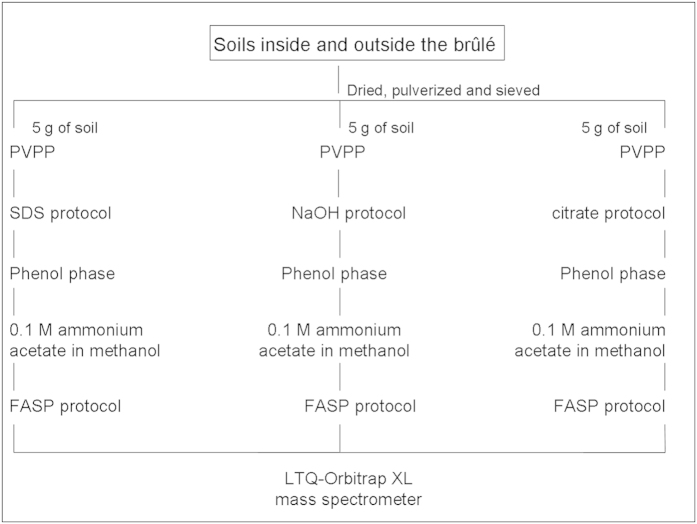
Schematic representation of the protein extraction methods.

Proteins from eight soil samples (four replicates inside and four outside the brûlé) collected in a French truffle-ground were extracted using three extraction methods to maximize the yield of proteins (Fig. 2). The extracts were pooled and examined by LC-MS/MS to identify proteins. We then cross-referenced all the proteins from the four replicates against a database specifically built for this work, based on the set of organisms previously identified in this soil by metagenomics, and against the *T. melanosporum* database. In the first case, we identified 638 proteins: 411 proteins were specific to the samples from inside the brûlé, 309 were specific to the samples from outside the brûlé, and 82 were common (Supplementary Table S1). In the second case, we identified 265 *T. melanosporum* proteins: 148 proteins were specific to the samples from inside the brûlé, 92 were specific to the samples from outside the brûlé, and only 25 were common between the two environments (Supplementary Table S2).

### Organismal classification of the identified proteins

All the pooled proteins from the biological replicates were used to carry out a functional and a putative phylogenetic classification, following the method described previously^17^. The proteins from eukaryotic species (168) were less abundant than proteins from bacteria (243) inside the brûlé as well as outside (71 vs 238) (Supplementary Fig. S1). At the kingdom level, we identified 77 proteins from Fungi, 243 from Bacteria, and 91 from Viridiplantae inside the brûlé. Outside the brûlé, the same kingdoms were represented, but with different numbers of proteins (64 proteins from Fungi, 238 from Bacteria, and 7 from Viridiplantae). The phyla represented by the most number of proteins inside the brûlé were Actinobacteria, followed by Streptophyta, Ascomycota, Proteobacteria, Firmicutes, Bacteroidetes, Glomeromycota, Cyanobacteria, Nitrospirae, and Basidiomycota; outside the brûlé, Actinobacteria still predominated, followed by Proteobacteria, Ascomycota, Firmicutes, Bacteroidetes, Streptophyta, Basidiomycota, Cyanobacteria, Glomeromycota, and Nitrospirae. At the class level, the predominant class inside the brûlé was the Actinobacteria, followed by Magnoliopsida, Gammaproteobacteria, Bacilli, Eurotiomycetes, Sordariomycetes, Pezizomycetes (which includes truffle fungi), Flavobacteriia, Rubrobacteria, and Glomeromycetes, while the predominant classes outside the brûlé were Actinobacteria, Gammaproteobacteria, Bacilli, Sordariomycetes, Eurotiomycetes, Rubrobacteria, Deltaproteobacteria, Betaproteobacteria, Flavobacteriia, and Magnoliopsida.

### Gene Ontology and KEGG classification

**Figure 3:**
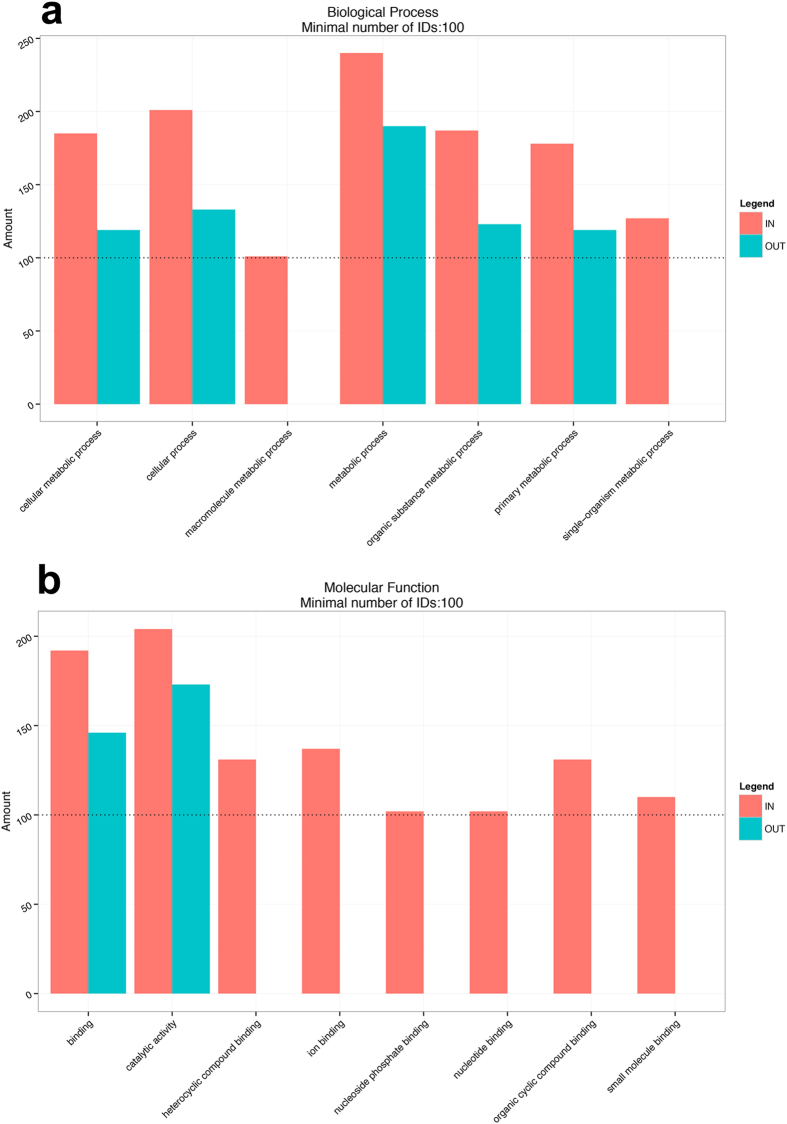
Categorization by Gene Ontology analysis into biological processes (a) and in molecular functions (b) having a minimum of 100 proteins identified.

The identified proteins were annotated by Blast2GO and categorized according to their functions into biological processes, molecular functions, and cellular components. Gene Ontology analyses showed that inside the brûlé the biological process categories having at least 100 protein identifications (IDs) were related to metabolic processes (Fig. 3a). The biological process categories were the same in the two environments, with the exception of macromolecule metabolic process and single-organism metabolic process, which were assigned only for proteins identified inside the brûlé.

The molecular functions of proteins with a minimum of 100 IDs were mostly related to “binding” (Fig. 3b). The molecular functions were principally assigned to proteins identified inside the brûlé, with the exception of binding and catalytic activity, which were common to soil inside and outside the brûlé. The cellular component categories of cell, membrane, and organelle proteins occurred in both environments whereas macromolecular complex was only present inside the brûlé (data not shown).

An analysis of differences in GO term frequency between the two sets of protein sequences (inside *vs* outside) was conducted for biological processes and molecular functions. The first analysis showed that 57 processes inside the brûlé were over-represented compared to those outside (Fisher Exact Test, *p* value < 0.05) (Table 1). The processes were principally related to Bacteria (*Actinomyces, Arthrobacter, Bacillus, Elizabethkingia, Flavobacterium, Frankia, Massilia, Microbacterium, Mycobacterium, Myxococcus, Nitrospira, Pedobacter, Pseudomonas, Riemerella, Rubrobacter*, and *Streptosporangium*), followed by Mesangiospermae (*Artemisia, Astragalus, Capsella, Festuca, Lathyrus, Lotus, Plantago, Quercus, Sedum, Taraxacum, Trifolium*, and *Vicia*) and Fungi (*Aspergillus, Beauveria, Fusarium, Gibberella, Hypocrea, Penicillium, Pyronema, Rhyzophagus, Scleroderma, Tuber*, and *Verticillium*). The respective proteins of the 57 processes were assigned to organisms and are shown in Supplementary Table S3. The list of proteins over-represented inside the brûlé included proteins related to stress, such as a heat shock protein from *Trifolium pratense*, a heat shock protein 70 from *Aspergillus flavus*, a group 2 late embryogenesis abundant protein (LEA) and a superoxide dismutase from *Lotus japonicus*, a chaperonin cpn60-mitochondrial-like protein and a heat shock protein 60-2 from *Capsella rubella*, a superoxide dismutase from *Riemerella anatipestifer*, and a molecular chaperone from *Nitrospira defluvii*. The over-represented proteins also included enzymes involved in glycolysis and the Krebs cycle, such as glyceraldehyde-3-phosphate dehydrogenase, fructose bisphosphate aldolase, enolase, ATP citrate lyase, phosphoenolpyruvate carboxylase, and isocitrate dehydrogenase principally identified in Mesangiospermae, proteins related to sulphur in plants and bacteria, an integrase of *Pseudomonas*, and a tyrosinase precursor of *T. melanosporum*. Out of the 57, 14 processes were present only inside the brûlé; these were related to response to some types of stress (osmotic and salt), stimuli (abiotic and temperature), inorganic substances, and metal ions (cadmium), protein catabolic processes, organic hydroxy compound metabolic process, sulphur compound metabolic process, and system development (Table 1). The response proteins belonged to Mesangiospermae, especially to *Capsella* and *Lotus*, followed by *Lathyrus, Quercus*, and *Vicia* (only one protein for these last three genera). Only one protein of a fungus, *A. flavus*, was present in this category. For protein catabolic processes, all proteins, except one from a fungus (*A. rambelli*), were from plants (*Capsella, Lotus, Taraxacum, Trifolium*, and *Plantago*). In the category of organic hydroxy compound metabolic process, proteins came mostly from plants (*Capsella, Astragalus*, and *Lotus*) and bacteria (*Rubrobacter, Pseudomonas, Streptosporangium, Actinomyces, Arthrobacter*, and *Bacillus*); two proteins from *Aspergillus* were also found. The process concerning sulphur involved only plant (*Capsella, Lotus, Lathyrus*, and *Vicia*) and bacterial (*Pseudomonas, Mycobacterium*, and *Bacillus*) proteins, such as S-adenosylmethionine synthase, sulphite reductase [NADPH] flavoprotein alpha-component and cysteine synthase. In the process concerning system development, only plant (*Capsella, Lotus, Astragalus, Trifolium, Salvia, Vicia*, and *Lathyrus*) proteins such as actin and beta-tubulin were found.

Using the Fisher’s exact test to identify the molecular functions showed that lyase activity was only represented inside the brûlé, not outside (*p* value < 0.05) (data not shown). It was related to Bacteria (*Bacillus, Elizabethkingia, Flavobacterium, Mycobacterium*, and *Pseudomonas*), Mesangiospermae (*Capsella, Lathyrus, Lotus*, and *Trifolium*), and Fungi (*Aspergillus*) (data not shown).

After the categorization of proteins into biological processes, molecular functions, and cellular components, the next step was to assign them to the metabolic pathways through KEGG analysis. In this regard, pathways involved in carbohydrate, fatty acid, nucleotide, and amino acid metabolism, carbon fixation in photosynthetic and in prokaryotic organisms, and sulphur metabolism were the most represented among those identified inside and outside the brûlé and these pathways are listed in Supplementary Table S4.

### Gene Ontology and KEGG classification of *Tuber melanosporum* proteins

**Figure 4:**
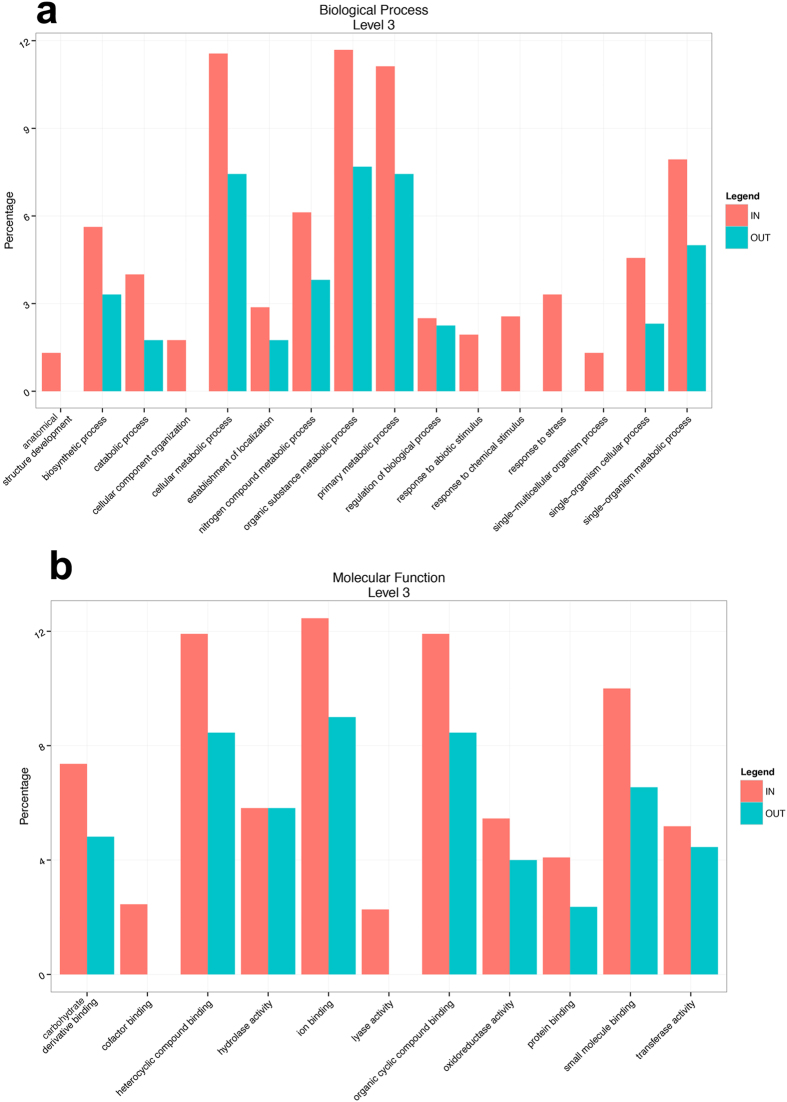
Categorization by Gene Ontology analysis in biological processes level 3 (a) and in molecular functions level 3 (b) of the *T. melanosporum* proteins.

Using the database of sequences from *T. melanosporum*, the dominant fungal organism inside the brûlé^11^, 16 biological processes were identified inside the brûlé and 10 outside (Fig. 4a). The elements that differed between the two environments were represented by a few proteins involved in the response to stress, response to chemical stimulus, response to abiotic stimulus, cell component organization, single-multicellular organism process, and anatomical structure development (assigned only inside). Eleven molecular functions were identified inside the brûlé (Fig. 4b) and the most abundant functions were: ion binding, heterocyclic compound binding, organic cyclic compound binding, small molecule binding, carbohydrate derivate binding, hydrolase activity, transferase activity, and protein binding. Outside the brûlé, nine functions were identified whose most abundant functions were the same as those identified inside the brûlé. Some functions were present inside and not outside, but they are represented by only a few sequences (cofactor binding and lyase activity). Interestingly, the proteins identified only inside were stress proteins, such as heat shock protein 60 and 98, Hsp90 co-chaperone Cdc37 and sti1, laccase, and tyrosinase (Supplementary Table S2).

The most-represented components in the cellular component category were found in both environments: cell, organelle, macromolecular complex, membrane, and membrane-enclosed lumen. The only element present inside and absent outside was the extracellular region (data not shown). However, although there were some differences in the Gene Ontology categories between *T. melanosporum* proteins inside and outside the brûlé, the Fisher Exact Test showed that they were not significant (*p* value > 0.05). Interestingly a branched-chain-amino-acid aminotransferase protein (mitochondrial) involved in the Ehrlich pathway, and an adenosyl-homocysteinase protein involved in cysteine/methionine biosynthesis and interconversion, were found inside and outside the brûlé, respectively.

For the KEGG analysis, pathways involved in nucleotide, amino acid, carbohydrate, and lipid metabolism were identified inside and outside the brûlé (data not shown).

## Discussion

The brûlé associated with the French truffle-ground at La Bigouse has been extensively investigated by metagenomics analysis, which produced a list of the microbes living in this ecological niche. In the present research, we built a database based on our previous metagenomics data^11^,^12^,^14^,^15^ and used it for metaproteomics analysis to link the composition of the microbial community to the ecological processes occurring in the brûlé.

Protein extraction from the soil remains a challenge because humic acids can interfere with quantification, separation, and identification of proteins^29^,^30^,^31^,^32^, and a combination of different extraction protocols instead of only one specific protocol was suggested for significantly higher coverage of the metaproteome^33^. Therefore, in this study, for the first time three different methods were employed and the resulting three extractions were combined prior to LC-MS/MS analyses. The identified proteins from four soil samples for each of the two habitats, inside and outside the brûlé, were pooled before the organismal classification and Gene Ontology analysis, to provide an overall view of the two environments, as suggested by Bastida and colleagues^17^. To our knowledge, this study also represents the first time a database of sequences based on metagenomics experiments was cross-referenced with a proteomic database to assign proteins to the organisms living in the selected truffle-ground and to infer metabolic processes.

The proteins detected in this analysis belong to Bacteria and Eukaryota, such as fungi and plants. Interestingly, more proteins were detected in the soil inside the brûlé than in the soil outside the brûlé and surprisingly, plant proteins were more abundant inside than outside *(p* value < 0.05). In particular, proteins from most of the herbaceous plants inhabiting the brûlé were found, indicating that the plants are active despite their scant abundance. For fungi, four Basidiomycota proteins were found outside the brûlé, and only one was found inside, consistent with previous studies that found more ectomycorrhizal Basidiomycota internal transcribed spacer (ITS) sequences outside the brûlé than inside^11^,^12^. The four Basidiomycota proteins found outside the brûlé belonged to the genera *Amanita* and *Scleroderma* whereas the one found inside belonged to *Scleroderma*. In a previous study^11^, *Scleroderma* sp*. and Xerocosmus rubellus* ITS sequences were exclusively found inside the brûlé; *Amanita, Tricholoma, Pulvinula*, and *Inocybe* were exclusively found outside; and *Tomentella* and *Hymenogaster* were common to the two habitats. Based on these data, proteins belonging to the genus *Scleroderma* were not expected in the outside environment. The presence of proteins belonging only to the genera *Amanita* and *Scleroderma* probably relates to the fact that among the genomes of all the Basidiomycota detected through their ITS sequences, only the genomes of *A. muscaria* and *S. citrinum* have been sequenced^34^. Two proteins found for *Scleroderma* and *Amanita* are uncharacterized and the other two were a Translation elongation factor EF1-alpha (fragment) of *A. mafingensis* and an ATP synthase f1 alpha subunit of *S. citrinum*.

Glomeromycota proteins were identified inside and outside the brûlé and assigned to the *Rhizophagus* genus only, although metagenomics studies have found other genera of this phylum^14^. Most of the *R. irregularis* proteins were uncharacterized, with the exception of proteins related to structural functions (actin, 40S ribosomal subunit, binding protein). However, ‘omics’ information for AMF remains limited; for *R. irregularis* (formerly *Glomus intraradices*), only genome and transcriptome data sets are available so far^35^,^36^,^37^, while for *Gigaspora margarita* transcriptome and proteome data are available^38^,^39^.

Proteins of the bacterial phyla identified inside and outside the brûlé^15^ were detected, but their abundance differed from the abundance of the corresponding organisms. This is in line with the fact that the protein organismal classification reflects the presence and activity of different taxonomic groups, but it is not related to the number of species present.

Taken as a whole, the results show good consistency between the metaproteomics and metagenomics data^11^,^12^,^14^,^15^. For example, *T. melanosporum* proteins were present in both the environments, as expected^11^,^12^. Some obvious discrepancies depend on the fact that identified proteins are ascribed to different organisms on the basis of the known protein sequence annotation, thus causing a bias related to sequence richness in databases^31^,^33^,^40^,^41^. Moreover, the organisms identified in the truffle-ground by DNA-based techniques could be dead, their metabolic processes could be inactive, and thus their proteins might not be detected.

The functional analysis identified proteins involved in diverse biological processes, such as those dealing with primary metabolism (carbohydrate, amino acid, lipid, energy production, and transport), and in different molecular functions, such as those related to binding and catalytic activity, both inside and outside the brûlé. Differences were evident: we identified significantly more biological processes inside the brûlé than outside and surprisingly, fourteen processes were only present inside the brûlé. These processes were related to response to some types of stress (osmotic and salt), stimuli (abiotic and temperature), inorganic substances, and metal ions (cadmium), catabolic process of protein, organic hydroxy compound metabolic process, sulphur compound metabolic process, and system development. The remaining 43 processes were over-represented inside the brûlé compared to outside. This demonstrated how the two environments differed from a metabolic point of view. The list of proteins over-represented inside showed that the proteins related to stress were present in plants, fungi, and bacteria. Since the category ‘response to stress’ principally consisted of proteins identified in herbaceous plants, we could hypothesize that those few plants living in the brûlé experience stress conditions. Given that our database included both the herbaceous plants and the host plant, one could wonder whether the plant tree also experiences stress conditions. However, our soil sampling strategy (soil collection and further removal of visible remains of plants) did not favour the retrieval of tree proteins; therefore, the presence of tree stress proteins in the brûlé cannot be excluded.

Most of the stress response proteins were heat shock proteins, LEA proteins, and superoxide dismutase. Heat shock proteins play a role in response to environmental stress conditions such as heat, cold, and drought, as well as to chemicals and other stresses, preventing aggregation and assisting in the refolding of non-native proteins^42^. LEA proteins are hydrophilic proteins that accumulate at the last stage of embryogenesis during seed dehydration^43^. They may play a protective role in plant vegetative tissues in different stress conditions. For example, Xu and colleagues demonstrated that in transgenic rice the expression of a LEA protein conferred tolerance to water deficit and salinity^44^. Within the defence proteins, superoxide dismutase plays a role in the detoxification of reactive oxygen species, catalysing the breakdown of superoxide into hydrogen peroxide and water^45^.

Carbohydrate catabolism was also active inside the brûlé, as indicated by the presence of enzymes, mostly identified in Mesangiospermae, involved in glycolysis and the Krebs cycle. Sulphur metabolism was also active, with proteins principally present in bacteria and plants. The key protein (s-adenosylmethionine synthetase) in sulphur metabolism was found in *Mycobacterium*, which is a genus found in ectomycorrhiza and soil adherent to the *T. melanosporum* fruiting body^46^.

The integrase found in *Pseudomonas* has a role in site-specific integration into the chromosome of bacterial host of the clc element, involved in the degradation of 3-chlorobenzoic acid via chlorocatechol^47^.

In the functional analysis of *T. melanosporum* proteins, some molecular functions and biological processes were detected only inside the brûlé, even if with a reduced number of IDs. The proteins present only inside the brûlé were heat shock proteins or other co-chaperones, together with proteins related to melanin, such as laccase and tyrosinase. Moreover, a protein involved in sulphur metabolism was found inside the brûlé, and another related to the Ehrlich pathway was found outside. Sulphur-containing volatiles and fusel alcohols, which are produced in truffles via amino acid catabolism through the Ehrlich pathway, are the major constituents of the truffle aroma^1^,^7^. However, the Fisher Exact Test indicated that these differences between the two environments were not significant (*p* value > 0.05). Interestingly, a *T. melanosporum* tyrosinase-precursor was significantly over-represented inside the brûlé when the soil protein dataset was analysed using the database, which we constructed from previous metagenomics data. The tyrosinase plays a role in the oxidation of diphenols and production of melanin; also, the gene coding for this protein is up-regulated during the symbiotic stage^1^. This finding is in agreement with the abundance of *T. melanosporum* inside the brûlé, where it is present as mycelium, as well as ectomycorrhiza and fruiting-body. However, on the basis of *T. melanosporum* proteins found in the brûlé, we can suggest that *T. melanosporum* proteins are not directly responsible for the phenomenon of the brûlé, i.e., the scant herbaceous vegetation. This finding seems to exclude a role of *T. melanosporum* proteins in affecting the herbaceous plants differently from *T. melanosporum* volatile organic compounds, whose role has been widely acknowledged by other authors^6^,^7^,^8^.

## Conclusions

**Figure 5:**
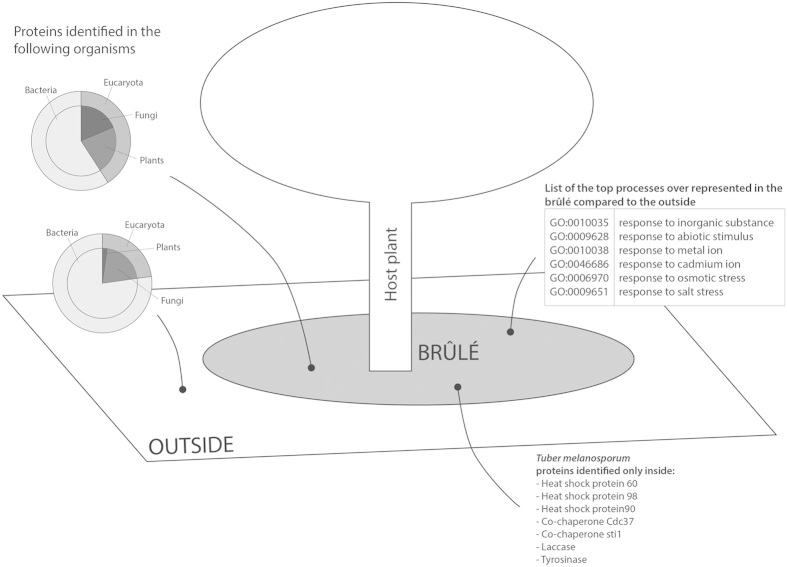
Schematic representation of the proteins and the processes detected inside and outside the *T. melanosporum* brûlé.

In this study, an optimized protein extraction protocol maximized protein yields, allowing us to successfully conduct a metaproteomics analysis of the soil from a truffle-ground. The resulting protein dataset was cross-referenced with the genomes/proteomes of microbes already identified in previous metagenomics analyses performed in the same environment. This combination produced novel and unexpected insights into the functionality of truffle-ground soils, and in particular in the brûlé area (Fig. 5). Even if the brûlé has a reduced biodiversity of bacteria, fungi and plants, as has been observed^11^,^12^,^15^, the biological processes active in the brûlé soil were over-represented compared with the soil outside the brûlé. Irrespective of its appearance and contrary to the word that identifies it, the brûlé seems to be a very active environment, dominated by broad stress responses from most of its components, and in particular by herbaceous plants. We can hypothesize that truffle metabolites, such as volatile organic compounds, may directly or indirectly elicit stress and defence responses in fungi and bacteria, but mostly in the surrounding herbaceous plants. Indeed, under laboratory conditions, *Arabidopsis* exposed to truffle volatile organic compounds produce an oxidative burst^6^. Although the in-field plants are good hosts for AM fungi, which can alleviate their stress responses^13^, their vegetative growth and multiplication are affected, leading to establishment of the brûlé.

We suggest that the description of complex natural events like the brûlé requires multiple approaches; in this context, the combination of metagenomics and metaproteomics has given a first glance at the functioning of a complex soil niche where the truffle is the main actor.

## Methods

### Site description and soil sampling

The sampling area is a productive and natural *T. melanosporum* truffle-ground situated in La Bigouse, and it belongs to La Station de la Trufficulture de Cahors-Le Montat (Station d’Experimentation sur la Truffe, Lycee Professionnel Agricole Lacoste, Le Montat, France).

The brûlé around the host tree *Quercus pubescens* showed an irregular shape^14^. The soil features were described^11^,^14^. In brief, inside the brûlé the pH is 7.84, the soil has a clay texture, 11.5 of C/N, 3% of N, 11.9% calcium carbonate, 2.3 meq/100 g of P, 0.27 meq/100 g of K, 0.16 meq/100 g of Mg, 40% limestone, and 59.7% organic matter. Outside the brûlé the pH is 7.76, the soil has a very clay texture, 13.2 of C/N, 3% N, 12.2% calcium carbonate, 2 meq/100 g of P, 0.31 meq/100 g of K, 0.19 meq/100 g of Mg, 25% limestone, and 68.3% organic matter. The herbaceous plants, present in a patchy distribution inside the brûlé and in a uniform distribution outside, belonged to the *Achillea, Alyssum, Arenaria, Artemisia, Astragalus, Bromus, Capsella, Cerastium, Cerinte, Clinopodium, Cynodon, Erigeron, Festuca, Gallium, Hieracium, Knautia, Lathyrus, Leucanthemum, Lotus, Myosotis, Plantago, Pimpinella, Poa, Reseda, Salvia, Sedum, Sonchus, Taraxacum, Trifolium, Veronica, Vicia*, and *Vulpia* genera^14^.

The soils were harvested in March 2008 in four replicates (both inside and outside the brûlé). Each replicate was composed of three homogeneously mixed subsamples in an effort to reduce the spatial variability of the soil. The soil cores were collected at a depth of approximately 10–15 cm, the plant remains were removed, the soil samples were sieved (2 mm), and then stored at −80 °C for future analyses.

### Protein extraction methods

Three different extraction methods with minor changes were used for each of the eight soil samples (Fig. 2): a citrate extraction method^18^, an SDS lysis method^33^, and a NaOH extraction^33^. The three protocols shared the first steps: the soils were dried, pulverized, sieved, and 5 grams of soil was ground in liquid N_2_ with 10% PVPP (w/w) before being used for the appropriate extraction method. In the citrate protocol, the soil was homogenized with 25 ml of citrate buffer (0.25 M, pH 8) and 2 mM PMSF; the homogenate was shaken for 3 h at room temperature, then centrifuged for 15′ at 16,000 g at 4 °C. In the SDS protocol, the soil was homogenized with SDS buffer (50 mM Tris-HCl pH 7.5, 1% SDS) and 2 mM PMSF, vortexed and sonicated for 5′ at low temperature. The homogenate was boiled for 20′, then vortexed and sonicated. These steps were followed by a centrifugation for 20′ at 16,000 g at 4 °C. In the NaOH protocol, the soil was homogenized in NaOH (0.1 M) and 2 mM PMSF, vortexed and sonicated on ice at 90% pulsing and a maximum of 40% energy twice for 1′. The homogenate was shaken for 30′ at 20 °C, then centrifuged for 20′ at 16,000 g at 4 °C. All the supernatants were filtered through a nylon mesh (0.45 μm). The different filtered supernatants were amended with phenol saturated with Tris 100 mM pH 8. The suspensions were vortexed for 30′ at 4 °C, followed by centrifugation (30 min at 4 °C at 14,000 g). The upper phases were removed and the lower phenol phases were precipitated with 5 volumes of 0.1 M ammonium acetate overnight at −20 °C. The precipitated proteins, obtained after a centrifugation of 30′ at 4 °C at 14,000 g, were washed with 5 volumes of 100% chilled methanol and then with 5 volumes of 100% chilled acetone. The proteins obtained from each method were solubilized and pooled after the trypsin digestion using the FASP protocol^48^. The peptide concentration was measured with a NanoDrop (Thermo Scientific) before the sample was stored at −20 °C.

### Mass spectrometry analysis

Samples were cleaned up on a C18 SPE column (Thermo Fisher Scientific, San Jose, CA, USA). Half of each sample was analyzed using a LTQ-Orbitrap XL mass spectrometer (Thermo Fisher Scientific, San Jose, CA, USA) coupled to an EasyLC (Thermo Fisher Scientific (Proxeon), Odense, Denmark). Peptides were loaded directly onto the analytical column at a flow rate of 1.5–2 μl/min using a wash-volume of 4 times the injection volume, and were separated by reversed-phase chromatography using a 25-cm column with an inner diameter of 75 μm, packed with 5 μm C18 particles (Nikkyo Technos Co., Ltd. Japan). Chromatographic gradients started at 93% buffer A and 7% buffer B for 4 minutes with a flow rate of 300 nl/min, in 1 minute increased to 95% buffer A and then gradually increased to 65% buffer A and 35% buffer B in 120 min. After each analysis, the column was washed for 10 min with 10% buffer A and 90% buffer B (Buffer A: 0.1% formic acid in water; Buffer B: 0.1% formic acid in acetonitrile).

The mass spectrometer was operated in positive ionization mode with nanospray voltage set at 2.5 kV and source temperature at 200 °C. Ultramark 1621 for the FT mass analyzer was used for external calibration prior the analyses. Moreover, an internal calibration was also performed using the background polysiloxane ion signal at m/z 445.1200. The instrument was operated in DDA mode and full MS scans with 1 micro scans at resolution of 60,000 were used over a mass range of m/z 350–1500 with detection in the Orbitrap. Auto gain control (AGC) was set to 1E6, and dynamic exclusion (60 seconds) and charge state filtering disqualifying singly charged peptides were both activated. In each cycle of DDA analysis, following each survey scan the top twelve most intense ions with multiple charged ions above a threshold ion count of 5000 were selected for fragmentation at normalized collision energy of 35%. Fragment ion spectra produced via collision-induced dissociation were acquired in the ion trap, AGC was set to 5E4, and isolation window of 2.0 m/z and maximum injection time of 50 ms was used. All data were acquired with Xcalibur software v2.2.

### Data Analysis

Proteome Discoverer software suite (v1.4, Thermo Fisher Scientific) and the Mascot search engine (v2.5, Matrix Science^49^) were used for peptide identification and quantification. The data were searched against two databases: a database built on the metagenomics datasets selected on the bases of the literature concerning the organisms identified in the same soils^11^,^12^,^14^,^15^ and a *T. melanosporum* database from MycorWeb (version of February 2015; 12826 sequences (http://mycor.nancy.inra.fr/IMGC/TuberGenome/download.php?select=fast). A list of common contaminants was added to the databases. Trypsin was chosen as the enzyme and a maximum of two miscleavages were allowed. Carbamidomethylation (C) was set as a fixed modification, whereas oxidation (M) and acetylation (N-terminal) were used as variable modifications. Searches were performed using a peptide tolerance of 10 ppm and a product ion tolerance of 0.6 Da. Gene Ontology (GO) and Kyoto Encyclopedia of Genes and Genomes (KEGG) analyses and Fisher Exact Test were performed by Blast2GO software^50^.

## Additional Information

**How to cite this article**: Zampieri, E. *et al*. Soil metaproteomics reveals an inter-kingdom stress response to the presence of black truffles. *Sci. Rep*. **6**, 25773; doi: 10.1038/srep25773 (2016).

## Supplementary Material

Supplementary Information

## Figures and Tables

**Figure 1:**
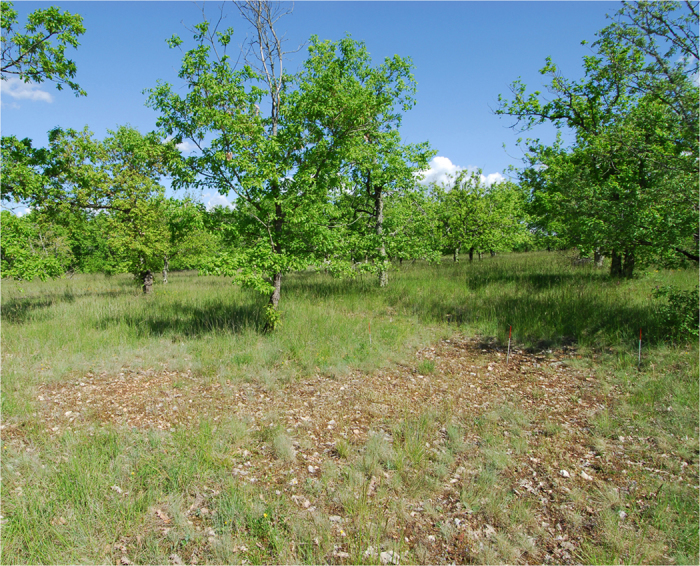
Picture of a brûlé in the Cahors truffle-ground.

**Table 1:** Processes over-represented inside (Test) the brûlé compared to the outside (Ref) based on Fisher Exact Test.

GO-ID	Term	Test	Ref	Over/Under
GO:0010035	response to inorganic substance	28	0	OVER
GO:0009628	response to abiotic stimulus	27	0	OVER
GO:0010038	response to metal ion	26	0	OVER
GO:0046686	response to cadmium ion	24	0	OVER
GO:0006970	response to osmotic stress	17	0	OVER
GO:0009651	response to salt stress	16	0	OVER
GO:0051603	proteolysis involved in cellular protein catabolic process	15	0	OVER
GO:0044257	cellular protein catabolic process	15	0	OVER
GO:0044265	cellular macromolecule catabolic process	15	0	OVER
GO:0030163	protein catabolic process	15	0	OVER
GO:0006790	sulphur compound metabolic process	14	0	OVER
GO:0009266	response to temperature stimulus	13	0	OVER
GO:0044272	sulphur compound biosynthetic process	13	0	OVER
GO:0048731	system development	13	0	OVER
GO:0008652	cellular amino acid biosynthetic process	19	1	OVER
GO:0006996	organelle organization	19	1	OVER
GO:0032501	multicellular organismal process	18	1	OVER
GO:0044707	single + AC0-multicellular organism process	18	1	OVER
GO:0007275	multicellular organismal development	18	1	OVER
GO:1901607	alpha + AC0-amino acid biosynthetic process	17	1	OVER
GO:0010033	response to organic substance	16	1	OVER
GO:0009057	macromolecule catabolic process	16	1	OVER
GO:1901700	response to oxygen + AC0-containing compound	16	1	OVER
GO:0032502	developmental process	21	2	OVER
GO:0046365	monosaccharide catabolic process	20	2	OVER
GO:0044724	single + AC0-organism carbohydrate catabolic process	20	2	OVER
GO:0006007	glucose catabolic process	20	2	OVER
GO:0019320	hexose catabolic process	20	2	OVER
GO:0006950	response to stress	42	3	OVER
GO:0042221	response to chemical stimulus	36	3	OVER
GO:0006006	glucose metabolic process	27	3	OVER
GO:0006520	cellular amino acid metabolic process	27	3	OVER
GO:0044711	single + AC0-organism biosynthetic process	26	3	OVER
GO:0044283	small molecule biosynthetic process	25	3	OVER
GO:0016053	organic acid biosynthetic process	22	3	OVER
GO:1901605	alpha + AC0-amino acid metabolic process	22	3	OVER
GO:0046394	carboxylic acid biosynthetic process	22	3	OVER
GO:0016052	carbohydrate catabolic process	20	3	OVER
GO:1901566	organonitrogen compound biosynthetic process	29	5	OVER
GO:0019318	hexose metabolic process	28	5	OVER
GO:0016043	cellular component organization	25	5	OVER
GO:0006082	organic acid metabolic process	35	7	OVER
GO:0043436	oxoacid metabolic process	34	7	OVER
GO:0019752	carboxylic acid metabolic process	33	7	OVER
GO:0071840	cellular component organization or biogenesis	30	7	OVER
GO:0005996	monosaccharide metabolic process	29	7	OVER
GO:0044267	cellular protein metabolic process	49	9	OVER
GO:0050896	response to stimulus	63	17	OVER
GO:1901575	organic substance catabolic process	48	17	OVER
GO:0009056	catabolic process	54	18	OVER
GO:1901564	organonitrogen compound metabolic process	58	19	OVER
GO:0019538	protein metabolic process	53	20	OVER
GO:0044281	small molecule metabolic process	65	23	OVER
GO:0044237	cellular metabolic process	142	76	OVER
GO:0044238	primary metabolic process	138	79	OVER
GO:0071704	organic substance metabolic process	146	82	OVER
GO:0009987	cellular process	153	85	OVER
