# Supplementary material for: Soil metaproteomics reveals an inter-kingdom stress response to the presence of black truffles
Source: Sci Rep. 2016 May 10;6:25773. doi: 10.1038/srep25773 (PMC4861934; doi:10.1038/srep25773)
Supplement: Supplementary Information [file srep25773-s1.pdf]

Soil metaproteomics reveals an inter-kingdom stress response to the presence of black truffles

Elisa Zampieri[1+], Marco Chiapello[1+], Stefania Daghino[1], Paola Bonfante[1], Antonietta Mello[2*]

[1]Department of Life Sciences and Systems Biology, University of Torino, Viale P.A. Mattioli 25, I-10125 Torino, Italy

[2]Institute for Sustainable Plant Protection, CNR, Torino Unit, Viale P.A. Mattioli 25, I-10125 Torino, Italy

***Corresponding author**

+ these authors contributed equally to the work

Supplementary Table S1: Proteins identified in the database constructed for this study.

Accession (indicates the protein ID), gi (indicates the protein Gene Index number from NCBI), UNIPROT (indicates the ID from UNIPROT website), desc (indicates the protein description), Description (indicates the origin of the sequences), ∑Coverage (indicates the total sum of all coverages), ∑#Proteins (indicates the total sum of all proteins identified), ∑#Unique Peptides (indicates the total sum of all unique peptides), ∑#Peptides (indicates the total sum of all identified peptides), ∑#PSMs (indicates the total sum of all the peptide-spectrum match), score (indicates the MASCOT score), #AAs (indicates the protein length), MW (indicates the protein weight), calc pI (indicates the calculated pI).

Accession	Description	ΣCoverage	Σ# Proteins	Σ# Unique Peptides	Σ# Peptides	Σ# PSMs
A0A015J624	Actin OS=Rhizophagus irregularis DAOM 197198w GN=RirG_137620 PE=3 SV=1 - [A0A015J624_9GLOM]	16.80	239	2	6	62
C4MMH6	Actin (Fragment) OS=Mortierella indohii PE=3 SV=1 - [C4MMH6_9FUNG]	23.05	173	1	4	43
Q6UDA0	Actin OS=Trifolium pratense PE=2 SV=1 - [Q6UDA0_TRIPR]	18.30	62	1	5	35
A0A031M2U0	60 kDa chaperonin OS=Pseudomonas bauzanensis GN=groL PE=3 SV=1 - [A0A031M2U0_9PSED]	11.46	62	1	7	24
U2ZLC7	60 kDa chaperonin OS=Pseudomonas alcaligenes NBRC 14159 GN=groL PE=3 SV=1 - [U2ZLC7_PSEAC]	6.96	192	1	4	20
A0A031M2A5	60 kDa chaperonin OS=Pseudomonas bauzanensis GN=groL PE=3 SV=1 - [A0A031M2A5_9PSED]	9.78	46	0	6	20
A0A031M511	60 kDa chaperonin OS=Pseudomonas bauzanensis GN=groL PE=3 SV=1 - [A0A031M511_9PSED]	12.82	20	1	7	20
D5G725	Whole genome shotgun sequence assembly, scaffold_13, strain Mel28 OS=Tuber melanosporum (strain Mel28) GN=GSTUM_00004570001 PE=4 SV=1 - [D5G725_TUBMM]	24.18	1	3	3	19
Q1AXU6	60 kDa chaperonin OS=Rubrobacter xylanophilus (strain DSM 9941 / NBRC 16129) GN=groL PE=3 SV=1 - [CH60_RUBXD]	10.00	179	5	6	16
E7BCR3	Calmodulin (Fragment) OS=Aspergillus lentulus GN=caM PE=4 SV=1 - [E7BCR3_9EURO]	39.74	389	4	4	13
D8PD19	60 kDa chaperonin OS=Candidatus Nitrospira defluvii GN=groL PE=3 SV=1 - [D8PD19_9BACT]	5.68	4	1	4	13
D9J011	Putative actin protein OS=Salvia miltiorrhiza PE=2 SV=1 - [D9J011_SALMI]	13.53	42	1	4	11
A0A097NU89	Putative heat shock protein (Fragment) OS=Taraxacum brevicorniculatum GN=HSP PE=2 SV=1 - [A0A097NU89_9ASTR]	12.54	297	4	6	11
D2S4D2	PQQ-dependent dehydrogenase, methanol/ethanol family OS=Geodermatophilus obscurus (strain ATCC 25078 / DSM 43160 / JCM 3152 / G-20) GN=Gobs_2451 PE=4 SV=1 - [D2S4D2_GEOOG]	6.42	1	3	3	10
F8CQR1	60 kDa chaperonin OS=Myxococcus fulvus (strain ATCC BAA-855 / HW-1) GN=groL PE=3 SV=1 - [F8CQR1_MYXFH]	6.23	162	1	4	10
N1V613	60 kDa chaperonin OS=Arthrobacter crystallopoietes BAB-32 GN=groL PE=3 SV=1 - [N1V613_9MICC]	4.49	9	1	3	10
X5C9W2	Glyceraldehyde-3-phosphate dehydrogenase (Fragment) OS=Sedum nussbaumerianum GN=GapC PE=3 SV=1 - [X5C9W2_9MAGN]	26.13	22	1	4	10

Accession	Description	ΣCoverage	Σ# Proteins	Σ# Unique Peptides	Σ# Peptides	Σ# PSMs
D2B144	Formamidase OS=Streptosporangium roseum (strain ATCC 12428 / DSM 43021 / JCM 3005 / NI 9100) GN=Sros_0422 PE=4 SV=1 - [D2B144_STRRD]	13.73	1	4	5	9
D5G530	Whole genome shotgun sequence assembly, scaffold_108, strain Mel28 OS=Tuber melanosporum (strain Mel28) GN=GSTUM_00000288001 PE=3 SV=1 - [D5G530_TUBMM]	10.99	342	1	5	9
A0A0D9N6M8	Hsp70 protein OS=Aspergillus flavus AF70 GN=P034_05357812 PE=3 SV=1 - [A0A0D9N6M8_ASPFL]	11.13	343	1	5	9
P42653	14-3-3-like protein A OS=Vicia faba PE=2 SV=1 - [1433A_VICFA]	22.99	75	3	6	9
U3MV00	NAD+-dependent glyceraldehyde-3-phosphate dehydrogenase (Fragment) OS=Taraxacum officinale GN=GAPC-2 PE=3 SV=1 - [U3MV00_TAROF]	26.50	29	3	6	9
A0A023X6K3	Formaldehyde dehydrogenase, glutathione-independent OS=Rubrobacter radiotolerans GN=RradSPS_2573 PE=3 SV=1 - [A0A023X6K3_9ACTN]	11.19	16	2	3	8
Q1D2S1	60 kDa chaperonin 2 OS=Myxococcus xanthus (strain DK 1622) GN=groL2 PE=3 SV=1 - [CH602_MYXXD]	7.10	5	1	4	8
A0A0A2C600	60 kDa chaperonin OS=Prochlorococcus marinus str. PAC1 GN=groL PE=3 SV=1 - [A0A0A2C600_PROMR]	3.26	53	1	2	8
C2ZER9	Uncharacterized protein OS=Bacillus cereus AH1272 GN=bcere0029_47110 PE=4 SV=1 - [C2ZER9_BACCE]	7.63	4	1	1	8
F4CRF7	Formamidase OS=Pseudonocardia dioxanivorans (strain ATCC 55486 / DSM 44775 / JCM 13855 / CB1190) GN=Psed_3049 PE=4 SV=1 - [F4CRF7_PSEUX]	7.67	27	3	3	7
C0VYK7	Glycerol kinase OS=Actinomyces coleocanis DSM 15436 GN=glpK PE=3 SV=1 - [C0VYK7_9ACTO]	6.17	7	3	3	7
A0A023X255	Glycerol kinase OS=Rubrobacter radiotolerans GN=glpK PE=3 SV=1 - [A0A023X255_9ACTN]	8.09	1	3	3	7
F8CAY9	60 kDa chaperonin OS=Myxococcus fulvus (strain ATCC BAA-855 / HW-1) GN=groL PE=3 SV=1 - [F8CAY9_MYXFH]	6.92	6	1	4	7
A0A0B4CW17	Adenosylhomocysteinase OS=Arthrobacter phenanthrenivorans GN=ahcY PE=3 SV=1 - [A0A0B4CW17_9MICC]	2.46	5	1	1	7
S6WAM9	MoxR protein (Fragment) OS=Pseudomonas syringae pv. actinidiae ICMP 19096 GN=A245_15202 PE=4 SV=1 - [S6WAM9_PSESF]	7.06	133	1	1	7
A0A0D0V2Q0	Adenosylhomocysteinase OS=Micromonospora carbonacea GN=ahcY PE=3 SV=1 - [A0A0D0V2Q0_9ACTN]	4.61	36	2	2	6
A0A024QIB3	S-(Hydroxymethyl)glutathione dehydrogenase OS=Mycobacterium neoaurum GN=BN1047_00150 PE=3 SV=1 - [A0A024QIB3_MYCNE]	6.68	21	1	2	6
A0A031LYZ6	ATP synthase subunit beta OS=Pseudomonas bauzanensis GN=atpD PE=3 SV=1 - [A0A031LYZ6_9PSED]	6.54	229	1	2	6

Accession	Description	ΣCoverage	Σ# Proteins	Σ# Unique Peptides	Σ# Peptides	Σ# PSMs
R0IPS1	Uncharacterized protein OS=Capsella rubella GN=CARUB_v10012149mg PE=3 SV=1 - [R0IPS1_9BRAS]	6.62	293	1	3	6
A0A086WFH7	Elongation factor Tu (Fragment) OS=Massilia consociata GN=tuf PE=4 SV=1 - [A0A086WFH7_9BURK]	13.79	5	1	1	6
H0QKE9	Putative uncharacterized protein OS=Arthrobacter globiformis NBRC 12137 GN=ARGLB_037_02400 PE=4 SV=1 - [H0QKE9_ARTGO]	4.71	3	1	1	6
D2X5N3	Glyceraldehyde-3-phosphate dehydrogenase (Fragment) OS=Artemisia annua PE=2 SV=1 - [D2X5N3_ARTAN]	22.73	17	2	4	6
P31926	Sucrose synthase OS=Vicia faba GN=SUCS PE=2 SV=1 - [SUSY_VICFA]	5.58	2	3	3	6
Q2PEX3	Putative HEAT SHOCK PROTEIN 81-2 (Fragment) OS=Trifolium pratense PE=2 SV=1 - [Q2PEX3_TRIPR]	11.51	133	4	5	6
K3VXF8	Uncharacterized protein OS=Fusarium pseudograminearum (strain CS3096) GN=FPSE_10991 PE=4 SV=1 - [K3VXF8_FUSPC]	0.38	1	1	1	5
D2SGA0	Malate dehydrogenase OS=Geodermatophilus obscurus (strain ATCC 25078 / DSM 43160 / JCM 3152 / G-20) GN=mdh PE=3 SV=1 - [D2SGA0_GEOOG]	8.20	9	2	2	5
A0A023X1G9	Sulfurtransferase OS=Rubrobacter radiotolerans GN=RradSPS_0885 PE=4 SV=1 - [A0A023X1G9_9ACTN]	7.56	1	1	1	5
A0A0F0HPK1	Aconitate hydratase OS=Saccharothrix sp. ST-888 GN=acnA PE=3 SV=1 - [A0A0F0HPK1_9PSEU]	4.31	162	2	3	5
G3FEZ4	Elongation factor Tu (Fragment) OS=Mycobacterium conceptionense GN=tuf PE=4 SV=1 - [G3FEZ4_9MYCO]	5.83	1	1	1	5
I3S5Q3	Uncharacterized protein OS=Lotus japonicus PE=2 SV=1 - [I3S5Q3_LOTJA]	17.76	75	2	5	5
Q684J8	Glutamate decarboxylase OS=Lotus japonicus GN=gad1 PE=2 SV=1 - [Q684J8_LOTJA]	5.71	6	2	2	5
D5GF49	Whole genome shotgun sequence assembly, scaffold_296, strain Mel28 OS=Tuber melanosporum (strain Mel28) GN=GSTUM_00001850001 PE=4 SV=1 - [D5GF49_TUBMM]	37.04	1	3	3	5
A1UHF2	Amino acid/amide ABC transporter substrate-binding protein, HAAT family OS=Mycobacterium sp. (strain KMS) GN=Mkms_3066 PE=4 SV=1 - [A1UHF2_MYCSK]	6.20	1	2	2	4
N1V4B6	Glutathione-independent formaldehyde dehydrogenase OS=Arthrobacter crystallopoietes BAB-32 GN=D477_007634 PE=3 SV=1 - [N1V4B6_9MICC]	6.19	15	1	2	4
Q07838	Acetamidase OS=Mycobacterium smegmatis GN=amdA PE=1 SV=2 - [AMDA_MYCSM]	6.40	4	1	2	4
R0HQP2	Uncharacterized protein OS=Capsella rubella GN=CARUB_v10024476mg PE=4 SV=1 - [R0HQP2_9BRAS]	4.90	6	2	2	4

Accession	Description	ΣCoverage	Σ# Proteins	Σ# Unique Peptides	Σ# Peptides	Σ# PSMs
E5WLJ3	Zinc alcohol dehydrogenase OS=Bacillus sp. 2_A_57_CT2 GN=HMPREF1013_03327 PE=3 SV=1 - [E5WLJ3_9BACI]	7.92	30	2	3	4
A0A066WMI9	Uncharacterized protein OS=Flavobacterium sp. EM1321 GN=FEM21_18330 PE=4 SV=1 - [A0A066WMI9_9FLAO]	1.98	2	1	1	4
X8BKS3	Isocitrate/isopropylmalate dehydrogenase family protein OS=Mycobacterium xenopi 3993 GN=I552_0311 PE=3 SV=1 - [X8BKS3_MYCXE]	18.06	49	1	1	4
A0A024M4M9	Heat shock protein Hsp20 OS=Mycobacterium farcinogenes GN=BN975_04407 PE=3 SV=1 - [A0A024M4M9_9MYCO]	7.69	4	1	1	4
A0A023X435	DNA-directed RNA polymerase subunit beta' OS=Rubrobacter radiotolerans GN=rpoC PE=3 SV=1 - [A0A023X435_9ACTN]	1.32	302	2	2	4
J2MVZ3	Outer membrane porin OprF OS=Pseudomonas fluorescens Q2-87 GN=oprF PE=3 SV=1 - [J2MVZ3_PSEFL]	11.63	2	1	4	4
I0L6Q5	Cellulose binding secreted glycosyl hydrolase OS=Micromonospora lupini str. Lupac 08 GN=MILUP08_44377 PE=4 SV=1 - [I0L6Q5_9ACTN]	0.61	1	1	1	4
B1PZ38	OmpF OS=Pseudomonas putida PE=3 SV=1 - [B1PZ38_PSEPU]	11.66	55	1	4	4
D5GIA8	Whole genome shotgun sequence assembly, scaffold_46, strain Mel28 OS=Tuber melanosporum (strain Mel28) GN=GSTUM_00008389001 PE=4 SV=1 - [D5GIA8_TUBMM]	7.35	1	2	2	4
A0A024HBV6	Putative amino-acid ABC transporter-binding protein yhdW OS=Pseudomonas knackmussii (strain DSM 6978 / LMG 23759 / B13) GN=yhdW PE=3 SV=1 - [A0A024HBV6_PSEKB]	9.65	15	3	3	4
U5IBT8	Ascorbate peroxidase (Fragment) OS=Trifolium repens GN=APX PE=2 SV=1 - [U5IBT8_TRIRP]	10.99	1	2	2	4
R0GZH8	Adenosylhomocysteinase OS=Capsella rubella GN=CARUB_v10004693mg PE=3 SV=1 - [R0GZH8_9BRAS]	6.39	68	3	3	4
R0HG12	Uncharacterized protein OS=Capsella rubella GN=CARUB_v10017235mg PE=3 SV=1 - [R0HG12_9BRAS]	6.87	12	1	3	4
I3SNN1	Uncharacterized protein OS=Lotus japonicus PE=2 SV=1 - [I3SNN1_LOTJA]	10.10	2	1	2	4
I3S3D3	Nucleoside diphosphate kinase OS=Lotus japonicus PE=2 SV=1 - [I3S3D3_LOTJA]	20.00	1	2	2	4
D5GLX2	Whole genome shotgun sequence assembly, scaffold_7, strain Mel28 OS=Tuber melanosporum (strain Mel28) GN=GSTUM_00010457001 PE=4 SV=1 - [D5GLX2_TUBMM]	14.29	1	1	1	4
I3T0F4	Fructose-bisphosphate aldolase OS=Lotus japonicus PE=2 SV=1 - [I3T0F4_LOTJA]	11.78	4	1	2	4
I3T8P4	Uncharacterized protein OS=Lotus japonicus PE=2 SV=1 - [I3T8P4_LOTJA]	12.15	1	1	1	4

Accession	Description	ΣCoverage	Σ# Proteins	Σ# Unique Peptides	Σ# Peptides	Σ# PSMs
C8VFF7	Putative Zn(II)2Cys6 transcription factor (Eurofung) OS=Emericella nidulans (strain FGSC A4 / ATCC 38163 / CBS 112.46 / NRRL 194 / M139) GN=ANIA_05775 PE=4 SV=1 - [C8VFF7_EMENI]	1.33	1	1	1	3
M2V5V0	Cytochrome c peroxidase OS=Pseudomonas stutzeri NF13 GN=B381_05316 PE=4 SV=1 - [M2V5V0_PSEST]	2.37	2	1	1	3
D2S8V2	Amino acid adenylation domain protein OS=Geodermatophilus obscurus (strain ATCC 25078 / DSM 43160 / JCM 3152 / G-20) GN=Gobs_3073 PE=4 SV=1 - [D2S8V2_GEOOG]	1.43	1	1	1	3
Q8GI57	Superoxide dismutase OS=Rubrobacter radiotolerans GN=sodA PE=3 SV=2 - [Q8GI57_9ACTN]	5.83	1	1	1	3
Q0CKI2	Glycerol kinase 2 OS=Aspergillus terreus (strain NIH 2624 / FGSC A1156) GN=ATEG_05802 PE=3 SV=1 - [Q0CKI2_ASPTN]	2.23	3	1	1	3
B5AEM9	6-phosphogluconate dehydrogenase, decarboxylating (Fragment) OS=Mycobacterium lepromatosis GN=gnd1 PE=3 SV=1 - [B5AEM9_9MYCO]	2.07	3	1	1	3
U2AB71	Glucose-6-phosphate isomerase OS=Bacillus sp. EGD-AK10 GN=pgi PE=3 SV=1 - [U2AB71_9BACI]	2.26	1	1	1	3
L8TPB7	Glycerol kinase OS=Arthrobacter nitrophenolicus GN=glpK PE=3 SV=1 - [L8TPB7_9MICC]	3.17	1	1	1	3
A0A099CPS9	RNA polymerase sigma24 factor OS=Mycobacterium rufum GN=EU78_21860 PE=4 SV=1 - [A0A099CPS9_9MYCO]	2.72	3	1	1	3
A0A031MCA3	Amino acid ABC transporter substrate-binding protein OS=Pseudomonas bauzanensis GN=CF98_32735 PE=4 SV=1 - [A0A031MCA3_9PSED]	3.49	1	2	2	3
X8FJG5	Formylglycine-generating sulfatase enzyme family protein OS=Mycobacterium ulcerans str. Harvey GN=I551_1306 PE=4 SV=1 - [X8FJG5_MYCUL]	10.62	96	1	1	3
A0A0D0WZL0	Sulfurtransferase OS=Micromonospora carbonacea GN=TK50_01705 PE=4 SV=1 - [A0A0D0WZL0_9ACTN]	4.67	5	1	1	3
A0A074TMY9	Aconitate hydratase (Fragment) OS=Microbacterium sp. SUBG005 GN=HR12_20220 PE=4 SV=1 - [A0A074TMY9_9MICO]	15.25	234	1	2	3
W7VQP0	D-xylulose 5-phosphate/D-fructose 6-phosphate phosphoketolase OS=Micromonospora sp. M42 GN=MCBG_00088 PE=4 SV=1 - [W7VQP0_9ACTN]	2.60	5	2	2	3
F3EXH9	ABC transporter periplasmic substrate-binding protein OS=Pseudomonas amygdali pv. mori str. 301020 GN=PSYMO_14635 PE=4 SV=1 - [F3EXH9_PSEA0]	4.44	132	2	2	3
A0A0F7P5R1	Superoxide dismutase OS=Elizabethkingia meningoseptica FMS-007 GN=M876_16000 PE=4 SV=1 - [A0A0F7P5R1_ELIME]	5.56	9	1	1	3
I3T598	Uncharacterized protein OS=Lotus japonicus PE=2 SV=1 - [I3T598_LOTJA]	4.79	5	1	1	3

Accession	Description	ΣCoverage	Σ# Proteins	Σ# Unique Peptides	Σ# Peptides	Σ# PSMs
A0A0E4CR92	Uncharacterized protein OS=Mycobacterium lentiflavum GN=BN1232_06067 PE=4 SV=1 - [A0A0E4CR92_9MYCO]	8.86	2	1	1	3
A0A0A1Z7Q1	SSU ribosomal protein S19p (S15e) OS=Prochlorococcus marinus str. GP2 GN=EU91_1720 PE=3 SV=1 - [A0A0A1Z7Q1_PROMR]	16.33	157	1	1	3
X7Z574	Uncharacterized protein OS=Mycobacterium xenopi 4042 GN=I553_7048 PE=4 SV=1 - [X7Z574_MYCXE]	5.56	2	1	1	3
W9N0A1	Uncharacterized protein OS=Fusarium oxysporum f. sp. lycopersici MN25 GN=FOWG_00222 PE=4 SV=1 - [W9N0A1_FUSOX]	1.99	10	1	1	3
U2SUS3	TolA protein OS=Myxococcus sp. (contaminant ex DSM 436) GN=A176_00378 PE=4 SV=1 - [U2SUS3_9DELT]	1.73	1	1	1	3
A0A0C2I3T2	Histidine kinase OS=Pseudomonas batumici GN=UCMB321_2298 PE=4 SV=1 - [A0A0C2I3T2_9PSED]	2.67	1	1	1	3
L8AJT4	Adenosylhomocysteinase OS=Bacillus subtilis BEST7613 GN=ahcY PE=3 SV=1 - [L8AJT4_BACIU]	3.06	1	1	1	3
D5GIK5	Whole genome shotgun sequence assembly, scaffold_48, strain Mel28 OS=Tuber melanosporum (strain Mel28) GN=GSTUM_00008539001 PE=4 SV=1 - [D5GIK5_TUBMM]	3.01	1	2	2	3
D5GLX7	Whole genome shotgun sequence assembly, scaffold_7, strain Mel28 OS=Tuber melanosporum (strain Mel28) GN=GSTUM_00010465001 PE=4 SV=1 - [D5GLX7_TUBMM]	11.22	1	1	1	3
I3SZE9	Uncharacterized protein OS=Lotus japonicus PE=2 SV=1 - [I3SZE9_LOTJA]	16.11	8	2	2	3
R0G5L8	Uncharacterized protein OS=Capsella rubella GN=CARUB_v10013828mg PE=3 SV=1 - [R0G5L8_9BRAS]	10.44	20	3	3	3
R0GR46	Uncharacterized protein OS=Capsella rubella GN=CARUB_v10000282mg PE=3 SV=1 - [R0GR46_9BRAS]	3.92	2	3	3	3
Q58ZF1	Beta-tubulin (Fragment) OS=Lotus corniculatus PE=2 SV=1 - [Q58ZF1_LOTCO]	8.35	15	1	3	3
R0F5B5	Uncharacterized protein OS=Capsella rubella GN=CARUB_v10004805mg PE=3 SV=1 - [R0F5B5_9BRAS]	4.49	1	1	2	3
R0INE5	Uncharacterized protein OS=Capsella rubella GN=CARUB_v10010553mg PE=3 SV=1 - [R0INE5_9BRAS]	27.63	6	3	3	3
A0A059XMU8	Cysteine synthase OS=Lathyrus sativus GN=Bsas3;1 PE=2 SV=1 - [A0A059XMU8_LATSA]	8.66	1	2	3	3
D2BBD1	60 kDa chaperonin OS=Streptosporangium roseum (strain ATCC 12428 / DSM 43021 / JCM 3005 / NI 9100) GN=groL PE=3 SV=1 - [D2BBD1_STRRD]	4.07	5	1	2	3
Q1AUM3	Alcohol dehydrogenase GroES-like protein OS=Rubrobacter xylanophilus (strain DSM 9941 / NBRC 16129) GN=Rxyl_1958 PE=3 SV=1 - [Q1AUM3_RUBXD]	2.27	2	1	1	2

Accession	Description	ΣCoverage	Σ# Proteins	Σ# Unique Peptides	Σ# Peptides	Σ# PSMs
U3HB76	ADP-heptose:LPS heptosyl transferase OS=Pseudomonas alcaligenes OT 69 GN=L682_15865 PE=4 SV=1 - [U3HB76_PSEAC]	3.14	1	1	1	2
A0A081I584	Peptidyl-prolyl cis-trans isomerase OS=Mycobacterium sp. TKK-01-0059 GN=K883_01847 PE=3 SV=1 - [A0A081I584_9MYCO]	7.95	32	1	1	2
J3AB25	Transcriptional regulator OS=Pseudomonas sp. GM48 GN=PMI28_00382 PE=4 SV=1 - [J3AB25_9PSED]	2.70	1	1	1	2
L0IVW3	Urocanate hydratase OS=Mycobacterium smegmatis JS623 GN=hutU PE=3 SV=1 - [L0IVW3_MYCSM]	4.52	40	2	2	2
A0A0D7FCN8	Sugar ABC transporter OS=Pseudomonas oryzihabitans GN=UM91_16390 PE=4 SV=1 - [A0A0D7FCN8_9PSED]	4.39	2	1	1	2
Q1AZT7	Xanthine dehydrogenase, molybdenum binding subunit apoprotein OS=Rubrobacter xylanophilus (strain DSM 9941 / NBRC 16129) GN=Rxyl_0112 PE=4 SV=1 - [Q1AZT7_RUBXD]	2.83	2	2	2	2
A4VIS3	Uncharacterized protein OS=Pseudomonas stutzeri (strain A1501) GN=PST_1179 PE=3 SV=1 - [A4VIS3_PSEU5]	13.20	101	2	2	2
W4PWG4	Serine/threonine protein kinase PrkC OS=Bacillus wakoensis JCM 9140 GN=JCM9140_112 PE=4 SV=1 - [W4PWG4_9BACI]	1.20	4	1	1	2
A0A064CC06	ABC transporter substrate-binding protein OS=Mycobacterium aromaticivorans JS19b1 = JCM 16368 GN=Y900_002625 PE=4 SV=1 - [A0A064CC06_9MYCO]	4.18	14	2	2	2
A0A031M6T0	Amino acid ABC transporter substrate-bindnig protein OS=Pseudomonas bauzanensis GN=CF98_13305 PE=4 SV=1 - [A0A031M6T0_9PSED]	6.73	1	2	2	2
X8C966	Thiamine pyrophosphate enzyme, N-terminal TPP binding domain protein OS=Mycobacterium xenopi 3993 GN=I552_2955 PE=4 SV=1 - [X8C966_MYCXE]	7.82	164	1	1	2
A0A077LJN0	Formaldehyde dehydrogenase OS=Pseudomonas sp. StFLB209 GN=PSCI_1312 PE=3 SV=1 - [A0A077LJN0_9PSED]	3.76	2	1	1	2
A0A023X481	Malate synthase OS=Rubrobacter radiotolerans GN=RradSPS_1855 PE=3 SV=1 - [A0A023X481_9ACTN]	5.59	1	2	2	2
A0A0F0H9G5	LytR family transcriptional regulator (Fragment) OS=Saccharothrix sp. ST-888 GN=UK12_33610 PE=4 SV=1 - [A0A0F0H9G5_9PSEU]	6.50	2	1	1	2
X8FHU5	EPTC-inducible aldehyde dehydrogenase OS=Mycobacterium ulcerans str. Harvey GN=I551_0830 PE=3 SV=1 - [X8FHU5_MYCUL]	4.44	13	1	1	2
A0A024EJ81	Uncharacterized protein OS=Pseudomonas mandelii JR-1 GN=OU5_5773 PE=4 SV=1 - [A0A024EJ81_9PSED]	13.19	47	1	1	2
A0A0F7BTD3	Uncharacterized protein OS=Myxococcus fulvus 124B02 GN=MFUL124B02_31275 PE=4 SV=1 - [A0A0F7BTD3_MYXFU]	5.02	1	1	1	2
D2B9Y7	Glycerone kinase OS=Streptosporangium roseum (strain ATCC 12428 / DSM 43021 / JCM 3005 / NI 9100) GN=Sros_3048 PE=4 SV=1 - [D2B9Y7_STRRD]	2.12	25	1	1	2

Accession	Description	ΣCoverage	Σ# Proteins	Σ# Unique Peptides	Σ# Peptides	Σ# PSMs
W4Q7M1	Glycerol kinase OS=Bacillus wakoensis JCM 9140 GN=JCM9140_4266 PE=3 SV=1 - [W4Q7M1_9BACI]	2.22	1	1	1	2
A0A023WYP6	Hydantase: amidase, hydantoinase/carbamoylase family OS=Rubrobacter radiotolerans GN=RradSPS_0069 PE=4 SV=1 - [A0A023WYP6_9ACTN]	2.66	2	1	1	2
C7YL24	Putative uncharacterized protein OS=Nectria haematococca (strain 77-13-4 / ATCC MYA-4622 / FGSC 9596 / MPVI) GN=NECHADRAFT_99492 PE=4 SV=1 - [C7YL24_NECH7]	3.14	1	1	1	2
G4I7D7	Peptidase M48 Ste24p OS=Mycobacterium rhodesiae JS60 GN=MycrhDRAFT_6000 PE=4 SV=1 - [G4I7D7_MYCRH]	1.39	1	1	1	2
C7Z1H4	Putative uncharacterized protein OS=Nectria haematococca (strain 77-13-4 / ATCC MYA-4622 / FGSC 9596 / MPVI) GN=NECHADRAFT_86153 PE=4 SV=1 - [C7Z1H4_NECH7]	4.00	1	1	1	2
B2B374	Podospora anserina S mat+ genomic DNA chromosome 6, supercontig 2 OS=Podospora anserina (strain S / ATCC MYA-4624 / DSM 980 / FGSC 10383) GN=PODANS_6_820 PE=4 SV=1 - [B2B374_PODAN]	0.51	1	1	1	2
S6IZP4	30S ribosomal protein S3 OS=Pseudomonas sp. CF161 GN=rpsC PE=3 SV=1 - [S6IZP4_9PSED]	11.36	229	2	2	2
A0A0F5MQC0	Luciferase OS=Mycobacterium arupense GN=WR43_20880 PE=4 SV=1 - [A0A0F5MQC0_9MYCO]	3.64	1	1	1	2
F9F4B5	Uncharacterized protein (Fragment) OS=Fusarium oxysporum (strain Fo5176) GN=FOXB_01240 PE=4 SV=1 - [F9F4B5_FUSOF]	4.94	1	1	1	2
A0A085FS16	Formate dehydrogenase-O, major subunit OS=Massilia sp. LC238 GN=FG94_01125 PE=4 SV=1 - [A0A085FS16_9BURK]	1.28	4	1	1	2
A0A0B4KEB7	Translation elongation factor EF1-alpha (Fragment) OS=Amanita mafingensis GN=tef1 PE=4 SV=1 - [A0A0B4KEB7_9AGAR]	15.45	985	2	2	2
A0A017SQ96	Histone H4 (Fragment) OS=Aspergillus ruber CBS 135680 GN=EURHEDRAFT_447549 PE=3 SV=1 - [A0A017SQ96_9EURO]	25.29	33	2	2	2
A0A0D0EX19	Histidine kinase OS=Flavobacterium hibernum GN=IW18_15540 PE=4 SV=1 - [A0A0D0EX19_9FLAO]	0.66	1	1	1	2
A0A0F0HX18	Uncharacterized protein OS=Saccharothrix sp. ST-888 GN=UK12_00790 PE=4 SV=1 - [A0A0F0HX18_9PSEU]	1.31	1	1	1	2
A0A085FJB3	General stress protein OS=Massilia sp. LC238 GN=FG94_02347 PE=4 SV=1 - [A0A085FJB3_9BURK]	10.71	3	1	1	2
G9G3Y9	Beta-tubulin (Fragment) OS=Fusarium cf. dimerum 6-a DPGS-2011 PE=3 SV=1 - [G9G3Y9_9HYPO]	3.36	1	1	1	2
A0A0A1CW01	Uncharacterized protein OS=Arthrobacter sp. PAMC25486 GN=ART_1828 PE=4 SV=1 - [A0A0A1CW01_9MICC]	4.11	1	1	1	2
K0XAB6	VirD4 OS=Pseudomonas fluorescens R124 GN=virD4 PE=4 SV=1 - [K0XAB6_PSEFL]	2.16	1	1	1	2

Accession	Description	ΣCoverage	Σ# Proteins	Σ# Unique Peptides	Σ# Peptides	Σ# PSMs
U1S6V9	Chemotaxis protein OS=Pseudomonas mendocina EGD-AQ5 GN=O203_20480 PE=4 SV=1 - [U1S6V9_PSEME]	1.78	1	1	1	2
I0LBY2	Xylose binding protein transport system OS=Micromonospora lupini str. Lupac 08 GN=MILUP08_46224 PE=4 SV=1 - [I0LBY2_9ACTN]	2.47	5	1	1	2
N1VC98	Glyceraldehyde-3-phosphate dehydrogenase OS=Arthrobacter crystallopoietes BAB-32 GN=D477_001879 PE=3 SV=1 - [N1VC98_9MICC]	4.46	2	1	1	2
D5GAH8	Whole genome shotgun sequence assembly, scaffold_181, strain Mel28 OS=Tuber melanosporum (strain Mel28) GN=GSTUM_00003593001 PE=4 SV=1 - [D5GAH8_TUBMM]	9.50	1	1	1	2
D5GHM3	Whole genome shotgun sequence assembly, scaffold_41, strain Mel28 OS=Tuber melanosporum (strain Mel28) GN=GSTUM_00008041001 PE=4 SV=1 - [D5GHM3_TUBMM]	11.74	1	1	1	2
W7ZA32	ATP synthase subunit beta OS=Bacillus sp. JCM 19046 GN=atpD PE=3 SV=1 - [W7ZA32_9BACI]	5.32	233	1	2	2
G2Z5G4	Uncharacterized protein OS=Flavobacterium branchiophilum (strain FL-15) GN=FBFL15_0567 PE=4 SV=1 - [G2Z5G4_FLABF]	1.81	1	1	1	2
J3C4Y2	Beta-glucosidase-like glycosyl hydrolase OS=Flavobacterium sp. CF136 GN=PMI10_01979 PE=3 SV=1 - [J3C4Y2_9FLAO]	2.15	3	2	2	2
I3SH51	Uncharacterized protein OS=Lotus japonicus PE=2 SV=1 - [I3SH51_LOTJA]	2.99	3	2	2	2
R0GU86	Uncharacterized protein (Fragment) OS=Capsella rubella GN=CARUB_v100044650mg PE=4 SV=1 - [R0GU86_9BRAS]	12.10	11	2	2	2
R0GKE5	Uncharacterized protein OS=Capsella rubella GN=CARUB_v10028626mg PE=4 SV=1 - [R0GKE5_9BRAS]	4.77	2	2	2	2
R0HKB9	Uncharacterized protein (Fragment) OS=Capsella rubella GN=CARUB_v10013241mg PE=3 SV=1 - [R0HKB9_9BRAS]	3.89	1	1	2	2
R0FNU6	Fructose-bisphosphate aldolase (Fragment) OS=Capsella rubella GN=CARUB_v10017399mg PE=3 SV=1 - [R0FNU6_9BRAS]	6.31	4	1	2	2
Q9XHC7	Phosphoenol pyruvate carboxylase OS=Lotus corniculatus PE=2 SV=1 - [Q9XHC7_LOTCO]	1.04	7	1	1	2
R0FV42	Uncharacterized protein OS=Capsella rubella GN=CARUB_v10022887mg PE=3 SV=1 - [R0FV42_9BRAS]	4.63	1	1	2	2
R0GRF1	Uncharacterized protein OS=Capsella rubella GN=CARUB_v10010247mg PE=4 SV=1 - [R0GRF1_9BRAS]	5.45	3	1	1	2
D8PIF0	Uncharacterized protein OS=Candidatus Nitrospira defluvii GN=NIDE3350 PE=4 SV=1 - [D8PIF0_9BACT]	9.33	1	1	1	2
R0FX04	Uncharacterized protein OS=Capsella rubella GN=CARUB_v10023630mg PE=3 SV=1 - [R0FX04_9BRAS]	4.62	2	1	1	2
E3Q1S2	Putative isocitrate dehydrogenase (Fragment) OS=Quercus pubescens GN=IDH (NADP+) PE=3 SV=1 - [E3Q1S2_9ROSI]	17.81	7	1	1	2

Accession	Description	ΣCoverage	Σ# Proteins	Σ# Unique Peptides	Σ# Peptides	Σ# PSMs
R0FHR7	Uncharacterized protein OS=Capsella rubella GN=CARUB_v10001942mg PE=3 SV=1 - [R0FHR7_9BRAS]	9.72	15	2	2	2
I3T4F6	Uncharacterized protein OS=Lotus japonicus PE=2 SV=1 - [I3T4F6_LOTJA]	4.04	1	1	1	2
I3T5V4	Uncharacterized protein OS=Lotus japonicus PE=2 SV=1 - [I3T5V4_LOTJA]	3.85	2	1	1	2
Q2PEW2	Putative cytoplasmic aconitate hydratase (Fragment) OS=Trifolium pratense PE=2 SV=1 - [Q2PEW2_TRIPR]	8.86	1	2	2	2
I3T2W0	Uncharacterized protein OS=Lotus japonicus PE=2 SV=1 - [I3T2W0_LOTJA]	12.32	6	2	2	2
Q3LVQ8	TO25-123 (Fragment) OS=Taraxacum officinale GN=To25-123 PE=2 SV=1 - [Q3LVQ8_TAROF]	29.85	7	1	1	2
M5R2U9	Hydrolase OS=Bacillus stratosphericus LAMA 585 GN=C883_1449 PE=4 SV=1 - [M5R2U9_9BACI]	2.76	2	1	1	2
Q6Q4Z3	LOS2 OS=Capsella bursa-pastoris GN=los2 PE=2 SV=1 - [Q6Q4Z3_CAPBU]	5.41	5	2	2	2
I3S0W6	Proteasome subunit alpha type OS=Lotus japonicus PE=2 SV=1 - [I3S0W6_LOTJA]	5.49	2	1	1	2
R0HNM5	Uncharacterized protein OS=Capsella rubella GN=CARUB_v10014518mg PE=4 SV=1 - [R0HNM5_9BRAS]	8.02	2	1	1	2
W7VC77	Uncharacterized protein OS=Micromonospora sp. M42 GN=MCBG_01046 PE=4 SV=1 - [W7VC77_9ACTN]	2.63	16	1	1	2
R0HKA2	Uncharacterized protein OS=Capsella rubella GN=CARUB_v10019000mg PE=4 SV=1 - [R0HKA2_9BRAS]	4.25	2	1	1	2
A0A075EAM3	Farnesyl diphosphate synthase OS=Astragalus membranaceus GN=FPS PE=2 SV=1 - [A0A075EAM3_ASTME]	6.43	1	2	2	2
R0I261	6-phosphogluconate dehydrogenase, decarboxylating OS=Capsella rubella GN=CARUB_v10013557mg PE=3 SV=1 - [R0I261_9BRAS]	4.12	14	1	2	2
D6PPS9	AT3G02360-like protein (Fragment) OS=Capsella grandiflora PE=4 SV=1 - [D6PPS9_9BRAS]	13.90	14	1	2	2
A0A059WQB8	Eukaryotic translation initiation factor 5A OS=Quercus suber PE=2 SV=1 - [A0A059WQB8_QUESU]	6.29	1	1	1	2
C2NS66	Uncharacterized protein OS=Bacillus cereus BGSC 6E1 GN=bcere0004_55930 PE=4 SV=1 - [C2NS66_BACCE]	3.83	1	1	1	2
A0A0A0E634	Multidrug transporter AcrB OS=Bacillus niacini GN=NP83_10025 PE=4 SV=1 - [A0A0A0E634_9BACI]	0.99	2	1	1	2
I3S9M6	Uncharacterized protein OS=Lotus japonicus PE=2 SV=1 - [I3S9M6_LOTJA]	12.22	95	1	1	2
U4L650	Similar to Heat shock protein 90 homolog acc. no. O43109 OS=Pyronema omphalodes (strain CBS 100304) GN=PCON_11855 PE=3 SV=1 - [U4L650_PYROM]	2.97	127	1	2	2

Accession	Description	ΣCoverage	Σ# Proteins	Σ# Unique Peptides	Σ# Peptides	Σ# PSMs
R0G9G5	Uncharacterized protein OS=Capsella rubella GN=CARUB_v10015564mg PE=3 SV=1 - [R0G9G5_9BRAS]	2.75	127	1	2	2
I3S358	Uncharacterized protein OS=Lotus japonicus PE=2 SV=1 - [I3S358_LOTJA]	3.25	4	1	1	2
I3S2A7	Uncharacterized protein OS=Lotus japonicus PE=2 SV=1 - [I3S2A7_LOTJA]	8.67	3	1	1	2
A0A0B7JX17	alpha-1,2-Mannosidase OS=Bionectria ochroleuca GN=BN869_000003091_1 PE=3 SV=1 - [A0A0B7JX17_BIOOC]	1.57	1	1	1	2
Q83XX0	Cytosine-specific methyltransferase OS=Arthrobacter sp. S GN=asiSIM PE=3 SV=1 - [Q83XX0_9MICC]	1.79	1	1	1	2
Q0REU0	Uncharacterized protein OS=Frankia alni (strain ACN14a) GN=FRAAL5383 PE=4 SV=1 - [Q0REU0_FRAAA]	2.30	2	1	1	2
S7ZEP9	Uncharacterized protein OS=Penicillium oxalicum (strain 114-2 / CGMCC 5302) GN=PDE_04094 PE=4 SV=1 - [S7ZEP9_PENO1]	5.90	1	1	1	2
G9QDX3	Uncharacterized protein OS=Bacillus sp. 7_6_55CFAA_CT2 GN=HMPREF1014_04865 PE=4 SV=1 - [G9QDX3_9BACI]	21.65	1	1	1	2
G8RQS2	UDP-N-acetylmuramyl tripeptide synthase OS=Mycobacterium rhodesiae (strain NBB3) GN=MycrhN_2075 PE=4 SV=1 - [G8RQS2_MYCRN]	2.39	1	1	1	1
G8RMF4	FHA domain-containing protein OS=Mycobacterium rhodesiae (strain NBB3) GN=MycrhN_1842 PE=4 SV=1 - [G8RMF4_MYCRN]	5.10	1	1	1	1
S7U6Y1	Aldo/keto reductase family protein OS=Geobacillus sp. WSUCF1 GN=I656_02275 PE=4 SV=1 - [S7U6Y1_9BACI]	2.97	50	1	1	1
Q1ASQ4	Amidohydrolase 2 OS=Rubrobacter xylanophilus (strain DSM 9941 / NBRC 16129) GN=Rxyl_2657 PE=4 SV=1 - [Q1ASQ4_RUBXD]	2.40	3	1	1	1
K0WK83	ATPase OS=Pseudomonas fluorescens R124 GN=I1A_002839 PE=4 SV=1 - [K0WK83_PSEFL]	2.69	1	1	1	1
R7YW04	Uncharacterized protein OS=Coniosporium apollinis (strain CBS 100218) GN=W97_05237 PE=4 SV=1 - [R7YW04_CONA1]	2.68	1	1	1	1
D8PA76	Glucose-6-phosphate isomerase OS=Candidatus Nitrospira defluvii GN=NIDE0356 PE=3 SV=1 - [D8PA76_9BACT]	1.76	1	1	1	1
J2UVE8	Arylsulfatase A family protein OS=Pseudomonas sp. GM74 GN=PMI34_02565 PE=4 SV=1 - [J2UVE8_9PSED]	1.93	4	1	1	1
Q1AT20	Fructose-bisphosphate aldolase OS=Rubrobacter xylanophilus (strain DSM 9941 / NBRC 16129) GN=Rxyl_2541 PE=4 SV=1 - [Q1AT20_RUBXD]	4.11	1	1	1	1
Q1ASC0	4Fe-4S ferredoxin, iron-sulfur binding protein OS=Rubrobacter xylanophilus (strain DSM 9941 / NBRC 16129) GN=Rxyl_2795 PE=4 SV=1 - [Q1ASC0_RUBXD]	15.19	1	1	1	1
A0JTM5	Helicase-associated OS=Arthrobacter sp. (strain FB24) GN=Arth_0998 PE=4 SV=1 - [A0JTM5_ARTS2]	5.53	1	1	1	1

Accession	Description	ΣCoverage	Σ# Proteins	Σ# Unique Peptides	Σ# Peptides	Σ# PSMs
E7NB53	Putative glutamate-1-semialdehyde-2,1-aminomutase OS=Actinomyces sp. oral taxon 171 str. F0337 GN=HMPREF9057_02025 PE=3 SV=1 - [E7NB53_9ACTO]	3.07	4	1	1	1
Q1ATJ8	Sulfurtransferase OS=Rubrobacter xylanophilus (strain DSM 9941 / NBRC 16129) GN=Rxyl_0976 PE=4 SV=1 - [Q1ATJ8_RUBXD]	5.46	1	1	1	1
A0A0D6IES8	PQQ-dependent dehydrogenase OS=Mycobacterium smegmatis GN=exaA PE=4 SV=1 - [A0A0D6IES8_MYCSM]	2.85	1	1	1	1
K9DBA8	Uncharacterized protein OS=Massilia timonae CCUG 45783 GN=HMPREF9710_03338 PE=4 SV=1 - [K9DBA8_9BURK]	4.82	1	1	1	1
A1UAH7	Transcriptional regulator, TetR family OS=Mycobacterium sp. (strain KMS) GN=Mkms_0619 PE=4 SV=1 - [A1UAH7_MYCSK]	5.67	1	1	1	1
Q1AUH6	Glyceraldehyde-3-phosphate dehydrogenase OS=Rubrobacter xylanophilus (strain DSM 9941 / NBRC 16129) GN=Rxyl_2005 PE=3 SV=1 - [Q1AUH6_RUBXD]	4.50	1	1	1	1
A0A0B4EI70	Alanine racemase OS=Arthrobacter phenanthrenivorans GN=RM50_12410 PE=3 SV=1 - [A0A0B4EI70_9MICC]	4.00	1	1	1	1
X8DPH6	Uncharacterized protein OS=Mycobacterium abscessus subsp. bolletii 1513 GN=I540_3184 PE=4 SV=1 - [X8DPH6_MYCAB]	1.12	1	1	1	1
Z2DAB9	Uncharacterized protein OS=Pseudomonas aeruginosa VRFPA09 GN=Z046_30850 PE=4 SV=1 - [Z2DAB9_PSEAI]	3.90	1	1	1	1
A0A023X607	Aerobic-type carbon monoxide dehydrogenase middle subunit CoxM/CutM-like protein OS=Rubrobacter radiotolerans GN=RradSPS_2624 PE=4 SV=1 - [A0A023X607_9ACTN]	3.19	1	1	1	1
A0A0C2TM17	Uncharacterized protein OS=Amanita muscaria Koide BX008 GN=M378DRAFT_8869 PE=4 SV=1 - [A0A0C2TM17_AMAMU]	1.45	1	1	1	1
A0A0A1YKY1	Chemotaxis protein CheW OS=Pseudomonas taeanensis MS-3 GN=TMS3_0101175 PE=4 SV=1 - [A0A0A1YKY1_9PSED]	6.17	1	1	1	1
A0A088NID7	Integrase OS=Pseudomonas mosselii SJ10 GN=O165_005490 PE=4 SV=1 - [A0A088NID7_9PSED]	2.25	2	1	1	1
A0A090M9Q3	Superoxide dismutase [Cu-Zn] OS=Fusarium acuminatum CS5907 GN=BN851_0126010 PE=3 SV=1 - [A0A090M9Q3_9HYPO]	8.44	12	1	1	1
W1SN17	Histidine kinase OS=Bacillus vireti LMG 21834 GN=BAVI_11954 PE=4 SV=1 - [W1SN17_9BACI]	2.74	1	1	1	1
A0A024GYA6	Bacterial extracellular solute-binding family protein OS=Arthrobacter siccitolerans GN=smoE PE=4 SV=1 - [A0A024GYA6_9MICC]	3.59	6	1	1	1
A0A0F7E1Y7	Tyrosine-protein kinase OS=Myxococcus fulvus 124B02 GN=MFUL124B02_22795 PE=4 SV=1 - [A0A0F7E1Y7_MYXFU]	0.59	1	1	1	1
A0A024GZA8	Major Facilitator Superfamily protein OS=Arthrobacter siccitolerans GN=nadE PE=4 SV=1 - [A0A024GZA8_9MICC]	2.10	1	1	1	1
W4HSK4	Sulfurtransferase OS=Mycobacterium gastri 'Wayne' GN=MGAST_27295 PE=4 SV=1 - [W4HSK4_MYCGS]	14.00	27	1	1	1

Accession	Description	ΣCoverage	Σ# Proteins	Σ# Unique Peptides	Σ# Peptides	Σ# PSMs
J2TJZ9	Uncharacterized protein OS=Pseudomonas sp. GM74 GN=PMI34_05616 PE=4 SV=1 - [J2TJZ9_9PSED]	4.11	1	1	1	1
F8AXU2	ABC-type transporter, periplasmic subunit family 3 OS=Frankia symbiont subsp. Datisca glomerata GN=FsymDg_4249 PE=4 SV=1 - [F8AXU2_FRADG]	3.15	3	1	1	1
B3Z4Z6	Glycosyltransferase, group 2 family OS=Bacillus cereus NVH0597-99 GN=BC059799_2980 PE=4 SV=1 - [B3Z4Z6_BACCE]	1.39	20	1	1	1
A0A0C1C4E1	Uncharacterized protein OS=Aspergillus ustus GN=HK57_00172 PE=3 SV=1 - [A0A0C1C4E1_9EURO]	0.46	1	1	1	1
A0A090M9M4	WGS project CBMG000000000 data, contig CS5907-c002627 OS=Fusarium acuminatum CS5907 GN=BN851_0118130 PE=4 SV=1 - [A0A090M9M4_9HYPO]	1.87	1	1	1	1
G9NEH9	Uncharacterized protein OS=Hypocrea atroviridis (strain ATCC 20476 / IMI 206040) GN=TRIATDRAFT_288710 PE=4 SV=1 - [G9NEH9_HYPAI]	2.15	1	1	1	1
K0K6R6	Putative UDP-N-acetylglucosamine 2-epimerase OS=Saccharothrix espanaensis (strain ATCC 51144 / DSM 44229 / JCM 9112 / NBRC 15066 / NRRL 15764) GN=BN6_59560 PE=3 SV=1 - [K0K6R6_SACES]	1.89	1	1	1	1
K3UCX8	Uncharacterized protein OS=Fusarium pseudograminearum (strain CS3096) GN=FPSE_10500 PE=4 SV=1 - [K3UCX8_FUSPC]	2.21	1	1	1	1
C5IY18	Putative lipase OS=Penicillium sp. XMZ-9 PE=4 SV=1 - [C5IY18_9EURO]	2.37	1	1	1	1
A0A0F7BPL2	Methyl-accepting chemotaxis protein OS=Myxococcus fulvus 124B02 GN=MFUL124B02_34875 PE=4 SV=1 - [A0A0F7BPL2_MYXFU]	1.30	2	1	1	1
I3SJR5	Uncharacterized protein OS=Lotus japonicus PE=2 SV=1 - [I3SJR5_LOTJA]	4.86	1	1	1	1
A0A0D1NDU4	Um strain SMUC14 PROKKA_contig000008, whole genome shotgun sequence OS=Mycobacterium immunogenum GN=TL11_10680 PE=4 SV=1 - [A0A0D1NDU4_9MYCO]	1.35	2	1	1	1
A0A0F5MWK6	Dihydrodipicolinate reductase OS=Mycobacterium arupense GN=WR43_14640 PE=4 SV=1 - [A0A0F5MWK6_9MYCO]	2.49	1	1	1	1
L8F5X2	HicB family protein OS=Mycobacterium smegmatis MKD8 GN=D806_5252 PE=4 SV=1 - [L8F5X2_MYCSM]	6.98	1	1	1	1
C7ZM70	Predicted protein OS=Nectria haematococca (strain 77-13-4 / ATCC MYA-4622 / FGSC 9596 / MPVI) GN=NECHADRAFT_79819 PE=4 SV=1 - [C7ZM70_NECH7]	0.81	1	1	1	1
A0A0A2A1V6	Toxin secretion ABC transporter ATP-binding protein OS=Prochlorococcus marinus str. MIT 9201 GN=EU95_2023 PE=4 SV=1 - [A0A0A2A1V6_PROMR]	0.91	1	1	1	1
A0A0C3AIH0	Uncharacterized protein (Fragment) OS=Scleroderma citrinum Foug A GN=SCLCIDRAFT_1213206 PE=4 SV=1 - [A0A0C3AIH0_9HOMO]	10.11	1	1	1	1

Accession	Description	ΣCoverage	Σ# Proteins	Σ# Unique Peptides	Σ# Peptides	Σ# PSMs
A0A0F0HT56	Peptidase M48 OS=Saccharothrix sp. ST-888 GN=UK12_08840 PE=3 SV=1 - [A0A0F0HT56_9PSEU]	2.39	1	1	1	1
A0A024M803	Uncharacterized protein OS=Mycobacterium farcinogenes GN=BN975_05568 PE=4 SV=1 - [A0A024M803_9MYCO]	7.41	1	1	1	1
A0A0B7KIN2	Uncharacterized protein OS=Bionectria ochroleuca GN=BN869_000013506_1 PE=4 SV=1 - [A0A0B7KIN2_BIOOC]	1.63	1	1	1	1
A0A024HKW1	Transcriptional regulator OS=Pseudomonas knackmussii (strain DSM 6978 / LMG 23759 / B13) GN=PKB_3810 PE=4 SV=1 - [A0A024HKW1_PSEKB]	5.29	1	1	1	1
C7ZLV7	Putative uncharacterized protein OS=Nectria haematococca (strain 77-13-4 / ATCC MYA-4622 / FGSC 9596 / MPVI) GN=NECHADRAFT_92359 PE=4 SV=1 - [C7ZLV7_NECH7]	0.91	6	1	1	1
L0IRX5	Aerobic-type carbon monoxide dehydrogenase, small subunit CoxS/CutS like protein OS=Mycobacterium smegmatis JS623 GN=Mycsm_00511 PE=4 SV=1 - [L0IRX5_MYCSM]	5.36	1	1	1	1
A0A0C9T0H7	Phosphatase OS=Arthrobacter sp. AK-YN10 GN=M707_23350 PE=4 SV=1 - [A0A0C9T0H7_9MICC]	4.74	1	1	1	1
A0A098F8J9	N-acetyltransferase GCN5 OS=Bacillus simplex GN=BN1180_00655 PE=4 SV=1 - [A0A098F8J9_9BACI]	6.13	1	1	1	1
A0A010TBX4	Acetyl-coenzyme A carboxylase carboxyl transferase subunit alpha OS=Pseudomonas fluorescens HK44 GN=accA PE=3 SV=1 - [A0A010TBX4_PSEFL]	3.17	12	1	1	1
A0A016PEV8	Fusarium graminearum chromosome 1, complete genome OS=Gibberella zeae GN=FG01704.1 PE=4 SV=1 - [A0A016PEV8_GIBZA]	3.00	3	1	1	1
T2GMZ9	Virulence factor Mce family protein OS=Mycobacterium avium subsp. hominissuis TH135 GN=MAH_0799 PE=4 SV=1 - [T2GMZ9_MYCAV]	2.19	1	1	1	1
A0A0F0LCP7	Inner membrane protein YjeH OS=Microbacterium azadirachtae GN=yjeH PE=4 SV=1 - [A0A0F0LCP7_9MICO]	2.69	1	1	1	1
A0A011T9U1	Phosphomannose isomerase OS=Microbacterium sp. MRS-1 GN=AS96_03235 PE=3 SV=1 - [A0A011T9U1_9MICO]	2.51	1	1	1	1
A0A098U5P6	Carboxymethylenebutenolidase OS=Massilia sp. JS1662 GN=IA69_23215 PE=4 SV=1 - [A0A098U5P6_9BURK]	3.72	1	1	1	1
A0A031M416	Glycine/betaine ABC transporter substrate-binding protein OS=Pseudomonas bauzanensis GN=CF98_21385 PE=4 SV=1 - [A0A031M416_9PSED]	3.82	1	1	1	1
A0A011UWW4	Uncharacterized protein OS=Microbacterium sp. MRS-1 GN=AS96_03250 PE=4 SV=1 - [A0A011UWW4_9MICO]	2.95	1	1	1	1
G0RUM1	G-protein coupled receptor protein OS=Hypocrea jecorina (strain QM6a) GN=TRIREDRAFT_123806 PE=4 SV=1 - [G0RUM1_HYPJQ]	3.03	1	1	1	1

Accession	Description	ΣCoverage	Σ# Proteins	Σ# Unique Peptides	Σ# Peptides	Σ# PSMs
C7YUF6	Putative uncharacterized protein OS=Nectria haematococca (strain 77-13-4 / ATCC MYA-4622 / FGSC 9596 / MPVI) GN=NECHADRAFT_96302 PE=3 SV=1 - [C7YUF6_NECH7]	0.70	1	1	1	1
S6VEF8	YD repeat protein (Fragment) OS=Pseudomonas syringae pv. actinidiae ICMP 19096 GN=A245_09246 PE=4 SV=1 - [S6VEF8_PSESF]	1.52	2	1	1	1
W9NWG2	2-oxoisovalerate dehydrogenase E1 component, beta subunit OS=Fusarium oxysporum f. sp. pisi HDV247 GN=FOVG_16672 PE=4 SV=1 - [W9NWG2_FUSOX]	7.45	1	1	1	1
K0K2X4	Uncharacterized protein OS=Saccharothrix espanaensis (strain ATCC 51144 / DSM 44229 / JCM 9112 / NBRC 15066 / NRRL 15764) GN=BN6_53760 PE=4 SV=1 - [K0K2X4_SACES]	3.58	1	1	1	1
I8QQH1	Site-specific recombinase, DNA invertase Pin OS=Frankia sp. QA3 GN=FraQA3DRAFT_4501 PE=4 SV=1 - [I8QQH1_9ACTN]	6.08	2	1	1	1
S2F291	Uncharacterized protein OS=Pseudomonas sp. G5(2012) GN=PG5_33390 PE=4 SV=1 - [S2F291_9PSED]	2.03	1	1	1	1
L7U6L6	Putative T5 A1-like protein OS=Myxococcus stipitatus (strain DSM 14675 / JCM 12634 / Mx s8) GN=MYSTI_01906 PE=4 SV=1 - [L7U6L6_MYXSD]	2.53	1	1	1	1
A0A0A2W5H1	Signal recognition particle protein OS=Beauveria bassiana D1-5 GN=BBAD15_g447 PE=3 SV=1 - [A0A0A2W5H1_BEABA]	3.09	1	1	1	1
A0A0D1LPU8	Um strain SMUC14 PROKKA_contig000031, whole genome shotgun sequence OS=Mycobacterium immunogenum GN=TL11_22860 PE=3 SV=1 - [A0A0D1LPU8_9MYCO]	3.37	1	1	1	1
A1C5B6	Peroxidase, putative OS=Aspergillus clavatus (strain ATCC 1007 / CBS 513.65 / DSM 816 / NCTC 3887 / NRRL 1) GN=ACLA_002950 PE=4 SV=1 - [A1C5B6_ASPCL]	4.76	1	1	1	1
D3D8T4	Rhamnose ABC transporter, periplasmic rhamnose-binding protein OS=Frankia sp. EUN1f GN=FrEUN1fDRAFT_6206 PE=4 SV=1 - [D3D8T4_9ACTN]	3.99	1	1	1	1
A0A0A4GVG8	Uncharacterized protein OS=Pseudomonas mediterranea CFBP 5447 GN=N005_01210 PE=4 SV=1 - [A0A0A4GVG8_9PSED]	6.81	1	1	1	1
D2SEG1	Alanine dehydrogenase OS=Geodermatophilus obscurus (strain ATCC 25078 / DSM 43160 / JCM 3152 / G-20) GN=Gobs_1930 PE=3 SV=1 - [D2SEG1_GEOOG]	3.46	2	1	1	1
I0RI17	Polysaccharide deacetylase OS=Mycobacterium phlei RIVM601174 GN=MPHLEI_21654 PE=4 SV=1 - [I0RI17_MYCPH]	3.97	2	1	1	1
A0A0F4U1F0	Capsular biosynthesis protein OS=Pseudomonas fluorescens GN=VC35_04675 PE=4 SV=1 - [A0A0F4U1F0_PSEFL]	3.68	2	1	1	1
F3P7I7	Conserved domain protein OS=Actinomyces sp. oral taxon 170 str. F0386 GN=HMPREF9056_01003 PE=4 SV=1 - [F3P7I7_9ACTO]	3.87	1	1	1	1

Accession	Description	ΣCoverage	Σ# Proteins	Σ# Unique Peptides	Σ# Peptides	Σ# PSMs
A0A028V6D9	Uncharacterized protein OS=Pseudomonas pseudoalcaligenes AD6 GN=AU05_19950 PE=4 SV=1 - [A0A028V6D9_PSEPS]	10.00	1	1	1	1
W6TPS6	Uncharacterized protein OS=Pedobacter sp. V48 GN=N824_28895 PE=4 SV=1 - [W6TPS6_9SPHI]	1.91	1	1	1	1
A0A031MB14	Uncharacterized protein OS=Pseudomonas bauzanensis GN=CF98_38170 PE=4 SV=1 - [A0A031MB14_9PSED]	2.37	1	1	1	1
S6TXI0	dTDP-4-dehydrorhamnose reductase (Fragment) OS=Pseudomonas syringae pv. actinidiae ICMP 18807 GN=A244_23415 PE=4 SV=1 - [S6TXI0_PSESF]	7.37	3	1	1	1
A0A0D7ECH2	Tn7 transposition protein C OS=Pseudomonas stutzeri GN=LO50_01055 PE=4 SV=1 - [A0A0D7ECH2_PSEST]	3.70	1	1	1	1
A0A0D0K6Z4	Phosphoglucosamine mutase OS=Microbacterium sp. MEJ108Y GN=glmM PE=3 SV=1 - [A0A0D0K6Z4_9MICO]	3.76	1	1	1	1
A0A0B6AJK2	UPF0317 protein BG04_4870 OS=Bacillus megaterium NBRC 15308 = ATCC 14581 GN=BG04_4870 PE=3 SV=1 - [A0A0B6AJK2_BACME]	6.08	1	1	1	1
A0A024M4C4	TetR family transcriptional regulator OS=Mycobacterium farcinogenes GN=BN975_03945 PE=4 SV=1 - [A0A024M4C4_9MYCO]	8.10	1	1	1	1
A0A073K5A1	Uncharacterized protein OS=Bacillus gaemokensis GN=BAGA_27530 PE=4 SV=1 - [A0A073K5A1_9BACI]	2.99	1	1	1	1
W4R200	Sucrose-6-phosphate hydrolase OS=Bacillus akibai (strain ATCC 43226 / DSM 21942 / JCM 9157 / 1139) GN=JCM9157_4885 PE=4 SV=1 - [W4R200_BACA3]	3.95	1	1	1	1
G8RGU7	Uncharacterized protein OS=Mycobacterium rhodesiae (strain NBB3) GN=MycrhN_1445 PE=4 SV=1 - [G8RGU7_MYCRN]	6.67	1	1	1	1
A0A0A8X5E9	Acyl-CoA dehydrogenase OS=Bacillus selenatarsenatis SF-1 GN=SAMD00020551_1488 PE=3 SV=1 - [A0A0A8X5E9_9BACI]	7.98	2	1	1	1
R0H2U3	Uncharacterized protein OS=Capsella rubella GN=CARUB_v10016748mg PE=4 SV=1 - [R0H2U3_9BRAS]	1.11	1	1	1	1
B6H2S2	Pc13g15220 protein OS=Penicillium chrysogenum (strain ATCC 28089 / DSM 1075 / Wisconsin 54-1255) GN=Pc13g15220 PE=4 SV=1 - [B6H2S2_PENCW]	0.92	1	1	1	1
N1V6C2	LytR family transcriptional regulator OS=Arthrobacter crystallopoietes BAB-32 GN=D477_003483 PE=4 SV=1 - [N1V6C2_9MICC]	1.53	1	1	1	1
D5JBG8	RNA polymerase II second largest subunit (Fragment) OS=Coniosporium apollinis GN=RPB2 PE=3 SV=1 - [D5JBG8_9EURO]	2.02	1	1	1	1
A3IFX0	Uncharacterized protein OS=Bacillus sp. B14905 GN=BB14905_12650 PE=4 SV=1 - [A3IFX0_9BACI]	2.27	1	1	1	1
J7XK69	Polysaccharide deacetylase family sporulation protein PdaB OS=Bacillus cereus BAG3X2-1 GN=IE3_05440 PE=4 SV=1 - [J7XK69_BACCE]	1.37	2	1	1	1
K9GZT5	Uncharacterized protein OS=Penicillium digitatum (strain Pd1 / CECT 20795) GN=PDIP_19270 PE=4 SV=1 - [K9GZT5_PEND1]	1.64	1	1	1	1

Accession	Description	ΣCoverage	Σ# Proteins	Σ# Unique Peptides	Σ# Peptides	Σ# PSMs
A3IBM8	Transcriptional regulator, IclR family protein OS=Bacillus sp. B14905 GN=BB14905_00065 PE=4 SV=1 - [A3IBM8_9BACI]	3.11	1	1	1	1
W5J0N0	Uncharacterized protein OS=Pseudomonas sp. (strain M1) GN=PM1_0217830 PE=4 SV=1 - [W5J0N0_PSEUO]	8.57	1	1	1	1
J2S452	Glycine/D-amino acid oxidase, deaminating OS=Pseudomonas sp. GM48 GN=PMI28_03220 PE=4 SV=1 - [J2S452_9PSED]	1.38	2	1	1	1
X7XR24	Uncharacterized protein OS=Mycobacterium kansasii 732 GN=I546_7246 PE=4 SV=1 - [X7XR24_MYCKA]	3.32	1	1	1	1
E1UUP6	tRNA dimethylallyltransferase OS=Bacillus amyloliquefaciens (strain ATCC 23350 / DSM 7 / BCRC 11601 / NBRC 15535 / NRRL B-14393) GN=miaA PE=3 SV=1 - [E1UUP6_BACAS]	2.23	1	1	1	1
V9X2B0	Uncharacterized protein OS=Pseudomonas sp. FGI182 GN=C163_20950 PE=4 SV=1 - [V9X2B0_9PSED]	0.79	1	1	1	1
A0A0D0UXL3	Micromonospora carbonacea strain JXNU-1 contig2, whole genome shotgun sequence OS=Micromonospora carbonacea GN=TK50_16880 PE=4 SV=1 - [A0A0D0UXL3_9ACTN]	10.77	3	1	1	1
A0A062VT07	Uncharacterized protein OS=Microbacterium sp. CH12i GN=DC31_08790 PE=4 SV=1 - [A0A062VT07_9MICO]	5.83	2	1	1	1
A0A0D0WUY0	Micromonospora carbonacea strain JXNU-1 contig2, whole genome shotgun sequence OS=Micromonospora carbonacea GN=TK50_17930 PE=4 SV=1 - [A0A0D0WUY0_9ACTN]	2.72	2	1	1	1
I3DTS6	Uncharacterized protein OS=Bacillus methanolicus MGA3 GN=BMMGA3_13955 PE=4 SV=1 - [I3DTS6_BACMT]	2.33	1	1	1	1
X0L2P5	Uncharacterized protein OS=Fusarium oxysporum f. sp. vasinfectum 25433 GN=FOTG_11773 PE=4 SV=1 - [X0L2P5_FUSOX]	1.14	1	1	1	1
J1HF28	Beta-lactamase OS=Actinomyces georgiae F0490 GN=HMPREF1317_1408 PE=3 SV=1 - [J1HF28_9ACTO]	1.65	1	1	1	1
G7XIF6	Hsp70 family chaperone Lhs1/Orp150 OS=Aspergillus kawachii (strain NBRC 4308) GN=AKAW_04859 PE=3 SV=1 - [G7XIF6_ASPKW]	0.80	1	1	1	1
A0A0D1NIM0	Um strain SMUC14 PROKKA_contig000001, whole genome shotgun sequence OS=Mycobacterium immunogenum GN=TL11_00705 PE=4 SV=1 - [A0A0D1NIM0_9MYCO]	0.81	1	1	1	1
K5BKC8	NADH-ubiquinone/plastoquinone oxidoreductase chain 6 family protein OS=Mycobacterium hassiacum DSM 44199 GN=C731_1419 PE=3 SV=1 - [K5BKC8_9MYCO]	3.56	2	1	1	1
A0A0A2KUU6	tRNA (Uracil-O(2)-)-methyltransferase OS=Penicillium italicum GN=PITC_053930 PE=4 SV=1 - [A0A0A2KUU6_PENIT]	2.09	1	1	1	1
A0A077LEH7	tRNA modification GTPase MnmE OS=Pseudomonas sp. StFLB209 GN=trmE PE=3 SV=1 - [A0A077LEH7_9PSED]	2.19	7	1	1	1
J8MG65	Uncharacterized protein OS=Bacillus cereus VD156 GN=IK7_00694 PE=4 SV=1 - [J8MG65_BACCE]	1.32	1	1	1	1

Accession	Description	ΣCoverage	Σ# Proteins	Σ# Unique Peptides	Σ# Peptides	Σ# PSMs
I1RLA4	Uncharacterized protein OS=Gibberella zeae (strain PH-1 / ATCC MYA-4620 / FGSC 9075 / NRRL 31084) GN=FGSG_04690 PE=4 SV=1 - [I1RLA4_GIBZE]	0.85	2	1	1	1
E5WG96	Uncharacterized protein OS=Bacillus sp. 2_A_57_CT2 GN=HMPREF1013_01471 PE=4 SV=1 - [E5WG96_9BACI]	7.58	1	1	1	1
A0A077LQU3	UbiE/COQ5 family methyltransferase OS=Pseudomonas sp. StFLB209 GN=PSCI_3812 PE=4 SV=1 - [A0A077LQU3_9PSED]	3.94	2	1	1	1
A0A0F5HJJ3	Putative iron binding protein from the HesB_IscA_SufA family OS=Bacillus thermotolerans GN=QY97_03658 PE=4 SV=1 - [A0A0F5HJJ3_9BACI]	8.20	1	1	1	1
A0A024JV63	Caib/baif family protein OS=Mycobacterium triplex GN=BN973_01885 PE=4 SV=1 - [A0A024JV63_9MYCO]	2.74	1	1	1	1
A0A0G0A566	Uncharacterized protein OS=Trichoderma harzianum GN=THAR02_07564 PE=4 SV=1 - [A0A0G0A566_TRIHA]	3.63	2	1	1	1
A0A0F7DZ16	Ribosome-associated GTPase OS=Myxococcus fulvus 124B02 GN=MFUL124B02_14415 PE=4 SV=1 - [A0A0F7DZ16_MYXFU]	3.04	1	1	1	1
R9CKL5	Putative Zn-dependent protease OS=Elizabethkingia meningoseptica ATCC 13253 = NBRC 12535 GN=L100_09379 PE=4 SV=1 - [R9CKL5_ELIME]	5.24	1	1	1	1
N1V2N9	Cobalamin/Fe3+-siderophore ABC transporter ATPase OS=Arthrobacter crystallopoietes BAB-32 GN=D477_019723 PE=3 SV=1 - [N1V2N9_9MICC]	5.88	1	1	1	1
J8DLV5	Uncharacterized protein OS=Bacillus cereus HuA4-10 GN=IGC_03523 PE=4 SV=1 - [J8DLV5_BACCE]	2.67	1	1	1	1
A1CQU3	6-phosphofructo-2-kinase 1 OS=Aspergillus clavatus (strain ATCC 1007 / CBS 513.65 / DSM 816 / NCTC 3887 / NRRL 1) GN=ACLA_027360 PE=4 SV=1 - [A1CQU3_ASPCL]	1.97	1	1	1	1
A0A0A2V3K7	DNA-directed RNA polymerase subunit beta OS=Beauveria bassiana D1-5 GN=BBAD15_g12413 PE=4 SV=1 - [A0A0A2V3K7_BEABA]	2.35	12	1	1	1
A0A024K1D6	Acyl-CoA dehydrogenase domain-containing protein OS=Mycobacterium triplex GN=BN973_03778 PE=4 SV=1 - [A0A024K1D6_9MYCO]	2.06	1	1	1	1
A0A0D8BND0	Uncharacterized protein OS=Frankia sp. CpI1-S GN=FF36_00527 PE=4 SV=1 - [A0A0D8BND0_9ACTN]	2.99	1	1	1	1
F5YWZ3	Uncharacterized protein OS=Mycobacterium sp. (strain JDM601) GN=JDM601_0080 PE=4 SV=1 - [F5YWZ3_MYCSD]	5.23	1	1	1	1
A0A015JCA9	Uncharacterized protein OS=Rhizophagus irregularis DAOM 197198w GN=RirG_141620 PE=4 SV=1 - [A0A015JCA9_9GLOM]	1.83	1	1	1	1
A0A0C1BUI9	ABC-type nitrate/sulfonate/bicarbonate transport system OS=Arthrobacter sp. MWB30 GN=ANMWB30_32660 PE=4 SV=1 - [A0A0C1BUI9_9MICC]	4.97	1	1	1	1

Accession	Description	ΣCoverage	Σ# Proteins	Σ# Unique Peptides	Σ# Peptides	Σ# PSMs
A0A0F4XU85	Chemotaxis protein CheY OS=Pseudomonas kilonensis GN=VP02_02940 PE=4 SV=1 - [A0A0F4XU85_9PSED]	5.26	2	1	1	1
F0EEG1	Uncharacterized protein OS=Pseudomonas sp. (strain TJI-51) GN=G1E_29997 PE=4 SV=1 - [F0EEG1_PSEDT]	5.43	1	1	1	1
A0A0A2IGV6	Peptidase S8/S53, subtilisin/kexin/sedolisin OS=Penicillium expansum GN=PEX1_009640 PE=4 SV=1 - [A0A0A2IGV6_PENEN]	1.16	1	1	1	1
A0A0B5CL03	CRISPR-associated protein Cas3 OS=Pseudomonas balearica DSM 6083 GN=CL52_18065 PE=4 SV=1 - [A0A0B5CL03_9PSED]	0.80	2	1	1	1
W0H4I4	PAS domain S-box/diguanylate cyclase (GGDEF) domain-containing protein OS=Pseudomonas cichorii JBC1 GN=PCH70_05990 PE=4 SV=1 - [W0H4I4_PSECI]	0.66	1	1	1	1
A0A0A1I278	Uncharacterized protein OS=Pseudomonas sp. SHC52 GN=BN844_0717 PE=4 SV=1 - [A0A0A1I278_9PSED]	0.57	1	1	1	1
A0A0D0FXI4	Contig42, whole genome shotgun sequence OS=Pedobacter sp. NL19 GN=TH53_10665 PE=4 SV=1 - [A0A0D0FXI4_9SPHI]	2.11	1	1	1	1
B1J556	Rhomboid family protein OS=Pseudomonas putida (strain W619) GN=PputW619_1574 PE=4 SV=1 - [B1J556_PSEPW]	2.40	3	1	1	1
A0A0A2L1D5	Uncharacterized protein OS=Penicillium italicum GN=PITC_062320 PE=4 SV=1 - [A0A0A2L1D5_PENIT]	1.42	1	1	1	1
A0A0A1YR67	2-hydroxymuconate tautomerase OS=Pseudomonas taeanensis MS-3 GN=TMS3_0107635 PE=3 SV=1 - [A0A0A1YR67_9PSED]	12.12	2	1	1	1
A0A0C5BYJ1	Bacillus coagulans strain HM-08, complete genome OS=Bacillus coagulans GN=SB48_HM08orf00258 PE=3 SV=1 - [A0A0C5BYJ1_BACCO]	3.10	2	1	1	1
A0A090CF27	Uncharacterized protein OS=Podospora anserina (strain S / ATCC MYA-4624 / DSM 980 / FGSC 10383) PE=4 SV=1 - [A0A090CF27_PODAN]	0.38	2	1	1	1
A0A031LZF0	10 kDa chaperonin OS=Pseudomonas bauzanensis GN=groS PE=3 SV=1 - [A0A031LZF0_9PSED]	9.62	1	1	1	1
A0A0D8BCI2	Sugar kinase, ribokinase OS=Frankia sp. CpI1-S GN=FF36_04009 PE=3 SV=1 - [A0A0D8BCI2_9ACTN]	3.31	1	1	1	1
L1M425	YD repeat-containing protein OS=Pseudomonas putida CSV86 GN=CSV86_08066 PE=4 SV=1 - [L1M425_PSEPU]	0.76	1	1	1	1
A0A086WAL2	Uncharacterized protein OS=Massilia consociata GN=JN27_14185 PE=4 SV=1 - [A0A086WAL2_9BURK]	11.76	3	1	1	1
S2VXJ7	UDP-glucose 4-epimerase OS=Actinomyces europaeus ACS-120-V-Col10b GN=HMPREF9238_00595 PE=3 SV=1 - [S2VXJ7_9ACTO]	3.59	1	1	1	1
B2B614	Podospora anserina S mat+ genomic DNA chromosome 2, supercontig 2 OS=Podospora anserina (strain S / ATCC MYA-4624 / DSM 980 / FGSC 10383) GN=PODANS_2_6410 PE=4 SV=1 - [B2B614_PODAN]	4.70	2	1	1	1
A0A0B7JH19	Uncharacterized protein OS=Bionectria ochroleuca GN=BN869_000000108_1 PE=4 SV=1 - [A0A0B7JH19_BIOOC]	0.25	1	1	1	1

Accession	Description	ΣCoverage	Σ# Proteins	Σ# Unique Peptides	Σ# Peptides	Σ# PSMs
A0A0D6SU66	Uncharacterized protein OS=Bacillus mycoides GN=SZ39_2879 PE=4 SV=1 - [A0A0D6SU66_BACMY]	3.67	1	1	1	1
S6I769	Glutathione S-transferase-like protein OS=Pseudomonas sp. CFII68 GN=CFII68_20153 PE=3 SV=1 - [S6I769_9PSED]	5.17	1	1	1	1
A0A0D0JBW8	Contig_12, whole genome shotgun sequence OS=Microbacterium sp. MEJ108Y GN=RU09_07050 PE=4 SV=1 - [A0A0D0JBW8_9MICO]	1.30	1	1	1	1
Q7TUF7	tRNA nucleotidyltransferase/poly(A) polymerase OS=Prochlorococcus marinus subsp. pastoris (strain CCMP1986 / MED4) GN=PMM0303 PE=3 SV=1 - [Q7TUF7_PROMP]	2.65	1	1	1	1
G2X640	Protein kinase domain-containing protein OS=Verticillium dahliae (strain VdLs.17 / ATCC MYA-4575 / FGSC 10137) GN=VDAG_05356 PE=4 SV=1 - [G2X640_VERDV]	4.04	1	1	1	1
A0A0D9AGE0	Transcriptional regulator OS=Pseudomonas stutzeri GN=UF78_17860 PE=4 SV=1 - [A0A0D9AGE0_PSEST]	5.71	2	1	1	1
G8RQY9	RNA polymerase sigma-70 factor, TIGR02957 family OS=Mycobacterium rhodesiae (strain NBB3) GN=MycrhN_3391 PE=4 SV=1 - [G8RQY9_MYCRN]	2.68	1	1	1	1
U2SE16	Multimodular transpeptidase-transglycosylase protein OS=Myxococcus sp. (contaminant ex DSM 436) GN=A176_02306 PE=4 SV=1 - [U2SE16_9DELT]	1.14	1	1	1	1
F2ZLR9	Uncharacterized protein (Fragment) OS=Pseudomonas coronafaciens pv. oryzae str. 1_6 GN=POR16_16569 PE=4 SV=1 - [F2ZLR9_9PSED]	14.71	78	1	1	1
Q5ATH9	Exo-1,4-beta-xylosidase bxlB OS=Emericella nidulans (strain FGSC A4 / ATCC 38163 / CBS 112.46 / NRRL 194 / M139) GN=bxlB PE=1 SV=1 - [BXLB_EMENI]	1.31	1	1	1	1
Q1I5G9	Putative Subtilisin-like serine protease OS=Pseudomonas entomophila (strain L48) GN=PSEEN4433 PE=4 SV=1 - [Q1I5G9_PSEE4]	0.91	1	1	1	1
D3D6E8	Uncharacterized protein OS=Frankia sp. EUN1f GN=FrEUN1fDRAFT_5370 PE=4 SV=1 - [D3D6E8_9ACTN]	4.65	1	1	1	1
S8AST1	Uncharacterized protein OS=Penicillium oxalicum (strain 114-2 / CGMCC 5302) GN=PDE_04102 PE=4 SV=1 - [S8AST1_PENO1]	1.01	1	1	1	1
S6VZT7	Succinate--CoA ligase (ADP-forming) (Fragment) OS=Pseudomonas syringae pv. actinidiae ICMP 19096 GN=sucC PE=4 SV=1 - [S6VZT7_PSESF]	5.14	62	1	1	1
Q8NJ38	Binding protein (Fragment) OS=Rhizophagus irregularis GN=bip PE=3 SV=1 - [Q8NJ38_RHIIR]	7.05	149	1	1	1
C6XUS7	PAS sensor protein OS=Pedobacter heparinus (strain ATCC 13125 / DSM 2366 / NCIB 9290) GN=Phep_1716 PE=4 SV=1 - [C6XUS7_PEDHD]	3.36	1	1	1	1
A6CMZ6	YktC (Fragment) OS=Bacillus sp. SG-1 GN=BSG1_18055 PE=4 SV=1 - [A6CMZ6_9BACI]	4.06	1	1	1	1

Accession	Description	ΣCoverage	Σ# Proteins	Σ# Unique Peptides	Σ# Peptides	Σ# PSMs
Q0CH27	Putative uncharacterized protein OS=Aspergillus terreus (strain NIH 2624 / FGSC A1156) GN=ATEG_07015 PE=4 SV=1 - [Q0CH27_ASPTN]	2.70	9	1	1	1
A0A0C2S9W8	Uncharacterized protein OS=Amanita muscaria Koide BX008 GN=M378DRAFT_14711 PE=4 SV=1 - [A0A0C2S9W8_AMAMU]	2.95	1	1	1	1
A0A0C1DB62	TonB-dependent receptor OS=Pedobacter kyungheensis GN=OC25_22435 PE=3 SV=1 - [A0A0C1DB62_9SPHI]	1.36	1	1	1	1
A3IAL0	Uncharacterized protein OS=Bacillus sp. B14905 GN=BB14905_14785 PE=4 SV=1 - [A3IAL0_9BACI]	3.41	1	1	1	1
B6HS39	Pc22g25180 protein OS=Penicillium chrysogenum (strain ATCC 28089 / DSM 1075 / Wisconsin 54-1255) GN=Pc22g25180 PE=4 SV=1 - [B6HS39_PENCW]	0.50	1	1	1	1
Q5WHI9	Multiple resistance and pH regulation related protein MrpC OS=Bacillus clausii (strain KSM-K16) GN=ABC1631 PE=4 SV=1 - [Q5WHI9_BACSK]	4.32	1	1	1	1
Q5B1N2	Uncharacterized protein OS=Emericella nidulans (strain FGSC A4 / ATCC 38163 / CBS 112.46 / NRRL 194 / M139) GN=AN5548.2 PE=3 SV=1 - [Q5B1N2_EMENI]	0.56	1	1	1	1
A0A010RWA6	Peptidase M14 OS=Pseudomonas fluorescens HK44 GN=HK44_016505 PE=4 SV=1 - [A0A010RWA6_PSEFL]	2.16	7	1	1	1
S6H896	Monofunctional biosynthetic peptidoglycan transglycosylase OS=Pseudomonas sp. CFII68 GN=mtgA PE=3 SV=1 - [S6H896_9PSED]	3.33	1	1	1	1
J2NKM8	Aspartate/tyrosine/aromatic aminotransferase OS=Pseudomonas sp. GM18 GN=PMI21_05112 PE=4 SV=1 - [J2NKM8_9PSED]	2.09	2	1	1	1
A0A0D9NE40	Pyridine nucleotide-disulfide oxidoreductase OS=Aspergillus flavus AF70 GN=P034_00843479 PE=4 SV=1 - [A0A0D9NE40_ASPFL]	1.32	7	1	1	1
A1C7Z1	DNA polymerase OS=Aspergillus clavatus (strain ATCC 1007 / CBS 513.65 / DSM 816 / NCTC 3887 / NRRL 1) GN=ACLA_075510 PE=3 SV=1 - [A1C7Z1_ASPCL]	0.81	1	1	1	1
A0A0C1WV03	Trehalose permease IIC protein OS=Pseudomonas fluorescens GN=NX10_15735 PE=4 SV=1 - [A0A0C1WV03_PSEFL]	1.88	2	1	1	1
A0A0F8WI72	Uncharacterized protein OS=Aspergillus rambellii GN=ARAM_004769 PE=4 SV=1 - [A0A0F8WI72_9EURO]	0.36	1	1	1	1
A0A0A2JAC8	Uncharacterized protein OS=Penicillium expansum GN=PEX1_103920 PE=4 SV=1 - [A0A0A2JAC8_PENEN]	1.04	1	1	1	1
V6T2S8	Uncharacterized protein OS=Bacillus sp. 17376 GN=G3A_07175 PE=4 SV=1 - [V6T2S8_9BACI]	0.78	1	1	1	1
A0A0E4CRD1	Putative nucleic-acid-binding protein containing a Zn-ribbon OS=Mycobacterium lentiflavum GN=BN1232_06187 PE=4 SV=1 - [A0A0E4CRD1_9MYCO]	6.25	1	1	1	1
A0A015JHH0	Uncharacterized protein OS=Rhizophagus irregularis DAOM 197198w GN=RirG_122610 PE=4 SV=1 - [A0A015JHH0_9GLOM]	4.11	1	1	1	1

Accession	Description	ΣCoverage	Σ# Proteins	Σ# Unique Peptides	Σ# Peptides	Σ# PSMs
A0A0C2HVW5	Translation elongation factor Tu OS=Pseudomonas batumici GN=UCMB321_4948 PE=4 SV=1 - [A0A0C2HVW5_9PSED]	20.00	89	1	1	1
A0A0E2WL07	Uncharacterized protein OS=Mycobacterium avium subsp. hominissuis 100 GN=MAV100_25915 PE=4 SV=1 - [A0A0E2WL07_MYCAV]	2.28	1	1	1	1
Q1AY45	Glycosyl transferase, group 1 OS=Rubrobacter xylanophilus (strain DSM 9941 / NBRC 16129) GN=Rxyl_0713 PE=4 SV=1 - [Q1AY45_RUBXD]	2.47	1	1	1	1
A0A0D2XJG1	Uncharacterized protein (Fragment) OS=Fusarium oxysporum f. sp. lycopersici (strain 4287 / CBS 123668 / FGSC 9935 / NRRL 34936) GN=FOXG_04071 PE=4 SV=1 - [A0A0D2XJG1_FUSO4]	2.46	7	1	1	1
S8AWJ2	Uncharacterized protein OS=Penicillium oxalicum (strain 114-2 / CGMCC 5302) GN=PDE_05610 PE=4 SV=1 - [S8AWJ2_PENO1]	0.83	1	1	1	1
A0A0A8JD12	Sulfur carrier protein OS=Bacillus sp. (strain OxB-1) GN=OXB_0161 PE=4 SV=1 - [A0A0A8JD12_BACSX]	3.25	1	1	1	1
A0A0E4H2B3	DNA primase OS=Mycobacterium lentiflavum GN=dnaG PE=3 SV=1 - [A0A0E4H2B3_9MYCO]	1.72	1	1	1	1
J8VCL6	Nitrogen regulatory protein P-II OS=Pseudomonas putida S11 GN=PPS11_13676 PE=4 SV=1 - [J8VCL6_PSEPU]	12.64	22	1	1	1
A0A099S0Q6	Uncharacterized protein OS=Pseudomonas sp. HMP271 GN=DP64_00780 PE=4 SV=1 - [A0A099S0Q6_9PSED]	5.95	1	1	1	1
F4CMF9	O-succinylbenzoate--CoA ligase OS=Pseudonocardia dioxanivorans (strain ATCC 55486 / DSM 44775 / JCM 13855 / CB1190) GN=Psed_1343 PE=4 SV=1 - [F4CMF9_PSEUX]	1.92	1	1	1	1
R8QAL6	Uncharacterized protein OS=Bacillus cereus VD118 GN=IIQ_05386 PE=3 SV=1 - [R8QAL6_BACCE]	1.87	1	1	1	1
A0A086A3H6	Uncharacterized protein OS=Flavobacterium hydatis GN=IW20_20135 PE=4 SV=1 - [A0A086A3H6_FLAHY]	2.34	1	1	1	1
A0A083UNL6	TetR family transcriptional regulator OS=Pseudomonas putida GN=HA62_13960 PE=4 SV=1 - [A0A083UNL6_PSEPU]	5.31	3	1	1	1
G9P620	Uncharacterized protein OS=Hypocrea atroviridis (strain ATCC 20476 / IMI 206040) GN=TRIATDRAFT_286212 PE=4 SV=1 - [G9P620_HYPAI]	1.39	5	1	1	1
A0A0F5KV12	Uncharacterized protein OS=Bacillus sp. TH008 GN=TH62_08995 PE=4 SV=1 - [A0A0F5KV12_9BACI]	13.19	1	1	1	1
Q0RMP9	Uncharacterized protein OS=Frankia alni (strain ACN14a) GN=FRAAL2551 PE=4 SV=1 - [Q0RMP9_FRAAA]	26.53	1	1	1	1
T2HDR7	Uncharacterized protein OS=Pseudomonas putida NBRC 14164 GN=PP4_47550 PE=4 SV=1 - [T2HDR7_PSEPU]	4.55	1	1	1	1
A0A077EF30	Leucine aminopeptidase-related protein OS=Elizabethkingia anophelis NUHP1 GN=BD94_1393 PE=4 SV=1 - [A0A077EF30_9FLAO]	3.36	6	1	1	1
S8AS60	Uncharacterized protein OS=Penicillium oxalicum (strain 114-2 / CGMCC 5302) GN=PDE_03804 PE=4 SV=1 - [S8AS60_PENO1]	4.31	1	1	1	1

Accession	Description	ΣCoverage	Σ# Proteins	Σ# Unique Peptides	Σ# Peptides	Σ# PSMs
F3CDS5	Taurine dioxygenase (Fragment) OS=Pseudomonas savastanoi pv. glycinea str. race 4 GN=Pgy4_31086 PE=4 SV=1 - [F3CDS5_PSESG]	44.74	2	1	1	1
A0A0B8VLI6	ATPase (Fragment) OS=Pseudomonas coronafaciens GN=OA77_17325 PE=4 SV=1 - [A0A0B8VLI6_9PSED]	2.62	2	1	1	1
S6P2P9	Diguanylate cyclase (Fragment) OS=Pseudomonas syringae pv. actinidiae ICMP 18804 GN=A249_36857 PE=4 SV=1 - [S6P2P9_PSESF]	7.34	9	1	1	1
U2XR86	Uncharacterized protein OS=Microbacterium sp. TS-1 GN=MTS1_03375 PE=4 SV=1 - [U2XR86_9MICO]	1.62	1	1	1	1
A0A0A1Z132	DNA methyltransferase OS=Pseudomonas fluorescens LMG 5329 GN=K814_0110225 PE=4 SV=1 - [A0A0A1Z132_PSEFL]	1.68	3	1	1	1
H0JGX1	Putative uncharacterized protein OS=Pseudomonas psychrotolerans L19 GN=PPL19_18040 PE=4 SV=1 - [H0JGX1_9PSED]	2.11	1	1	1	1
D5GPN9	Whole genome shotgun sequence assembly, scaffold_93, strain Mel28 OS=Tuber melanosporum (strain Mel28) GN=GSTUM_00011952001 PE=4 SV=1 - [D5GPN9_TUBMM]	6.71	1	1	1	1
F4CPU4	ABC-type transporter, periplasmic subunit family 3 OS=Pseudonocardia dioxanivorans (strain ATCC 55486 / DSM 44775 / JCM 13855 / CB1190) GN=Psed_3961 PE=3 SV=1 - [F4CPU4_PSEUX]	4.07	1	1	1	1
F9G3E6	Uncharacterized protein OS=Fusarium oxysporum (strain Fo5176) GN=FOXB_13178 PE=3 SV=1 - [F9G3E6_FUSOF]	3.76	7	1	1	1
J3HGL5	Uncharacterized protein OS=Pseudomonas sp. GM67 GN=PMI33_00138 PE=4 SV=1 - [J3HGL5_9PSED]	6.84	1	1	1	1
C4RIX2	Sugar ABC transporter periplasmic sugar-binding protein OS=Micromonospora sp. ATCC 39149 GN=MCAG_05041 PE=4 SV=1 - [C4RIX2_9ACTN]	4.18	2	1	1	1
A1C3C7	Elongation factor Tu (Fragment) OS=Mycobacterium thermoresistibile GN=tuf PE=4 SV=1 - [A1C3C7_MYCTH]	8.37	3	1	1	1
A6CUG3	Serine hydroxymethyltransferase (Fragment) OS=Bacillus sp. SG-1 GN=glyA PE=3 SV=1 - [A6CUG3_9BACI]	2.70	169	1	1	1
Q50177	Probable cyclic synthetase OS=Mycobacterium leprae GN=o1401 PE=4 SV=1 - [Q50177_MYCLR]	0.64	1	1	1	1
D5GKN9	Whole genome shotgun sequence assembly, scaffold_6, strain Mel28 OS=Tuber melanosporum (strain Mel28) GN=GSTUM_00009675001 PE=4 SV=1 - [D5GKN9_TUBMM]	1.32	1	1	1	1
W9D2K2	Uncharacterized protein OS=Frankia sp. CcI6 GN=CcI6DRAFT_01734 PE=4 SV=1 - [W9D2K2_9ACTN]	0.81	6	1	1	1
F3G1S6	NADH:flavin oxidoreductase/NADH oxidase (Fragment) OS=Pseudomonas syringae pv. pisi str. 1704B GN=PSYPI_00750 PE=4 SV=1 - [F3G1S6_PSESJ]	5.09	71	1	1	1
A0A061JS92	Catechol 2,3 dioxygenase OS=Pseudomonas stutzeri KOS6 GN=B597_004160 PE=3 SV=1 - [A0A061JS92_PSEST]	5.83	1	1	1	1

Accession	Description	ΣCoverage	Σ# Proteins	Σ# Unique Peptides	Σ# Peptides	Σ# PSMs
A0A0F0KM13	Uncharacterized protein OS=Microbacterium azadirachtae GN=RL72_02447 PE=4 SV=1 - [A0A0F0KM13_9MICO]	3.88	2	1	1	1
R0GBH8	Uncharacterized protein OS=Capsella rubella GN=CARUB_v10027115mg PE=4 SV=1 - [R0GBH8_9BRAS]	4.63	1	1	1	1
A0A085CIB6	Aconitate hydratase OS=Elizabethkingia meningoseptica GN=acnA PE=3 SV=1 - [A0A085CIB6_ELIME]	0.87	12	1	1	1
A0A028VDZ0	UDP-N-acetyl-D-glucosamine dehydrogenase OS=Pseudomonas pseudoalcaligenes AD6 GN=AU05_09430 PE=3 SV=1 - [A0A028VDZ0_PSEPS]	2.52	1	1	1	1
A0A0D8BBH4	Putative stress response protein, TerZ-and CABP1 OS=Frankia sp. CpI1-S GN=FF36_04125 PE=4 SV=1 - [A0A0D8BBH4_9ACTN]	7.85	8	1	1	1
L8AT32	Uncharacterized protein OS=Bacillus subtilis BEST7613 GN=BEST7613_3008 PE=4 SV=1 - [L8AT32_BACIU]	4.32	1	1	1	1
A0A0F0F9B2	Phosphoribosylglycinamide synthetase OS=Pseudomonas sp. 2(2015) GN=UB48_16455 PE=4 SV=1 - [A0A0F0F9B2_9PSED]	1.40	1	1	1	1
S6TVS3	Cobalamin biosynthesis protein CobG (Fragment) OS=Pseudomonas syringae pv. actinidiae ICMP 19096 GN=A245_43450 PE=4 SV=1 - [S6TVS3_PSESF]	3.23	1	1	1	1
W9NFV5	ATPase OS=Fusarium oxysporum f. sp. pisi HDV247 GN=FOVG_19580 PE=3 SV=1 - [W9NFV5_FUSOX]	1.10	9	1	1	1
A0A074TM82	Lysyl-tRNA synthetase (Fragment) OS=Microbacterium sp. SUBG005 GN=HR12_40240 PE=3 SV=1 - [A0A074TM82_9MICO]	2.88	25	1	1	1
Q2UEU4	Diadenosine polyphosphate hydrolase and related proteins of the histidine triad OS=Aspergillus oryzae (strain ATCC 42149 / RIB 40) GN=AO090026000478 PE=4 SV=1 - [Q2UEU4_ASPOR]	3.98	3	1	1	1
W6QLJ0	Protein SOF1 OS=Penicillium roqueforti FM164 GN=SOF1 PE=4 SV=1 - [W6QLJ0_PENRO]	1.57	1	1	1	1
R0HRK1	Uncharacterized protein OS=Capsella rubella GN=CARUB_v10015686mg PE=3 SV=1 - [R0HRK1_9BRAS]	2.00	1	1	1	1
L1LRP3	HlyD family secretion protein OS=Pseudomonas putida CSV86 GN=CSV86_28838 PE=4 SV=1 - [L1LRP3_PSEPU]	2.68	1	1	1	1
I2FG13	Integrase catalytic protein OS=Pseudomonas sp. K-62 PE=4 SV=1 - [I2FG13_9PSED]	1.99	1	1	1	1
Q5WKV9	Uncharacterized protein OS=Bacillus clausii (strain KSM-K16) GN=ABC0454 PE=4 SV=1 - [Q5WKV9_BACSK]	2.02	1	1	1	1
A0A099CG66	Uncharacterized protein OS=Mycobacterium rufum GN=EU78_07630 PE=4 SV=1 - [A0A099CG66_9MYCO]	2.80	1	1	1	1
E8ND94	Beta-glucosidase-related glycosidase OS=Microbacterium testaceum (strain StLB037) GN=MTES_1988 PE=4 SV=1 - [E8ND94_MICTS]	1.07	1	1	1	1
G8X7L2	Trigger factor OS=Flavobacterium columnare (strain ATCC 49512 / CIP 103533 / TG 44/87) GN=FCOL_04470 PE=4 SV=1 - [G8X7L2_FLACA]	2.04	1	1	1	1

Accession	Description	ΣCoverage	Σ# Proteins	Σ# Unique Peptides	Σ# Peptides	Σ# PSMs
A0A0D2YKP5	Uncharacterized protein OS=Fusarium oxysporum f. sp. lycopersici (strain 4287 / CBS 123668 / FGSC 9935 / NRRL 34936) GN=FOXG_17452 PE=4 SV=1 - [A0A0D2YKP5_FUSO4]	1.64	4	1	1	1
A0A086WCF6	LacI family transcriptional regulator OS=Massilia consociata GN=JN27_07515 PE=4 SV=1 - [A0A086WCF6_9BURK]	2.51	8	1	1	1
A0A0F2R7M9	Histidine kinase OS=Pseudomonas sp. BRH_c35 GN=VR76_01455 PE=4 SV=1 - [A0A0F2R7M9_9PSED]	2.38	5	1	1	1
X5LF85	Mycocerosic acid synthase OS=Mycobacterium vulneris GN=BN979_01455 PE=4 SV=1 - [X5LF85_9MYCO]	0.47	1	1	1	1
Q53D73	Superoxide dismutase OS=Lotus japonicus GN=sodB PE=2 SV=1 - [Q53D73_LOTJA]	2.57	1	1	1	1
N0AYG0	Sulfite reductase [NADPH] flavoprotein alpha-component OS=Bacillus sp. 1NLA3E GN=B1NLA3E_08545 PE=4 SV=1 - [N0AYG0_9BACI]	1.32	1	1	1	1
D8PDK9	DNA repair protein RecN OS=Candidatus Nitrospira defluvii GN=recN PE=3 SV=1 - [D8PDK9_9BACT]	1.59	1	1	1	1
A0A096PFI9	WGS project CBMG000000000 data, contig CS5907-c001243 OS=Fusarium acuminatum CS5907 GN=BN851_0065560 PE=4 SV=1 - [A0A096PFI9_9HYPO]	0.88	1	1	1	1
E4TCS5	Superoxide dismutase OS=Riemerella anatipestifer (strain ATCC 11845 / DSM 15868 / JCM 9532 / NCTC 11014) GN=RA0C_1707 PE=3 SV=1 - [E4TCS5_RIEAD]	4.04	17	1	1	1
A0A077FBH6	Permease OS=Pseudomonas alkylphenolia GN=PSAKL28_14500 PE=4 SV=1 - [A0A077FBH6_9PSED]	3.61	1	1	1	1
X8CIL2	Putative antar domain protein OS=Mycobacterium intracellulare 1956 GN=I550_3816 PE=4 SV=1 - [X8CIL2_MYCIT]	7.36	2	1	1	1
A0A015I5U3	Kre33p OS=Rhizophagus irregularis DAOM 197198w GN=RirG_253250 PE=4 SV=1 - [A0A015I5U3_9GLOM]	0.83	1	1	1	1
A0A0C7CQZ9	Pseudomonas aeruginosa genome assembly PAE221 OS=Pseudomonas aeruginosa GN=PAE221_00162 PE=4 SV=1 - [A0A0C7CQZ9_PSEAI]	1.46	1	1	1	1
H0QH02	Putative uncharacterized protein OS=Arthrobacter globiformis NBRC 12137 GN=ARGLB_008_01260 PE=4 SV=1 - [H0QH02_ARTGO]	10.38	1	1	1	1
D2B009	Glycerol uptake operon antiterminator OS=Streptosporangium roseum (strain ATCC 12428 / DSM 43021 / JCM 3005 / NI 9100) GN=Sros_4359 PE=4 SV=1 - [D2B009_STRRD]	5.42	1	1	1	1
A0A0D1AYE2	SPG23_c13, whole genome shotgun sequence OS=Arthrobacter sp. SPG23 GN=TV39_06060 PE=4 SV=1 - [A0A0D1AYE2_9MICC]	1.04	1	1	1	1
A0A0A2KRG3	Lipopolysaccharide-modifying protein OS=Penicillium italicum GN=PITC_014460 PE=4 SV=1 - [A0A0A2KRG3_PENIT]	3.79	1	1	1	1
A0A0F8V3G8	Malate synthase, glyoxysomal (Fragment) OS=Aspergillus rambellii GN=ARAM_006359 PE=4 SV=1 - [A0A0F8V3G8_9EURO]	8.33	60	1	1	1

Accession	Description	ΣCoverage	Σ# Proteins	Σ# Unique Peptides	Σ# Peptides	Σ# PSMs
D3CYF5	Amidohydrolase 2 OS=Frankia sp. EUN1f GN=FrEUN1fDRAFT_2575 PE=4 SV=1 - [D3CYF5_9ACTN]	2.98	1	1	1	1
U7DIU2	Protein disaggregation chaperone OS=Pseudomonas fluorescens NCIMB 11764 GN=B723_14840 PE=3 SV=1 - [U7DIU2_PSEFL]	1.12	1	1	1	1
G9MQ72	Uncharacterized protein OS=Hypocrea virens (strain Gv29-8 / FGSC 10586) GN=TRIVIDRAFT_92769 PE=4 SV=1 - [G9MQ72_HYPVG]	2.19	3	1	1	1
A0A010SPQ5	Permease OS=Pseudomonas fluorescens HK44 GN=HK44_028470 PE=4 SV=1 - [A0A010SPQ5_PSEFL]	3.68	33	1	1	1
E1VSE9	DNA polymerase III, subunits gamma and tau OS=Arthrobacter arilaitensis (strain DSM 16368 / CIP 108037 / JCM 13566 / Re117) GN=dnaX PE=4 SV=1 - [E1VSE9_ARTAR]	1.05	1	1	1	1
A0A099MVQ1	Uncharacterized protein OS=Pseudomonas plecoglossicida GN=GT93_01735 PE=4 SV=1 - [A0A099MVQ1_9PSED]	10.58	1	1	1	1
V7MM41	TetR family transcriptional regulator OS=Mycobacterium avium subsp. hominissuis 10-5606 GN=N602_26635 PE=4 SV=1 - [V7MM41_MYCAV]	5.70	1	1	1	1
A0A0A4GKY2	Skp OS=Pseudomonas mediterranea CFBP 5447 GN=N005_15635 PE=4 SV=1 - [A0A0A4GKY2_9PSED]	7.78	2	1	1	1
A0A0F4SMF9	Urocanate hydratase (Fragment) OS=Pseudomonas fluorescens GN=VC35_28195 PE=4 SV=1 - [A0A0F4SMF9_PSEFL]	11.61	133	1	1	1
G7XI77	9-cis-epoxycarotenoid dioxygenase OS=Aspergillus kawachii (strain NBRC 4308) GN=AKAW_04750 PE=4 SV=1 - [G7XI77_ASPKW]	2.13	1	1	1	1
A0A024M0C4	S-adenosylmethionine synthase OS=Mycobacterium farcinogenes GN=metK_2 PE=3 SV=1 - [A0A024M0C4_9MYCO]	3.67	2	1	1	1
A0A0F4TJB0	S-(hydroxymethyl)glutathione dehydrogenase OS=Pseudomonas fluorescens GN=VC34_11090 PE=3 SV=1 - [A0A0F4TJB0_PSEFL]	3.76	1	1	1	1
X8AV24	S-adenosylmethionine synthase OS=Mycobacterium xenopi 3993 GN=metK PE=3 SV=1 - [X8AV24_MYCXE]	5.73	52	1	1	1
A0A0D0M7J5	Contig_2, whole genome shotgun sequence OS=Flavobacterium sp. MEB061 GN=RT99_00890 PE=4 SV=1 - [A0A0D0M7J5_9FLAO]	5.20	2	1	1	1
A0A077FJA6	Twin-arginine translocation pathway signal protein OS=Pseudomonas alkylphenolia GN=PSAKL28_50140 PE=4 SV=1 - [A0A077FJA6_9PSED]	7.91	1	1	1	1
J0WGP0	Formylglycine-generating sulfatase enzyme OS=Actinomyces massiliensis F0489 GN=HMPREF1318_1235 PE=4 SV=1 - [J0WGP0_9ACTO]	6.12	15	1	1	1
A0A097F8L6	Chitinase OS=Beauveria bassiana GN=Chi-10 PE=2 SV=1 - [A0A097F8L6_BEABA]	1.50	2	1	1	1
U1U9Y9	Cro/Cl family transcriptional regulator OS=Pseudomonas fluorescens EGD-AQ6 GN=O204_08925 PE=4 SV=1 - [U1U9Y9_PSEFL]	7.14	1	1	1	1
M4X557	dTDP-glucose 4,6-dehydratase OS=Pseudomonas denitrificans ATCC 13867 GN=H681_21600 PE=4 SV=1 - [M4X557_PSEDE]	6.91	1	1	1	1

Accession	Description	ΣCoverage	Σ# Proteins	Σ# Unique Peptides	Σ# Peptides	Σ# PSMs
D5PB09	Type VII secretion protein EccCa OS=Mycobacterium parascrofulaceum ATCC BAA-614 GN=eccCa PE=4 SV=1 - [D5PB09_9MYCO]	1.14	1	1	1	1
G9P7S1	Uncharacterized protein OS=Hypocrea atroviridis (strain ATCC 20476 / IMI 206040) GN=TRIATDRAFT_30317 PE=4 SV=1 - [G9P7S1_HYPAI]	2.56	1	1	1	1
R0FV13	Uncharacterized protein OS=Capsella rubella GN=CARUB_v10022843mg PE=3 SV=1 - [R0FV13_9BRAS]	2.81	1	1	1	1
U9U829	Uncharacterized protein OS=Rhizophagus irregularis (strain DAOM 181602 / DAOM 197198 / MUCL 43194) GN=GLOINDRAFT_22708 PE=4 SV=1 - [U9U829_RHIID]	13.57	2	1	1	1
A0A023X530	Uncharacterized protein possibly involved in utilization of glycolate and propanediol OS=Rubrobacter radiotolerans GN=RradSPS_1891 PE=4 SV=1 - [A0A023X530_9ACTN]	14.81	1	1	1	1
A0A015LZ30	Uncharacterized protein OS=Rhizophagus irregularis DAOM 197198w GN=RirG_184440 PE=4 SV=1 - [A0A015LZ30_9GLOM]	8.26	1	1	1	1
H9T851	Ubiquitin (Fragment) OS=Plantago ovata PE=2 SV=1 - [H9T851_PLAOV]	19.40	155	1	1	1
C3HDD4	PTS system, N-acetylglucosamine-specific IIBC subunit OS=Bacillus thuringiensis serovar pulsiensis BGSC 4CC1 GN=bthur0012_4310 PE=4 SV=1 - [C3HDD4_BACTU]	1.41	1	1	1	1
R0HZ92	Uncharacterized protein OS=Capsella rubella GN=CARUB_v10020374mg PE=4 SV=1 - [R0HZ92_9BRAS]	1.69	2	1	1	1
A0JT14	Uncharacterized protein OS=Arthrobacter sp. (strain FB24) GN=Arth_0786 PE=4 SV=1 - [A0JT14_ARTS2]	3.81	1	1	1	1
E6TXI0	Polyphosphate kinase OS=Bacillus cellulosilyticus (strain ATCC 21833 / DSM 2522 / FERM P-1141 / JCM 9156 / N-4) GN=ppk PE=3 SV=1 - [E6TXI0_BACCJ]	1.10	1	1	1	1
K9FA32	MFS transporter, putative OS=Penicillium digitatum (strain Pd1 / CECT 20795) GN=PDIP_81000 PE=4 SV=1 - [K9FA32_PEND1]	1.89	1	1	1	1
I3SSN9	Uncharacterized protein OS=Lotus japonicus PE=2 SV=1 - [I3SSN9_LOTJA]	6.16	2	1	1	1
R0GQV3	S-(hydroxymethyl)glutathione dehydrogenase OS=Capsella rubella GN=CARUB_v10026595mg PE=3 SV=1 - [R0GQV3_9BRAS]	2.84	8	1	1	1
I3SU87	Uncharacterized protein OS=Lotus japonicus PE=2 SV=1 - [I3SU87_LOTJA]	4.88	2	1	1	1
R0IHA5	Uncharacterized protein OS=Capsella rubella GN=CARUB_v10010178mg PE=4 SV=1 - [R0IHA5_9BRAS]	6.22	4	1	1	1
W8GN23	Enhanced green fluorescent protein OS=Mycobacterium tuberculosis (strain ATCC 25618 / H37Rv) GN=eGFP PE=4 SV=1 - [W8GN23_MYCTU]	4.60	1	1	1	1
Q2PEP8	Putative rubisco subunit binding-protein alpha subunit (Fragment) OS=Trifolium pratense PE=2 SV=1 - [Q2PEP8_TRIPR]	2.60	7	1	1	1

Accession	Description	ΣCoverage	Σ# Proteins	Σ# Unique Peptides	Σ# Peptides	Σ# PSMs
Q3LVN1	TO45-3 (Fragment) OS=Taraxacum officinale GN=To45-3 PE=2 SV=1 - [Q3LVN1_TAROF]	10.81	2	1	1	1
D5G6T3	Whole genome shotgun sequence assembly, scaffold_124, strain Mel28 OS=Tuber melanosporum (strain Mel28) GN=GSTUM_00002258001 PE=4 SV=1 - [D5G6T3_TUBMM]	4.02	1	1	1	1
R0HEU4	Alpha-mannosidase OS=Capsella rubella GN=CARUB_v10002909mg PE=3 SV=1 - [R0HEU4_9BRAS]	1.17	1	1	1	1
G3LKE9	AT1G35720-like protein (Fragment) OS=Capsella grandiflora PE=4 SV=1 - [G3LKE9_9BRAS]	6.86	4	1	1	1
H9BPH6	Pathogenesis-related protein 10 OS=Vicia faba GN=PR10 PE=2 SV=1 - [H9BPH6_VICFA]	11.28	1	1	1	1
I3T7W2	Uricase OS=Lotus japonicus PE=2 SV=1 - [I3T7W2_LOTJA]	4.23	3	1	1	1
H9T7Q2	Profilin OS=Quercus suber PE=2 SV=1 - [H9T7Q2_QUESU]	9.92	10	1	1	1
R0GR76	Proteasome subunit beta type OS=Capsella rubella GN=CARUB_v10009991mg PE=3 SV=1 - [R0GR76_9BRAS]	4.74	1	1	1	1
J7LJ06	NAD-specific glutamate dehydrogenase GdhB OS=Arthrobacter sp. Rue61a GN=gdhB1 PE=4 SV=1 - [J7LJ06_9MICC]	0.80	1	1	1	1
Q0RK08	Putative antibiotic ABC transporter protein, ATP-binding OS=Frankia alni (strain ACN14a) GN=FRAAL3509 PE=3 SV=1 - [Q0RK08_FRAAA]	1.35	4	1	1	1
Q1AVY1	Pyrroline-5-carboxylate reductase OS=Rubrobacter xylanophilus (strain DSM 9941 / NBRC 16129) GN=proC PE=3 SV=1 - [Q1AVY1_RUBXD]	3.82	1	1	1	1
Q9MB61	Asparagine synthetase OS=Astragalus sinicus GN=AsAS1 PE=2 SV=1 - [Q9MB61_ASTSI]	3.08	3	1	1	1
J1SXQ9	Translation elongation factor Tu OS=Myxococcus sp. (contaminant ex DSM 436) GN=A176_01559 PE=4 SV=1 - [J1SXQ9_9DELT]	4.95	7	1	1	1
D8PD20	10 kDa chaperonin OS=Candidatus Nitrospira defluvii GN=groS PE=3 SV=1 - [D8PD20_9BACT]	9.00	1	1	1	1
I3SQF0	Uncharacterized protein OS=Lotus japonicus PE=2 SV=1 - [I3SQF0_LOTJA]	6.94	1	1	1	1
Q43781	Aspartate aminotransferase OS=Lotus japonicus GN=AAT PE=2 SV=1 - [Q43781_LOTJA]	3.35	1	1	1	1
W9MDE0	ADP-ribosylation factor OS=Fusarium oxysporum f. sp. lycopersici MN25 GN=FOWG_07266 PE=3 SV=1 - [W9MDE0_FUSOX]	7.05	41	1	1	1
U4LD80	Similar to Nucleoporin NUP192 acc. no. P47054 OS=Pyronema omphalodes (strain CBS 100304) GN=PCON_08148 PE=4 SV=1 - [U4LD80_PYROM]	0.36	1	1	1	1
A0A0A8JM98	Chaperone protein DnaK OS=Bacillus sp. (strain OxB-1) GN=dnaK PE=2 SV=1 - [A0A0A8JM98_BACSX]	1.16	1	1	1	1
U1Z025	Uncharacterized protein OS=Bacillus sp. EGD-AK10 GN=N880_26500 PE=4 SV=1 - [U1Z025_9BACI]	2.22	1	1	1	1

Accession	Description	ΣCoverage	Σ# Proteins	Σ# Unique Peptides	Σ# Peptides	Σ# PSMs
Q88C94	Uncharacterized protein OS=Pseudomonas putida (strain KT2440) GN=PP_5287 PE=4 SV=1 - [Q88C94_PSEPK]	15.22	2	1	1	1
C2UY19	Uncharacterized protein OS=Bacillus cereus Rock3-28 GN=bcere0019_31350 PE=4 SV=1 - [C2UY19_BACCE]	5.77	4	1	1	1
A0A051TRF4	Uncharacterized protein OS=Mycobacterium tuberculosis TKK-01-0051 GN=K875_05040 PE=4 SV=1 - [A0A051TRF4_MYCTX]	3.01	2	1	1	1
W9NIB8	Uncharacterized protein OS=Fusarium oxysporum f. sp. pisi HDV247 GN=FOVG_16360 PE=4 SV=1 - [W9NIB8_FUSOX]	1.54	1	1	1	1
R8MNB2	Uncharacterized protein OS=Bacillus cereus VD214 GN=IKI_05803 PE=4 SV=1 - [R8MNB2_BACCE]	4.38	9	1	1	1
K0K3B8	Uncharacterized protein OS=Saccharothrix espanaensis (strain ATCC 51144 / DSM 44229 / JCM 9112 / NBRC 15066 / NRRL 15764) GN=BN6_40970 PE=4 SV=1 - [K0K3B8_SACES]	9.86	51	1	1	1
A0A0D7XC92	Esterase (Fragment) OS=Bacillus amyloliquefaciens GN=UZ38_34170 PE=4 SV=1 - [A0A0D7XC92_BACAM]	4.05	1	1	1	1
A0A0F7BPC5	2-amino-4-hydroxy-6-hydroxymethyldihydropteridine pyrophosphokinase OS=Myxococcus fulvus 124B02 GN=MFUL124B02_34485 PE=4 SV=1 - [A0A0F7BPC5_MYXFU]	4.60	1	1	1	1
I3UZG1	Uncharacterized protein OS=Pseudomonas putida ND6 GN=YSA_07612 PE=4 SV=1 - [I3UZG1_PSEPU]	3.60	1	1	1	1
I3SLT0	Uncharacterized protein OS=Lotus japonicus PE=2 SV=1 - [I3SLT0_LOTJA]	4.62	4	1	1	1
Q0CHN8	Predicted protein OS=Aspergillus terreus (strain NIH 2624 / FGSC A1156) GN=ATEG_06796 PE=4 SV=1 - [Q0CHN8_ASPTN]	3.20	1	1	1	1
J3E9X9	Arabinose import ATP-binding protein AraG OS=Pseudomonas sp. GM16 GN=araG PE=3 SV=1 - [J3E9X9_9PSED]	1.95	1	1	1	1
A0A0F0ICQ7	Uncharacterized protein conserved in bacteria DUF2264 OS=Aspergillus parasiticus SU-1 GN=P875_00011240 PE=4 SV=1 - [A0A0F0ICQ7_ASPPA]	1.40	3	1	1	1
G9QLS3	Competence protein ComEA helix-hairpin-helix repeat region OS=Bacillus smithii 7_3_47FAA GN=HMPREF1015_01981 PE=4 SV=1 - [G9QLS3_9BACI]	1.09	1	1	1	1
A0A078M4Q5	Heavy metal translocating P-type ATPase OS=Pseudomonas sp. 12M76_air GN=BN1049_00371 PE=3 SV=1 - [A0A078M4Q5_9PSED]	1.24	3	1	1	1
A0A0D0GRZ7	Contig41, whole genome shotgun sequence OS=Pedobacter sp. NL19 GN=TH53_10400 PE=4 SV=1 - [A0A0D0GRZ7_9SPHI]	1.04	1	1	1	1
A0A0A8JM46	tRNA-specific 2-thiouridylase MnmA OS=Bacillus sp. (strain OxB-1) GN=mnmA PE=3 SV=1 - [A0A0A8JM46_BACSX]	2.14	1	1	1	1
W9IY52	Uncharacterized protein OS=Fusarium oxysporum FOSC 3-a GN=FOYG_02266 PE=4 SV=1 - [W9IY52_FUSOX]	0.97	6	1	1	1

Accession	Description	ΣCoverage	Σ# Proteins	Σ# Unique Peptides	Σ# Peptides	Σ# PSMs
A0A024QWF0	HAD-superfamily hydrolase OS=Mycobacterium neoaurum GN=BN1047_04733 PE=4 SV=1 - [A0A024QWF0_MYCNE]	3.75	1	1	1	1
G9N9Q5	Uncharacterized protein OS=Hypocrea virens (strain Gv29-8 / FGSC 10586) GN=TRIVIDRAFT_40355 PE=4 SV=1 - [G9N9Q5_HYPVG]	1.66	3	1	1	1
A0A0A8JEH9	GTPase HflX OS=Bacillus sp. (strain OxB-1) GN=hflX PE=3 SV=1 - [A0A0A8JEH9_BACSX]	2.84	1	1	1	1
F8AWN3	Uncharacterized protein OS=Frankia symbiont subsp. Datisca glomerata GN=FsymDg_1937 PE=4 SV=1 - [F8AWN3_FRADG]	2.61	1	1	1	1
R0IFZ3	Uncharacterized protein OS=Capsella rubella GN=CARUB_v10020944mg PE=4 SV=1 - [R0IFZ3_9BRAS]	5.16	2	1	1	1
A0A0D6SZ14	Uncharacterized protein OS=Pseudomonas sp. FeS53a GN=SZ55_1295 PE=4 SV=1 - [A0A0D6SZ14_9PSED]	1.28	5	1	1	1
R0EZ20	Uncharacterized protein OS=Capsella rubella GN=CARUB_v10027353mg PE=4 SV=1 - [R0EZ20_9BRAS]	7.46	3	1	1	1
A0A024M601	GTPase Obg OS=Mycobacterium farcinogenes GN=obgE PE=3 SV=1 - [A0A024M601_9MYCO]	2.28	5	1	1	1
I3S8F0	Uncharacterized protein OS=Lotus japonicus PE=2 SV=1 - [I3S8F0_LOTJA]	7.19	3	1	1	1
A0A078MHP2	Bifunctional uroporphyrinogen-III synthetase/response regulator domain protein OS=Pseudomonas sp. 12M76_air GN=BN1049_02269 PE=4 SV=1 - [A0A078MHP2_9PSED]	3.55	1	1	1	1
W9T7U3	Protein Surf1 OS=Pseudomonas sp. BAY1663 GN=surf1 PE=4 SV=1 - [W9T7U3_9PSED]	3.03	1	1	1	1
I3TAM1	Uncharacterized protein OS=Lotus japonicus PE=2 SV=1 - [I3TAM1_LOTJA]	5.88	2	1	1	1
A0A085VDE2	Uncharacterized protein OS=Pseudomonas syringae GN=IV01_19025 PE=4 SV=1 - [A0A085VDE2_PSESX]	13.24	1	1	1	1
A0A0D0VX62	Micromonospora carbonacea strain JXNU-1 contig1, whole genome shotgun sequence OS=Micromonospora carbonacea GN=TK50_07730 PE=4 SV=1 - [A0A0D0VX62_9ACTN]	2.13	1	1	1	1
R0F3J6	Uncharacterized protein OS=Capsella rubella GN=CARUB_v10004406mg PE=3 SV=1 - [R0F3J6_9BRAS]	1.98	2	1	1	1
A0A0D9AZE0	Transcriptional regulator OS=Pseudomonas fluorescens GN=UG46_18570 PE=4 SV=1 - [A0A0D9AZE0_PSEFL]	4.76	1	1	1	1
A0A0C1FSI5	Uncharacterized protein OS=Pedobacter kyungheensis GN=OC25_05015 PE=4 SV=1 - [A0A0C1FSI5_9SPHI]	1.89	1	1	1	1
A0A0F4TTX1	Type IV secretion protein Rhs OS=Pseudomonas fluorescens GN=VC35_10585 PE=4 SV=1 - [A0A0F4TTX1_PSEFL]	0.76	1	1	1	1
S0DX72	Related to ubiquitin-activating enzyme homolog UBA2 OS=Gibberella fujikuroi (strain CBS 195.34 / IMI 58289 / NRRL A-6831) GN=FFUJ_13224 PE=4 SV=1 - [S0DX72_GIBF5]	1.75	1	1	1	1

Accession	Description	ΣCoverage	Σ# Proteins	Σ# Unique Peptides	Σ# Peptides	Σ# PSMs
P52416	Glucose-1-phosphate adenylyltransferase small subunit 1, chloroplastic OS=Vicia faba GN=AGPC PE=2 SV=1 - [GLGS1_VICFA]	2.36	3	1	1	1
I3DWI3	Putative GTPase OS=Bacillus methanolicus PB1 GN=PB1_13639 PE=4 SV=1 - [I3DWI3_BACMT]	1.06	1	1	1	1
R0HN35	40S ribosomal protein SA OS=Capsella rubella GN=CARUB_v10014291mg PE=3 SV=1 - [R0HN35_9BRAS]	4.44	2	1	1	1
S0DWL1	Related to endocytosis ankyrin repeat protein Nuc-2 OS=Gibberella fujikuroi (strain CBS 195.34 / IMI 58289 / NRRL A-6831) GN=FFUJ_13023 PE=4 SV=1 - [S0DWL1_GIBF5]	1.44	1	1	1	1
B2CBB3	Glucose-6-phosphate isomerase (Fragment) OS=Festuca ovina GN=PgiC PE=3 SV=1 - [B2CBB3_9POAL]	2.86	38	1	1	1
I3S077	Uncharacterized protein OS=Lotus japonicus PE=2 SV=1 - [I3S077_LOTJA]	3.69	4	1	1	1
G7X7D9	Mitochondrion protein OS=Aspergillus kawachii (strain NBRC 4308) GN=AKAW_00837 PE=4 SV=1 - [G7X7D9_ASPKW]	0.67	1	1	1	1
Q50442	Mpr OS=Mycobacterium smegmatis GN=mpr PE=4 SV=1 - [Q50442_MYCSM]	6.51	1	1	1	1
U2XBN4	Putative unusual protein kinase OS=Microbacterium sp. TS-1 GN=MTS1_03064 PE=4 SV=1 - [U2XBN4_9MICO]	2.43	1	1	1	1
U1Q602	Uncharacterized protein OS=Actinomyces johnsonii F0510 GN=HMPREF1549_02207 PE=4 SV=1 - [U1Q602_9ACTO]	2.58	1	1	1	1
F3HN82	Threonine/serine transporter OS=Pseudomonas syringae pv. maculicola str. ES4326 GN=PMA4326_18473 PE=4 SV=1 - [F3HN82_PSEYM]	3.76	1	1	1	1
A0A075EAM1	Acetoacetyl-coenzyme A thiolase OS=Astragalus membranaceus PE=2 SV=1 - [A0A075EAM1_ASTME]	6.25	1	1	1	1
D5GL90	Whole genome shotgun sequence assembly, scaffold_66, strain Mel28 OS=Tuber melanosporum (strain Mel28) GN=GSTUM_00010075001 PE=4 SV=1 - [D5GL90_TUBMM]	2.76	1	1	1	1
V7K184	Glutamate decarboxylase OS=Mycobacterium avium subsp. silvaticum ATCC 49884 GN=P863_22855 PE=3 SV=1 - [V7K184_MYCAV]	3.05	4	1	1	1
R0FUG6	Uncharacterized protein OS=Capsella rubella GN=CARUB_v10022595mg PE=4 SV=1 - [R0FUG6_9BRAS]	2.03	1	1	1	1
V7JIP4	Adenylyl cyclase OS=Mycobacterium avium subsp. paratuberculosis 10-4404 GN=O979_18725 PE=4 SV=1 - [V7JIP4_MYCPC]	1.69	4	1	1	1
Q2B9M8	Uncharacterized protein OS=Bacillus sp. NRRL B-14911 GN=B14911_23032 PE=4 SV=1 - [Q2B9M8_9BACI]	3.49	1	1	1	1
A0A0D9NAR3	Dioxygenase OS=Aspergillus flavus AF70 GN=P034_00320759 PE=4 SV=1 - [A0A0D9NAR3_ASPFL]	2.36	4	1	1	1
Q0CDA9	Putative uncharacterized protein OS=Aspergillus terreus (strain NIH 2624 / FGSC A1156) GN=ATEG_08325 PE=4 SV=1 - [Q0CDA9_ASPTN]	2.45	2	1	1	1

Accession	Description	ΣCoverage	Σ# Proteins	Σ# Unique Peptides	Σ# Peptides	Σ# PSMs
A0A0C3E2C5	Uncharacterized protein (Fragment) OS=Scleroderma citrinum Foug A GN=SCLCIDRAFT_119291 PE=4 SV=1 - [A0A0C3E2C5_9HOMO]	7.50	114	1	1	1
J9AIS4	Uncharacterized protein OS=Bacillus cereus BAG6X1-1 GN=IEO_03042 PE=4 SV=1 - [J9AIS4_BACCE]	4.59	1	1	1	1
A0A0A2J8X6	AMP-dependent synthetase/ligase OS=Penicillium expansum GN=PEX1_094100 PE=4 SV=1 - [A0A0A2J8X6_PENEN]	0.08	5	1	1	1
R4UL46	Adenine-specific DNA methyltransferase OS=Mycobacterium abscessus subsp. bolletii 50594 GN=MASS_1p0008 PE=4 SV=1 - [R4UL46_MYCAB]	2.51	1	1	1	1
G7XPT4	Similar to An02g08460 OS=Aspergillus kawachii (strain NBRC 4308) GN=AKAW_07092 PE=4 SV=1 - [G7XPT4_ASPKW]	2.63	12	1	1	1
W4HZN3	Acyl-CoA dehydrogenase OS=Mycobacterium gastri 'Wayne' GN=MGAST_12590 PE=4 SV=1 - [W4HZN3_MYCGS]	1.34	1	1	1	1
A0A0D1P625	Pseudomonas putida strain UASWS0946 Contig161, whole genome shotgun sequence OS=Pseudomonas putida GN=QV12_08885 PE=4 SV=1 - [A0A0D1P625_PSEPU]	2.07	1	1	1	1
A0A0D0MCU6	Strain LMCA8 contig_63, whole genome shotgun sequence OS=Pseudomonas viridiflava GN=RT94_25300 PE=4 SV=1 - [A0A0D0MCU6_PSEVI]	1.49	2	1	1	1
G9MZ07	Uncharacterized protein OS=Hypocrea virens (strain Gv29-8 / FGSC 10586) GN=TRIVIDRAFT_68874 PE=4 SV=1 - [G9MZ07_HYPVG]	1.34	2	1	1	1
T5KKJ6	Uncharacterized protein OS=Microbacterium maritypicum MF109 GN=L687_00815 PE=3 SV=1 - [T5KKJ6_9MICO]	3.20	1	1	1	1
I0S2G8	Uncharacterized protein OS=Mycobacterium phlei RIVM601174 GN=MPHLEI_03418 PE=4 SV=1 - [I0S2G8_MYCPH]	4.35	1	1	1	1
A0A0C1CMS5	Histidine kinase OS=Flavobacterium sp. AED GN=OA85_12325 PE=4 SV=1 - [A0A0C1CMS5_9FLAO]	1.11	1	1	1	1
U3HNX5	Short-chain dehydrogenase OS=Pseudomonas stutzeri MF28 GN=L686_16155 PE=3 SV=1 - [U3HNX5_PSEST]	3.23	2	1	1	1
A0A060LYD6	Uroporphyrinogen III cosynthase OS=Bacillus lehensis G1 GN=BleG1_2620 PE=4 SV=1 - [A0A060LYD6_9BACI]	3.60	1	1	1	1
A0A0E3XTF2	Glycosyl transferase OS=Mycobacterium chelonae GN=GR01_19115 PE=4 SV=1 - [A0A0E3XTF2_MYCCH]	3.40	1	1	1	1
A0A0F0LWE5	Putative aminoacrylate hydrolase RutD OS=Microbacterium ginsengisoli GN=rutD_3 PE=4 SV=1 - [A0A0F0LWE5_9MICO]	2.21	1	1	1	1
A0A0C2XXU8	Uncharacterized protein OS=Bacillus badius GN=SD77_3542 PE=4 SV=1 - [A0A0C2XXU8_BACBA]	21.62	1	1	1	1
R0GVN5	Uncharacterized protein OS=Capsella rubella GN=CARUB_v10028635mg PE=4 SV=1 - [R0GVN5_9BRAS]	1.88	2	1	1	1
X8BEW3	E1-E2 ATPase family protein OS=Mycobacterium xenopi 4042 GN=I553_6532 PE=4 SV=1 - [X8BEW3_MYCXE]	4.90	1	1	1	1

Accession	Description	ΣCoverage	Σ# Proteins	Σ# Unique Peptides	Σ# Peptides	Σ# PSMs
A0A062VS01	Uncharacterized protein OS=Microbacterium sp. CH12i GN=DC31_01680 PE=4 SV=1 - [A0A062VS01_9MICO]	4.83	1	1	1	1
A0A0C1ENM9	Penicillin-binding protein OS=Flavobacterium sp. AED GN=OA85_15660 PE=4 SV=1 - [A0A0C1ENM9_9FLAO]	1.54	1	1	1	1
A0A0A1Z4D5	LuxR family transcriptional regulator OS=Pseudomonas fluorescens LMG 5329 GN=K814_0109775 PE=4 SV=1 - [A0A0A1Z4D5_PSEFL]	5.29	12	1	1	1
C9SAY9	Putative uncharacterized protein OS=Verticillium alfalfae (strain VaMs.102 / ATCC MYA-4576 / FGSC 10136) GN=VDBG_01672 PE=4 SV=1 - [C9SAY9_VERA1]	2.07	1	1	1	1
A1T133	Transcriptional regulator, Fis family OS=Mycobacterium vanbaalenii (strain DSM 7251 / PYR-1) GN=Mvan_0032 PE=4 SV=1 - [A1T133_MYCVP]	2.33	1	1	1	1
A0A016PQ79	Nonribosomal peptide synthetase OS=Gibberella zeae GN=NPS13 PE=4 SV=1 - [A0A016PQ79_GIBZA]	1.02	3	1	1	1
D3D2P4	Uncharacterized protein OS=Frankia sp. EUN1f GN=FrEUN1fDRAFT_4066 PE=4 SV=1 - [D3D2P4_9ACTN]	0.92	1	1	1	1
A0A0F8UYB0	Negative regulator of mitosis OS=Aspergillus rambellii GN=ARAM_006998 PE=4 SV=1 - [A0A0F8UYB0_9EURO]	0.53	2	1	1	1
X8AHA4	Uncharacterized protein OS=Mycobacterium xenopi 4042 GN=I553_4507 PE=4 SV=1 - [X8AHA4_MYCXE]	21.74	10	1	1	1
A0A096AF01	Uncharacterized protein OS=Arthrobacter albus DNF00011 GN=HMPREF2128_10735 PE=4 SV=1 - [A0A096AF01_9MICC]	10.68	1	1	1	1
E5WTC9	Uncharacterized protein OS=Bacillus sp. 2_A_57_CT2 GN=HMPREF1013_05721 PE=4 SV=1 - [E5WTC9_9BACI]	3.24	1	1	1	1
A0A0F0LYB2	Uncharacterized protein OS=Microbacterium azadirachtae GN=RS86_00081 PE=4 SV=1 - [A0A0F0LYB2_9MICO]	1.59	1	1	1	1
Q9F0I1	Putative gamma-glutamyltranspeptidase GgtB (Fragment) OS=Pseudomonas chlororaphis subsp. aureofaciens GN=ggtB PE=4 SV=1 - [Q9F0I1_9PSED]	6.94	39	1	1	1
A0A0E8P0D6	Putative ABC transporter ATP-binding protein OS=Mycobacterium tuberculosis GN=ERS094182_04395 PE=3 SV=1 - [A0A0E8P0D6_MYCTX]	8.17	1	1	1	1
K1KIU5	Uncharacterized protein OS=Bacillus isronensis B3W22 GN=B857_03242 PE=4 SV=1 - [K1KIU5_9BACI]	3.90	1	1	1	1
R4M166	Amidase OS=Mycobacterium tuberculosis str. Haarlem/NITR202 GN=I917_20160 PE=4 SV=1 - [R4M166_MYCTX]	2.20	1	1	1	1
A0A0D9MWF6	Fungal specific transcription factor domain protein OS=Aspergillus flavus AF70 GN=P034_00052337 PE=4 SV=1 - [A0A0D9MWF6_ASPFL]	0.79	4	1	1	1
S6J9V2	Putative lipoprotein OS=Pseudomonas sp. CF161 GN=CF161_10136 PE=4 SV=1 - [S6J9V2_9PSED]	2.79	2	1	1	1

Accession	Description	ΣCoverage	Σ# Proteins	Σ# Unique Peptides	Σ# Peptides	Σ# PSMs
E3J3W9	Acetoacetyl-CoA synthase OS=Frankia sp. (strain EuI1c) GN=FraEuI1c_2568 PE=4 SV=1 - [E3J3W9_FRASU]	1.51	1	1	1	1
S6ISY7	Alcohol dehydrogenase OS=Pseudomonas sp. CFII68 GN=CFII68_11419 PE=4 SV=1 - [S6ISY7_9PSED]	9.38	1	1	1	1
D5GIC0	Whole genome shotgun sequence assembly, scaffold_47, strain Mel28 OS=Tuber melanosporum (strain Mel28) GN=GSTUM_00008418001 PE=4 SV=1 - [D5GIC0_TUBMM]	3.83	1	1	1	1
A4IMU2	DNA replication helicase, Dna2-like protein OS=Geobacillus thermodenitrificans (strain NG80-2) GN=GTNG_1276 PE=4 SV=1 - [A4IMU2_GEOTN]	0.64	1	1	1	1
A8F9K2	S8A subfamily serine protease OS=Bacillus pumilus (strain SAFR-032) GN=wprA PE=4 SV=1 - [A8F9K2_BACP2]	1.35	1	1	1	1
A0A078MLM6	Ribosomal RNA small subunit methyltransferase E OS=Arthrobacter sp. 11W110_air GN=rsmE PE=3 SV=1 - [A0A078MLM6_9MICC]	3.16	2	1	1	1
K0V2B2	Putative transmembrane protein OS=Mycobacterium vaccae ATCC 25954 GN=MVAC_26210 PE=4 SV=1 - [K0V2B2_MYCVA]	2.16	1	1	1	1
G9MZ13	Uncharacterized protein OS=Hypocrea virens (strain Gv29-8 / FGSC 10586) GN=TRIVIDRAFT_90070 PE=3 SV=1 - [G9MZ13_HYPVG]	1.32	4	1	1	1
A0A086T652	Translin-associated protein X-like protein OS=Acremonium chrysogenum ATCC 11550 GN=ACRE_043390 PE=4 SV=1 - [A0A086T652_ACRCH]	2.71	1	1	1	1
W6QGP6	F-box domain, cyclin-like OS=Penicillium roqueforti FM164 GN=PROQFM164_S04g000491 PE=4 SV=1 - [W6QGP6_PENRO]	1.47	1	1	1	1
A0A078M003	Oxidoreductase, zinc-binding OS=Pseudomonas sp. 20_BN GN=BN1079_03306 PE=3 SV=1 - [A0A078M003_9PSED]	2.56	1	1	1	1
W7VCE4	Daunorubicin biosynthesis sensory transduction protein DnrJ OS=Micromonospora sp. M42 GN=MCBG_01984 PE=3 SV=1 - [W7VCE4_9ACTN]	2.17	1	1	1	1
E3IYQ7	Uncharacterized protein OS=Frankia sp. (strain EuI1c) GN=FraEuI1c_0972 PE=4 SV=1 - [E3IYQ7_FRASU]	2.46	1	1	1	1
F8CAY8	10 kDa chaperonin OS=Myxococcus fulvus (strain ATCC BAA-855 / HW-1) GN=groS PE=3 SV=1 - [F8CAY8_MYXFH]	9.38	3	1	1	1
Q9RBD9	Protein RecA (Fragment) OS=Arthrobacter sp. 'SMCC G980' GN=recA PE=3 SV=1 - [Q9RBD9_9MICC]	4.50	2	1	1	1
S0EDC7	Uncharacterized protein OS=Gibberella fujikuroi (strain CBS 195.34 / IMI 58289 / NRRL A-6831) GN=FFUJ_12778 PE=4 SV=1 - [S0EDC7_GIBF5]	6.67	1	1	1	1
C3AQN5	Penicillin-binding protein transpeptidase OS=Bacillus mycoides Rock1-4 GN=bmyco0002_35170 PE=4 SV=1 - [C3AQN5_BACMY]	1.33	5	1	1	1

Accession	Description	ΣCoverage	Σ# Proteins	Σ# Unique Peptides	Σ# Peptides	Σ# PSMs
F4DPY6	Mannose-1-phosphate guanylyltransferase OS=Pseudomonas mendocina (strain NK-01) GN=MDS_4598 PE=3 SV=1 - [F4DPY6_PSEMN]	1.74	2	1	1	1
A0A0F0KHB3	Bacterial regulatory protein, luxR family OS=Microbacterium foliorum GN=RN50_02122 PE=4 SV=1 - [A0A0F0KHB3_9MICO]	1.95	1	1	1	1
A0A0F8TZF3	Uncharacterized protein OS=Aspergillus rambellii GN=ARAM_006485 PE=4 SV=1 - [A0A0F8TZF3_9EURO]	1.75	1	1	1	1
M4WYC2	FAD dependent oxidoreductase OS=Pseudomonas denitrificans ATCC 13867 GN=H681_16500 PE=4 SV=1 - [M4WYC2_PSEDE]	1.95	1	1	1	1
V7KVR6	Uncharacterized protein OS=Mycobacterium avium subsp. silvaticum ATCC 49884 GN=P863_04285 PE=4 SV=1 - [V7KVR6_MYCAV]	14.29	1	1	1	1
N1RTR0	Mitochondrial dicarboxylate transporter OS=Fusarium oxysporum f. sp. cubense (strain race 4) GN=FOC4_g10005679 PE=3 SV=1 - [N1RTR0_FUSC4]	6.13	1	1	1	1
Q7TUY3	Possible Thermophilic metalloprotease (M29) OS=Prochlorococcus marinus (strain MIT 9313) GN=PMT_0948 PE=4 SV=1 - [Q7TUY3_PROMM]	8.59	1	1	1	1
J0XS87	Protein RecA OS=Actinomyces georgiae F0490 GN=recA PE=3 SV=1 - [J0XS87_9ACTO]	3.05	8	1	1	1
A0A0D9M4U1	Uncharacterized protein OS=Penicillium solitum GN=U727_00432520161 PE=4 SV=1 - [A0A0D9M4U1_9EURO]	2.50	1	1	1	1
M7MQK9	AMP-dependent synthetase and ligase OS=Arthrobacter gangotriensis Lz1y GN=ADIAG_02112 PE=4 SV=1 - [M7MQK9_9MICC]	2.26	1	1	1	1
B2B7L5	Podospora anserina S mat+ genomic DNA chromosome 2, supercontig 2 OS=Podospora anserina (strain S / ATCC MYA-4624 / DSM 980 / FGSC 10383) GN=PODANS_2_11520 PE=4 SV=1 - [B2B7L5_PODAN]	2.68	1	1	1	1
A0A0D6GAW8	GntR family transcriptional regulator OS=Mycobacterium smegmatis GN=ydfH_3 PE=4 SV=1 - [A0A0D6GAW8_MYCSM]	5.66	4	1	1	1
F9G053	Uncharacterized protein OS=Fusarium oxysporum (strain Fo5176) GN=FOXB_12035 PE=4 SV=1 - [F9G053_FUSOF]	1.94	1	1	1	1
I9KG21	Uncharacterized protein OS=Frankia sp. QA3 GN=FraQA3DRAFT_2700 PE=4 SV=1 - [I9KG21_9ACTN]	2.11	1	1	1	1
Q7V650	Putative exopolyphosphatase OS=Prochlorococcus marinus (strain MIT 9313) GN=ppx PE=4 SV=1 - [Q7V650_PROMM]	2.30	1	1	1	1
A0A0F7TGE8	Uncharacterized protein OS=Penicillium brasilianum GN=PMG11_01850 PE=4 SV=1 - [A0A0F7TGE8_9EURO]	1.59	1	1	1	1
A0A0F0I304	PXA domain protein OS=Aspergillus parasiticus SU-1 GN=P875_00042199 PE=4 SV=1 - [A0A0F0I304_ASPPA]	1.20	1	1	1	1
A0A0C3E1D2	Uncharacterized protein OS=Scleroderma citrinum Foug A GN=SCLCIDRAFT_1215206 PE=3 SV=1 - [A0A0C3E1D2_9HOMO]	2.95	1	1	1	1

Accession	Description	ΣCoverage	Σ# Proteins	Σ# Unique Peptides	Σ# Peptides	Σ# PSMs
U9TV30	Uncharacterized protein OS=Rhizophagus irregularis (strain DAOM 181602 / DAOM 197198 / MUCL 43194) GN=GLOINDRAFT_27667 PE=4 SV=1 - [U9TV30_RHIID]	7.17	1	1	1	1
H7EXR9	Substrate-binding protein OS=Pseudomonas stutzeri ATCC 14405 = CCUG 16156 GN=PstZobell_14016 PE=4 SV=1 - [H7EXR9_PSEST]	2.63	1	1	1	1

	A10				B10				C10				D10			
Accession	Score A2	Coverage A2	# Peptides A2	# PSM A2	Score B2	Coverage B2	# Peptides B2	# PSM B2	Score C2	Coverage C2	# Peptides C2	# PSM C2	Score D2	Coverage D2	# Peptides D2	# PSM D2
A0A015J624	51.62	10.40	3	4	45.29	12.27	3	11	102.33	12.27	3	9	95.75	14.93	4	9
C4MMH6	51.62	6.69	1	2	37.59	20.45	3	10	78.57	12.64	2	7	65.80	12.64	2	5
Q6UDA0	51.62	7.43	2	3	37.59	7.96	2	6	78.57	7.96	2	4	84.87	10.61	3	7
A0A031M2U0					83.02	8.13	5	6	34.42	6.84	4	4	76.17	6.84	4	5
U2ZLC7					64.17	6.96	4	5	59.19	3.48	2	2	0.00	4.03	2	2
A0A031M2A5					83.02	6.27	4	5	34.42	4.98	3	3	76.17	6.83	4	5
A0A031M511					83.02	6.23	4	5	34.42	8.06	4	4	76.17	6.78	4	5
D5G725																
Q1AXU6	50.79	1.85	1	1	0.00	5.56	3	5	44.94	4.26	2	2	0.00	7.04	3	4
E7BCR3					50.09	14.10	1	2	35.24	14.10	1	1	35.96	14.10	1	3
D8PD19					77.78	3.85	3	4	34.42	4.40	3	3	71.18	2.56	2	2
D9J011	0.00	2.65	1	1	0.00	3.18	1	1	0.00	3.18	1	1	64.97	5.84	2	3
A0A097NU89					0.00	2.48	1	1								
D2S4D2	184.66	6.42	3	5												
F8CQR1					64.17	2.93	2	3								
N1V613					0.00	3.18	2	2	59.19	1.87	1	1	0.00	3.18	2	2
X5C9W2																
D2B144	43.11	7.23	3	3												

	A10				B10				C10				D10			
Accession	Score A2	Coverage A2	# Peptides A2	# PSM A2	Score B2	Coverage B2	# Peptides B2	# PSM B2	Score C2	Coverage C2	# Peptides C2	# PSM C2	Score D2	Coverage D2	# Peptides D2	# PSM D2
D5G530					0.00	4.26	2	2	0.00	1.42	1	1				
A0A0D9N6M8					0.00	4.31	2	2	0.00	3.41	2	2				
P42653																
U3MV00																
A0A023X6K3	107.40	11.19	3	4												
Q1D2S1					64.74	2.91	2	2	52.96	1.64	1	1				
A0A0A2C600					52.63	3.26	2	2	47.26	1.92	1	1	0.00	1.92	1	1
C2ZER9									0.00	7.63	1	1				
F4CRF7	58.81	5.04	2	2												
C0VYK7	40.64	6.17	3	4												
A0A023X255	142.91	8.09	3	4												
F8CAY9					64.74	2.91	2	2	52.96	3.83	2	2				
A0A0B4CW17					0.00	2.46	1	2	0.00	2.46	1	1				
S6WAM9													45.99	7.06	1	3
A0A0D0V2Q0	70.42	4.61	2	2					52.16	2.40	1	1	0.00	2.40	1	1
A0A024QIB3	71.88	6.68	2	3												
A0A031LYZ6	148.07	3.93	1	3										0.00		
R0IPS1					0.00	2.46	1	1								

	A10				B10				C10				D10			
Accession	Score A2	Coverage A2	# Peptides A2	# PSM A2	Score B2	Coverage B2	# Peptides B2	# PSM B2	Score C2	Coverage C2	# Peptides C2	# PSM C2	Score D2	Coverage D2	# Peptides D2	# PSM D2
A0A086WFH7					0.00	13.79	1	2	55.35	13.79	1	1	0.00	13.79	1	2
H0QKE9									48.48	4.71	1	1	69.64	4.71	1	2
D2X5N3																
P31926																
Q2PEX3																
K3VXF8	0.00	0.38	1	1					0.00	0.38	1	1	0.00	0.38	1	1
D2SGA0	64.28	8.20	2	2												
A0A023X1G9	122.73	7.56	1	3												
A0A0F0HPK1	0.00	2.16	2	3												
G3FEZ4					49.15	5.83	1	1	74.02	5.83	1	2				
I3S5Q3																
Q684J8																
D5GF49																
A1UHF2	49.99	6.20	2	2												
N1V4B6	83.21	6.19	2	2												
Q07838	0.00	6.40	2	2												
R0HQP2	54.09	2.45	1	1												
E5WLJ3	37.81	7.92	3	3												

	A10				B10				C10				D10			
Accession	Score A2	Coverage A2	# Peptides A2	# PSM A2	Score B2	Coverage B2	# Peptides B2	# PSM B2	Score C2	Coverage C2	# Peptides C2	# PSM C2	Score D2	Coverage D2	# Peptides D2	# PSM D2
A0A066WMI9	66.04	1.98	1	1					53.46	1.98	1	1				
X8BKS3	67.17	18.06	1	1					47.70	18.06	1	1	69.75	18.06	1	1
A0A024M4M9	0.00	7.69	1	1												
A0A023X435					0.00	0.62	1	1	53.15	0.70	1	1				
J2MVZ3													0.00	11.63	4	4
I0L6Q5													0.00	0.61	1	1
B1PZ38													0.00	11.66	4	4
D5GIA8																
A0A024HBV6																
U5IBT8																
R0GZH8																
R0HG12																
I3SNN1																
I3S3D3																
D5GLX2																
I3T0F4																
I3T8P4																

	A10				B10				C10				D10			
Accession	Score A2	Coverage A2	# Peptides A2	# PSM A2	Score B2	Coverage B2	# Peptides B2	# PSM B2	Score C2	Coverage C2	# Peptides C2	# PSM C2	Score D2	Coverage D2	# Peptides D2	# PSM D2
C8VFF7	0.00	1.33	1	1									57.13	1.33	1	1
M2V5V0	47.94	2.37	1	1									44.89	2.37	1	2
D2S8V2	0.00	1.43	1	2												
Q8GI57	56.10	5.83	1	2												
Q0CKI2	60.00	2.23	1	2												
B5AEM9	59.31	2.07	1	2												
U2AB71	55.70	2.26	1	2												
L8TPB7	45.57	3.17	1	2												
A0A099CPS9	47.18	2.72	1	1		0.00										
A0A031MCA3	56.12	3.49	1	1												
X8FJG5	62.59	10.62	1	1												
A0A0D0WZL0	45.17	4.67	1	3												
A0A074TMY9	50.37	15.25	2	2												
W7VQP0	37.25	2.60	2	2												
F3EXH9	38.11	4.44	2	2												
A0A0F7P5R1	0.00	5.56	1	1												

	A10				B10				C10				D10			
Accession	Score A2	Coverage A2	# Peptides A2	# PSM A2	Score B2	Coverage B2	# Peptides B2	# PSM B2	Score C2	Coverage C2	# Peptides C2	# PSM C2	Score D2	Coverage D2	# Peptides D2	# PSM D2
I3T598					0.00	4.79	1	2								
A0A0E4CR92					0.00	8.86	1	1		0.00			0.00	8.86	1	1
A0A0A1Z7Q1					0.00	16.33	1	1	0.00	16.33	1	1				
X7Z574					0.00	5.56	1	1					0.00	5.56	1	1
W9N0A1					0.00	1.99	1	3								
U2SUS3									46.73	1.73	1	1				
A0A0C2I3T2									0.00	2.67	1	3				
L8AJT4																
D5GIK5																
D5GLX7																
I3SZE9																
R0G5L8																
R0GR46																
Q58ZF1																
R0F5B5																
R0INE5																
A0A059XMU8																
D2BBD1																

	A10				B10				C10				D10			
Accession	Score A2	Coverage A2	# Peptides A2	# PSM A2	Score B2	Coverage B2	# Peptides B2	# PSM B2	Score C2	Coverage C2	# Peptides C2	# PSM C2	Score D2	Coverage D2	# Peptides D2	# PSM D2
Q1AUM3	43.52	2.27	1	1												
U3HB76	0.00	3.14	1	1												
A0A081I584	46.89	7.95	1	1												
J3AB25	41.13	2.70	1	2												
L0IVW3	39.78	4.52	2	2												
A0A0D7FCN8	69.63	4.39	1	2												
Q1AZT7	0.00	2.83	2	2												
A4VIS3	35.32	13.20	2	2												
W4PWG4	0.00	1.20	1	1												
A0A064CC06	53.97	4.18	2	2												
A0A031M6T0	49.77	6.73	2	2												
X8C966	41.00	7.82	1	1												
A0A077LJN0	86.53	3.76	1	1												
A0A023X481	33.22	5.59	2	2												
A0A0F0H9G5		0.00								0.00				0.00		
X8FHU5	31.16	4.44	1	1												
A0A024EJ81	32.39	13.19	1	1												

	A10				B10				C10				D10			
Accession	Score A2	Coverage A2	# Peptides A2	# PSM A2	Score B2	Coverage B2	# Peptides B2	# PSM B2	Score C2	Coverage C2	# Peptides C2	# PSM C2	Score D2	Coverage D2	# Peptides D2	# PSM D2
A0A0F7BTD3	58.12	5.02	1	1												
D2B9Y7	0.00	2.12	1	1												
W4Q7M1	40.03	2.22	1	1												
A0A023WYP6	35.64	2.66	1	2												
C7YL24	0.00	3.14	1	2												
G4I7D7					0.00	1.39	1	2								
C7Z1H4					50.01	4.00	1	2								
B2B374						0.00							64.55	0.51	1	1
S6IZP4					0.00	6.06	1	1					0.00	5.30	1	1
A0A0F5MQC0					0.00	3.64	1	1								
F9F4B5					0.00	4.94	1	2								
A0A085FS16					0.00	1.28	1	2								
A0A0B4KEB7									0.00	6.50	1	1	0.00	8.94	1	1
A0A017SQ96									39.63	25.29	2	2				
A0A0D0EX19									0.00	0.66	1	1	0.00	0.66	1	1
A0A0F0HX18									0.00	1.31	1	1				
A0A085FJB3									43.32	10.71	1	1	43.51	10.71	1	1

	A10				B10				C10				D10			
Accession	Score A2	Coverage A2	# Peptides A2	# PSM A2	Score B2	Coverage B2	# Peptides B2	# PSM B2	Score C2	Coverage C2	# Peptides C2	# PSM C2	Score D2	Coverage D2	# Peptides D2	# PSM D2
G9G3Y9													0.00	3.36	1	2
A0A0A1CW01													0.00	4.11	1	2
K0XAB6													0.00	2.16	1	2
U1S6V9													0.00	1.78	1	2
I0LBY2																
N1VC98																
D5GAH8																
D5GHM3																
W7ZA32																
G2Z5G4																
J3C4Y2																
I3SH51																
R0GU86																
R0GKE5																
R0HKB9																
R0FNU6																
Q9XHC7																
R0FV42																

	A10				B10				C10				D10			
Accession	Score A2	Coverage A2	# Peptides A2	# PSM A2	Score B2	Coverage B2	# Peptides B2	# PSM B2	Score C2	Coverage C2	# Peptides C2	# PSM C2	Score D2	Coverage D2	# Peptides D2	# PSM D2
R0GRF1																
D8PIF0																
R0FX04																
E3Q1S2																
R0FHR7																
I3T4F6																
I3T5V4																
Q2PEW2																
I3T2W0																
Q3LVQ8																
M5R2U9																
Q6Q4Z3																
I3S0W6																
R0HNM5																
W7VC77																
R0HKA2																
A0A075EAM3																
R0I261																
D6PPS9																
A0A059WQB8																

	A10				B10				C10				D10			
Accession	Score A2	Coverage A2	# Peptides A2	# PSM A2	Score B2	Coverage B2	# Peptides B2	# PSM B2	Score C2	Coverage C2	# Peptides C2	# PSM C2	Score D2	Coverage D2	# Peptides D2	# PSM D2
C2NS66																
A0A0A0E634																
I3S9M6																
U4L650																
R0G9G5																
I3S358																
I3S2A7																
A0A0B7JX17																
Q83XX0																
Q0REU0																
S7ZEP9																
G9QDX3																
G8RQS2		0.00														
G8RMF4	44.56	5.10	1	1												
S7U6Y1	54.89	2.97	1	1												
Q1ASQ4	0.00	2.40	1	1												
K0WK83	0.00	2.69	1	1												
R7YW04	0.00	2.68	1	1												
D8PA76	0.00	1.76	1	1												

	A10				B10				C10				D10			
Accession	Score A2	Coverage A2	# Peptides A2	# PSM A2	Score B2	Coverage B2	# Peptides B2	# PSM B2	Score C2	Coverage C2	# Peptides C2	# PSM C2	Score D2	Coverage D2	# Peptides D2	# PSM D2
J2UVE8	40.79	1.93	1	1												
Q1AT20	66.45	4.11	1	1												
Q1ASC0	55.52	15.19	1	1												
A0JTM5	0.00	5.53	1	1												
E7NB53	74.58	3.07	1	1												
Q1ATJ8	34.34	5.46	1	1												
A0A0D6IES8	28.52	2.85	1	1												
K9DBA8	26.09	4.82	1	1												
A1UAH7	0.00	5.67	1	1												
Q1AUH6	0.00	4.50	1	1												
A0A0B4EI70	56.11	4.00	1	1												
X8DPH6	50.52	1.12	1	1												
Z2DAB9	0.00	3.90	1	1												
A0A023X607	53.25	3.19	1	1												
A0A0C2TM17	0.00	1.45	1	1												
A0A0A1YKY1	42.83	6.17	1	1												
A0A088NID7	0.00	2.25	1	1												

	A10				B10				C10				D10			
Accession	Score A2	Coverage A2	# Peptides A2	# PSM A2	Score B2	Coverage B2	# Peptides B2	# PSM B2	Score C2	Coverage C2	# Peptides C2	# PSM C2	Score D2	Coverage D2	# Peptides D2	# PSM D2
A0A090M9Q3	75.27	8.44	1	1												
W1SN17	0.00	2.74	1	1												
A0A024GYA6	42.92	3.59	1	1												
A0A0F7E1Y7		0.00			0.00	0.59	1	1								
A0A024GZA8		0.00														
W4HSK4	25.28	14.00	1	1												
J2TJZ9	0.00	4.11	1	1												
F8AXU2	0.00	3.15	1	1												
B3Z4Z6	0.00	1.39	1	1												
A0A0C1C4E1	0.00	0.46	1	1												
A0A090M9M4	0.00	1.87	1	1												
G9NEH9	0.00	2.15	1	1												
K0K6R6	0.00	1.89	1	1												
K3UCX8	0.00	2.21	1	1												
C5IY18	0.00	2.37	1	1												
A0A0F7BPL2	0.00	1.30	1	1												
I3SJR5	0.00	4.86	1	1												

	A10				B10				C10				D10			
Accession	Score A2	Coverage A2	# Peptides A2	# PSM A2	Score B2	Coverage B2	# Peptides B2	# PSM B2	Score C2	Coverage C2	# Peptides C2	# PSM C2	Score D2	Coverage D2	# Peptides D2	# PSM D2
A0A0D1NDU4		0.00														
A0A0F5MWK6	0.00	2.49	1	1												
L8F5X2	0.00	6.98	1	1												
C7ZM70	0.00	0.81	1	1												
A0A0A2A1V6	0.00	0.91	1	1												
A0A0C3AIH0	0.00	10.11	1	1												
A0A0F0HT56	0.00	2.39	1	1												
A0A024M803	0.00	7.41	1	1												
A0A0B7KIN2	0.00	1.63	1	1												
A0A024HKW1	0.00	5.29	1	1												
C7ZLV7	0.00	0.91	1	1												
L0IRX5	0.00	5.36	1	1												
A0A0C9T0H7	30.46	4.74	1	1												
A0A098F8J9	0.00	6.13	1	1												
A0A010TBX4	0.00	3.17	1	1												
A0A016PEV8	0.00	3.00	1	1												

	A10				B10				C10				D10			
Accession	Score A2	Coverage A2	# Peptides A2	# PSM A2	Score B2	Coverage B2	# Peptides B2	# PSM B2	Score C2	Coverage C2	# Peptides C2	# PSM C2	Score D2	Coverage D2	# Peptides D2	# PSM D2
T2GMZ9	0.00	2.19	1	1												
A0A0F0LCP7	21.29	2.69	1	1												
A0A011T9U1	0.00	2.51	1	1												
A0A098U5P6	0.00	3.72	1	1												
A0A031M416	0.00	3.82	1	1												
A0A011UWW4	0.00	2.95	1	1												
G0RUM1	0.00	3.03	1	1												
C7YUF6	0.00	0.70	1	1												
S6VEF8	0.00	1.52	1	1												
W9NWG2	33.42	7.45	1	1												
K0K2X4	0.00	3.58	1	1												
I8QQH1	0.00	6.08	1	1												
S2F291	0.00	2.03	1	1												
L7U6L6	0.00	2.53	1	1												
A0A0A2W5H1	0.00	3.09	1	1												
A0A0D1LPU8	0.00	3.37	1	1												

	A10				B10				C10				D10			
Accession	Score A2	Coverage A2	# Peptides A2	# PSM A2	Score B2	Coverage B2	# Peptides B2	# PSM B2	Score C2	Coverage C2	# Peptides C2	# PSM C2	Score D2	Coverage D2	# Peptides D2	# PSM D2
A1C5B6	0.00	4.76	1	1												
D3D8T4	28.34	3.99	1	1												
A0A0A4GVG8		0.00														
D2SEG1	34.65	3.46	1	1												
I0RI17	0.00	3.97	1	1												
A0A0F4U1F0		0.00			0.00	3.68	1	1								
F3P7I7	0.00	3.87	1	1												
A0A028V6D9	0.00	10.00	1	1												
W6TPS6	0.00	1.91	1	1												
A0A031MB14	0.00	2.37	1	1												
S6TXI0	0.00	7.37	1	1												
A0A0D7ECH2	0.00	3.70	1	1												
A0A0D0K6Z4	0.00	3.76	1	1												
A0A0B6AJK2	0.00	6.08	1	1												
A0A024M4C4	0.00	8.10	1	1												
A0A073K5A1	0.00	2.99	1	1												
W4R200	0.00	3.95	1	1												

	A10				B10				C10				D10			
Accession	Score A2	Coverage A2	# Peptides A2	# PSM A2	Score B2	Coverage B2	# Peptides B2	# PSM B2	Score C2	Coverage C2	# Peptides C2	# PSM C2	Score D2	Coverage D2	# Peptides D2	# PSM D2
G8RGU7	0.00	6.67	1	1												
A0A0A8X5E9	0.00	7.98	1	1												
R0H2U3						0.00										
B6H2S2					0.00	0.92	1	1								
N1V6C2						0.00										
D5JBG8					0.00	2.02	1	1								
A3IFX0					0.00	2.27	1	1								
J7XK69					0.00	1.37	1	1						0.00		
K9GZT5					0.00	1.64	1	1								
A3IBM8					0.00	3.11	1	1								
W5J0N0					0.00	8.57	1	1								
J2S452					0.00	1.38	1	1								
X7XR24					0.00	3.32	1	1								
E1UUP6					0.00	2.23	1	1								
V9X2B0						0.00							0.00	0.79	1	1
A0A0D0UXL3					0.00	10.77	1	1								
A0A062VT07					0.00	5.83	1	1								
A0A0D0WUY0					0.00	2.72	1	1								

	A10				B10				C10				D10			
Accession	Score A2	Coverage A2	# Peptides A2	# PSM A2	Score B2	Coverage B2	# Peptides B2	# PSM B2	Score C2	Coverage C2	# Peptides C2	# PSM C2	Score D2	Coverage D2	# Peptides D2	# PSM D2
I3DTS6						0.00			0.00	2.33	1	1		0.00		
X0L2P5					0.00	1.14	1	1								
J1HF28						0.00										
G7XIF6					0.00	0.80	1	1								
A0A0D1NIM0					0.00	0.81	1	1								
K5BKC8					0.00	3.56	1	1								
A0A0A2KUU6					0.00	2.09	1	1								
A0A077LEH7					0.00	2.19	1	1								
J8MG65					0.00	1.32	1	1								
I1RLA4					0.00	0.85	1	1								
E5WG96					0.00	7.58	1	1								
A0A077LQU3					0.00	3.94	1	1								
A0A0F5HJJ3					0.00	8.20	1	1								
A0A024JV63					0.00	2.74	1	1								
A0A0G0A566					0.00	3.63	1	1								
A0A0F7DZ16					0.00	3.04	1	1								
R9CKL5					0.00	5.24	1	1								

	A10				B10				C10				D10			
Accession	Score A2	Coverage A2	# Peptides A2	# PSM A2	Score B2	Coverage B2	# Peptides B2	# PSM B2	Score C2	Coverage C2	# Peptides C2	# PSM C2	Score D2	Coverage D2	# Peptides D2	# PSM D2
N1V2N9					0.00	5.88	1	1								
J8DLV5									28.54	2.67	1	1				
A1CQU3									0.00	1.97	1	1				
A0A0A2V3K7										0.00						
A0A024K1D6										0.00						
A0A0D8BND0									0.00	2.99	1	1				
F5YWZ3									0.00	5.23	1	1				
A0A015JCA9										0.00						
A0A0C1BUI9									0.00	4.97	1	1				
A0A0F4XU85									0.00	5.26	1	1				
F0EEG1									0.00	5.43	1	1				
A0A0A2IGV6									0.00	1.16	1	1				
A0A0B5CL03									0.00	0.80	1	1				
W0H4I4										0.00			0.00	0.66	1	1
A0A0A1I278									0.00	0.57	1	1				
A0A0D0FXI4									0.00	2.11	1	1				
B1J556									0.00	2.40	1	1				

	A10				B10				C10				D10			
Accession	Score A2	Coverage A2	# Peptides A2	# PSM A2	Score B2	Coverage B2	# Peptides B2	# PSM B2	Score C2	Coverage C2	# Peptides C2	# PSM C2	Score D2	Coverage D2	# Peptides D2	# PSM D2
A0A0A2L1D5									0.00	1.42	1	1				
A0A0A1YR67									0.00	12.12	1	1				
A0A0C5BYJ1									0.00	3.10	1	1				
A0A090CF27									0.00	0.38	1	1				
A0A031LZF0									49.57	9.62	1	1				
A0A0D8BCI2									0.00	3.31	1	1				
L1M425									0.00	0.76	1	1				
A0A086WAL2									0.00	11.76	1	1				
S2VXJ7									0.00	3.59	1	1				
B2B614									0.00	4.70	1	1				
A0A0B7JH19									0.00	0.25	1	1				
A0A0D6SU66									38.16	3.67	1	1				
S6I769									0.00	5.17	1	1				
A0A0D0JBW8									0.00	1.30	1	1				
Q7TUF7									0.00	2.65	1	1				
G2X640									0.00	4.04	1	1				
A0A0D9AGE0									31.93	5.71	1	1				

	A10				B10				C10				D10			
Accession	Score A2	Coverage A2	# Peptides A2	# PSM A2	Score B2	Coverage B2	# Peptides B2	# PSM B2	Score C2	Coverage C2	# Peptides C2	# PSM C2	Score D2	Coverage D2	# Peptides D2	# PSM D2
G8RQY9													0.00	2.68	1	1
U2SE16													0.00	1.14	1	1
F2ZLR9													0.00	14.71	1	1
Q5ATH9													0.00	1.31	1	1
Q1I5G9													0.00	0.91	1	1
D3D6E8													0.00	4.65	1	1
S8AST1													0.00	1.01	1	1
S6VZT7													0.00	5.14	1	1
Q8NJ38													0.00	7.05	1	1
C6XUS7													0.00	3.36	1	1
A6CMZ6													0.00	4.06	1	1
Q0CH27													0.00	2.70	1	1
A0A0C2S9W8													0.00	2.95	1	1
A0A0C1DB62													0.00	1.36	1	1
A3IAL0													0.00	3.41	1	1
B6HS39													0.00	0.50	1	1
Q5WHI9													0.00	4.32	1	1

	A10				B10				C10				D10			
Accession	Score A2	Coverage A2	# Peptides A2	# PSM A2	Score B2	Coverage B2	# Peptides B2	# PSM B2	Score C2	Coverage C2	# Peptides C2	# PSM C2	Score D2	Coverage D2	# Peptides D2	# PSM D2
Q5B1N2													0.00	0.56	1	1
A0A010RWA6													0.00	2.16	1	1
S6H896													0.00	3.33	1	1
J2NKM8													0.00	2.09	1	1
A0A0D9NE40													0.00	1.32	1	1
A1C7Z1													0.00	0.81	1	1
A0A0C1WV03													0.00	1.88	1	1
A0A0F8WI72														0.00		
A0A0A2JAC8													0.00	1.04	1	1
V6T2S8													0.00	0.78	1	1
A0A0E4CRD1													0.00	6.25	1	1
A0A015JHH0													0.00	4.11	1	1
A0A0C2HVW5													0.00	20.00	1	1
A0A0E2WL07													0.00	2.28	1	1
Q1AY45													0.00	2.47	1	1
A0A0D2XJG1													0.00	2.46	1	1
S8AWJ2													0.00	0.83	1	1

	A10				B10				C10				D10			
Accession	Score A2	Coverage A2	# Peptides A2	# PSM A2	Score B2	Coverage B2	# Peptides B2	# PSM B2	Score C2	Coverage C2	# Peptides C2	# PSM C2	Score D2	Coverage D2	# Peptides D2	# PSM D2
A0A0A8JD12													0.00	3.25	1	1
A0A0E4H2B3													0.00	1.72	1	1
J8VCL6													0.00	12.64	1	1
A0A099S0Q6													0.00	5.95	1	1
F4CMF9													0.00	1.92	1	1
R8QAL6													0.00	1.87	1	1
A0A086A3H6													0.00	2.34	1	1
A0A083UNL6													0.00	5.31	1	1
G9P620													0.00	1.39	1	1
A0A0F5KV12													0.00	13.19	1	1
Q0RMP9													0.00	26.53	1	1
T2HDR7													0.00	4.55	1	1
A0A077EF30													0.00	3.36	1	1
S8AS60													0.00	4.31	1	1
F3CDS5													0.00	44.74	1	1
A0A0B8VLI6													0.00	2.62	1	1
S6P2P9																
U2XR86																
A0A0A1Z132																

	A10				B10				C10				D10			
Accession	Score A2	Coverage A2	# Peptides A2	# PSM A2	Score B2	Coverage B2	# Peptides B2	# PSM B2	Score C2	Coverage C2	# Peptides C2	# PSM C2	Score D2	Coverage D2	# Peptides D2	# PSM D2
H0JGX1																
D5GPN9																
F4CPU4																
F9G3E6																
J3HGL5																
C4RIX2																
A1C3C7																
A6CUG3																
Q50177																
D5GKN9																
W9D2K2																
F3G1S6																
A0A061JS92																
A0A0F0KM13																
R0GBH8																
A0A085CIB6																
A0A028VDZ0																

	A10				B10				C10				D10			
Accession	Score A2	Coverage A2	# Peptides A2	# PSM A2	Score B2	Coverage B2	# Peptides B2	# PSM B2	Score C2	Coverage C2	# Peptides C2	# PSM C2	Score D2	Coverage D2	# Peptides D2	# PSM D2
A0A0D8BBH4																
L8AT32																
A0A0F0F9B2																
S6TVS3																
W9NFV5																
A0A074TM82																
Q2UEU4																
W6QLJ0																
R0HRK1																
L1LRP3																
I2FG13																
Q5WKV9																
A0A099CG66																
E8ND94																
G8X7L2																
A0A0D2YKP5																
A0A086WCF6																
A0A0F2R7M9																

	A10				B10				C10				D10			
Accession	Score A2	Coverage A2	# Peptides A2	# PSM A2	Score B2	Coverage B2	# Peptides B2	# PSM B2	Score C2	Coverage C2	# Peptides C2	# PSM C2	Score D2	Coverage D2	# Peptides D2	# PSM D2
X5LF85																
Q53D73																
N0AYG0																
D8PDK9																
A0A096PFI9																
E4TCS5																
A0A077FBH6																
X8CIL2																
A0A015I5U3																
A0A0C7CQZ9																
H0QH02																
D2B009																
A0A0D1AYE2																
A0A0A2KRG3																
A0A0F8V3G8																
D3CYF5																
U7DIU2																
G9MQ72																

	A10				B10				C10				D10			
Accession	Score A2	Coverage A2	# Peptides A2	# PSM A2	Score B2	Coverage B2	# Peptides B2	# PSM B2	Score C2	Coverage C2	# Peptides C2	# PSM C2	Score D2	Coverage D2	# Peptides D2	# PSM D2
A0A010SPQ5																
E1VSE9																
A0A099MVQ1																
V7MM41																
A0A0A4GKY2																
A0A0F4SMF9																
G7XI77																
A0A024M0C4																
A0A0F4TJB0																
X8AV24																
A0A0D0M7J5																
A0A077FJA6																
J0WGP0																
A0A097F8L6																
U1U9Y9																
M4X557																
D5PB09																
G9P7S1																

	A10				B10				C10				D10			
Accession	Score A2	Coverage A2	# Peptides A2	# PSM A2	Score B2	Coverage B2	# Peptides B2	# PSM B2	Score C2	Coverage C2	# Peptides C2	# PSM C2	Score D2	Coverage D2	# Peptides D2	# PSM D2
R0FV13																
U9U829																
A0A023X530																
A0A015LZ30																
H9T851																
C3HDD4																
R0HZ92																
A0JT14																
E6TXI0																
K9FA32																
I3SSN9																
R0GQV3																
I3SU87																
R0IHA5																
W8GN23																
Q2PEP8																
Q3LVN1																

	A10				B10				C10				D10			
Accession	Score A2	Coverage A2	# Peptides A2	# PSM A2	Score B2	Coverage B2	# Peptides B2	# PSM B2	Score C2	Coverage C2	# Peptides C2	# PSM C2	Score D2	Coverage D2	# Peptides D2	# PSM D2
D5G6T3																
R0HEU4																
G3LKE9																
H9BPH6																
I3T7W2																
H9T7Q2																
R0GR76																
J7LJ06																
Q0RK08																
Q1AVY1																
Q9MB61																
J1SXQ9																
D8PD20																
I3SQF0																
Q43781																
W9MDE0																
U4LD80																
A0A0A8JM98																
U1Z025																

	A10				B10				C10				D10			
Accession	Score A2	Coverage A2	# Peptides A2	# PSM A2	Score B2	Coverage B2	# Peptides B2	# PSM B2	Score C2	Coverage C2	# Peptides C2	# PSM C2	Score D2	Coverage D2	# Peptides D2	# PSM D2
Q88C94																
C2UY19																
A0A051TRF4																
W9NIB8																
R8MNB2																
K0K3B8																
A0A0D7XC92																
A0A0F7BPC5																
I3UZG1																
I3SLT0																
Q0CHN8																
J3E9X9																
A0A0F0ICQ7																
G9QLS3																
A0A078M4Q5																
A0A0D0GRZ7																
A0A0A8JM46																
W9IY52																

	A10				B10				C10				D10			
Accession	Score A2	Coverage A2	# Peptides A2	# PSM A2	Score B2	Coverage B2	# Peptides B2	# PSM B2	Score C2	Coverage C2	# Peptides C2	# PSM C2	Score D2	Coverage D2	# Peptides D2	# PSM D2
A0A024QWF0																
G9N9Q5																
A0A0A8JEH9																
F8AWN3																
R0IFZ3																
A0A0D6SZ14																
R0EZ20																
A0A024M601																
I3S8F0																
A0A078MHP2																
W9T7U3																
I3TAM1																
A0A085VDE2																
A0A0D0VX62																
R0F3J6																
A0A0D9AZE0																
A0A0C1FSI5																
A0A0F4TTX1																

	A10				B10				C10				D10			
Accession	Score A2	Coverage A2	# Peptides A2	# PSM A2	Score B2	Coverage B2	# Peptides B2	# PSM B2	Score C2	Coverage C2	# Peptides C2	# PSM C2	Score D2	Coverage D2	# Peptides D2	# PSM D2
S0DX72																
P52416																
I3DWI3																
R0HN35																
S0DWL1																
B2CBB3																
I3S077																
G7X7D9																
Q50442																
U2XBN4																
U1Q602																
F3HN82																
A0A075EAM1																
D5GL90																
V7K184																
R0FUG6																
V7JIP4																
Q2B9M8																

	A10				B10				C10				D10			
Accession	Score A2	Coverage A2	# Peptides A2	# PSM A2	Score B2	Coverage B2	# Peptides B2	# PSM B2	Score C2	Coverage C2	# Peptides C2	# PSM C2	Score D2	Coverage D2	# Peptides D2	# PSM D2
A0A0D9NAR3																
Q0CDA9																
A0A0C3E2C5																
J9AIS4																
A0A0A2J8X6																
R4UL46																
G7XPT4																
W4HZN3																
A0A0D1P625																
A0A0D0MCU6																
G9MZ07																
T5KKJ6																
I0S2G8																
A0A0C1CMS5																
U3HNX5																
A0A060LYD6																
A0A0E3XTF2																
A0A0F0LWE5																

	A10				B10				C10				D10			
Accession	Score A2	Coverage A2	# Peptides A2	# PSM A2	Score B2	Coverage B2	# Peptides B2	# PSM B2	Score C2	Coverage C2	# Peptides C2	# PSM C2	Score D2	Coverage D2	# Peptides D2	# PSM D2
A0A0C2XXU8																
R0GVN5																
X8BEW3																
A0A062VS01																
A0A0C1ENM9																
A0A0A1Z4D5																
C9SAY9																
A1T133																
A0A016PQ79																
D3D2P4																
A0A0F8UYB0																
X8AHA4																
A0A096AF01																
E5WTC9																
A0A0F0LYB2																
Q9F0I1																
A0A0E8P0D6																
K1KIU5																

	A10				B10				C10				D10			
Accession	Score A2	Coverage A2	# Peptides A2	# PSM A2	Score B2	Coverage B2	# Peptides B2	# PSM B2	Score C2	Coverage C2	# Peptides C2	# PSM C2	Score D2	Coverage D2	# Peptides D2	# PSM D2
R4M166																
A0A0D9MWF6																
S6J9V2																
E3J3W9																
S6ISY7																
D5GIC0																
A4IMU2																
A8F9K2																
A0A078MLM6																
K0V2B2																
G9MZ13																
A0A086T652																
W6QGP6																
A0A078M003																
W7VCE4																
E3IYQ7																
F8CAY8																
Q9RBD9																

	A10				B10				C10				D10			
Accession	Score A2	Coverage A2	# Peptides A2	# PSM A2	Score B2	Coverage B2	# Peptides B2	# PSM B2	Score C2	Coverage C2	# Peptides C2	# PSM C2	Score D2	Coverage D2	# Peptides D2	# PSM D2
S0EDC7																
C3AQN5																
F4DPY6																
A0A0F0KHB3																
A0A0F8TZF3																
M4WYC2																
V7KVR6																
N1RTR0																
Q7TUY3																
J0XS87																
A0A0D9M4U1																
M7MQK9																
B2B7L5																
A0A0D6GAW8																
F9G053																
I9KG21																
Q7V650																

	A10				B10				C10				D10			
Accession	Score A2	Coverage A2	# Peptides A2	# PSM A2	Score B2	Coverage B2	# Peptides B2	# PSM B2	Score C2	Coverage C2	# Peptides C2	# PSM C2	Score D2	Coverage D2	# Peptides D2	# PSM D2
A0A0F7TGE8																
A0A0F0I304																
A0A0C3E1D2																
U9TV30																
H7EXR9																

	A1I				B1I				C1I				D1I			
Accession	Score E2	Coverage E2	# Peptides E2	# PSM E2	Score F2	Coverage F2	# Peptides F2	# PSM F2	Score G2	Coverage G2	# Peptides G2	# PSM G2	Score H2	Coverage H2	# Peptides H2	# PSM H2
A0A015J624	70.67	14.67	4	5	104.52	14.93	4	6	0.00	14.13	4	8	52.00	16.80	6	10
C4MMH6	0.00	12.64	2	3	86.85	12.64	2	4	0.00	15.24	3	6	0.00	15.24	3	6
Q6UDA0	70.67	7.43	2	3	108.26	15.12	4	6	0.00	4.77	1	1	0.00	7.43	2	5
A0A031M2U0					77.47	6.10	4	6					0.00	5.36	3	3
U2ZLC7					86.09	5.68	3	4	0.00	4.76	3	3	62.28	6.96	4	4
A0A031M2A5					67.86	2.58	2	4					0.00	5.35	3	3
A0A031M511					77.47	7.33	4	6								
D5G725	54.25	18.68	2	4	28.27	18.68	2	3	0.00	24.18	3	5	59.36	24.18	3	7
Q1AXU6					48.57	3.52	2	2					0.00	3.15	2	2
E7BCR3					69.99	28.21	3	3	0.00	25.64	2	4				
D8PD19					67.86	2.56	2	4								
D9J011	70.67	2.65	1	1	49.78	10.34	3	3					0.00	2.65	1	1
A0A097NU89					144.00	12.54	6	10								
D2S4D2	114.07	5.21	2	5												
F8CQR1					55.03	3.48	2	2	0.00	2.93	2	2	0.00	4.40	3	3
N1V613					65.44	1.87	1	1	0.00	3.18	2	2	62.28	3.18	2	2
X5C9W2					245.23	26.13	4	10								
D2B144	0.00	13.73	5	6												

	A1I				B1I				C1I				D1I			
Accession	Score E2	Coverage E2	# Peptides E2	# PSM E2	Score F2	Coverage F2	# Peptides F2	# PSM F2	Score G2	Coverage G2	# Peptides G2	# PSM G2	Score H2	Coverage H2	# Peptides H2	# PSM H2
D5G530					75.50	9.57	4	6								
A0A0D9N6M8					69.89	7.72	3	5								
P42653					235.33	22.99	6	9								
U3MV00					46.52	26.50	6	9								
A0A023X6K3	148.68	8.71	2	4												
Q1D2S1					78.74	3.46	2	3					0.00	3.64	2	2
A0A0A2C600									0.00	3.26	2	2	0.00	3.26	2	2
C2ZER9					29.30	7.63	1	3	0.00	7.63	1	2	0.00	7.63	1	2
F4CRF7	0.00	7.67	3	5												
C0VYK7	0.00	2.99	1	3												
A0A023X255	99.37	5.92	2	3												
F8CAY9					78.74	3.46	2	3								
A0A0B4CW17					35.65	2.46	1	1	0.00	2.46	1	2	0.00	2.46	1	1
S6WAM9					63.44	7.06	1	3					0.00	7.06	1	1
A0A0D0V2Q0	0.00	2.40	1	1	39.96	2.40	1	1								
A0A024QIB3	85.34	4.95	1	3												
A0A031LYZ6	97.72	6.54	2	3												
R0IPS1					74.93	6.62	3	5								

	A1I				B1I				C1I				D1I			
Accession	Score E2	Coverage E2	# Peptides E2	# PSM E2	Score F2	Coverage F2	# Peptides F2	# PSM F2	Score G2	Coverage G2	# Peptides G2	# PSM G2	Score H2	Coverage H2	# Peptides H2	# PSM H2
A0A086WFH7													56.06	13.79	1	1
H0QKE9					48.52	4.71	1	1	0.00	4.71	1	1	0.00	4.71	1	1
D2X5N3					46.22	22.73	4	6								
P31926					81.50	5.58	3	6								
Q2PEX3					60.76	11.51	5	6								
K3VXF8	0.00	0.38	1	1		0.00							0.00	0.38	1	1
D2SGA0	0.00	8.20	2	3												
A0A023X1G9	43.54	7.56	1	2												
A0A0F0HPK1	0.00	3.41	2	2												
G3FEZ4					50.42	5.83	1	1	0.00	5.83	1	1				
I3S5Q3					48.12	17.76	5	5								
Q684J8					251.61	5.71	2	5								
D5GF49													154.39	37.04	3	5
A1UHF2	0.00	6.20	2	2												
N1V4B6	79.52	3.71	1	2												
Q07838	45.21	6.40	2	2												
R0HQP2	0.00	2.45	1	1	41.78	4.90	2	2								
E5WLJ3	0.00	3.71	1	1												

	A1I				B1I				C1I				D1I			
Accession	Score E2	Coverage E2	# Peptides E2	# PSM E2	Score F2	Coverage F2	# Peptides F2	# PSM F2	Score G2	Coverage G2	# Peptides G2	# PSM G2	Score H2	Coverage H2	# Peptides H2	# PSM H2
A0A066WMI9	0.00	1.98	1	1	62.03	1.98	1	1								
X8BKS3	62.75	18.06	1	1												
A0A024M4M9	0.00	7.69	1	1					0.00	7.69	1	1	0.00	7.69	1	1
A0A023X435					0.00	0.62	1	2								
J2MVZ3																
I0L6Q5					44.50	0.61	1	1	0.00	0.61	1	1	0.00	0.61	1	1
B1PZ38																
D5GIA8	0.00	7.35	2	4												
A0A024HBV6	82.36	9.65	3	4												
U5IBT8					50.02	10.99	2	4								
R0GZH8					81.83	6.39	3	4								
R0HG12					102.08	6.87	3	4								
I3SNN1					93.00	10.10	2	4								
I3S3D3					86.14	20.00	2	4								
D5GLX2					96.60	14.29	1	1	0.00	14.29	1	2	0.00	14.29	1	1
I3T0F4					207.48	11.78	2	4								
I3T8P4					53.83	12.15	1	4								

	A1I				B1I				C1I				D1I			
Accession	Score E2	Coverage E2	# Peptides E2	# PSM E2	Score F2	Coverage F2	# Peptides F2	# PSM F2	Score G2	Coverage G2	# Peptides G2	# PSM G2	Score H2	Coverage H2	# Peptides H2	# PSM H2
C8VFF7	0.00	1.33	1	1												
M2V5V0																
D2S8V2	0.00	1.43	1	1												
Q8GI57	58.26	5.83	1	1												
Q0CKI2	0.00	2.23	1	1												
B5AEM9	0.00	2.07	1	1												
U2AB71	0.00	2.26	1	1												
L8TPB7	0.00	3.17	1	1												
A0A099CPS9	0.00	2.72	1	1					0.00	2.72	1	1				
A0A031MCA3	0.00	3.49	2	2												
X8FJG5	66.70	10.62	1	2												
A0A0D0WZL0																
A0A074TMY9	0.00	10.73	1	1												
W7VQP0	0.00	1.36	1	1												
F3EXH9	0.00	2.35	1	1												
A0A0F7P5R1	0.00	5.56	1	2												

	A1I				B1I				C1I				D1I			
Accession	Score E2	Coverage E2	# Peptides E2	# PSM E2	Score F2	Coverage F2	# Peptides F2	# PSM F2	Score G2	Coverage G2	# Peptides G2	# PSM G2	Score H2	Coverage H2	# Peptides H2	# PSM H2
I3T598									0.00	4.79	1	1				
A0A0E4CR92									0.00	8.86	1	1				
A0A0A1Z7Q1													0.00	16.33	1	1
X7Z574										0.00			0.00	5.56	1	1
W9N0A1																
U2SUS3									0.00	1.73	1	1	0.00	1.73	1	1
A0A0C2I3T2																
L8AJT4	0.00	3.06	1	3												
D5GIK5	0.00	3.01	2	3												
D5GLX7	160.58	11.22	1	3												
I3SZE9					47.90	16.11	2	3								
R0G5L8					63.37	10.44	3	3								
R0GR46					49.42	3.92	3	3								
Q58ZF1					61.37	8.35	3	3								
R0F5B5					56.92	4.49	2	3								
R0INE5					44.62	27.63	3	3								
A0A059XMU8					63.48	8.66	3	3								
D2BBD1													64.72	4.07	2	3

	A1I				B1I				C1I				D1I			
Accession	Score E2	Coverage E2	# Peptides E2	# PSM E2	Score F2	Coverage F2	# Peptides F2	# PSM F2	Score G2	Coverage G2	# Peptides G2	# PSM G2	Score H2	Coverage H2	# Peptides H2	# PSM H2
Q1AUM3	0.00	2.27	1	1												
U3HB76	0.00	3.14	1	1												
A0A081I584	68.32	7.95	1	1												
J3AB25																
L0IVW3																
A0A0D7FCN8																
Q1AZT7																
A4VIS3																
W4PWG4	0.00	1.20	1	1												
A0A064CC06																
A0A031M6T0		0.00														
X8C966	0.00	7.82	1	1												
A0A077LJN0	0.00	3.76	1	1												
A0A023X481																
A0A0F0H9G5		0.00			0.00	6.50	1	1	0.00	6.50	1	1		0.00		
X8FHU5	0.00	4.44	1	1												
A0A024EJ81	0.00	13.19	1	1												

	A1I				B1I				C1I				D1I			
Accession	Score E2	Coverage E2	# Peptides E2	# PSM E2	Score F2	Coverage F2	# Peptides F2	# PSM F2	Score G2	Coverage G2	# Peptides G2	# PSM G2	Score H2	Coverage H2	# Peptides H2	# PSM H2
A0A0F7BTD3	39.15	5.02	1	1												
D2B9Y7	0.00	2.12	1	1												
W4Q7M1	0.00	2.22	1	1												
A0A023WYP6																
C7YL24																
G4I7D7																
C7Z1H4																
B2B374					0.00	0.51	1	1								
S6IZP4																
A0A0F5MQC0													0.00	3.64	1	1
F9F4B5																
A0A085FS16																
A0A0B4KEB7																
A0A017SQ96																
A0A0D0EX19																
A0A0F0HX18	0.00	1.31	1	1												
A0A085FJB3																

	A1I				B1I				C1I				D1I			
Accession	Score E2	Coverage E2	# Peptides E2	# PSM E2	Score F2	Coverage F2	# Peptides F2	# PSM F2	Score G2	Coverage G2	# Peptides G2	# PSM G2	Score H2	Coverage H2	# Peptides H2	# PSM H2
G9G3Y9																
A0A0A1CW01																
K0XAB6																
U1S6V9																
I0LBY2	0.00	2.47	1	2												
N1VC98	0.00	4.46	1	2												
D5GAH8	63.59	9.50	1	2												
D5GHM3	39.38	11.74	1	2												
W7ZA32	71.18	5.32	2	2												
G2Z5G4					34.01	1.81	1	1	0.00	1.81	1	1				
J3C4Y2					42.41	2.15	2	2								
I3SH51					0.00	2.99	2	2								
R0GU86					49.12	12.10	2	2								
R0GKE5					26.95	4.77	2	2								
R0HKB9					64.13	3.89	2	2								
R0FNU6					32.72	6.31	2	2								
Q9XHC7					39.47	1.04	1	2								
R0FV42					55.38	4.63	2	2								

	A1I				B1I				C1I				D1I			
Accession	Score E2	Coverage E2	# Peptides E2	# PSM E2	Score F2	Coverage F2	# Peptides F2	# PSM F2	Score G2	Coverage G2	# Peptides G2	# PSM G2	Score H2	Coverage H2	# Peptides H2	# PSM H2
R0GRF1					53.41	5.45	1	2								
D8PIF0					51.27	9.33	1	1	0.00	9.33	1	1				
R0FX04					68.97	4.62	1	2								
E3Q1S2					97.11	17.81	1	2								
R0FHR7					54.09	9.72	2	2								
I3T4F6					47.80	4.04	1	2								
I3T5V4					29.76	3.85	1	2								
Q2PEW2					42.91	8.86	2	2								
I3T2W0					48.78	12.32	2	2								
Q3LVQ8					143.34	29.85	1	2								
M5R2U9					0.00	2.76	1	1					0.00	2.76	1	1
Q6Q4Z3					39.91	5.41	2	2								
I3S0W6					39.81	5.49	1	2								
R0HNM5					25.29	8.02	1	2								
W7VC77					0.00	2.63	1	1					0.00	2.63	1	1
R0HKA2					44.54	4.25	1	2								
A0A075EAM3					43.44	6.43	2	2								
R0I261					0.00	4.12	2	2								
D6PPS9					0.00	13.90	2	2								
A0A059WQB8					48.95	6.29	1	2								

	A1I				B1I				C1I				D1I			
Accession	Score E2	Coverage E2	# Peptides E2	# PSM E2	Score F2	Coverage F2	# Peptides F2	# PSM F2	Score G2	Coverage G2	# Peptides G2	# PSM G2	Score H2	Coverage H2	# Peptides H2	# PSM H2
C2NS66					0.00	3.83	1	2								
A0A0A0E634					0.00	0.99	1	1					0.00	0.99	1	1
I3S9M6					39.19	12.22	1	2								
U4L650					44.29	2.97	2	2								
R0G9G5					44.29	1.50	1	1	0.00	1.25	1	1				
I3S358					37.58	3.25	1	2								
I3S2A7					0.00	8.67	1	2								
A0A0B7JX17					31.94	1.57	1	2								
Q83XX0									0.00	1.79	1	2				
Q0REU0									0.00	2.30	1	1	0.00	2.30	1	1
S7ZEP9									0.00	5.90	1	2				
G9QDX3									0.00	21.65	1	2				
G8RQS2	0.00	2.39	1	1												
G8RMF4																
S7U6Y1																
Q1ASQ4																
K0WK83																
R7YW04																
D8PA76																

	A1I				B1I				C1I				D1I			
Accession	Score E2	Coverage E2	# Peptides E2	# PSM E2	Score F2	Coverage F2	# Peptides F2	# PSM F2	Score G2	Coverage G2	# Peptides G2	# PSM G2	Score H2	Coverage H2	# Peptides H2	# PSM H2
J2UVE8																
Q1AT20																
Q1ASC0																
A0JTM5		0.00														
E7NB53																
Q1ATJ8																
A0A0D6IES8																
K9DBA8																
A1UAH7																
Q1AUH6																
A0A0B4EI70		0.00														
X8DPH6																
Z2DAB9																
A0A023X607																
A0A0C2TM17																
A0A0A1YKY1																
A0A088NID7																

	A1I				B1I				C1I				D1I			
Accession	Score E2	Coverage E2	# Peptides E2	# PSM E2	Score F2	Coverage F2	# Peptides F2	# PSM F2	Score G2	Coverage G2	# Peptides G2	# PSM G2	Score H2	Coverage H2	# Peptides H2	# PSM H2
A0A090M9Q3																
W1SN17																
A0A024GYA6																
A0A0F7E1Y7		0.00				0.00				0.00						
A0A024GZA8	0.00	2.10	1	1												
W4HSK4																
J2TJZ9																
F8AXU2																
B3Z4Z6																
A0A0C1C4E1																
A0A090M9M4																
G9NEH9																
K0K6R6																
K3UCX8																
C5IY18																
A0A0F7BPL2																
I3SJR5														0.00		

	A1I				B1I				C1I				D1I			
Accession	Score E2	Coverage E2	# Peptides E2	# PSM E2	Score F2	Coverage F2	# Peptides F2	# PSM F2	Score G2	Coverage G2	# Peptides G2	# PSM G2	Score H2	Coverage H2	# Peptides H2	# PSM H2
A0A0D1NDU4													0.00	1.35	1	1
A0A0F5MWK6																
L8F5X2																
C7ZM70																
A0A0A2A1V6																
A0A0C3AIH0																
A0A0F0HT56																
A0A024M803																
A0A0B7KIN2																
A0A024HKW1																
C7ZLV7																
L0IRX5																
A0A0C9T0H7																
A0A098F8J9																
A0A010TBX4																
A0A016PEV8																

	A1I				B1I				C1I				D1I			
Accession	Score E2	Coverage E2	# Peptides E2	# PSM E2	Score F2	Coverage F2	# Peptides F2	# PSM F2	Score G2	Coverage G2	# Peptides G2	# PSM G2	Score H2	Coverage H2	# Peptides H2	# PSM H2
T2GMZ9																
A0A0F0LCP7																
A0A011T9U1																
A0A098U5P6																
A0A031M416																
A0A011UWW4																
G0RUM1																
C7YUF6																
S6VEF8																
W9NWG2																
K0K2X4																
I8QQH1																
S2F291																
L7U6L6																
A0A0A2W5H1																
A0A0D1LPU8																

	A1I				B1I				C1I				D1I			
Accession	Score E2	Coverage E2	# Peptides E2	# PSM E2	Score F2	Coverage F2	# Peptides F2	# PSM F2	Score G2	Coverage G2	# Peptides G2	# PSM G2	Score H2	Coverage H2	# Peptides H2	# PSM H2
A1C5B6																
D3D8T4																
A0A0A4GVG8	0.00	6.81	1	1												
D2SEG1																
I0RI17																
A0A0F4U1F0																
F3P7I7																
A0A028V6D9																
W6TPS6																
A0A031MB14																
S6TXI0																
A0A0D7ECH2																
A0A0D0K6Z4																
A0A0B6AJK2																
A0A024M4C4																
A0A073K5A1																
W4R200																

	A1I				B1I				C1I				D1I			
Accession	Score E2	Coverage E2	# Peptides E2	# PSM E2	Score F2	Coverage F2	# Peptides F2	# PSM F2	Score G2	Coverage G2	# Peptides G2	# PSM G2	Score H2	Coverage H2	# Peptides H2	# PSM H2
G8RGU7																
A0A0A8X5E9																
R0H2U3	0.00	1.11	1	1		0.00										
B6H2S2																
N1V6C2		0.00			0.00	1.53	1	1								
D5JBG8																
A3IFX0																
J7XK69																
K9GZT5																
A3IBM8																
W5J0N0																
J2S452																
X7XR24																
E1UUP6																
V9X2B0																
A0A0D0UXL3																
A0A062VT07																
A0A0D0WUY0																

	A1I				B1I				C1I				D1I			
Accession	Score E2	Coverage E2	# Peptides E2	# PSM E2	Score F2	Coverage F2	# Peptides F2	# PSM F2	Score G2	Coverage G2	# Peptides G2	# PSM G2	Score H2	Coverage H2	# Peptides H2	# PSM H2
I3DTS6										0.00						
X0L2P5																
J1HF28					0.00	1.65	1	1								
G7XIF6																
A0A0D1NIM0																
K5BKC8																
A0A0A2KUU6																
A0A077LEH7																
J8MG65																
I1RLA4																
E5WG96																
A0A077LQU3																
A0A0F5HJJ3																
A0A024JV63																
A0A0G0A566																
A0A0F7DZ16																
R9CKL5																

	A1I				B1I				C1I				D1I			
Accession	Score E2	Coverage E2	# Peptides E2	# PSM E2	Score F2	Coverage F2	# Peptides F2	# PSM F2	Score G2	Coverage G2	# Peptides G2	# PSM G2	Score H2	Coverage H2	# Peptides H2	# PSM H2
N1V2N9																
J8DLV5																
A1CQU3																
A0A0A2V3K7													0.00	2.35	1	1
A0A024K1D6													0.00	2.06	1	1
A0A0D8BND0														0.00		
F5YWZ3																
A0A015JCA9													0.00	1.83	1	1
A0A0C1BUI9																
A0A0F4XU85																
F0EEG1																
A0A0A2IGV6																
A0A0B5CL03																
W0H4I4																
A0A0A1I278																
A0A0D0FXI4																
B1J556																

	A1I				B1I				C1I				D1I			
Accession	Score E2	Coverage E2	# Peptides E2	# PSM E2	Score F2	Coverage F2	# Peptides F2	# PSM F2	Score G2	Coverage G2	# Peptides G2	# PSM G2	Score H2	Coverage H2	# Peptides H2	# PSM H2
A0A0A2L1D5																
A0A0A1YR67																
A0A0C5BYJ1																
A0A090CF27																
A0A031LZF0																
A0A0D8BCI2																
L1M425																
A0A086WAL2																
S2VXJ7																
B2B614																
A0A0B7JH19																
A0A0D6SU66																
S6I769																
A0A0D0JBW8																
Q7TUF7																
G2X640																
A0A0D9AGE0																

	A1I				B1I				C1I				D1I			
Accession	Score E2	Coverage E2	# Peptides E2	# PSM E2	Score F2	Coverage F2	# Peptides F2	# PSM F2	Score G2	Coverage G2	# Peptides G2	# PSM G2	Score H2	Coverage H2	# Peptides H2	# PSM H2
G8RQY9																
U2SE16																
F2ZLR9																
Q5ATH9																
Q1I5G9																
D3D6E8						0.00										
S8AST1																
S6VZT7																
Q8NJ38																
C6XUS7																
A6CMZ6																
Q0CH27																
A0A0C2S9W8																
A0A0C1DB62																
A3IAL0																
B6HS39																
Q5WHI9																

	A1I				B1I				C1I				D1I			
Accession	Score E2	Coverage E2	# Peptides E2	# PSM E2	Score F2	Coverage F2	# Peptides F2	# PSM F2	Score G2	Coverage G2	# Peptides G2	# PSM G2	Score H2	Coverage H2	# Peptides H2	# PSM H2
Q5B1N2																
A0A010RWA6																
S6H896																
J2NKM8																
A0A0D9NE40																
A1C7Z1																
A0A0C1WV03										0.00						
A0A0F8WI72									0.00	0.36	1	1				
A0A0A2JAC8																
V6T2S8																
A0A0E4CRD1																
A0A015JHH0																
A0A0C2HVW5																
A0A0E2WL07																
Q1AY45																
A0A0D2XJG1																
S8AWJ2																

	A1I				B1I				C1I				D1I			
Accession	Score E2	Coverage E2	# Peptides E2	# PSM E2	Score F2	Coverage F2	# Peptides F2	# PSM F2	Score G2	Coverage G2	# Peptides G2	# PSM G2	Score H2	Coverage H2	# Peptides H2	# PSM H2
A0A0A8JD12																
A0A0E4H2B3																
J8VCL6																
A0A099S0Q6																
F4CMF9																
R8QAL6																
A0A086A3H6																
A0A083UNL6																
G9P620																
A0A0F5KV12																
Q0RMP9																
T2HDR7																
A0A077EF30																
S8AS60																
F3CDS5																
A0A0B8VLI6																
S6P2P9		0.00			0.00	7.34	1	1								
U2XR86	0.00	1.62	1	1												
A0A0A1Z132	0.00	1.68	1	1												

	A1I				B1I				C1I				D1I			
Accession	Score E2	Coverage E2	# Peptides E2	# PSM E2	Score F2	Coverage F2	# Peptides F2	# PSM F2	Score G2	Coverage G2	# Peptides G2	# PSM G2	Score H2	Coverage H2	# Peptides H2	# PSM H2
H0JGX1	0.00	2.11	1	1												
D5GPN9	0.00	6.71	1	1												
F4CPU4	0.00	4.07	1	1												
F9G3E6	0.00	3.76	1	1												
J3HGL5	0.00	6.84	1	1												
C4RIX2	0.00	4.18	1	1												
A1C3C7	55.28	8.37	1	1												
A6CUG3	0.00	2.70	1	1												
Q50177	0.00	0.64	1	1												
D5GKN9	0.00	1.32	1	1												
W9D2K2	0.00	0.81	1	1												
F3G1S6	0.00	5.09	1	1												
A0A061JS92	0.00	5.83	1	1												
A0A0F0KM13	0.00	3.88	1	1												
R0GBH8	0.00	4.63	1	1												
A0A085CIB6	0.00	0.87	1	1												
A0A028VDZ0	0.00	2.52	1	1												

	A1I				B1I				C1I				D1I			
Accession	Score E2	Coverage E2	# Peptides E2	# PSM E2	Score F2	Coverage F2	# Peptides F2	# PSM F2	Score G2	Coverage G2	# Peptides G2	# PSM G2	Score H2	Coverage H2	# Peptides H2	# PSM H2
A0A0D8BBH4	62.55	7.85	1	1												
L8AT32	0.00	4.32	1	1												
A0A0F0F9B2	0.00	1.40	1	1												
S6TVS3	0.00	3.23	1	1												
W9NFV5	0.00	1.10	1	1												
A0A074TM82	0.00	2.88	1	1												
Q2UEU4	0.00	3.98	1	1												
W6QLJ0	0.00	1.57	1	1												
R0HRK1	0.00	2.00	1	1												
L1LRP3	0.00	2.68	1	1												
I2FG13	0.00	1.99	1	1												
Q5WKV9	0.00	2.02	1	1												
A0A099CG66	0.00	2.80	1	1		0.00										
E8ND94	0.00	1.07	1	1												
G8X7L2	0.00	2.04	1	1												
A0A0D2YKP5	0.00	1.64	1	1												
A0A086WCF6	0.00	2.51	1	1												
A0A0F2R7M9	0.00	2.38	1	1												

	A1I				B1I				C1I				D1I			
Accession	Score E2	Coverage E2	# Peptides E2	# PSM E2	Score F2	Coverage F2	# Peptides F2	# PSM F2	Score G2	Coverage G2	# Peptides G2	# PSM G2	Score H2	Coverage H2	# Peptides H2	# PSM H2
X5LF85		0.00											0.00	0.47	1	1
Q53D73	0.00	2.57	1	1												
N0AYG0	0.00	1.32	1	1												
D8PDK9	0.00	1.59	1	1												
A0A096PFI9	0.00	0.88	1	1												
E4TCS5	0.00	4.04	1	1												
A0A077FBH6	0.00	3.61	1	1												
X8CIL2	0.00	7.36	1	1												
A0A015I5U3	0.00	0.83	1	1												
A0A0C7CQZ9	0.00	1.46	1	1												
H0QH02	0.00	10.38	1	1												
D2B009	0.00	5.42	1	1												
A0A0D1AYE2	0.00	1.04	1	1												
A0A0A2KRG3	0.00	3.79	1	1												
A0A0F8V3G8	0.00	8.33	1	1												
D3CYF5	0.00	2.98	1	1												
U7DIU2	0.00	1.12	1	1												
G9MQ72	0.00	2.19	1	1												

	A1I				B1I				C1I				D1I			
Accession	Score E2	Coverage E2	# Peptides E2	# PSM E2	Score F2	Coverage F2	# Peptides F2	# PSM F2	Score G2	Coverage G2	# Peptides G2	# PSM G2	Score H2	Coverage H2	# Peptides H2	# PSM H2
A0A010SPQ5	0.00	3.68	1	1												
E1VSE9	0.00	1.05	1	1												
A0A099MVQ1	0.00	10.58	1	1												
V7MM41	0.00	5.70	1	1												
A0A0A4GKY2	0.00	7.78	1	1												
A0A0F4SMF9	47.84	11.61	1	1												
G7XI77	0.00	2.13	1	1												
A0A024M0C4	0.00	3.67	1	1												
A0A0F4TJB0	0.00	3.76	1	1												
X8AV24	0.00	5.73	1	1												
A0A0D0M7J5	0.00	5.20	1	1												
A0A077FJA6	0.00	7.91	1	1												
J0WGP0	0.00	6.12	1	1												
A0A097F8L6	0.00	1.50	1	1												
U1U9Y9	0.00	7.14	1	1												
M4X557	0.00	6.91	1	1												
D5PB09	0.00	1.14	1	1												
G9P7S1	0.00	2.56	1	1												

	A1I				B1I				C1I				D1I			
Accession	Score E2	Coverage E2	# Peptides E2	# PSM E2	Score F2	Coverage F2	# Peptides F2	# PSM F2	Score G2	Coverage G2	# Peptides G2	# PSM G2	Score H2	Coverage H2	# Peptides H2	# PSM H2
R0FV13	0.00	2.81	1	1												
U9U829	0.00	13.57	1	1												
A0A023X530	0.00	14.81	1	1												
A0A015LZ30	0.00	8.26	1	1												
H9T851					33.22	19.40	1	1								
C3HDD4					0.00	1.41	1	1								
R0HZ92					57.52	1.69	1	1								
A0JT14					45.93	3.81	1	1								
E6TXI0					0.00	1.10	1	1								
K9FA32					35.38	1.89	1	1								
I3SSN9					55.86	6.16	1	1								
R0GQV3					43.97	2.84	1	1								
I3SU87					40.53	4.88	1	1								
R0IHA5					55.60	6.22	1	1								
W8GN23					49.86	4.60	1	1								
Q2PEP8					35.27	2.60	1	1								
Q3LVN1					49.06	10.81	1	1								

	A1I				B1I				C1I				D1I			
Accession	Score E2	Coverage E2	# Peptides E2	# PSM E2	Score F2	Coverage F2	# Peptides F2	# PSM F2	Score G2	Coverage G2	# Peptides G2	# PSM G2	Score H2	Coverage H2	# Peptides H2	# PSM H2
D5G6T3					24.98	4.02	1	1								
R0HEU4					52.99	1.17	1	1								
G3LKE9					45.97	6.86	1	1								
H9BPH6					58.50	11.28	1	1								
I3T7W2					39.25	4.23	1	1								
H9T7Q2					51.24	9.92	1	1								
R0GR76					26.07	4.74	1	1								
J7LJ06					25.83	0.80	1	1								
Q0RK08					0.00	1.35	1	1								
Q1AVY1					0.00	3.82	1	1								
Q9MB61					0.00	3.08	1	1								
J1SXQ9					29.71	4.95	1	1								
D8PD20					0.00	9.00	1	1								
I3SQF0					0.00	6.94	1	1								
Q43781					29.37	3.35	1	1								
W9MDE0					32.37	7.05	1	1								
U4LD80					0.00	0.36	1	1								
A0A0A8JM98					0.00	1.16	1	1								
U1Z025					0.00	2.22	1	1								

	A1I				B1I				C1I				D1I			
Accession	Score E2	Coverage E2	# Peptides E2	# PSM E2	Score F2	Coverage F2	# Peptides F2	# PSM F2	Score G2	Coverage G2	# Peptides G2	# PSM G2	Score H2	Coverage H2	# Peptides H2	# PSM H2
Q88C94					0.00	15.22	1	1								
C2UY19					0.00	5.77	1	1								
A0A051TRF4					0.00	3.01	1	1								
W9NIB8					0.00	1.54	1	1								
R8MNB2					46.69	4.38	1	1								
K0K3B8					0.00	9.86	1	1								
A0A0D7XC92					0.00	4.05	1	1								
A0A0F7BPC5					0.00	4.60	1	1								
I3UZG1					35.10	3.60	1	1								
I3SLT0					35.81	4.62	1	1								
Q0CHN8					34.28	3.20	1	1								
J3E9X9					0.00	1.95	1	1								
A0A0F0ICQ7					0.00	1.40	1	1								
G9QLS3					0.00	1.09	1	1								
A0A078M4Q5					0.00	1.24	1	1								
A0A0D0GRZ7					0.00	1.04	1	1								
A0A0A8JM46					0.00	2.14	1	1								
W9IY52					0.00	0.97	1	1								

	A1I				B1I				C1I				D1I			
Accession	Score E2	Coverage E2	# Peptides E2	# PSM E2	Score F2	Coverage F2	# Peptides F2	# PSM F2	Score G2	Coverage G2	# Peptides G2	# PSM G2	Score H2	Coverage H2	# Peptides H2	# PSM H2
A0A024QWF0					0.00	3.75	1	1								
G9N9Q5					28.17	1.66	1	1								
A0A0A8JEH9					32.61	2.84	1	1								
F8AWN3					0.00	2.61	1	1								
R0IFZ3					31.13	5.16	1	1								
A0A0D6SZ14					30.48	1.28	1	1								
R0EZ20					0.00	7.46	1	1								
A0A024M601					0.00	2.28	1	1								
I3S8F0					43.50	7.19	1	1								
A0A078MHP2					0.00	3.55	1	1								
W9T7U3					0.00	3.03	1	1								
I3TAM1					38.04	5.88	1	1								
A0A085VDE2					42.45	13.24	1	1								
A0A0D0VX62					0.00	2.13	1	1								
R0F3J6					33.75	1.98	1	1								
A0A0D9AZE0					0.00	4.76	1	1								
A0A0C1FSI5					36.83	1.89	1	1								
A0A0F4TTX1					42.83	0.76	1	1								

	A1I				B1I				C1I				D1I			
Accession	Score E2	Coverage E2	# Peptides E2	# PSM E2	Score F2	Coverage F2	# Peptides F2	# PSM F2	Score G2	Coverage G2	# Peptides G2	# PSM G2	Score H2	Coverage H2	# Peptides H2	# PSM H2
S0DX72					0.00	1.75	1	1								
P52416					33.83	2.36	1	1								
I3DWI3					23.90	1.06	1	1								
R0HN35					0.00	4.44	1	1								
S0DWL1					34.78	1.44	1	1								
B2CBB3					0.00	2.86	1	1								
I3S077					0.00	3.69	1	1								
G7X7D9					34.20	0.67	1	1								
Q50442					28.77	6.51	1	1								
U2XBN4					30.37	2.43	1	1								
U1Q602					24.30	2.58	1	1								
F3HN82					22.72	3.76	1	1								
A0A075EAM1					32.47	6.25	1	1								
D5GL90									0.00	2.76	1	1				
V7K184									0.00	3.05	1	1				
R0FUG6									0.00	2.03	1	1				
V7JIP4									0.00	1.69	1	1				
Q2B9M8									0.00	3.49	1	1				

	A1I				B1I				C1I				D1I			
Accession	Score E2	Coverage E2	# Peptides E2	# PSM E2	Score F2	Coverage F2	# Peptides F2	# PSM F2	Score G2	Coverage G2	# Peptides G2	# PSM G2	Score H2	Coverage H2	# Peptides H2	# PSM H2
A0A0D9NAR3									0.00	2.36	1	1				
Q0CDA9									0.00	2.45	1	1				
A0A0C3E2C5									0.00	7.50	1	1				
J9AIS4									0.00	4.59	1	1				
A0A0A2J8X6									0.00	0.08	1	1				
R4UL46									0.00	2.51	1	1				
G7XPT4									0.00	2.63	1	1				
W4HZN3									0.00	1.34	1	1				
A0A0D1P625									0.00	2.07	1	1				
A0A0D0MCU6									0.00	1.49	1	1				
G9MZ07									0.00	1.34	1	1				
T5KKJ6									0.00	3.20	1	1				
I0S2G8									0.00	4.35	1	1				
A0A0C1CMS5									0.00	1.11	1	1				
U3HNX5									0.00	3.23	1	1				
A0A060LYD6									0.00	3.60	1	1				
A0A0E3XTF2									0.00	3.40	1	1				
A0A0F0LWE5									0.00	2.21	1	1				

	A1I				B1I				C1I				D1I			
Accession	Score E2	Coverage E2	# Peptides E2	# PSM E2	Score F2	Coverage F2	# Peptides F2	# PSM F2	Score G2	Coverage G2	# Peptides G2	# PSM G2	Score H2	Coverage H2	# Peptides H2	# PSM H2
A0A0C2XXU8									0.00	21.62	1	1				
R0GVN5									0.00	1.88	1	1				
X8BEW3									0.00	4.90	1	1				
A0A062VS01									0.00	4.83	1	1				
A0A0C1ENM9									0.00	1.54	1	1				
A0A0A1Z4D5									0.00	5.29	1	1				
C9SAY9									0.00	2.07	1	1				
A1T133									0.00	2.33	1	1				
A0A016PQ79									0.00	1.02	1	1				
D3D2P4									0.00	0.92	1	1				
A0A0F8UYB0									0.00	0.53	1	1				
X8AHA4									0.00	21.74	1	1				
A0A096AF01									0.00	10.68	1	1				
E5WTC9									0.00	3.24	1	1				
A0A0F0LYB2									0.00	1.59	1	1				
Q9F0I1									0.00	6.94	1	1				
A0A0E8P0D6									0.00	8.17	1	1				
K1KIU5													0.00	3.90	1	1

	A1I				B1I				C1I				D1I			
Accession	Score E2	Coverage E2	# Peptides E2	# PSM E2	Score F2	Coverage F2	# Peptides F2	# PSM F2	Score G2	Coverage G2	# Peptides G2	# PSM G2	Score H2	Coverage H2	# Peptides H2	# PSM H2
R4M166													0.00	2.20	1	1
A0A0D9MWF6													0.00	0.79	1	1
S6J9V2													0.00	2.79	1	1
E3J3W9													0.00	1.51	1	1
S6ISY7													0.00	9.38	1	1
D5GIC0													0.00	3.83	1	1
A4IMU2													0.00	0.64	1	1
A8F9K2													0.00	1.35	1	1
A0A078MLM6													0.00	3.16	1	1
K0V2B2													0.00	2.16	1	1
G9MZ13													0.00	1.32	1	1
A0A086T652													0.00	2.71	1	1
W6QGP6													0.00	1.47	1	1
A0A078M003													0.00	2.56	1	1
W7VCE4													0.00	2.17	1	1
E3IYQ7													0.00	2.46	1	1
F8CAY8													0.00	9.38	1	1
Q9RBD9													0.00	4.50	1	1

	A1I				B1I				C1I				D1I			
Accession	Score E2	Coverage E2	# Peptides E2	# PSM E2	Score F2	Coverage F2	# Peptides F2	# PSM F2	Score G2	Coverage G2	# Peptides G2	# PSM G2	Score H2	Coverage H2	# Peptides H2	# PSM H2
S0EDC7													0.00	6.67	1	1
C3AQN5													0.00	1.33	1	1
F4DPY6													0.00	1.74	1	1
A0A0F0KHB3													0.00	1.95	1	1
A0A0F8TZF3													0.00	1.75	1	1
M4WYC2													0.00	1.95	1	1
V7KVR6													0.00	14.29	1	1
N1RTR0													0.00	6.13	1	1
Q7TUY3													0.00	8.59	1	1
J0XS87													0.00	3.05	1	1
A0A0D9M4U1													0.00	2.50	1	1
M7MQK9													0.00	2.26	1	1
B2B7L5													0.00	2.68	1	1
A0A0D6GAW8													0.00	5.66	1	1
F9G053													0.00	1.94	1	1
I9KG21													0.00	2.11	1	1
Q7V650													0.00	2.30	1	1

	A1I				B1I				C1I				D1I			
Accession	Score E2	Coverage E2	# Peptides E2	# PSM E2	Score F2	Coverage F2	# Peptides F2	# PSM F2	Score G2	Coverage G2	# Peptides G2	# PSM G2	Score H2	Coverage H2	# Peptides H2	# PSM H2
A0A0F7TGE8													0.00	1.59	1	1
A0A0F0I304													0.00	1.20	1	1
A0A0C3E1D2													0.00	2.95	1	1
U9TV30													0.00	7.17	1	1
H7EXR9													0.00	2.63	1	1

Supplementary Table S2: Proteins identified in the Tuber melanosporum database.

Accession (indicates the protein ID), gi (indicates the protein Gene Index number from NCBI), UNIPROT (indicates the ID from UNIPROT website), desc (indicates the protein description), Description (indicates the origin of the sequences), ∑Coverage (indicates the total sum of all coverages), ∑#Proteins (indicates the total sum of all proteins identified), ∑#Unique Peptides (indicates the total sum of all unique peptides), ∑#Peptides (indicates the total sum of all identified peptides), ∑#PSMs (indicates the total sum of all the peptide-spectrum match), score (indicates the MASCOT score), #AAs (indicates the protein length), MW (indicates the protein weight), calc pI (indicates the calculated pI).

Accession	UNIPROT	Description	ΣCoverage	Σ# Proteins	Σ# Unique Peptides	Σ# Peptides	Σ# PSMs
GSTUMT00001850001	Q9UT45|ENG1_SCHPO	No hit found	37.04	1	3	3	5
GSTUMT00010275001	P61859|CALM_NEUCR	hypothetical protein SNOG_12830 [Phaeosphaeria nodorum SN15]	31.63	1	4	4	16
GSTUMT00006386001	No hit found	No hit found	28.21	1	1	1	1
GSTUMT00011582001	P61864|UBIQ_YEAST	hypothetical protein Kpol_1070p6 [Vanderwaltozyma polyspora DSM 70294]	24.92	5	2	2	2
GSTUMT00004570001	No hit found	No hit found	24.18	1	3	3	19

Accession	UNIPROT	Description	ΣCoverage	Σ# Proteins	Σ# Unique Peptides	Σ# Peptides	Σ# PSMs
GSTUMT00010038001	No hit found	No hit found	23.26	1	1	1	1
GSTUMT00000409001	P04914|H4_NEUCR	Histone H4.1 sp|Q76MU7|H4_ASPOR Histone H4 emb|CAA39155.1| H4.1 [Emericella nidulans]	21.36	1	2	2	3
GSTUMT00006163001	O13419|ACT_BOTFU	YlACT1 [Yarrowia lipolytica]	16.57	1	3	3	41
GSTUMT00006162001	P10365|ACT_THELA	actin [Magnaporthe grisea 70-15]	16.39	1	1	1	6
GSTUMT00003270001	No hit found	No hit found	16.13	1	1	1	1
GSTUMT00010457001	No hit found	hypothetical protein CHGG_06730 [Chaetomium globosum CBS 148.51]	14.29	1	1	1	6

Accession	UNIPROT	Description	ΣCoverage	Σ# Proteins	Σ# Unique Peptides	Σ# Peptides	Σ# PSMs
GSTUMT00000720001	No hit found	No hit found	12.31	1	1	1	1
GSTUMT00008041001	P34735|YLU2_PICAN	hypothetical protein An03g00250 [Aspergillus niger]	11.74	1	1	1	2
GSTUMT00010465001	No hit found	hypothetical protein SNOG_15858 [Phaeosphaeria nodorum SN15]	11.22	1	1	1	3
GSTUMT00000288001	O93866|HSP70_TRIRU	heat shock 70 kDa protein [Coccidioides immitis RS]	10.99	1	4	5	10
GSTUMT00008579001	Q6BRN7|DUT_DEBHA	hypothetical protein Kpol_182p1 [Vanderwaltozyma polyspora DSM 70294]	9.71	1	1	1	1
GSTUMT00003593001	No hit found	No hit found	9.5	1	1	1	2

Accession	UNIPROT	Description	ΣCoverage	Σ# Proteins	Σ# Unique Peptides	Σ# Peptides	Σ# PSMs
GSTUMT00001163001	No hit found	No hit found	9.47	1	1	1	1
GSTUMT00001328001	No hit found	No hit found	8.79	1	1	1	1
GSTUMT00007325001	P53733|RT19_YEAST	mitochondrial ribosomal protein S19, putative [Aspergillus fumigatus A1163]	8.33	1	1	1	5
GSTUMT00005607001	No hit found	No hit found	7.77	1	1	1	1
GSTUMT00007614001	A5DN78|EFTU_PICGU	hypothetical protein Kpol_1009p12 [Vanderwaltozyma polyspora DSM 70294]	7.44	1	1	1	1
GSTUMT00008389001	No hit found	hypothetical protein CHGG_00107 [Chaetomium globosum CBS 148.51]	7.35	1	2	2	4

Accession	UNIPROT	Description	ΣCoverage	Σ# Proteins	Σ# Unique Peptides	Σ# Peptides	Σ# PSMs
GSTUMT00007211001	P04451|RL23_YEAST	hypothetical protein HCAG_04519 [Ajellomyces capsulatus NAm1]	7.14	1	1	1	1
GSTUMT00003125001	P12547|ORYZ_ASPOR	hypothetical protein SS1G_12605 [Sclerotinia sclerotiorum 1980]	7.11	1	2	2	2
GSTUMT00011952001	No hit found	GPI anchored CFEM domain protein [Monacrosporium haptotylum]	6.71	1	1	1	1
GSTUMT00003402001	Q9P720|RL16_NEUCR	60S ribosomal protein L16 [Chaetomium globosum CBS 148.51]	6.57	1	1	1	1
GSTUMT00007957001	P36579|ARF1_SCHPO	ADP-ribosylation factor 1 [Coprinopsis cinerea okayama7#130]	6.08	1	1	1	1
GSTUMT00003753001	P38891|BCA1_YEAST	hypothetical protein SNOG_06787 [Phaeosphaeria nodorum SN15]	6.02	1	1	1	1

Accession	UNIPROT	Description	ΣCoverage	Σ# Proteins	Σ# Unique Peptides	Σ# Peptides	Σ# PSMs
GSTUMT00004943001	O60076|TAF13_SCHPO	hypothetical protein [Yarrowia lipolytica]	5.83	1	1	1	2
GSTUMT00003005001	Q9RPT1|RHLG_PSEAE	hypothetical protein NCU03693 [Neurospora crassa OR74A]	5.76	1	1	1	1
GSTUMT00001214001	No hit found	No hit found	5.69	1	1	1	1
GSTUMT00000021001	Q01765|EF1A_PODCU	elongation factor 1-alpha [Botryotinia fuckeliana B05.10]	5.54	1	2	2	5
GSTUMT00010075001	No hit found	conserved hypothetical protein [Rhizobium leguminosarum bv. trifolii WSM1325]	5.28	1	2	2	4
GSTUMT00002474001	P23704|ATPB_NEUCR	ATP synthase beta chain [Coccidioides posadasii]	5.21	1	2	2	4

Accession	UNIPROT	Description	ΣCoverage	Σ# Proteins	Σ# Unique Peptides	Σ# Peptides	Σ# PSMs
GSTUMT00009481001	P62494|RB11A_RAT	hypothetical protein BC1G_09346 [Botryotinia fuckeliana B05.10]	5.19	1	1	1	2
GSTUMT00011335001	P04146|COPIA_DROME	putative protein [Arabidopsis thaliana]	5.17	1	1	1	1
GSTUMT00005695001	Q05654|RT21_SCHPO	hypothetical protein BC1G_09710 [Botryotinia fuckeliana B05.10]	5.13	4	1	1	1
GSTUMT00001678001	Q9P4E9|GSP1_CANAL	GTP-binding nuclear protein GSP1/Ran [Lodderomyces elongisporus NRRL YB-4239]	5.09	1	1	1	1
GSTUMT00005706001	Q09867|YAG2_SCHPO	hypothetical protein SS1G_07329 [Sclerotinia sclerotiorum 1980]	5	1	1	1	1
GSTUMT00006122001	Q12016|VPS68_YEAST	UPF0220 domain protein [Aspergillus clavatus NRRL 1]	4.76	1	1	1	1

Accession	UNIPROT	Description	ΣCoverage	Σ# Proteins	Σ# Unique Peptides	Σ# Peptides	Σ# PSMs
GSTUMT00008018001	P27073|RS19_EMENI	ribosomal protein S16 [Ajellomyces capsulatus NAm1]	4.61	1	1	1	1
GSTUMT00003527001	Q9BW72|HIG2A_HUMAN	hypothetical protein BC1G_15810 [Botryotinia fuckeliana B05.10]	4.58	1	1	1	1
GSTUMT00002908001	P38624|PSB6_YEAST	hypothetical protein AN3756.2 [Aspergillus nidulans FGSC A4]	4.37	1	1	1	1
GSTUMT00006299001	Q05654|RT21_SCHPO	hypothetical protein CNBD5450 [Cryptococcus neoformans var. neoformans B-3501A]	4.14	1	1	1	1
GSTUMT00005677001	Q10113|MAL3_SCHPO	hypothetical protein SS1G_07164 [Sclerotinia sclerotiorum 1980]	4.13	1	1	1	3
GSTUMT00007883001	Q00808|HETE1_PODAN	unnamed protein product [Aspergillus oryzae]	4.13	1	1	1	1

Accession	UNIPROT	Description	ΣCoverage	Σ# Proteins	Σ# Unique Peptides	Σ# Peptides	Σ# PSMs
GSTUMT00010477001	P83616|GRP78_ASPNG	dnaK-type molecular chaperone bipA-Aspergillus niger sp|P59769|GRP78_ASPAW 78 kDa glucose-regulated protein homolog precursor (GRP 78) (Immunoglobulin heavy chain-binding protein homolog) (BiP) sp|P83616|GRP78_ASPNG 78 kDa glucose-regulated protein homolo	4.12	1	1	2	3
GSTUMT00001231001	No hit found	hypothetical protein BC1G_14528 [Botryotinia fuckeliana B05.10]	4.11	1	1	1	1
GSTUMT00002258001	Q8K274|KT3K_MOUSE	hypothetical protein BC1G_02432 [Botryotinia fuckeliana B05.10]	4.02	1	1	1	1
GSTUMT00009943001	No hit found	No hit found	4.01	1	1	1	1
GSTUMT00009267001	No hit found	No hit found	3.89	1	1	1	1
GSTUMT00008418001	No hit found	hypothetical protein CC1G_03339 [Coprinopsis cinerea okayama7#130]	3.83	1	1	1	1

Accession	UNIPROT	Description	ΣCoverage	Σ# Proteins	Σ# Unique Peptides	Σ# Peptides	Σ# PSMs
GSTUMT00008015001	Q9P727|SUCA_NEUCR	unnamed protein product [Aspergillus oryzae]	3.68	1	1	1	1
GSTUMT00001583001	O00625|PIR_HUMAN	pirin [Neosartorya fischeri NRRL 181]	3.64	1	1	1	1
GSTUMT00008511001	A5UQR2|MOAC_ROSS1	molybdenum cofactor biosynthesis protein 1 B [Neurospora crassa OR74A]	3.54	1	1	1	1
GSTUMT00012196001	P49018|GPI8_YEAST	hypothetical protein BC1G_14244 [Botryotinia fuckeliana B05.10]	3.47	1	1	1	1
GSTUMT00001470001	No hit found	hypothetical protein BC1G_04038 [Botryotinia fuckeliana B05.10]	3.45	1	1	1	1
GSTUMT00003199001	Q8X034|RL15_NEUCR	60S ribosomal protein L15 [Ajellomyces capsulatus NAm1]	3.45	1	1	1	1

Accession	UNIPROT	Description	ΣCoverage	Σ# Proteins	Σ# Unique Peptides	Σ# Peptides	Σ# PSMs
GSTUMT00003201001	Q05024|YM74_YEAST	unnamed protein product [Aspergillus oryzae]	3.4	1	1	1	1
GSTUMT00001220001	No hit found	No hit found	3.32	1	1	1	1
GSTUMT00000125001	O13991|YEG9_SCHPO	hypothetical protein An17g00210 [Aspergillus niger]	3.29	1	1	1	1
GSTUMT00011316001	P78774|ARPC1_SCHPO	hypothetical protein NCU02781 [Neurospora crassa OR74A]	3.21	1	1	1	1
GSTUMT00002085001	No hit found	zinc metalloproteinase, putative [Aspergillus clavatus NRRL 1]	3.2	1	1	1	2
GSTUMT00004210001	P54865|XYND_CELFI	hypothetical protein SNOG_07620 [Phaeosphaeria nodorum SN15]	3.18	1	1	1	1

Accession	UNIPROT	Description	ΣCoverage	Σ# Proteins	Σ# Unique Peptides	Σ# Peptides	Σ# PSMs
GSTUMT00002243001	Q9USH7|PSY1_SCHPO	hypothetical protein BC1G_08548 [Botryotinia fuckeliana B05.10]	3.16	1	1	1	1
GSTUMT00011958001	Q99002|1433_TRIHA	14-3-3 protein [Botryotinia fuckeliana B05.10]	3.04	1	1	1	1
GSTUMT00011509001	Q1XGE2|HAC1_ASPOR	hypothetical protein An01g00160 [Aspergillus niger]	3.03	1	1	1	1
GSTUMT00006889001	No hit found	No hit found	3.02	1	1	1	1
GSTUMT00001784001	No hit found	No hit found	3.01	1	1	1	1
GSTUMT00008539001	Q92396|TYRO_PODAN	hypothetical protein SS1G_04725 [Sclerotinia sclerotiorum 1980]	3.01	1	2	2	3

Accession	UNIPROT	Description	ΣCoverage	Σ# Proteins	Σ# Unique Peptides	Σ# Peptides	Σ# PSMs
GSTUMT00009800001	Q99002|1433_TRIHA	hypothetical protein BC1G_12947 [Botryotinia fuckeliana B05.10]	3	1	1	1	1
GSTUMT00001821001	No hit found	hypothetical protein An02g09300 [Aspergillus niger]	2.97	1	1	1	1
GSTUMT00010624001	Q9H3J6|CL065_HUMAN	hypothetical protein SS1G_00010 [Sclerotinia sclerotiorum 1980]	2.95	1	1	1	1
GSTUMT00008548001	Q12458|YPR1_YEAST	hypothetical protein CIMG_06398 [Coccidioides immitis RS]	2.94	1	1	1	1
GSTUMT00002670001	Q09674|YA01_SCHPO	DUF1445 domain protein [Aspergillus fumigatus Af293]	2.84	1	1	1	1
GSTUMT00009269001	Q9UR07|RT23_SCHPO	hypothetical protein BC1G_11096 [Botryotinia fuckeliana B05.10]	2.79	1	1	1	8

Accession	UNIPROT	Description	ΣCoverage	Σ# Proteins	Σ# Unique Peptides	Σ# Peptides	Σ# PSMs
GSTUMT00009669001	P34237|CASP_YEAST	hypothetical protein SS1G_07014 [Sclerotinia sclerotiorum 1980]	2.75	1	2	2	3
GSTUMT00002014001	P10978|POLX_TOBAC	copia-type polyprotein, putative [Arabidopsis thaliana]	2.75	1	1	1	1
GSTUMT00010863001	O59760|YJM7_SCHPO	hypothetical protein An11g00640 [Aspergillus niger]	2.63	1	1	1	3
GSTUMT00001207001	No hit found	hypothetical protein MGG_02483 [Magnaporthe grisea 70-15]	2.62	1	1	1	1
GSTUMT00011332001	No hit found	hypothetical protein CHGG_00233 [Chaetomium globosum CBS 148.51]	2.56	1	1	1	1
GSTUMT00011943001	O14442|CP51_UNCNE	sterol 14alpha-demethylase [Pneumocystis carinii]	2.55	1	1	1	1

Accession	UNIPROT	Description	ΣCoverage	Σ# Proteins	Σ# Unique Peptides	Σ# Peptides	Σ# PSMs
GSTUMT00006007001	P14002|BGLB_CLOTH	hypothetical protein AN2217.2 [Aspergillus nidulans FGSC A4]	2.54	1	1	1	1
GSTUMT00006874001	O19048|PCBP1_RABIT	hypothetical protein CIMG_01064 [Coccidioides immitis RS]	2.54	1	1	1	2
GSTUMT00011675001	No hit found	hypothetical protein SS1G_01791 [Sclerotinia sclerotiorum 1980]	2.51	2	1	1	1
GSTUMT00006255001	P04051|RPC1_YEAST	DNA-directed RNA polymerase III largest subunit [Ajellomyces capsulatus NAm1]	2.49	1	1	1	1
GSTUMT00005542001	O67606|METE_AQUAE	truncated methionine synthase-like protein [Salinibacter ruber DSM 13855]	2.39	1	1	1	1
GSTUMT00012817001	No hit found	hypothetical protein An11g00300 [Aspergillus niger]	2.35	1	1	1	1

Accession	UNIPROT	Description	ΣCoverage	Σ# Proteins	Σ# Unique Peptides	Σ# Peptides	Σ# PSMs
GSTUMT00010261001	O94740|CDC37_SCHPO	hypothetical protein BC1G_10364 [Botryotinia fuckeliana B05.10]	2.33	1	1	1	1
GSTUMT00006412001	Q9BRT8|CBWD1_HUMAN	hypothetical protein CIMG_08141 [Coccidioides immitis RS]	2.32	1	1	1	1
GSTUMT00000874001	P36091|DCW1_YEAST	hypothetical protein BC1G_07186 [Botryotinia fuckeliana B05.10]	2.31	1	1	1	2
GSTUMT00007262001	No hit found	conserved hypothetical protein [Neosartorya fischeri NRRL 181]	2.31	1	1	1	1
GSTUMT00010001001	No hit found	predicted protein [Laccaria bicolor S238N-H82]	2.3	2	1	1	2
GSTUMT00003768001	P32496|RPN12_YEAST	hypothetical protein An16g02210 [Aspergillus niger]	2.3	1	1	1	2

Accession	UNIPROT	Description	ΣCoverage	Σ# Proteins	Σ# Unique Peptides	Σ# Peptides	Σ# PSMs
GSTUMT00007761001	Q9SR66|DML2_ARATH	conserved hypothetical protein [Aspergillus fumigatus A1163]	2.27	1	1	1	1
GSTUMT00001334001	P41890|SCN1_SCHPO	Cut9 interacting protein Scn1 [Aspergillus fumigatus Af293]	2.26	1	1	1	1
GSTUMT00011748001	P32785|FMT_YEAST	hypothetical protein NCU04313 [Neurospora crassa OR74A]	2.26	1	1	1	1
GSTUMT00011209001	No hit found	No hit found	2.23	1	1	1	1
GSTUMT00011415001	P39954|SAHH_YEAST	adenosylhomocysteinase [Ajellomyces capsulatus NAm1]	2.16	1	1	1	1
GSTUMT00008736001	P32804|ZRT1_YEAST	hypothetical protein AN1410.2 [Aspergillus nidulans FGSC A4]	2.14	1	1	1	1

Accession	UNIPROT	Description	ΣCoverage	Σ# Proteins	Σ# Unique Peptides	Σ# Peptides	Σ# PSMs
GSTUMT00006281001	O28972|Y1297_ARCFU	AAA family ATPase Rix7 (predicted) [Schizosaccharomyces pombe 972h-]	2.13	1	1	1	1
GSTUMT00002599001	Q9UR07|RT23_SCHPO	hypothetical protein MGG_13837 [Magnaporthe grisea 70-15]	2.12	1	1	1	2
GSTUMT00000583001	No hit found	hypothetical protein AN3674.2 [Aspergillus nidulans FGSC A4]	2.09	1	1	1	2
GSTUMT00012700001	No hit found	hypothetical protein SNOG_13095 [Phaeosphaeria nodorum SN15]	2.07	1	1	1	1
GSTUMT00007685001	No hit found	No hit found	2.05	1	1	1	1
GSTUMT00012729001	O94142|KO1_GIBFU	predicted protein [Laccaria bicolor S238N-H82]	2.05	1	1	1	1

Accession	UNIPROT	Description	ΣCoverage	Σ# Proteins	Σ# Unique Peptides	Σ# Peptides	Σ# PSMs
GSTUMT00010108001	O14435|GBB_CRYPA	hypothetical protein An18g02090 [Aspergillus niger]	2.02	1	1	1	2
GSTUMT00003152001	Q7S8R8|RPN1_NEUCR	26S proteasome regulatory subunit Mts4, putative [Aspergillus clavatus NRRL 1]	2	1	2	2	2
GSTUMT00008198001	No hit found	peroxin 14/17 [Penicillium chrysogenum]	2	1	1	1	1
GSTUMT00004165001	No hit found	kelch repeat protein [Aspergillus fumigatus Af293]	1.86	1	1	1	1
GSTUMT00007887001	Q7RVY5|HEM1_NEUCR	delta-aminolevulinic acid synthase [Phaeosphaeria nodorum SN15]	1.85	1	1	1	1
GSTUMT00001812001	Q9XZC0|LCTA_LATMA	Ankyrin [Thermofilum pendens Hrk 5]	1.85	1	1	1	1

Accession	UNIPROT	Description	ΣCoverage	Σ# Proteins	Σ# Unique Peptides	Σ# Peptides	Σ# PSMs
GSTUMT00004787001	P36013|MAOM_YEAST	hypothetical protein BC1G_13155 [Botryotinia fuckeliana B05.10]	1.85	1	1	1	1
GSTUMT00002932001	No hit found	hypothetical protein MGG_08820 [Magnaporthe grisea 70-15]	1.81	1	2	2	2
GSTUMT00009140001	Q32NW2|LENG8_XENLA	hypothetical protein NCU06594 [Neurospora crassa OR74A]	1.77	1	1	1	1
GSTUMT00004461001	Q91XF4|RN167_MOUSE	hypothetical protein SNOG_02512 [Phaeosphaeria nodorum SN15]	1.77	1	1	1	1
GSTUMT00009834001	Q9UR07|RT23_SCHPO	hypothetical protein MGG_13061 [Magnaporthe grisea 70-15]	1.76	1	1	1	1
GSTUMT00010450001	No hit found	hypothetical protein SNOG_06285 [Phaeosphaeria nodorum SN15]	1.76	1	1	1	1

Accession	UNIPROT	Description	ΣCoverage	Σ# Proteins	Σ# Unique Peptides	Σ# Peptides	Σ# PSMs
GSTUMT00009374001	P52890|ATF1_SCHPO	hypothetical protein An02g07070 [Aspergillus niger]	1.75	1	1	1	1
GSTUMT00011323001	P32363|GPI3_YEAST	unnamed protein product [Aspergillus oryzae]	1.75	1	1	1	1
GSTUMT00005017001	No hit found	No hit found	1.75	1	1	1	1
GSTUMT00008292001	P37302|APE3_YEAST	hypothetical protein CC1G_01543 [Coprinopsis cinerea okayama7#130]	1.73	1	1	1	1
GSTUMT00004969001	P78898|TGCE2_SCHPO	hypothetical protein HCAG_00673 [Ajellomyces capsulatus NAm1]	1.73	1	1	1	1
GSTUMT00003080001	No hit found	No hit found	1.71	1	1	1	1

Accession	UNIPROT	Description	ΣCoverage	Σ# Proteins	Σ# Unique Peptides	Σ# Peptides	Σ# PSMs
GSTUMT00005768001	No hit found	No hit found	1.7	1	1	1	1
GSTUMT00003525001	Q05654|RT21_SCHPO	hypothetical protein [Vitis vinifera]	1.69	1	1	1	1
GSTUMT00010278001	A6SNX1|DBP9_BOTFB	hypothetical protein SS1G_06425 [Sclerotinia sclerotiorum 1980]	1.69	1	1	1	1
GSTUMT00000105001	P38344|REI1_YEAST	predicted protein [Ajellomyces capsulatus NAm1]	1.68	1	1	1	1
GSTUMT00002056001	Q9UR07|RT23_SCHPO	hypothetical protein MGG_13837 [Magnaporthe grisea 70-15]	1.68	1	1	1	1
GSTUMT00000660001	P42251|PPBD_BACSU	hypothetical protein SS1G_10052 [Sclerotinia sclerotiorum 1980]	1.65	1	1	1	1

Accession	UNIPROT	Description	ΣCoverage	Σ# Proteins	Σ# Unique Peptides	Σ# Peptides	Σ# PSMs
GSTUMT00004057001	Q4WUK1|PFA5_ASPFU	DHHC zinc finger membrane protein [Aspergillus fumigatus A1163]	1.63	1	1	1	1
GSTUMT00009838001	O74460|YCGG_SCHPO	unnamed protein product [Aspergillus oryzae]	1.61	1	1	1	1
GSTUMT00000277001	O13853|ITS3_SCHPO	multicopy suppressor of stt4 mutation [Aspergillus clavatus NRRL 1]	1.61	1	1	1	1
GSTUMT00006911001	Q96WM9|LAC2_BOTFU	laccase precursor [Helotiaceae sp. UHH 1-13-18-4]	1.6	1	1	1	1
GSTUMT00007159001	Q5RC46|CECR1_PONPY	hypothetical protein BC1G_09011 [Botryotinia fuckeliana B05.10]	1.6	1	1	1	2
GSTUMT00012580001	No hit found	No hit found	1.57	1	1	1	1

Accession	UNIPROT	Description	ΣCoverage	Σ# Proteins	Σ# Unique Peptides	Σ# Peptides	Σ# PSMs
GSTUMT00012371001	No hit found	hypothetical protein FG03870.1 [Gibberella zeae PH-1]	1.57	1	1	1	1
GSTUMT00004219001	Q1XDF4|CLPC_PORYE	hypothetical protein MGL_3014 [Malassezia globosa CBS 7966]	1.56	1	1	1	2
GSTUMT00005928001	No hit found	No hit found	1.55	1	1	1	1
GSTUMT00002440001	Q10441|TILS_SCHPO	hypothetical protein An18g02520 [Aspergillus niger]	1.53	1	1	1	1
GSTUMT00000106001	No hit found	hypothetical protein SS1G_02405 [Sclerotinia sclerotiorum 1980]	1.53	1	1	1	1
GSTUMT00009341001	O60008|HSP60_PARBR	heat shock protein 60, mitochondrial precursor [Ajellomyces capsulatus NAm1]	1.52	1	1	1	1

Accession	UNIPROT	Description	ΣCoverage	Σ# Proteins	Σ# Unique Peptides	Σ# Peptides	Σ# PSMs
GSTUMT00003847001	Q28653|2A5D_RABIT	conserved hypothetical protein [Sclerotinia sclerotiorum 1980]	1.51	1	1	1	1
GSTUMT00001078001	P36604|GRP78_SCHPO	hypothetical protein SNOG_06313 [Phaeosphaeria nodorum SN15]	1.5	1	1	1	1
GSTUMT00011312001	P42641|YHBZ_ECOLI	hypothetical protein An01g13830 [Aspergillus niger]	1.5	1	1	1	1
GSTUMT00001275001	No hit found	hypothetical protein SNOG_06908 [Phaeosphaeria nodorum SN15]	1.45	1	1	1	1
GSTUMT00004257001	P00692|AMY_BACAM	hypothetical protein SS1G_10101 [Sclerotinia sclerotiorum 1980]	1.42	1	1	1	2
GSTUMT00011007001	Q9ULJ7|ANR50_HUMAN	Ankyrin repeat protein [Aspergillus fumigatus A1163]	1.42	1	1	1	1

Accession	UNIPROT	Description	ΣCoverage	Σ# Proteins	Σ# Unique Peptides	Σ# Peptides	Σ# PSMs
GSTUMT00008074001	Q99385|VCX1_YEAST	conserved hypothetical protein [Aspergillus terreus NIH2624]	1.4	2	1	1	1
GSTUMT00001308001	P23201|SPA2_YEAST	hypothetical protein An07g08290 [Aspergillus niger]	1.39	1	1	1	1
GSTUMT00008696001	P48826|G6PD_ASPNG	hypothetical protein SS1G_12886 [Sclerotinia sclerotiorum 1980]	1.37	1	1	1	1
GSTUMT00003182001	No hit found	conserved hypothetical protein [Aspergillus clavatus NRRL 1]	1.37	1	1	1	1
GSTUMT00004647001	P19807|HNM1_YEAST	unnamed protein product [Aspergillus oryzae]	1.33	1	1	1	1
GSTUMT00004452001	Q9URT2|YJU3_SCHPO	conserved hypothetical protein [Ajellomyces capsulatus NAm1]	1.32	1	1	1	1

Accession	UNIPROT	Description	ΣCoverage	Σ# Proteins	Σ# Unique Peptides	Σ# Peptides	Σ# PSMs
GSTUMT00007016001	Q10332|YBMA_SCHPO	hypothetical protein BC1G_04929 [Botryotinia fuckeliana B05.10]	1.32	1	1	1	1
GSTUMT00009675001	Q1EA11|CCR4_COCIM	conserved hypothetical protein [Ajellomyces capsulatus NAm1]	1.32	1	1	1	2
GSTUMT00010640001	P40010|NUG1_YEAST	hypothetical protein BC1G_11175 [Botryotinia fuckeliana B05.10]	1.31	1	1	1	1
GSTUMT00002283001	P28349|NIT4_NEUCR	hypothetical protein SNOG_08725 [Phaeosphaeria nodorum SN15]	1.31	1	1	1	1
GSTUMT00007918001	P15245|PH2M_TRICU	conserved hypothetical protein [Magnaporthe grisea 70-15]	1.3	1	1	1	1
GSTUMT00001196001	No hit found	No hit found	1.3	1	1	1	1

Accession	UNIPROT	Description	ΣCoverage	Σ# Proteins	Σ# Unique Peptides	Σ# Peptides	Σ# PSMs
GSTUMT00010240001	Q05164|HPF1_YEAST	hypothetical protein BC1G_10428 [Botryotinia fuckeliana B05.10]	1.29	1	1	1	1
GSTUMT00003604001	Q96MU7|YTDC1_HUMAN	hypothetical protein SS1G_00152 [Sclerotinia sclerotiorum 1980]	1.28	1	2	2	2
GSTUMT00005329001	Q0JMY1|PRP2B_ORYSJ	hypothetical protein CC1G_04200 [Coprinopsis cinerea okayama7#130]	1.27	1	1	1	1
GSTUMT00001651001	Q10499|YDGE_SCHPO	hypothetical protein SS1G_00860 [Sclerotinia sclerotiorum 1980]	1.27	1	1	1	1
GSTUMT00006989001	Q96UL8|PPCK_EMENI	phosphoenolpyruvate carboxykinase [Coccidioides immitis RS]	1.26	1	1	1	1
GSTUMT00008547001	Q1HG60|PIF1_RAT	Pif1/Rrm3 DNA-helicase-like protein [Rattus norvegicus]	1.25	1	1	1	1

Accession	UNIPROT	Description	ΣCoverage	Σ# Proteins	Σ# Unique Peptides	Σ# Peptides	Σ# PSMs
GSTUMT00009359001	Q70M86|STRN_SORMA	conserved hypothetical protein [Aspergillus terreus NIH2624]	1.24	1	1	1	1
GSTUMT00008029001	Q9USN3|UTP13_SCHPO	hypothetical protein SS1G_03089 [Sclerotinia sclerotiorum 1980]	1.24	1	1	1	1
GSTUMT00004213001	Q29510|CP2CU_RABIT	hypothetical protein An18g01480 [Aspergillus niger]	1.24	1	1	1	1
GSTUMT00006967001	Q9ULJ7|ANR50_HUMAN	hypothetical protein BC1G_05548 [Botryotinia fuckeliana B05.10]	1.24	1	1	1	1
GSTUMT00005973001	No hit found	hypothetical protein ACLA_087570 [Aspergillus clavatus NRRL 1]	1.23	2	1	1	1
GSTUMT00011138001	P34161|YOX1_YEAST	hypothetical protein AN5048.2 [Aspergillus nidulans FGSC A4]	1.23	1	1	1	1

Accession	UNIPROT	Description	ΣCoverage	Σ# Proteins	Σ# Unique Peptides	Σ# Peptides	Σ# PSMs
GSTUMT00000199001	Q60864|STIP1_MOUSE	conserved hypothetical protein [Coccidioides immitis RS]	1.23	1	1	1	1
GSTUMT00006203001	Q5R5S1|FBX21_PONPY	hypothetical protein SNOG_12123 [Phaeosphaeria nodorum SN15]	1.23	1	1	1	1
GSTUMT00007732001	O14369|SCE3_SCHPO	translation initiation factor 4B [Aspergillus fumigatus Af293]	1.22	1	1	1	1
GSTUMT00008870001	Q12751|YM48_YEAST	hypothetical protein CIMG_09716 [Coccidioides immitis RS]	1.21	1	1	1	1
GSTUMT00004983001	Q9H6R3|ACSS3_HUMAN	acyl-CoA synthetase, putative [Aspergillus fumigatus A1163]	1.21	1	1	1	1
GSTUMT00000086001	O94289|LUB1_SCHPO	hypothetical protein BC1G_00625 [Botryotinia fuckeliana B05.10]	1.2	1	1	1	1

Accession	UNIPROT	Description	ΣCoverage	Σ# Proteins	Σ# Unique Peptides	Σ# Peptides	Σ# PSMs
GSTUMT00010828001	Q8BJ37|TYDP1_MOUSE	unnamed protein product [Aspergillus oryzae]	1.18	1	1	1	1
GSTUMT00005488001	O42637|CIA84_NEUCR	hypothetical protein BC1G_09813 [Botryotinia fuckeliana B05.10]	1.18	1	1	1	1
GSTUMT00010343001	Q9YB37|Y1758_AERPE	hypothetical protein SNOG_06870 [Phaeosphaeria nodorum SN15]	1.15	1	1	1	2
GSTUMT00011768001	Q96KG9|NTKL_HUMAN	hypothetical protein FG10196.1 [Gibberella zeae PH-1]	1.14	1	1	1	1
GSTUMT00005941001	P10978|POLX_TOBAC	hypothetical protein CNBM0210 [Cryptococcus neoformans var. neoformans B-3501A]	1.14	2	1	1	1
GSTUMT00003996001	O13990|BGL2_SCHPO	hypothetical protein SNOG_09734 [Phaeosphaeria nodorum SN15]	1.13	1	1	1	1

Accession	UNIPROT	Description	ΣCoverage	Σ# Proteins	Σ# Unique Peptides	Σ# Peptides	Σ# PSMs
GSTUMT00001258001	Q8TG12|CSK2B_NEUCR	casein kinase II beta 1 subunit [Aspergillus terreus NIH2624]	1.13	1	1	1	1
GSTUMT00000754001	No hit found	hypothetical protein SS1G_04947 [Sclerotinia sclerotiorum 1980]	1.12	1	1	1	1
GSTUMT00010843001	Q9ULJ7|ANR50_HUMAN	hypothetical protein BC1G_05548 [Botryotinia fuckeliana B05.10]	1.12	1	1	1	1
GSTUMT00010787001	No hit found	hypothetical protein SNOG_06691 [Phaeosphaeria nodorum SN15]	1.11	1	1	1	1
GSTUMT00000550001	P31688|TPS2_YEAST	trehalose-phosphatase [Coccidioides immitis RS]	1.1	1	1	1	1
GSTUMT00000204001	P78813|YCTB_SCHPO	conserved hypothetical protein [Aspergillus terreus NIH2624]	1.09	1	1	1	1

Accession	UNIPROT	Description	ΣCoverage	Σ# Proteins	Σ# Unique Peptides	Σ# Peptides	Σ# PSMs
GSTUMT00006380001	Q9SS94|CD48C_ARATH	AAA family ATPase Rix7 (predicted) [Schizosaccharomyces pombe 972h-]	1.07	1	1	1	1
GSTUMT00006298001	No hit found	No hit found	1.06	1	1	1	1
GSTUMT00012201001	Q09897|CHR3_SCHPO	conserved hypothetical protein [Ajellomyces capsulatus NAm1]	1.06	1	1	1	1
GSTUMT00009286001	No hit found	hypothetical protein AN6241.2 [Aspergillus nidulans FGSC A4]	1.04	1	1	1	2
GSTUMT00012289001	Q00808|HETE1_PODAN	NACHT and Ankyrin domain protein [Aspergillus fumigatus Af293]	1.04	1	1	1	1
GSTUMT00002124001	O13790|CUL1_SCHPO	hypothetical protein SNOG_13309 [Phaeosphaeria nodorum SN15]	1.04	1	1	1	1

Accession	UNIPROT	Description	ΣCoverage	Σ# Proteins	Σ# Unique Peptides	Σ# Peptides	Σ# PSMs
GSTUMT00010874001	P05661|MYSA_DROME	involucrin repeat protein, putative [Aspergillus fumigatus A1163]	1.03	1	1	1	1
GSTUMT00001431001	Q4IBS9|BRO1_GIBZE	hypothetical protein BC1G_11654 [Botryotinia fuckeliana B05.10]	1.03	1	1	1	1
GSTUMT00005156001	No hit found	hypothetical protein BC1G_06100 [Botryotinia fuckeliana B05.10]	1.01	1	1	1	1
GSTUMT00000895001	Q10250|YD22_SCHPO	hypothetical protein BC1G_07335 [Botryotinia fuckeliana B05.10]	0.99	1	1	1	2
GSTUMT00011643001	O00534|LHR2A_HUMAN	von Willebrand domain protein [Neosartorya fischeri NRRL 181]	0.98	1	1	1	1
GSTUMT00008433001	Q9Y7K5|YGI3_SCHPO	hypothetical protein SS1G_04255 [Sclerotinia sclerotiorum 1980]	0.97	1	1	1	1

Accession	UNIPROT	Description	ΣCoverage	Σ# Proteins	Σ# Unique Peptides	Σ# Peptides	Σ# PSMs
GSTUMT00010895001	P47025|MDV1_YEAST	hypothetical protein CIMG_09746 [Coccidioides immitis RS]	0.96	1	1	1	1
GSTUMT00011664001	Q12381|PRP1_SCHPO	pre-mRNA splicing factor [Coccidioides immitis RS]	0.96	1	1	1	1
GSTUMT00009187001	Q10264|PSO2_SCHPO	DNA repair protein Pso2/Snm1 [Aspergillus fumigatus Af293]	0.95	1	1	1	1
GSTUMT00006670001	Q86Z98|KINH_GIBMO	kinesin heavy chain [Ajellomyces capsulatus NAm1]	0.95	1	1	1	1
GSTUMT00000606001	P35580|MYH10_HUMAN	Viral A-type inclusion protein repeat protein [Neosartorya fischeri NRRL 181]	0.94	1	1	1	1
GSTUMT00010384001	Q5A950|ATG26_CANAL	hypothetical protein CIMG_03067 [Coccidioides immitis RS]	0.94	1	1	1	1

Accession	UNIPROT	Description	ΣCoverage	Σ# Proteins	Σ# Unique Peptides	Σ# Peptides	Σ# PSMs
GSTUMT00009188001	O59741|YN25_SCHPO	hypothetical protein An16g04000 [Aspergillus niger]	0.92	1	1	1	1
GSTUMT00010823001	P16157|ANK1_HUMAN	predicted protein [Ajellomyces capsulatus NAm1]	0.9	1	1	1	1
GSTUMT00007233001	Q8BH16|FBXL2_MOUSE	hypothetical protein BC1G_01623 [Botryotinia fuckeliana B05.10]	0.88	1	1	1	1
GSTUMT00011841001	Q9S775|PKL_ARATH	conserved hypothetical protein [Aspergillus terreus NIH2624]	0.88	1	1	1	1
GSTUMT00007415001	No hit found	unnamed protein product [Aspergillus oryzae]	0.87	1	1	1	1
GSTUMT00012096001	O74760|EIF3A_SCHPO	eukaryotic translation initiation factor 3 subunit EifCa, putative [Aspergillus fumigatus Af293]	0.84	1	1	1	3

Accession	UNIPROT	Description	ΣCoverage	Σ# Proteins	Σ# Unique Peptides	Σ# Peptides	Σ# PSMs
GSTUMT00004436001	O14164|EIF3C_SCHPO	hypothetical protein BC1G_06212 [Botryotinia fuckeliana B05.10]	0.82	1	1	1	2
GSTUMT00011945001	Q09697|RGA8_SCHPO	hypothetical protein CHGG_02438 [Chaetomium globosum CBS 148.51]	0.82	1	1	1	1
GSTUMT00005806001	No hit found	hypothetical protein SNOG_03931 [Phaeosphaeria nodorum SN15]	0.81	1	1	1	1
GSTUMT00006632001	Q5T9A4|ATD3B_HUMAN	predicted protein [Ajellomyces capsulatus NAm1]	0.81	1	1	1	1
GSTUMT00002391001	Q9URU2|DNA2_SCHPO	hypothetical protein BC1G_14724 [Botryotinia fuckeliana B05.10]	0.8	1	1	1	1
GSTUMT00003873001	Q01317|NUC2_NEUCR	hypothetical protein SS1G_10215 [Sclerotinia sclerotiorum 1980]	0.8	1	1	1	1

Accession	UNIPROT	Description	ΣCoverage	Σ# Proteins	Σ# Unique Peptides	Σ# Peptides	Σ# PSMs
GSTUMT00004929001	Q7TNH6|NPHP3_MOUSE	hypothetical protein CHGG_04534 [Chaetomium globosum CBS 148.51]	0.79	1	1	1	1
GSTUMT00008270001	P30622|CLIP1_HUMAN	hypothetical protein FG00647.1 [Gibberella zeae PH-1]	0.77	1	1	1	1
GSTUMT00003607001	P12270|TPR_HUMAN	hypothetical protein SS1G_11653 [Sclerotinia sclerotiorum 1980]	0.76	1	2	2	2
GSTUMT00002950001	Q12324|YVC1_YEAST	cation channel family transporter, putative [Aspergillus clavatus NRRL 1]	0.75	1	1	1	1
GSTUMT00005645001	Q97AI2|HELS_THEVO	hypothetical protein NCU07411 [Neurospora crassa OR74A]	0.75	1	1	1	1
GSTUMT00004442001	Q9H2U1|DHX36_HUMAN	rCG44442 [Rattus norvegicus]	0.73	1	1	1	1

Accession	UNIPROT	Description	ΣCoverage	Σ# Proteins	Σ# Unique Peptides	Σ# Peptides	Σ# PSMs
GSTUMT00004800001	No hit found	No hit found	0.72	1	1	1	1
GSTUMT00000478001	Q7PHR1|KIF1A_ANOGA	unnamed protein product [Aspergillus oryzae]	0.72	1	1	1	1
GSTUMT00004203001	P22637|CHOD_BREST	hypothetical protein BC1G_10725 [Botryotinia fuckeliana B05.10]	0.71	1	1	1	1
GSTUMT00010317001	P16157|ANK1_HUMAN	Ankyrin repeat protein [Aspergillus fumigatus A1163]	0.71	1	1	1	1
GSTUMT00003660001	Q01112|CDC42_SCHPO	small GTPase CDC42 [Tuber borchii]	0.71	1	1	1	1
GSTUMT00003361001	Q9UTT1|UBP21_SCHPO	hypothetical protein ATEG_08092 [Aspergillus terreus NIH2624]	0.7	1	1	1	1

Accession	UNIPROT	Description	ΣCoverage	Σ# Proteins	Σ# Unique Peptides	Σ# Peptides	Σ# PSMs
GSTUMT00004542001	P58342|ATCU2_RHIME	hypothetical protein SS1G_02457 [Sclerotinia sclerotiorum 1980]	0.7	1	1	1	1
GSTUMT00004714001	O13686|YDY5_SCHPO	conserved hypothetical protein [Aspergillus terreus NIH2624]	0.69	1	1	1	1
GSTUMT00000552001	No hit found	No hit found	0.68	1	1	1	2
GSTUMT00006726001	O14064|BIR1_SCHPO	hypothetical protein An01g11910 [Aspergillus niger]	0.68	1	1	1	1
GSTUMT00010538001	P50998|AMPD_SCHPO	hypothetical protein BC1G_14173 [Botryotinia fuckeliana B05.10]	0.68	1	1	1	1
GSTUMT00000473001	No hit found	hypothetical protein An06g01110 [Aspergillus niger]	0.68	1	1	1	1

Accession	UNIPROT	Description	ΣCoverage	Σ# Proteins	Σ# Unique Peptides	Σ# Peptides	Σ# PSMs
GSTUMT00011756001	P41697|BUD6_YEAST	hypothetical protein AN1324.2 [Aspergillus nidulans FGSC A4]	0.68	1	1	1	1
GSTUMT00004025001	P04821|CDC25_YEAST	hypothetical protein MGG_00199 [Magnaporthe grisea 70-15]	0.68	1	1	1	1
GSTUMT00008991001	P22189|ATC3_SCHPO	potassium/sodium P-type ATPase, putative [Aspergillus clavatus NRRL 1]	0.65	1	1	1	2
GSTUMT00009928001	Q10251|IF2P_SCHPO	hypothetical protein HCAG_06015 [Ajellomyces capsulatus NAm1]	0.65	1	1	1	1
GSTUMT00011729001	P40383|XRN1_SCHPO	hypothetical protein CIMG_06514 [Coccidioides immitis RS]	0.64	1	1	1	1
GSTUMT00006678001	Q6CY10|LTE1_KLULA	hypothetical protein SS1G_12228 [Sclerotinia sclerotiorum 1980]	0.63	1	1	1	1

Accession	UNIPROT	Description	ΣCoverage	Σ# Proteins	Σ# Unique Peptides	Σ# Peptides	Σ# PSMs
GSTUMT00005574001	Q6C2R8|RAD5_YARLI	hypothetical protein CNBK2340 [Cryptococcus neoformans var. neoformans B-3501A]	0.61	1	1	1	1
GSTUMT00004673001	Q8X0X6|SPT16_NEUCR	hypothetical protein SS1G_04853 [Sclerotinia sclerotiorum 1980]	0.59	1	1	1	1
GSTUMT00011076001	No hit found	predicted protein [Laccaria bicolor S238N-H82]	0.59	1	1	1	1
GSTUMT00005899001	Q7S9L2|STU1_NEUCR	hypothetical protein MGG_04185 [Magnaporthe grisea 70-15]	0.59	1	1	1	1
GSTUMT00002146001	Q03868|SPO71_YEAST	hypothetical protein CIMG_00092 [Coccidioides immitis RS]	0.54	1	1	1	1
GSTUMT00008484001	P16157|ANK1_HUMAN	ankyrin repeat protein, putative [Trichomonas vaginalis G3]	0.53	1	1	1	1

Accession	UNIPROT	Description	ΣCoverage	Σ# Proteins	Σ# Unique Peptides	Σ# Peptides	Σ# PSMs
GSTUMT00009648001	Q9P7W4|POF10_SCHPO	hypothetical protein AN0557.2 [Aspergillus nidulans FGSC A4]	0.52	1	1	1	2
GSTUMT00004523001	Q4WYF1|HIR3_ASPFU	transcriptional corepressor of histone genes (Hir3), putative [Neosartorya fischeri NRRL 181]	0.52	1	1	1	3
GSTUMT00006345001	Q5UQX5|YR883_MIMIV	ankyrin repeat protein [Neosartorya fischeri NRRL 181]	0.5	1	1	1	1
GSTUMT00012126001	No hit found	predicted protein [Ajellomyces capsulatus NAm1]	0.5	1	1	1	1
GSTUMT00011240001	P13433|RPOM_YEAST	unnamed protein product [Kluyveromyces lactis]	0.49	1	1	1	1
GSTUMT00008590001	Q1DX43|SEC31_COCIM	hypothetical protein CIMG_05120 [Coccidioides immitis RS]	0.49	1	1	1	1

Accession	UNIPROT	Description	ΣCoverage	Σ# Proteins	Σ# Unique Peptides	Σ# Peptides	Σ# PSMs
GSTUMT00010668001	Q9UT24|BRR2_SCHPO	hypothetical protein ATEG_00094 [Aspergillus terreus NIH2624]	0.47	1	1	1	1
GSTUMT00005225001	P38735|YHD5_YEAST	ACL072Cp [Ashbya gossypii ATCC 10895]	0.45	1	1	1	1
GSTUMT00001634001	Q9UTJ8|RAD50_SCHPO	DNA repair protein Rad50 [Neosartorya fischeri NRRL 181]	0.45	1	1	1	1
GSTUMT00000794001	P45443|DYHC_NEUCR	dynein heavy chain [Chaetomium globosum CBS 148.51]	0.43	1	2	2	2
GSTUMT00005369001	Q10161|CLH_SCHPO	clathrin heavy chain [Aspergillus fumigatus Af293]	0.37	1	1	1	1
GSTUMT00003624001	Q07878|VPS13_YEAST	hypothetical protein SS1G_05729 [Sclerotinia sclerotiorum 1980]	0.32	1	1	1	1

Accession	UNIPROT	Description	ΣCoverage	Σ# Proteins	Σ# Unique Peptides	Σ# Peptides	Σ# PSMs
GSTUMT00007495001	Q4WXH8|DPOE_ASPFU	hypothetical protein CIMG_01894 [Coccidioides immitis RS]	0.32	1	1	1	1
GSTUMT00002496001	O43103|SID2_USTMA	hypothetical protein SNOG_02134 [Phaeosphaeria nodorum SN15]	0.16	1	1	1	1

	A10				B10				C10				D10			
Accession	Score A4	Coverage A4	# Peptides A4	# PSM A4	Score B4	Coverage B4	# Peptides B4	# PSM B4	Score C4	Coverage C4	# Peptides C4	# PSM C4	Score D4	Coverage D4	# Peptides D4	# PSM D4
GSTUMT00001850001																
GSTUMT00010275001					50.09	11.22	1	2	35.24	11.22	1	1	0	11.22	1	3
GSTUMT00006386001																
GSTUMT00011582001																
GSTUMT00004570001																
GSTUMT00010038001					0	23.26	1	1								

	A10				B10				C10				D10			
Accession	Score A4	Coverage A4	# Peptides A4	# PSM A4	Score B4	Coverage B4	# Peptides B4	# PSM B4	Score C4	Coverage C4	# Peptides C4	# PSM C4	Score D4	Coverage D4	# Peptides D4	# PSM D4
GSTUMT00000409001	0	11.65	1	1					42.87	21.36	2	2		0		
GSTUMT00006163001	51.62	5.42	1	2	38.19	16.57	3	10	78.89	10.24	2	7	62.89	10.24	2	5
GSTUMT00006162001	0	16.39	1	1									64.97	16.39	1	2
GSTUMT00003270001					0	16.13	1	1								
GSTUMT00010457001																
GSTUMT00000720001																

	A10				B10				C10				D10			
Accession	Score A4	Coverage A4	# Peptides A4	# PSM A4	Score B4	Coverage B4	# Peptides B4	# PSM B4	Score C4	Coverage C4	# Peptides C4	# PSM C4	Score D4	Coverage D4	# Peptides D4	# PSM D4
GSTUMT00008041001																
GSTUMT00010465001																
GSTUMT00000288001					0	4.26	2	2	45.06	1.42	1	1				
GSTUMT00008579001																
GSTUMT00003593001																
GSTUMT00001163001					0	9.47	1	1								

	A10				B10				C10				D10			
Accession	Score A4	Coverage A4	# Peptides A4	# PSM A4	Score B4	Coverage B4	# Peptides B4	# PSM B4	Score C4	Coverage C4	# Peptides C4	# PSM C4	Score D4	Coverage D4	# Peptides D4	# PSM D4
GSTUMT00001328001																
GSTUMT00007325001					0	8.33	1	1	41.54	8.33	1	1	0	8.33	1	1
GSTUMT00005607001																
GSTUMT00007614001																
GSTUMT00008389001																
GSTUMT00007211001																

	A10				B10				C10				D10			
Accession	Score A4	Coverage A4	# Peptides A4	# PSM A4	Score B4	Coverage B4	# Peptides B4	# PSM B4	Score C4	Coverage C4	# Peptides C4	# PSM C4	Score D4	Coverage D4	# Peptides D4	# PSM D4
GSTUMT00003125001																
GSTUMT00011952001																
GSTUMT00003402001	0	6.57	1	1												
GSTUMT00007957001																
GSTUMT00003753001	0	6.02	1	1												
GSTUMT00004943001																

	A10				B10				C10				D10			
Accession	Score A4	Coverage A4	# Peptides A4	# PSM A4	Score B4	Coverage B4	# Peptides B4	# PSM B4	Score C4	Coverage C4	# Peptides C4	# PSM C4	Score D4	Coverage D4	# Peptides D4	# PSM D4
GSTUMT00003005001	0	5.76	1	1												
GSTUMT00001214001	0	5.69	1	1												
GSTUMT00000021001									0	2.33	1	1	46.64	3.21	1	1
GSTUMT00010075001																
GSTUMT00002474001	0	2.29	1	1									0	2.29	1	1
GSTUMT00009481001																

	A10				B10				C10				D10			
Accession	Score A4	Coverage A4	# Peptides A4	# PSM A4	Score B4	Coverage B4	# Peptides B4	# PSM B4	Score C4	Coverage C4	# Peptides C4	# PSM C4	Score D4	Coverage D4	# Peptides D4	# PSM D4
GSTUMT00011335001									0	5.17	1	1				
GSTUMT00005695001																
GSTUMT00001678001																
GSTUMT00005706001																
GSTUMT00006122001	0	4.76	1	1												
GSTUMT00008018001									0	4.61	1	1				

	A10				B10				C10				D10			
Accession	Score A4	Coverage A4	# Peptides A4	# PSM A4	Score B4	Coverage B4	# Peptides B4	# PSM B4	Score C4	Coverage C4	# Peptides C4	# PSM C4	Score D4	Coverage D4	# Peptides D4	# PSM D4
GSTUMT00003527001					0	4.58	1	1								
GSTUMT00002908001																
GSTUMT00006299001	0	4.14	1	1												
GSTUMT00005677001									0	4.13	1	1	0	4.13	1	1
GSTUMT00007883001																
GSTUMT00010477001													0	2.44	1	1

	A10				B10				C10				D10			
Accession	Score A4	Coverage A4	# Peptides A4	# PSM A4	Score B4	Coverage B4	# Peptides B4	# PSM B4	Score C4	Coverage C4	# Peptides C4	# PSM C4	Score D4	Coverage D4	# Peptides D4	# PSM D4
GSTUMT00001231001																
GSTUMT00002258001																
GSTUMT00009943001	0	4.01	1	1												
GSTUMT00009267001					0	3.89	1	1								
GSTUMT00008418001																
GSTUMT00008015001																

	A10				B10				C10				D10			
Accession	Score A4	Coverage A4	# Peptides A4	# PSM A4	Score B4	Coverage B4	# Peptides B4	# PSM B4	Score C4	Coverage C4	# Peptides C4	# PSM C4	Score D4	Coverage D4	# Peptides D4	# PSM D4
GSTUMT00001583001																
GSTUMT00008511001	0	3.54	1	1												
GSTUMT00012196001	0	3.47	1	1												
GSTUMT00001470001																
GSTUMT00003199001																
GSTUMT00003201001																

	A10				B10				C10				D10			
Accession	Score A4	Coverage A4	# Peptides A4	# PSM A4	Score B4	Coverage B4	# Peptides B4	# PSM B4	Score C4	Coverage C4	# Peptides C4	# PSM C4	Score D4	Coverage D4	# Peptides D4	# PSM D4
GSTUMT00001220001																
GSTUMT00000125001																
GSTUMT00011316001																
GSTUMT00002085001																
GSTUMT00004210001													0	3.18	1	1
GSTUMT00002243001																

	A10				B10				C10				D10			
Accession	Score A4	Coverage A4	# Peptides A4	# PSM A4	Score B4	Coverage B4	# Peptides B4	# PSM B4	Score C4	Coverage C4	# Peptides C4	# PSM C4	Score D4	Coverage D4	# Peptides D4	# PSM D4
GSTUMT00011958001																
GSTUMT00011509001					0	3.03	1	1								
GSTUMT00006889001																
GSTUMT00001784001	0	3.01	1	1												
GSTUMT00008539001																
GSTUMT00009800001																

	A10				B10				C10				D10			
Accession	Score A4	Coverage A4	# Peptides A4	# PSM A4	Score B4	Coverage B4	# Peptides B4	# PSM B4	Score C4	Coverage C4	# Peptides C4	# PSM C4	Score D4	Coverage D4	# Peptides D4	# PSM D4
GSTUMT00001821001					0	2.97	1	1								
GSTUMT00010624001																
GSTUMT00008548001																
GSTUMT00002670001					0	2.84	1	1								
GSTUMT00009269001	0	2.79	1	1	0	2.79	1	1	0	2.79	1	1	0	2.79	1	1
GSTUMT00009669001	0	2.75	2	2												

	A10				B10				C10				D10			
Accession	Score A4	Coverage A4	# Peptides A4	# PSM A4	Score B4	Coverage B4	# Peptides B4	# PSM B4	Score C4	Coverage C4	# Peptides C4	# PSM C4	Score D4	Coverage D4	# Peptides D4	# PSM D4
GSTUMT00002014001																
GSTUMT00010863001																
GSTUMT00001207001									0	2.62	1	1				
GSTUMT00011332001																
GSTUMT00011943001													0	2.55	1	1
GSTUMT00006007001																

	A10				B10				C10				D10			
Accession	Score A4	Coverage A4	# Peptides A4	# PSM A4	Score B4	Coverage B4	# Peptides B4	# PSM B4	Score C4	Coverage C4	# Peptides C4	# PSM C4	Score D4	Coverage D4	# Peptides D4	# PSM D4
GSTUMT00006874001																
GSTUMT00011675001																
GSTUMT00006255001																
GSTUMT00005542001	0	2.39	1	1												
GSTUMT00012817001	0	2.35	1	1												
GSTUMT00010261001																

	A10				B10				C10				D10			
Accession	Score A4	Coverage A4	# Peptides A4	# PSM A4	Score B4	Coverage B4	# Peptides B4	# PSM B4	Score C4	Coverage C4	# Peptides C4	# PSM C4	Score D4	Coverage D4	# Peptides D4	# PSM D4
GSTUMT00006412001																
GSTUMT00000874001	0	2.31	1	1												
GSTUMT00007262001					0	2.31	1	1								
GSTUMT00010001001									0	2.3	1	1				
GSTUMT00003768001													0	2.3	1	1
GSTUMT00007761001	0	2.27	1	1												

	A10				B10				C10				D10			
Accession	Score A4	Coverage A4	# Peptides A4	# PSM A4	Score B4	Coverage B4	# Peptides B4	# PSM B4	Score C4	Coverage C4	# Peptides C4	# PSM C4	Score D4	Coverage D4	# Peptides D4	# PSM D4
GSTUMT00001334001																
GSTUMT00011748001																
GSTUMT00011209001					0	2.23	1	1								
GSTUMT00011415001																
GSTUMT00008736001					0	2.14	1	1								
GSTUMT00006281001																

	A10				B10				C10				D10			
Accession	Score A4	Coverage A4	# Peptides A4	# PSM A4	Score B4	Coverage B4	# Peptides B4	# PSM B4	Score C4	Coverage C4	# Peptides C4	# PSM C4	Score D4	Coverage D4	# Peptides D4	# PSM D4
GSTUMT00002599001	0	2.12	1	1												
GSTUMT00000583001																
GSTUMT00012700001																
GSTUMT00007685001					0	2.05	1	1								
GSTUMT00012729001																
GSTUMT00010108001																

	A10				B10				C10				D10			
Accession	Score A4	Coverage A4	# Peptides A4	# PSM A4	Score B4	Coverage B4	# Peptides B4	# PSM B4	Score C4	Coverage C4	# Peptides C4	# PSM C4	Score D4	Coverage D4	# Peptides D4	# PSM D4
GSTUMT00003152001									0	0.89	1	1				
GSTUMT00008198001																
GSTUMT00004165001																
GSTUMT00007887001	0	1.85	1	1												
GSTUMT00001812001									0	1.85	1	1				
GSTUMT00004787001																

	A10				B10				C10				D10			
Accession	Score A4	Coverage A4	# Peptides A4	# PSM A4	Score B4	Coverage B4	# Peptides B4	# PSM B4	Score C4	Coverage C4	# Peptides C4	# PSM C4	Score D4	Coverage D4	# Peptides D4	# PSM D4
GSTUMT00002932001					0	0.78	1	1								
GSTUMT00009140001																
GSTUMT00004461001																
GSTUMT00009834001																
GSTUMT00010450001																
GSTUMT00009374001	0	1.75	1	1												

	A10				B10				C10				D10			
Accession	Score A4	Coverage A4	# Peptides A4	# PSM A4	Score B4	Coverage B4	# Peptides B4	# PSM B4	Score C4	Coverage C4	# Peptides C4	# PSM C4	Score D4	Coverage D4	# Peptides D4	# PSM D4
GSTUMT00011323001																
GSTUMT00005017001																
GSTUMT00008292001					0	1.73	1	1								
GSTUMT00004969001													0	1.73	1	1
GSTUMT00003080001									0	1.71	1	1				
GSTUMT00005768001																

	A10				B10				C10				D10			
Accession	Score A4	Coverage A4	# Peptides A4	# PSM A4	Score B4	Coverage B4	# Peptides B4	# PSM B4	Score C4	Coverage C4	# Peptides C4	# PSM C4	Score D4	Coverage D4	# Peptides D4	# PSM D4
GSTUMT00003525001					0	1.69	1	1								
GSTUMT00010278001																
GSTUMT00000105001					0	1.68	1	1								
GSTUMT00002056001																
GSTUMT00000660001													0	1.65	1	1
GSTUMT00004057001	0	1.63	1	1												

	A10				B10				C10				D10			
Accession	Score A4	Coverage A4	# Peptides A4	# PSM A4	Score B4	Coverage B4	# Peptides B4	# PSM B4	Score C4	Coverage C4	# Peptides C4	# PSM C4	Score D4	Coverage D4	# Peptides D4	# PSM D4
GSTUMT00009838001		0														
GSTUMT00000277001	0	1.61	1	1												
GSTUMT00006911001																
GSTUMT00007159001																
GSTUMT00012580001					0	1.57	1	1								
GSTUMT00012371001																

	A10				B10				C10				D10			
Accession	Score A4	Coverage A4	# Peptides A4	# PSM A4	Score B4	Coverage B4	# Peptides B4	# PSM B4	Score C4	Coverage C4	# Peptides C4	# PSM C4	Score D4	Coverage D4	# Peptides D4	# PSM D4
GSTUMT00004219001																
GSTUMT00005928001																
GSTUMT00002440001																
GSTUMT00000106001																
GSTUMT00009341001																
GSTUMT00003847001																

	A10				B10				C10				D10			
Accession	Score A4	Coverage A4	# Peptides A4	# PSM A4	Score B4	Coverage B4	# Peptides B4	# PSM B4	Score C4	Coverage C4	# Peptides C4	# PSM C4	Score D4	Coverage D4	# Peptides D4	# PSM D4
GSTUMT00001078001	0	1.5	1	1												
GSTUMT00011312001																
GSTUMT00001275001													0	1.45	1	1
GSTUMT00004257001																
GSTUMT00011007001																
GSTUMT00008074001																

	A10				B10				C10				D10			
Accession	Score A4	Coverage A4	# Peptides A4	# PSM A4	Score B4	Coverage B4	# Peptides B4	# PSM B4	Score C4	Coverage C4	# Peptides C4	# PSM C4	Score D4	Coverage D4	# Peptides D4	# PSM D4
GSTUMT00001308001					0	1.39	1	1								
GSTUMT00008696001																
GSTUMT00003182001																
GSTUMT00004647001																
GSTUMT00004452001	0	1.32	1	1		0										
GSTUMT00007016001	0	1.32	1	1												

	A10				B10				C10				D10			
Accession	Score A4	Coverage A4	# Peptides A4	# PSM A4	Score B4	Coverage B4	# Peptides B4	# PSM B4	Score C4	Coverage C4	# Peptides C4	# PSM C4	Score D4	Coverage D4	# Peptides D4	# PSM D4
GSTUMT00009675001	0	1.32	1	1												
GSTUMT00010640001																
GSTUMT00002283001																
GSTUMT00007918001					0	1.3	1	1								
GSTUMT00001196001					0	1.3	1	1								
GSTUMT00010240001																

	A10				B10				C10				D10			
Accession	Score A4	Coverage A4	# Peptides A4	# PSM A4	Score B4	Coverage B4	# Peptides B4	# PSM B4	Score C4	Coverage C4	# Peptides C4	# PSM C4	Score D4	Coverage D4	# Peptides D4	# PSM D4
GSTUMT00003604001																
GSTUMT00005329001	0	1.27	1	1												
GSTUMT00001651001					0	1.27	1	1								
GSTUMT00006989001																
GSTUMT00008547001																
GSTUMT00009359001	0	1.24	1	1												

	A10				B10				C10				D10			
Accession	Score A4	Coverage A4	# Peptides A4	# PSM A4	Score B4	Coverage B4	# Peptides B4	# PSM B4	Score C4	Coverage C4	# Peptides C4	# PSM C4	Score D4	Coverage D4	# Peptides D4	# PSM D4
GSTUMT00008029001									0	1.24	1	1				
GSTUMT00004213001																
GSTUMT00006967001																
GSTUMT00005973001					0	1.23	1	1								
GSTUMT00011138001													0	1.23	1	1
GSTUMT00000199001																

	A10				B10				C10				D10			
Accession	Score A4	Coverage A4	# Peptides A4	# PSM A4	Score B4	Coverage B4	# Peptides B4	# PSM B4	Score C4	Coverage C4	# Peptides C4	# PSM C4	Score D4	Coverage D4	# Peptides D4	# PSM D4
GSTUMT00006203001																
GSTUMT00007732001	0	1.22	1	1												
GSTUMT00008870001						0										
GSTUMT00004983001																
GSTUMT00000086001																
GSTUMT00010828001									0	1.18	1	1				

	A10				B10				C10				D10			
Accession	Score A4	Coverage A4	# Peptides A4	# PSM A4	Score B4	Coverage B4	# Peptides B4	# PSM B4	Score C4	Coverage C4	# Peptides C4	# PSM C4	Score D4	Coverage D4	# Peptides D4	# PSM D4
GSTUMT00005488001																
GSTUMT00010343001													0	1.15	1	1
GSTUMT00011768001	0	1.14	1	1												
GSTUMT00005941001									0	1.14	1	1				
GSTUMT00003996001					0	1.13	1	1								
GSTUMT00001258001																

	A10				B10				C10				D10			
Accession	Score A4	Coverage A4	# Peptides A4	# PSM A4	Score B4	Coverage B4	# Peptides B4	# PSM B4	Score C4	Coverage C4	# Peptides C4	# PSM C4	Score D4	Coverage D4	# Peptides D4	# PSM D4
GSTUMT00000754001									0	1.12	1	1				
GSTUMT00010843001																
GSTUMT00010787001																
GSTUMT00000550001									0	1.1	1	1				
GSTUMT00000204001																
GSTUMT00006380001	0	1.07	1	1												

	A10				B10				C10				D10			
Accession	Score A4	Coverage A4	# Peptides A4	# PSM A4	Score B4	Coverage B4	# Peptides B4	# PSM B4	Score C4	Coverage C4	# Peptides C4	# PSM C4	Score D4	Coverage D4	# Peptides D4	# PSM D4
GSTUMT00006298001					0	1.06	1	1								
GSTUMT00012201001																
GSTUMT00009286001																
GSTUMT00012289001																
GSTUMT00002124001																
GSTUMT00010874001	0	1.03	1	1												

	A10				B10				C10				D10			
Accession	Score A4	Coverage A4	# Peptides A4	# PSM A4	Score B4	Coverage B4	# Peptides B4	# PSM B4	Score C4	Coverage C4	# Peptides C4	# PSM C4	Score D4	Coverage D4	# Peptides D4	# PSM D4
GSTUMT00001431001																
GSTUMT00005156001	0	1.01	1	1												
GSTUMT00000895001																
GSTUMT00011643001																
GSTUMT00008433001																
GSTUMT00010895001																

	A10				B10				C10				D10			
Accession	Score A4	Coverage A4	# Peptides A4	# PSM A4	Score B4	Coverage B4	# Peptides B4	# PSM B4	Score C4	Coverage C4	# Peptides C4	# PSM C4	Score D4	Coverage D4	# Peptides D4	# PSM D4
GSTUMT00011664001																
GSTUMT00009187001	0	0.95	1	1												
GSTUMT00006670001																
GSTUMT00000606001																
GSTUMT00010384001																
GSTUMT00009188001														0		

	A10				B10				C10				D10			
Accession	Score A4	Coverage A4	# Peptides A4	# PSM A4	Score B4	Coverage B4	# Peptides B4	# PSM B4	Score C4	Coverage C4	# Peptides C4	# PSM C4	Score D4	Coverage D4	# Peptides D4	# PSM D4
GSTUMT00010823001																
GSTUMT00007233001													0	0.88	1	1
GSTUMT00011841001																
GSTUMT00007415001																
GSTUMT00012096001					0	0.84	1	2								
GSTUMT00004436001	46.79	0.82	1	1												

	A10				B10				C10				D10			
Accession	Score A4	Coverage A4	# Peptides A4	# PSM A4	Score B4	Coverage B4	# Peptides B4	# PSM B4	Score C4	Coverage C4	# Peptides C4	# PSM C4	Score D4	Coverage D4	# Peptides D4	# PSM D4
GSTUMT00011945001													0	0.82	1	1
GSTUMT00005806001					0	0.81	1	1								
GSTUMT00006632001													0	0.81	1	1
GSTUMT00002391001					0	0.8	1	1								
GSTUMT00003873001																
GSTUMT00004929001					0	0.79	1	1								

	A10				B10				C10				D10			
Accession	Score A4	Coverage A4	# Peptides A4	# PSM A4	Score B4	Coverage B4	# Peptides B4	# PSM B4	Score C4	Coverage C4	# Peptides C4	# PSM C4	Score D4	Coverage D4	# Peptides D4	# PSM D4
GSTUMT00008270001																
GSTUMT00003607001		0			0	0.31	1	1								
GSTUMT00002950001													0	0.75	1	1
GSTUMT00005645001																
GSTUMT00004442001													0	0.73	1	1
GSTUMT00004800001	0	0.72	1	1												

	A10				B10				C10				D10			
Accession	Score A4	Coverage A4	# Peptides A4	# PSM A4	Score B4	Coverage B4	# Peptides B4	# PSM B4	Score C4	Coverage C4	# Peptides C4	# PSM C4	Score D4	Coverage D4	# Peptides D4	# PSM D4
GSTUMT00000478001																
GSTUMT00004203001									0	0.71	1	1				
GSTUMT00010317001																
GSTUMT00003660001																
GSTUMT00003361001									24.81	0.7	1	1				
GSTUMT00004542001																

	A10				B10				C10				D10			
Accession	Score A4	Coverage A4	# Peptides A4	# PSM A4	Score B4	Coverage B4	# Peptides B4	# PSM B4	Score C4	Coverage C4	# Peptides C4	# PSM C4	Score D4	Coverage D4	# Peptides D4	# PSM D4
GSTUMT00004714001																
GSTUMT00000552001	0	0.68	1	1												
GSTUMT00006726001					0	0.68	1	1								
GSTUMT00010538001													0	0.68	1	1
GSTUMT00000473001																
GSTUMT00011756001																

	A10				B10				C10				D10			
Accession	Score A4	Coverage A4	# Peptides A4	# PSM A4	Score B4	Coverage B4	# Peptides B4	# PSM B4	Score C4	Coverage C4	# Peptides C4	# PSM C4	Score D4	Coverage D4	# Peptides D4	# PSM D4
GSTUMT00004025001																
GSTUMT00008991001																
GSTUMT00009928001																
GSTUMT00011729001					0	0.64	1	1								
GSTUMT00006678001									0	0.63	1	1				
GSTUMT00005574001	0	0.61	1	1												

	A10				B10				C10				D10			
Accession	Score A4	Coverage A4	# Peptides A4	# PSM A4	Score B4	Coverage B4	# Peptides B4	# PSM B4	Score C4	Coverage C4	# Peptides C4	# PSM C4	Score D4	Coverage D4	# Peptides D4	# PSM D4
GSTUMT00004673001																
GSTUMT00011076001																
GSTUMT00005899001																
GSTUMT00002146001	0	0.54	1	1												
GSTUMT00008484001	0	0.53	1	1												
GSTUMT00009648001	0	0.52	1	1												

	A10				B10				C10				D10			
Accession	Score A4	Coverage A4	# Peptides A4	# PSM A4	Score B4	Coverage B4	# Peptides B4	# PSM B4	Score C4	Coverage C4	# Peptides C4	# PSM C4	Score D4	Coverage D4	# Peptides D4	# PSM D4
GSTUMT00004523001	0	0.52	1	1									0	0.52	1	1
GSTUMT00006345001						0				0				0		
GSTUMT00012126001																
GSTUMT00011240001					0	0.49	1	1								
GSTUMT00008590001																
GSTUMT00010668001													0	0.47	1	1

	A10				B10				C10				D10			
Accession	Score A4	Coverage A4	# Peptides A4	# PSM A4	Score B4	Coverage B4	# Peptides B4	# PSM B4	Score C4	Coverage C4	# Peptides C4	# PSM C4	Score D4	Coverage D4	# Peptides D4	# PSM D4
GSTUMT00005225001					0	0.45	1	1								
GSTUMT00001634001																
GSTUMT00000794001		0			0	0.21	1	1					0	0.21	1	1
GSTUMT00005369001	0	0.37	1	1												
GSTUMT00003624001	0	0.32	1	1												
GSTUMT00007495001																

	A10				B10				C10				D10			
Accession	Score A4	Coverage A4	# Peptides A4	# PSM A4	Score B4	Coverage B4	# Peptides B4	# PSM B4	Score C4	Coverage C4	# Peptides C4	# PSM C4	Score D4	Coverage D4	# Peptides D4	# PSM D4
GSTUMT00002496001																

	A1I				B1I				C1I				D1I			
Accession	Score E4	Coverage E4	# Peptides E4	# PSM E4	Score F4	Coverage F4	# Peptides F4	# PSM F4	Score G4	Coverage G4	# Peptides G4	# PSM G4	Score H4	Coverage H4	# Peptides H4	# PSM H4
GSTUMT00001850001													199.67	37.04	3	5
GSTUMT00010275001					82.31	22.45	3	3	46.03	20.41	2	4	29.33	20.41	2	3
GSTUMT00006386001	0	28.21	1	1												
GSTUMT00011582001					33.22	24.92	2	2								
GSTUMT00004570001	63.63	18.68	2	4	0	18.68	2	3	53.57	24.18	3	5	65.63	24.18	3	7
GSTUMT00010038001																

	A1I				B1I				C1I				D1I			
Accession	Score E4	Coverage E4	# Peptides E4	# PSM E4	Score F4	Coverage F4	# Peptides F4	# PSM F4	Score G4	Coverage G4	# Peptides G4	# PSM G4	Score H4	Coverage H4	# Peptides H4	# PSM H4
GSTUMT00000409001																
GSTUMT00006163001	32.68	10.24	2	3	62.32	10.24	2	4	0	10.24	2	5	29.75	10.24	2	5
GSTUMT00006162001	70.67	16.39	1	1	53.28	16.39	1	1					35.48	16.39	1	1
GSTUMT00003270001																
GSTUMT00010457001	0	14.29	1	2	96.6	14.29	1	1	50.37	14.29	1	2	0	14.29	1	1
GSTUMT00000720001	0	12.31	1	1												

	A1I				B1I				C1I				D1I			
Accession	Score E4	Coverage E4	# Peptides E4	# PSM E4	Score F4	Coverage F4	# Peptides F4	# PSM F4	Score G4	Coverage G4	# Peptides G4	# PSM G4	Score H4	Coverage H4	# Peptides H4	# PSM H4
GSTUMT00008041001	47.76	11.74	1	2												
GSTUMT00010465001	190	11.22	1	3												
GSTUMT00000288001					81.23	9.57	4	6	0	2.84	1	1				
GSTUMT00008579001									0	9.71	1	1				
GSTUMT00003593001	76.97	9.5	1	2												
GSTUMT00001163001																

	A1I				B1I				C1I				D1I			
Accession	Score E4	Coverage E4	# Peptides E4	# PSM E4	Score F4	Coverage F4	# Peptides F4	# PSM F4	Score G4	Coverage G4	# Peptides G4	# PSM G4	Score H4	Coverage H4	# Peptides H4	# PSM H4
GSTUMT00001328001									0	8.79	1	1				
GSTUMT00007325001					0	8.33	1	1					0	8.33	1	1
GSTUMT00005607001					0	7.77	1	1								
GSTUMT00007614001													0	7.44	1	1
GSTUMT00008389001	40.91	7.35	2	4												
GSTUMT00007211001	0	7.14	1	1												

	A1I				B1I				C1I				D1I			
Accession	Score E4	Coverage E4	# Peptides E4	# PSM E4	Score F4	Coverage F4	# Peptides F4	# PSM F4	Score G4	Coverage G4	# Peptides G4	# PSM G4	Score H4	Coverage H4	# Peptides H4	# PSM H4
GSTUMT00003125001	0	7.11	2	2												
GSTUMT00011952001	41.18	6.71	1	1												
GSTUMT00003402001																
GSTUMT00007957001					0	6.08	1	1								
GSTUMT00003753001																
GSTUMT00004943001									0	5.83	1	2				

	A1I				B1I				C1I				D1I			
Accession	Score E4	Coverage E4	# Peptides E4	# PSM E4	Score F4	Coverage F4	# Peptides F4	# PSM F4	Score G4	Coverage G4	# Peptides G4	# PSM G4	Score H4	Coverage H4	# Peptides H4	# PSM H4
GSTUMT00003005001																
GSTUMT00001214001																
GSTUMT00000021001					0	3.21	1	1					0	5.54	2	2
GSTUMT00010075001					0	2.51	1	1	37.55	2.76	1	1	0	5.28	2	2
GSTUMT00002474001	71.51	5.21	2	2												
GSTUMT00009481001					39.19	5.19	1	2								

	A1I				B1I				C1I				D1I			
Accession	Score E4	Coverage E4	# Peptides E4	# PSM E4	Score F4	Coverage F4	# Peptides F4	# PSM F4	Score G4	Coverage G4	# Peptides G4	# PSM G4	Score H4	Coverage H4	# Peptides H4	# PSM H4
GSTUMT00011335001		0														
GSTUMT00005695001					0	5.13	1	1								
GSTUMT00001678001					0	5.09	1	1								
GSTUMT00005706001													0	5	1	1
GSTUMT00006122001																
GSTUMT00008018001																

	A1I				B1I				C1I				D1I			
Accession	Score E4	Coverage E4	# Peptides E4	# PSM E4	Score F4	Coverage F4	# Peptides F4	# PSM F4	Score G4	Coverage G4	# Peptides G4	# PSM G4	Score H4	Coverage H4	# Peptides H4	# PSM H4
GSTUMT00003527001																
GSTUMT00002908001									0	4.37	1	1				
GSTUMT00006299001																
GSTUMT00005677001					0	4.13	1	1								
GSTUMT00007883001					0	4.13	1	1								
GSTUMT00010477001					0	1.68	1	1					0	2.44	1	1

	A1I				B1I				C1I				D1I			
Accession	Score E4	Coverage E4	# Peptides E4	# PSM E4	Score F4	Coverage F4	# Peptides F4	# PSM F4	Score G4	Coverage G4	# Peptides G4	# PSM G4	Score H4	Coverage H4	# Peptides H4	# PSM H4
GSTUMT00001231001													0	4.11	1	1
GSTUMT00002258001					0	4.02	1	1								
GSTUMT00009943001																
GSTUMT00009267001																
GSTUMT00008418001													39.43	3.83	1	1
GSTUMT00008015001					0	3.68	1	1								

	A1I				B1I				C1I				D1I			
Accession	Score E4	Coverage E4	# Peptides E4	# PSM E4	Score F4	Coverage F4	# Peptides F4	# PSM F4	Score G4	Coverage G4	# Peptides G4	# PSM G4	Score H4	Coverage H4	# Peptides H4	# PSM H4
GSTUMT00001583001					0	3.64	1	1								
GSTUMT00008511001																
GSTUMT00012196001																
GSTUMT00001470001					0	3.45	1	1								
GSTUMT00003199001					0	3.45	1	1								
GSTUMT00003201001					0	3.4	1	1								

	A1I				B1I				C1I				D1I			
Accession	Score E4	Coverage E4	# Peptides E4	# PSM E4	Score F4	Coverage F4	# Peptides F4	# PSM F4	Score G4	Coverage G4	# Peptides G4	# PSM G4	Score H4	Coverage H4	# Peptides H4	# PSM H4
GSTUMT00001220001	0	3.32	1	1												
GSTUMT00000125001									0	3.29	1	1				
GSTUMT00011316001	0	3.21	1	1												
GSTUMT00002085001					0	3.2	1	1					0	3.2	1	1
GSTUMT00004210001																
GSTUMT00002243001													0	3.16	1	1

	A1I				B1I				C1I				D1I			
Accession	Score E4	Coverage E4	# Peptides E4	# PSM E4	Score F4	Coverage F4	# Peptides F4	# PSM F4	Score G4	Coverage G4	# Peptides G4	# PSM G4	Score H4	Coverage H4	# Peptides H4	# PSM H4
GSTUMT00011958001					43.7	3.04	1	1								
GSTUMT00011509001																
GSTUMT00006889001					0	3.02	1	1								
GSTUMT00001784001																
GSTUMT00008539001	41.34	3.01	2	3												
GSTUMT00009800001					41.63	3	1	1								

	A1I				B1I				C1I				D1I			
Accession	Score E4	Coverage E4	# Peptides E4	# PSM E4	Score F4	Coverage F4	# Peptides F4	# PSM F4	Score G4	Coverage G4	# Peptides G4	# PSM G4	Score H4	Coverage H4	# Peptides H4	# PSM H4
GSTUMT00001821001																
GSTUMT00010624001	0	2.95	1	1												
GSTUMT00008548001									0	2.94	1	1				
GSTUMT00002670001																
GSTUMT00009269001	0	2.79	1	1	0	2.79	1	1	0	2.79	1	1	0	2.79	1	1
GSTUMT00009669001	0	1.83	1	1												

	A1I				B1I				C1I				D1I			
Accession	Score E4	Coverage E4	# Peptides E4	# PSM E4	Score F4	Coverage F4	# Peptides F4	# PSM F4	Score G4	Coverage G4	# Peptides G4	# PSM G4	Score H4	Coverage H4	# Peptides H4	# PSM H4
GSTUMT00002014001													0	2.75	1	1
GSTUMT00010863001									38.59	2.63	1	2	0	2.63	1	1
GSTUMT00001207001																
GSTUMT00011332001					0	2.56	1	1								
GSTUMT00011943001																
GSTUMT00006007001	0	2.54	1	1												

	A1I				B1I				C1I				D1I			
Accession	Score E4	Coverage E4	# Peptides E4	# PSM E4	Score F4	Coverage F4	# Peptides F4	# PSM F4	Score G4	Coverage G4	# Peptides G4	# PSM G4	Score H4	Coverage H4	# Peptides H4	# PSM H4
GSTUMT00006874001									0	2.54	1	2				
GSTUMT00011675001					0	2.51	1	1								
GSTUMT00006255001									0	2.49	1	1				
GSTUMT00005542001																
GSTUMT00012817001																
GSTUMT00010261001													0	2.33	1	1

	A1I				B1I				C1I				D1I			
Accession	Score E4	Coverage E4	# Peptides E4	# PSM E4	Score F4	Coverage F4	# Peptides F4	# PSM F4	Score G4	Coverage G4	# Peptides G4	# PSM G4	Score H4	Coverage H4	# Peptides H4	# PSM H4
GSTUMT00006412001									0	2.32	1	1				
GSTUMT00000874001	0	2.31	1	1												
GSTUMT00007262001																
GSTUMT00010001001													0	2.3	1	1
GSTUMT00003768001					0	2.3	1	1								
GSTUMT00007761001																

	A1I				B1I				C1I				D1I			
Accession	Score E4	Coverage E4	# Peptides E4	# PSM E4	Score F4	Coverage F4	# Peptides F4	# PSM F4	Score G4	Coverage G4	# Peptides G4	# PSM G4	Score H4	Coverage H4	# Peptides H4	# PSM H4
GSTUMT00001334001	0	2.26	1	1												
GSTUMT00011748001					0	2.26	1	1								
GSTUMT00011209001																
GSTUMT00011415001					61.64	2.16	1	1								
GSTUMT00008736001																
GSTUMT00006281001					0	2.13	1	1								

	A1I				B1I				C1I				D1I			
Accession	Score E4	Coverage E4	# Peptides E4	# PSM E4	Score F4	Coverage F4	# Peptides F4	# PSM F4	Score G4	Coverage G4	# Peptides G4	# PSM G4	Score H4	Coverage H4	# Peptides H4	# PSM H4
GSTUMT00002599001	0	2.12	1	1												
GSTUMT00000583001									0	2.09	1	1	0	2.09	1	1
GSTUMT00012700001													0	2.07	1	1
GSTUMT00007685001																
GSTUMT00012729001					0	2.05	1	1								
GSTUMT00010108001	0	2.02	1	1	0	2.02	1	1								

	A1I				B1I				C1I				D1I			
Accession	Score E4	Coverage E4	# Peptides E4	# PSM E4	Score F4	Coverage F4	# Peptides F4	# PSM F4	Score G4	Coverage G4	# Peptides G4	# PSM G4	Score H4	Coverage H4	# Peptides H4	# PSM H4
GSTUMT00003152001													0	1.11	1	1
GSTUMT00008198001													0	2	1	1
GSTUMT00004165001					0	1.86	1	1								
GSTUMT00007887001																
GSTUMT00001812001																
GSTUMT00004787001									0	1.85	1	1				

	A1I				B1I				C1I				D1I			
Accession	Score E4	Coverage E4	# Peptides E4	# PSM E4	Score F4	Coverage F4	# Peptides F4	# PSM F4	Score G4	Coverage G4	# Peptides G4	# PSM G4	Score H4	Coverage H4	# Peptides H4	# PSM H4
GSTUMT00002932001	0	1.03	1	1												
GSTUMT00009140001	0	1.77	1	1												
GSTUMT00004461001	0	1.77	1	1												
GSTUMT00009834001	0	1.76	1	1												
GSTUMT00010450001					0	1.76	1	1								
GSTUMT00009374001																

	A1I				B1I				C1I				D1I			
Accession	Score E4	Coverage E4	# Peptides E4	# PSM E4	Score F4	Coverage F4	# Peptides F4	# PSM F4	Score G4	Coverage G4	# Peptides G4	# PSM G4	Score H4	Coverage H4	# Peptides H4	# PSM H4
GSTUMT00011323001	0	1.75	1	1												
GSTUMT00005017001	0	1.75	1	1												
GSTUMT00008292001																
GSTUMT00004969001																
GSTUMT00003080001																
GSTUMT00005768001					0	1.7	1	1								

	A1I				B1I				C1I				D1I			
Accession	Score E4	Coverage E4	# Peptides E4	# PSM E4	Score F4	Coverage F4	# Peptides F4	# PSM F4	Score G4	Coverage G4	# Peptides G4	# PSM G4	Score H4	Coverage H4	# Peptides H4	# PSM H4
GSTUMT00003525001																
GSTUMT00010278001					0	1.69	1	1								
GSTUMT00000105001																
GSTUMT00002056001					0	1.68	1	1								
GSTUMT00000660001																
GSTUMT00004057001																

	A1I				B1I				C1I				D1I			
Accession	Score E4	Coverage E4	# Peptides E4	# PSM E4	Score F4	Coverage F4	# Peptides F4	# PSM F4	Score G4	Coverage G4	# Peptides G4	# PSM G4	Score H4	Coverage H4	# Peptides H4	# PSM H4
GSTUMT00009838001	0	1.61	1	1												
GSTUMT00000277001																
GSTUMT00006911001	0	1.6	1	1												
GSTUMT00007159001					39.21	1.6	1	2								
GSTUMT00012580001																
GSTUMT00012371001	0	1.57	1	1												

	A1I				B1I				C1I				D1I			
Accession	Score E4	Coverage E4	# Peptides E4	# PSM E4	Score F4	Coverage F4	# Peptides F4	# PSM F4	Score G4	Coverage G4	# Peptides G4	# PSM G4	Score H4	Coverage H4	# Peptides H4	# PSM H4
GSTUMT00004219001					0	1.56	1	1					0	1.56	1	1
GSTUMT00005928001	0	1.55	1	1												
GSTUMT00002440001					0	1.53	1	1								
GSTUMT00000106001					0	1.53	1	1								
GSTUMT00009341001					0	1.52	1	1								
GSTUMT00003847001					0	1.51	1	1								

	A1I				B1I				C1I				D1I			
Accession	Score E4	Coverage E4	# Peptides E4	# PSM E4	Score F4	Coverage F4	# Peptides F4	# PSM F4	Score G4	Coverage G4	# Peptides G4	# PSM G4	Score H4	Coverage H4	# Peptides H4	# PSM H4
GSTUMT00001078001																
GSTUMT00011312001					0	1.5	1	1								
GSTUMT00001275001																
GSTUMT00004257001	0	1.42	1	1	0	1.42	1	1								
GSTUMT00011007001					0	1.42	1	1								
GSTUMT00008074001									0	1.4	1	1				

	A1I				B1I				C1I				D1I			
Accession	Score E4	Coverage E4	# Peptides E4	# PSM E4	Score F4	Coverage F4	# Peptides F4	# PSM F4	Score G4	Coverage G4	# Peptides G4	# PSM G4	Score H4	Coverage H4	# Peptides H4	# PSM H4
GSTUMT00001308001														0		
GSTUMT00008696001					0	1.37	1	1								
GSTUMT00003182001									0	1.37	1	1				
GSTUMT00004647001									0	1.33	1	1				
GSTUMT00004452001																
GSTUMT00007016001																

	A1I				B1I				C1I				D1I			
Accession	Score E4	Coverage E4	# Peptides E4	# PSM E4	Score F4	Coverage F4	# Peptides F4	# PSM F4	Score G4	Coverage G4	# Peptides G4	# PSM G4	Score H4	Coverage H4	# Peptides H4	# PSM H4
GSTUMT00009675001	36.7	1.32	1	1												
GSTUMT00010640001					45.41	1.31	1	1								
GSTUMT00002283001													0	1.31	1	1
GSTUMT00007918001																
GSTUMT00001196001																
GSTUMT00010240001													0	1.29	1	1

	A1I				B1I				C1I				D1I			
Accession	Score E4	Coverage E4	# Peptides E4	# PSM E4	Score F4	Coverage F4	# Peptides F4	# PSM F4	Score G4	Coverage G4	# Peptides G4	# PSM G4	Score H4	Coverage H4	# Peptides H4	# PSM H4
GSTUMT00003604001									0	0.64	1	1	0	0.64	1	1
GSTUMT00005329001																
GSTUMT00001651001																
GSTUMT00006989001													0	1.26	1	1
GSTUMT00008547001									0	1.25	1	1				
GSTUMT00009359001																

	A1I				B1I				C1I				D1I			
Accession	Score E4	Coverage E4	# Peptides E4	# PSM E4	Score F4	Coverage F4	# Peptides F4	# PSM F4	Score G4	Coverage G4	# Peptides G4	# PSM G4	Score H4	Coverage H4	# Peptides H4	# PSM H4
GSTUMT00008029001																
GSTUMT00004213001	0	1.24	1	1												
GSTUMT00006967001					0	1.24	1	1								
GSTUMT00005973001																
GSTUMT00011138001																
GSTUMT00000199001					0	1.23	1	1								

	A1I				B1I				C1I				D1I			
Accession	Score E4	Coverage E4	# Peptides E4	# PSM E4	Score F4	Coverage F4	# Peptides F4	# PSM F4	Score G4	Coverage G4	# Peptides G4	# PSM G4	Score H4	Coverage H4	# Peptides H4	# PSM H4
GSTUMT00006203001									0	1.23	1	1				
GSTUMT00007732001																
GSTUMT00008870001									0	1.21	1	1				
GSTUMT00004983001									0	1.21	1	1				
GSTUMT00000086001	0	1.2	1	1												
GSTUMT00010828001																

	A1I				B1I				C1I				D1I			
Accession	Score E4	Coverage E4	# Peptides E4	# PSM E4	Score F4	Coverage F4	# Peptides F4	# PSM F4	Score G4	Coverage G4	# Peptides G4	# PSM G4	Score H4	Coverage H4	# Peptides H4	# PSM H4
GSTUMT00005488001									0	1.18	1	1				
GSTUMT00010343001						0							0	1.15	1	1
GSTUMT00011768001																
GSTUMT00005941001																
GSTUMT00003996001																
GSTUMT00001258001					0	1.13	1	1								

	A1I				B1I				C1I				D1I			
Accession	Score E4	Coverage E4	# Peptides E4	# PSM E4	Score F4	Coverage F4	# Peptides F4	# PSM F4	Score G4	Coverage G4	# Peptides G4	# PSM G4	Score H4	Coverage H4	# Peptides H4	# PSM H4
GSTUMT00000754001																
GSTUMT00010843001					0	1.12	1	1								
GSTUMT00010787001					0	1.11	1	1								
GSTUMT00000550001																
GSTUMT00000204001									0	1.09	1	1		0		
GSTUMT00006380001																

	A1I				B1I				C1I				D1I			
Accession	Score E4	Coverage E4	# Peptides E4	# PSM E4	Score F4	Coverage F4	# Peptides F4	# PSM F4	Score G4	Coverage G4	# Peptides G4	# PSM G4	Score H4	Coverage H4	# Peptides H4	# PSM H4
GSTUMT00006298001																
GSTUMT00012201001	0	1.06	1	1												
GSTUMT00009286001	37.23	1.04	1	1	0	1.04	1	1								
GSTUMT00012289001	0	1.04	1	1												
GSTUMT00002124001									0	1.04	1	1				
GSTUMT00010874001																

	A1I				B1I				C1I				D1I			
Accession	Score E4	Coverage E4	# Peptides E4	# PSM E4	Score F4	Coverage F4	# Peptides F4	# PSM F4	Score G4	Coverage G4	# Peptides G4	# PSM G4	Score H4	Coverage H4	# Peptides H4	# PSM H4
GSTUMT00001431001	0	1.03	1	1												
GSTUMT00005156001																
GSTUMT00000895001					0	0.99	1	2								
GSTUMT00011643001									0	0.98	1	1				
GSTUMT00008433001													0	0.97	1	1
GSTUMT00010895001													0	0.96	1	1

	A1I				B1I				C1I				D1I			
Accession	Score E4	Coverage E4	# Peptides E4	# PSM E4	Score F4	Coverage F4	# Peptides F4	# PSM F4	Score G4	Coverage G4	# Peptides G4	# PSM G4	Score H4	Coverage H4	# Peptides H4	# PSM H4
GSTUMT00011664001													0	0.96	1	1
GSTUMT00009187001																
GSTUMT00006670001	0	0.95	1	1												
GSTUMT00000606001					0	0.94	1	1								
GSTUMT00010384001									0	0.94	1	1				
GSTUMT00009188001					0	0.92	1	1						0		

	A1I				B1I				C1I				D1I			
Accession	Score E4	Coverage E4	# Peptides E4	# PSM E4	Score F4	Coverage F4	# Peptides F4	# PSM F4	Score G4	Coverage G4	# Peptides G4	# PSM G4	Score H4	Coverage H4	# Peptides H4	# PSM H4
GSTUMT00010823001									0	0.9	1	1				
GSTUMT00007233001																
GSTUMT00011841001													0	0.88	1	1
GSTUMT00007415001					0	0.87	1	1								
GSTUMT00012096001									0	0.84	1	1				
GSTUMT00004436001	44.55	0.82	1	1												

	A1I				B1I				C1I				D1I			
Accession	Score E4	Coverage E4	# Peptides E4	# PSM E4	Score F4	Coverage F4	# Peptides F4	# PSM F4	Score G4	Coverage G4	# Peptides G4	# PSM G4	Score H4	Coverage H4	# Peptides H4	# PSM H4
GSTUMT00011945001																
GSTUMT00005806001																
GSTUMT00006632001																
GSTUMT00002391001																
GSTUMT00003873001	0	0.8	1	1												
GSTUMT00004929001																

	A1I				B1I				C1I				D1I			
Accession	Score E4	Coverage E4	# Peptides E4	# PSM E4	Score F4	Coverage F4	# Peptides F4	# PSM F4	Score G4	Coverage G4	# Peptides G4	# PSM G4	Score H4	Coverage H4	# Peptides H4	# PSM H4
GSTUMT00008270001													0	0.77	1	1
GSTUMT00003607001					0	0.45	1	1								
GSTUMT00002950001																
GSTUMT00005645001													0	0.75	1	1
GSTUMT00004442001																
GSTUMT00004800001																

	A1I				B1I				C1I				D1I			
Accession	Score E4	Coverage E4	# Peptides E4	# PSM E4	Score F4	Coverage F4	# Peptides F4	# PSM F4	Score G4	Coverage G4	# Peptides G4	# PSM G4	Score H4	Coverage H4	# Peptides H4	# PSM H4
GSTUMT00000478001	0	0.72	1	1												
GSTUMT00004203001																
GSTUMT00010317001	0	0.71	1	1												
GSTUMT00003660001					0	0.71	1	1								
GSTUMT00003361001																
GSTUMT00004542001													0	0.7	1	1

	A1I				B1I				C1I				D1I			
Accession	Score E4	Coverage E4	# Peptides E4	# PSM E4	Score F4	Coverage F4	# Peptides F4	# PSM F4	Score G4	Coverage G4	# Peptides G4	# PSM G4	Score H4	Coverage H4	# Peptides H4	# PSM H4
GSTUMT00004714001	0	0.69	1	1												
GSTUMT00000552001	0	0.68	1	1												
GSTUMT00006726001																
GSTUMT00010538001																
GSTUMT00000473001	0	0.68	1	1												
GSTUMT00011756001													0	0.68	1	1

	A1I				B1I				C1I				D1I			
Accession	Score E4	Coverage E4	# Peptides E4	# PSM E4	Score F4	Coverage F4	# Peptides F4	# PSM F4	Score G4	Coverage G4	# Peptides G4	# PSM G4	Score H4	Coverage H4	# Peptides H4	# PSM H4
GSTUMT00004025001													0	0.68	1	1
GSTUMT00008991001					0	0.65	1	1	0	0.65	1	1				
GSTUMT00009928001					0	0.65	1	1								
GSTUMT00011729001																
GSTUMT00006678001																
GSTUMT00005574001																

	A1I				B1I				C1I				D1I			
Accession	Score E4	Coverage E4	# Peptides E4	# PSM E4	Score F4	Coverage F4	# Peptides F4	# PSM F4	Score G4	Coverage G4	# Peptides G4	# PSM G4	Score H4	Coverage H4	# Peptides H4	# PSM H4
GSTUMT00004673001	0	0.59	1	1												
GSTUMT00011076001					0	0.59	1	1								
GSTUMT00005899001									0	0.59	1	1				
GSTUMT00002146001																
GSTUMT00008484001																
GSTUMT00009648001	0	0.52	1	1												

	A1I				B1I				C1I				D1I			
Accession	Score E4	Coverage E4	# Peptides E4	# PSM E4	Score F4	Coverage F4	# Peptides F4	# PSM F4	Score G4	Coverage G4	# Peptides G4	# PSM G4	Score H4	Coverage H4	# Peptides H4	# PSM H4
GSTUMT00004523001									0	0.52	1	1				
GSTUMT00006345001					0	0.5	1	1								
GSTUMT00012126001	0	0.5	1	1												
GSTUMT00011240001																
GSTUMT00008590001													0	0.49	1	1
GSTUMT00010668001																

	A1I				B1I				C1I				D1I			
Accession	Score E4	Coverage E4	# Peptides E4	# PSM E4	Score F4	Coverage F4	# Peptides F4	# PSM F4	Score G4	Coverage G4	# Peptides G4	# PSM G4	Score H4	Coverage H4	# Peptides H4	# PSM H4
GSTUMT00005225001																
GSTUMT00001634001									0	0.45	1	1				
GSTUMT00000794001						0										
GSTUMT00005369001																
GSTUMT00003624001																
GSTUMT00007495001													0	0.32	1	1

	A1I				B1I				C1I				D1I			
Accession	Score E4	Coverage E4	# Peptides E4	# PSM E4	Score F4	Coverage F4	# Peptides F4	# PSM F4	Score G4	Coverage G4	# Peptides G4	# PSM G4	Score H4	Coverage H4	# Peptides H4	# PSM H4
GSTUMT00002496001									0	0.16	1	1				

Supplementary Table S3: Proteins over-represented inside the brûlé identified by Fisher Exact Test (p value < 0.05).

tr|X5C9W2|X5C9W2_9MAGN	GO:0005737	glyceraldehyde-3-phosphate partial in *Sedum nussbaumerianum*
tr|X5C9W2|X5C9W2_9MAGN	GO:0051287	
tr|X5C9W2|X5C9W2_9MAGN	GO:0050661	
tr|X5C9W2|X5C9W2_9MAGN	GO:0006096	
tr|X5C9W2|X5C9W2_9MAGN	GO:0004365	
tr|X5C9W2|X5C9W2_9MAGN	GO:0055114	
tr|X5C9W2|X5C9W2_9MAGN	EC:1.2.1.12	
tr|U3MV00|U3MV00_TAROF	GO:0051287	glyceraldehyde-3-phosphate dehydrogenase in *Taraxacum officinale*
tr|U3MV00|U3MV00_TAROF	GO:0006006	
tr|U3MV00|U3MV00_TAROF	GO:0004365	
tr|U3MV00|U3MV00_TAROF	GO:0055114	
tr|U3MV00|U3MV00_TAROF	GO:0050661	
tr|U3MV00|U3MV00_TAROF	EC:1.2.1.12	
tr|D2X5N3|D2X5N3_ARTAN	GO:0005737	glyceraldehyde-3-phosphate dehydrogenase in *Artemisia annua*
tr|D2X5N3|D2X5N3_ARTAN	GO:0051287	
tr|D2X5N3|D2X5N3_ARTAN	GO:0050661	
tr|D2X5N3|D2X5N3_ARTAN	GO:0006096	
tr|D2X5N3|D2X5N3_ARTAN	GO:0004365	
tr|D2X5N3|D2X5N3_ARTAN	GO:0055114	
tr|D2X5N3|D2X5N3_ARTAN	EC:1.2.1.12	
sp|P31926|SUSY_VICFA	GO:0009877	sucrose synthase in *Vicia faba*
sp|P31926|SUSY_VICFA	GO:0016157	
sp|P31926|SUSY_VICFA	GO:0009058	
sp|P31926|SUSY_VICFA	GO:0005985	
sp|P31926|SUSY_VICFA	EC:2.4.1.13	
tr|Q2PEX3|Q2PEX3_TRIPR	GO:0006457	heat shock protein in *Trifolium pratense*
tr|Q2PEX3|Q2PEX3_TRIPR	GO:0006950	
tr|Q2PEX3|Q2PEX3_TRIPR	GO:0005524	
tr|Q2PEX3|Q2PEX3_TRIPR	GO:0051082	
tr|Q684J8|Q684J8_LOTJA	GO:0005829	glutamate decarboxylase in *Lotus japonicus*
tr|Q684J8|Q684J8_LOTJA	GO:0005516	
tr|Q684J8|Q684J8_LOTJA	GO:0048767	
tr|Q684J8|Q684J8_LOTJA	GO:0015706	
tr|Q684J8|Q684J8_LOTJA	GO:0004351	
tr|Q684J8|Q684J8_LOTJA	GO:0010359	
tr|Q684J8|Q684J8_LOTJA	GO:0000041	
tr|Q684J8|Q684J8_LOTJA	GO:0006536	
tr|Q684J8|Q684J8_LOTJA	GO:0010167	
tr|Q684J8|Q684J8_LOTJA	GO:0046686	

tr|Q684J8|Q684J8_LOTJA	GO:0030170	
tr|Q684J8|Q684J8_LOTJA	EC:4.1.1.15	
tr|U5IBT8|U5IBT8_TRIRP	GO:0005737	cytosolic ascorbate peroxidase in *Trifolium repens*
tr|U5IBT8|U5IBT8_TRIRP	GO:0046872	
tr|U5IBT8|U5IBT8_TRIRP	GO:0042744	
tr|U5IBT8|U5IBT8_TRIRP	GO:0020037	
tr|U5IBT8|U5IBT8_TRIRP	GO:0016688	
tr|U5IBT8|U5IBT8_TRIRP	GO:0055114	
tr|U5IBT8|U5IBT8_TRIRP	EC:1.11.1.11	
tr|R0GZH8|R0GZH8_9BRAS	GO:0005774	s-adenosyl-l-homocysteine hydrolase in *Capsella rubella*
tr|R0GZH8|R0GZH8_9BRAS	GO:0051788	
tr|R0GZH8|R0GZH8_9BRAS	GO:0009853	
tr|R0GZH8|R0GZH8_9BRAS	GO:0006094	
tr|R0GZH8|R0GZH8_9BRAS	GO:0006346	
tr|R0GZH8|R0GZH8_9BRAS	GO:0048046	
tr|R0GZH8|R0GZH8_9BRAS	GO:0006833	
tr|R0GZH8|R0GZH8_9BRAS	GO:0009793	
tr|R0GZH8|R0GZH8_9BRAS	GO:0000166	
tr|R0GZH8|R0GZH8_9BRAS	GO:0004013	
tr|R0GZH8|R0GZH8_9BRAS	GO:0051049	
tr|R0GZH8|R0GZH8_9BRAS	GO:0006816	
tr|R0GZH8|R0GZH8_9BRAS	GO:0009651	
tr|R0GZH8|R0GZH8_9BRAS	GO:0080129	
tr|R0GZH8|R0GZH8_9BRAS	GO:0048767	
tr|R0GZH8|R0GZH8_9BRAS	GO:0005794	
tr|R0GZH8|R0GZH8_9BRAS	GO:0006972	
tr|R0GZH8|R0GZH8_9BRAS	GO:0005829	
tr|R0GZH8|R0GZH8_9BRAS	GO:0006730	
tr|R0GZH8|R0GZH8_9BRAS	GO:0006511	
tr|R0GZH8|R0GZH8_9BRAS	GO:0007030	
tr|R0GZH8|R0GZH8_9BRAS	GO:0016441	
tr|R0GZH8|R0GZH8_9BRAS	GO:0006096	
tr|R0GZH8|R0GZH8_9BRAS	GO:0046686	
tr|R0GZH8|R0GZH8_9BRAS	GO:0019344	
tr|R0GZH8|R0GZH8_9BRAS	GO:0009506	
tr|R0GZH8|R0GZH8_9BRAS	GO:0009266	
tr|R0GZH8|R0GZH8_9BRAS	GO:0005886	
tr|R0GZH8|R0GZH8_9BRAS	GO:0005507	
tr|R0GZH8|R0GZH8_9BRAS	EC:3.3.1.1	
tr|R0HG12|R0HG12_9BRAS	GO:0007010	tubulin beta-2 beta-3 chain in *Capsella rubella*
tr|R0HG12|R0HG12_9BRAS	GO:0006094	

tr|R0HG12|R0HG12_9BRAS	GO:0005773	
tr|R0HG12|R0HG12_9BRAS	GO:0045298	
tr|R0HG12|R0HG12_9BRAS	GO:0009651	
tr|R0HG12|R0HG12_9BRAS	GO:0010498	
tr|R0HG12|R0HG12_9BRAS	GO:0005525	
tr|R0HG12|R0HG12_9BRAS	GO:0005618	
tr|R0HG12|R0HG12_9BRAS	GO:0006184	
tr|R0HG12|R0HG12_9BRAS	GO:0005794	
tr|R0HG12|R0HG12_9BRAS	GO:0005730	
tr|R0HG12|R0HG12_9BRAS	GO:0005874	
tr|R0HG12|R0HG12_9BRAS	GO:0051258	
tr|R0HG12|R0HG12_9BRAS	GO:0046686	
tr|R0HG12|R0HG12_9BRAS	GO:0009506	
tr|R0HG12|R0HG12_9BRAS	GO:0003924	
tr|R0HG12|R0HG12_9BRAS	GO:0007018	
tr|R0HG12|R0HG12_9BRAS	GO:0005886	
tr|R0HG12|R0HG12_9BRAS	GO:0005200	
tr|I3SNN1|I3SNN1_LOTJA	GO:0004124	cysteine synthase in *Lotus japonicus*
tr|I3SNN1|I3SNN1_LOTJA	GO:0006535	
tr|I3SNN1|I3SNN1_LOTJA	GO:0016740	
tr|I3SNN1|I3SNN1_LOTJA	EC:2.5.1.47	
tr|I3S3D3|I3S3D3_LOTJA	GO:0048046	nucleoside diphosphate kinase 1 in *Lotus japonicus*
tr|I3S3D3|I3S3D3_LOTJA	GO:0004550	
tr|I3S3D3|I3S3D3_LOTJA	GO:0005773	
tr|I3S3D3|I3S3D3_LOTJA	GO:0006241	
tr|I3S3D3|I3S3D3_LOTJA	GO:0009585	
tr|I3S3D3|I3S3D3_LOTJA	GO:0009651	
tr|I3S3D3|I3S3D3_LOTJA	GO:0009507	
tr|I3S3D3|I3S3D3_LOTJA	GO:0005777	
tr|I3S3D3|I3S3D3_LOTJA	GO:0046872	
tr|I3S3D3|I3S3D3_LOTJA	GO:0006165	
tr|I3S3D3|I3S3D3_LOTJA	GO:0006183	
tr|I3S3D3|I3S3D3_LOTJA	GO:0006228	
tr|I3S3D3|I3S3D3_LOTJA	GO:0046686	
tr|I3S3D3|I3S3D3_LOTJA	GO:0005886	
tr|I3S3D3|I3S3D3_LOTJA	GO:0005524	
tr|I3S3D3|I3S3D3_LOTJA	EC:2.7.4.6	
tr|I3T0F4|I3T0F4_LOTJA	GO:0006096	fructose-bisphosphate aldolase in *Lotus japonicus*
tr|I3T0F4|I3T0F4_LOTJA	GO:0005737	
tr|I3T0F4|I3T0F4_LOTJA	GO:0004332	
tr|I3T0F4|I3T0F4_LOTJA	EC:4.1.2.13	

tr|I3T8P4|I3T8P4_LOTJA	GO:0019253	phosphoglycerate chloroplastic-like in *Lotus japonicus*
tr|I3T8P4|I3T8P4_LOTJA	GO:0004618	
tr|I3T8P4|I3T8P4_LOTJA	GO:0016310	
tr|I3T8P4|I3T8P4_LOTJA	GO:0005524	
tr|I3T8P4|I3T8P4_LOTJA	GO:0006096	
tr|I3T8P4|I3T8P4_LOTJA	GO:0009507	
tr|I3T8P4|I3T8P4_LOTJA	EC:2.7.2.3	
tr|L8AJT4|L8AJT4_BACIU	GO:0004013	adenosylhomocysteinase in *Bacillus subtilis*
tr|L8AJT4|L8AJT4_BACIU	GO:0005737	
tr|L8AJT4|L8AJT4_BACIU	GO:0006730	
tr|L8AJT4|L8AJT4_BACIU	EC:3.3.1.1	
tr|D5GIK5|D5GIK5_TUBMM	GO:0004553	tyrosinase precursor in *Tuber melanosporum*
tr|D5GIK5|D5GIK5_TUBMM	GO:0005975	
tr|D5GIK5|D5GIK5_TUBMM	GO:0005576	
tr|D5GIK5|D5GIK5_TUBMM	GO:0046872	
tr|D5GIK5|D5GIK5_TUBMM	GO:0016491	
tr|D5GIK5|D5GIK5_TUBMM	GO:0030248	
tr|D5GIK5|D5GIK5_TUBMM	GO:0055114	
tr|I3SZE9|I3SZE9_LOTJA	GO:0005829	calmodulin in *Lotus japonicus*
tr|I3SZE9|I3SZE9_LOTJA	GO:0055114	
tr|I3SZE9|I3SZE9_LOTJA	GO:0005509	
tr|I3SZE9|I3SZE9_LOTJA	GO:0010099	
tr|I3SZE9|I3SZE9_LOTJA	GO:0006084	
tr|I3SZE9|I3SZE9_LOTJA	GO:0005513	
tr|I3SZE9|I3SZE9_LOTJA	GO:0005774	
tr|I3SZE9|I3SZE9_LOTJA	GO:0005515	
tr|I3SZE9|I3SZE9_LOTJA	GO:0032440	
tr|I3SZE9|I3SZE9_LOTJA	GO:0009846	
tr|I3SZE9|I3SZE9_LOTJA	GO:0019722	
tr|I3SZE9|I3SZE9_LOTJA	GO:0005634	
tr|I3SZE9|I3SZE9_LOTJA	GO:0005886	
tr|I3SZE9|I3SZE9_LOTJA	GO:0009612	
tr|I3SZE9|I3SZE9_LOTJA	EC:1.3.1.74	
tr|R0G5L8|R0G5L8_9BRAS	GO:0003743	translational initiation factor 4a-1 in *Capsella rubella*
tr|R0G5L8|R0G5L8_9BRAS	GO:0006413	
tr|R0G5L8|R0G5L8_9BRAS	GO:0005829	
tr|R0G5L8|R0G5L8_9BRAS	GO:0005618	
tr|R0G5L8|R0G5L8_9BRAS	GO:0005730	
tr|R0G5L8|R0G5L8_9BRAS	GO:0009651	
tr|R0G5L8|R0G5L8_9BRAS	GO:0009506	
tr|R0G5L8|R0G5L8_9BRAS	GO:0008026	

tr|R0G5L8|R0G5L8_9BRAS	GO:0005774	
tr|R0G5L8|R0G5L8_9BRAS	GO:0005524	
tr|R0G5L8|R0G5L8_9BRAS	GO:0005515	
tr|R0G5L8|R0G5L8_9BRAS	GO:0006096	
tr|R0G5L8|R0G5L8_9BRAS	GO:0006094	
tr|R0G5L8|R0G5L8_9BRAS	GO:0046686	
tr|R0G5L8|R0G5L8_9BRAS	GO:0005794	
tr|R0G5L8|R0G5L8_9BRAS	GO:0005886	
tr|R0G5L8|R0G5L8_9BRAS	GO:0048046	
tr|R0GR46|R0GR46_9BRAS	GO:0005829	at5g17920 mpi7_60 in *Capsella rubella*
tr|R0GR46|R0GR46_9BRAS	GO:0005507	
tr|R0GR46|R0GR46_9BRAS	GO:0009086	
tr|R0GR46|R0GR46_9BRAS	GO:0008705	
tr|R0GR46|R0GR46_9BRAS	GO:0008270	
tr|R0GR46|R0GR46_9BRAS	GO:0005777	
tr|R0GR46|R0GR46_9BRAS	GO:0009651	
tr|R0GR46|R0GR46_9BRAS	GO:0016628	
tr|R0GR46|R0GR46_9BRAS	GO:0009506	
tr|R0GR46|R0GR46_9BRAS	GO:0009941	
tr|R0GR46|R0GR46_9BRAS	GO:0010584	
tr|R0GR46|R0GR46_9BRAS	GO:0003871	
tr|R0GR46|R0GR46_9BRAS	GO:0032259	
tr|R0GR46|R0GR46_9BRAS	GO:0005774	
tr|R0GR46|R0GR46_9BRAS	GO:0009570	
tr|R0GR46|R0GR46_9BRAS	GO:0010043	
tr|R0GR46|R0GR46_9BRAS	GO:0046686	
tr|R0GR46|R0GR46_9BRAS	GO:0080019	
tr|R0GR46|R0GR46_9BRAS	GO:0005794	
tr|R0GR46|R0GR46_9BRAS	GO:0005886	
tr|R0GR46|R0GR46_9BRAS	GO:0048046	
tr|R0GR46|R0GR46_9BRAS	EC:2.1.1.13	
tr|R0GR46|R0GR46_9BRAS	EC:2.1.1.14	
tr|Q58ZF1|Q58ZF1_LOTCO	GO:0048767	tubulin beta chain in *Lotus corniculatus*
tr|Q58ZF1|Q58ZF1_LOTCO	GO:0006833	
tr|Q58ZF1|Q58ZF1_LOTCO	GO:0005525	
tr|Q58ZF1|Q58ZF1_LOTCO	GO:0000271	
tr|Q58ZF1|Q58ZF1_LOTCO	GO:0043481	
tr|Q58ZF1|Q58ZF1_LOTCO	GO:0009825	
tr|Q58ZF1|Q58ZF1_LOTCO	GO:0007018	
tr|Q58ZF1|Q58ZF1_LOTCO	GO:0007010	
tr|Q58ZF1|Q58ZF1_LOTCO	GO:0019344	

tr|Q58ZF1|Q58ZF1_LOTCO	GO:0016126	
tr|Q58ZF1|Q58ZF1_LOTCO	GO:0005200	
tr|Q58ZF1|Q58ZF1_LOTCO	GO:0006096	
tr|Q58ZF1|Q58ZF1_LOTCO	GO:0009651	
tr|Q58ZF1|Q58ZF1_LOTCO	GO:0009740	
tr|Q58ZF1|Q58ZF1_LOTCO	GO:0010498	
tr|Q58ZF1|Q58ZF1_LOTCO	GO:0010162	
tr|Q58ZF1|Q58ZF1_LOTCO	GO:0005886	
tr|Q58ZF1|Q58ZF1_LOTCO	GO:0005737	
tr|Q58ZF1|Q58ZF1_LOTCO	GO:0006084	
tr|Q58ZF1|Q58ZF1_LOTCO	GO:0051258	
tr|Q58ZF1|Q58ZF1_LOTCO	GO:0016132	
tr|Q58ZF1|Q58ZF1_LOTCO	GO:0005874	
tr|Q58ZF1|Q58ZF1_LOTCO	GO:0006972	
tr|Q58ZF1|Q58ZF1_LOTCO	GO:0003924	
tr|Q58ZF1|Q58ZF1_LOTCO	GO:0046686	
tr|Q58ZF1|Q58ZF1_LOTCO	GO:0009409	
tr|Q58ZF1|Q58ZF1_LOTCO	GO:0009932	
tr|Q58ZF1|Q58ZF1_LOTCO	GO:0007030	
tr|Q58ZF1|Q58ZF1_LOTCO	GO:0006184	
tr|Q58ZF1|Q58ZF1_LOTCO	GO:0006094	
tr|Q58ZF1|Q58ZF1_LOTCO	GO:0071555	
tr|R0F5B5|R0F5B5_9BRAS	GO:0005774	tubulin beta-9 chain in *Capsella rubella*
tr|R0F5B5|R0F5B5_9BRAS	GO:0007010	
tr|R0F5B5|R0F5B5_9BRAS	GO:0006094	
tr|R0F5B5|R0F5B5_9BRAS	GO:0048046	
tr|R0F5B5|R0F5B5_9BRAS	GO:0045298	
tr|R0F5B5|R0F5B5_9BRAS	GO:0010498	
tr|R0F5B5|R0F5B5_9BRAS	GO:0005525	
tr|R0F5B5|R0F5B5_9BRAS	GO:0005618	
tr|R0F5B5|R0F5B5_9BRAS	GO:0006184	
tr|R0F5B5|R0F5B5_9BRAS	GO:0005794	
tr|R0F5B5|R0F5B5_9BRAS	GO:0005829	
tr|R0F5B5|R0F5B5_9BRAS	GO:0005874	
tr|R0F5B5|R0F5B5_9BRAS	GO:0051258	
tr|R0F5B5|R0F5B5_9BRAS	GO:0009570	
tr|R0F5B5|R0F5B5_9BRAS	GO:0046686	
tr|R0F5B5|R0F5B5_9BRAS	GO:0009506	
tr|R0F5B5|R0F5B5_9BRAS	GO:0003924	
tr|R0F5B5|R0F5B5_9BRAS	GO:0007018	
tr|R0F5B5|R0F5B5_9BRAS	GO:0005886	

tr|R0F5B5|R0F5B5_9BRAS	GO:0005200	
tr|R0INE5|R0INE5_9BRAS	GO:0001510	pointed first leaf in *Capsella rubella*
tr|R0INE5|R0INE5_9BRAS	GO:0006413	
tr|R0INE5|R0INE5_9BRAS	GO:0005618	
tr|R0INE5|R0INE5_9BRAS	GO:0003723	
tr|R0INE5|R0INE5_9BRAS	GO:0005730	
tr|R0INE5|R0INE5_9BRAS	GO:0009506	
tr|R0INE5|R0INE5_9BRAS	GO:0003735	
tr|R0INE5|R0INE5_9BRAS	GO:0022627	
tr|R0INE5|R0INE5_9BRAS	GO:0005774	
tr|R0INE5|R0INE5_9BRAS	GO:0042254	
tr|R0INE5|R0INE5_9BRAS	GO:0005515	
tr|R0INE5|R0INE5_9BRAS	GO:0005794	
tr|R0INE5|R0INE5_9BRAS	GO:0005886	
tr|A0A059XMU8|A0A059XMU8_LATSA	GO:0009750	l-3-cyanoalanine synthase mitochondrial-like in *Lathyrus sativus*
tr|A0A059XMU8|A0A059XMU8_LATSA	GO:0042744	
tr|A0A059XMU8|A0A059XMU8_LATSA	GO:0006833	
tr|A0A059XMU8|A0A059XMU8_LATSA	GO:0051410	
tr|A0A059XMU8|A0A059XMU8_LATSA	GO:0080147	
tr|A0A059XMU8|A0A059XMU8_LATSA	GO:0009651	
tr|A0A059XMU8|A0A059XMU8_LATSA	GO:0016740	
tr|A0A059XMU8|A0A059XMU8_LATSA	GO:0009507	
tr|A0A059XMU8|A0A059XMU8_LATSA	GO:0004124	
tr|A0A059XMU8|A0A059XMU8_LATSA	GO:0006972	
tr|A0A059XMU8|A0A059XMU8_LATSA	GO:0019288	
tr|A0A059XMU8|A0A059XMU8_LATSA	GO:0050017	
tr|A0A059XMU8|A0A059XMU8_LATSA	GO:0007030	
tr|A0A059XMU8|A0A059XMU8_LATSA	GO:0006535	
tr|A0A059XMU8|A0A059XMU8_LATSA	GO:0006096	
tr|A0A059XMU8|A0A059XMU8_LATSA	GO:0032880	
tr|A0A059XMU8|A0A059XMU8_LATSA	GO:0030170	
tr|A0A059XMU8|A0A059XMU8_LATSA	GO:0019761	
tr|A0A059XMU8|A0A059XMU8_LATSA	GO:0019500	
tr|A0A059XMU8|A0A059XMU8_LATSA	GO:0046686	
tr|A0A059XMU8|A0A059XMU8_LATSA	GO:0009684	
tr|A0A059XMU8|A0A059XMU8_LATSA	GO:0009266	
tr|A0A059XMU8|A0A059XMU8_LATSA	GO:0009836	
tr|A0A059XMU8|A0A059XMU8_LATSA	GO:0005507	
tr|A0A059XMU8|A0A059XMU8_LATSA	GO:0005739	
tr|A0A059XMU8|A0A059XMU8_LATSA	EC:4.4.1.9	
tr|A0A059XMU8|A0A059XMU8_LATSA	EC:2.5.1.47	

tr|N1VC98|N1VC98_9MICC	GO:0043797	glyceraldehyde-3-phosphate dehydrogenase in *Arthrobacter crystallopoietes*
tr|N1VC98|N1VC98_9MICC	GO:0051287	
tr|N1VC98|N1VC98_9MICC	GO:0006006	
tr|N1VC98|N1VC98_9MICC	GO:0004365	
tr|N1VC98|N1VC98_9MICC	GO:0055114	
tr|N1VC98|N1VC98_9MICC	GO:0050661	
tr|N1VC98|N1VC98_9MICC	EC:1.2.1.12	
tr|N1VC98|N1VC98_9MICC	EC:1.2.7.6	
tr|W7ZA32|W7ZA32_9BACI	GO:0046933	f0f1 atp synthase subunit beta in *Bacillus*
tr|W7ZA32|W7ZA32_9BACI	GO:0015991	
tr|W7ZA32|W7ZA32_9BACI	GO:0042777	
tr|W7ZA32|W7ZA32_9BACI	GO:0045261	
tr|W7ZA32|W7ZA32_9BACI	GO:0005886	
tr|W7ZA32|W7ZA32_9BACI	GO:0005524	
tr|J3C4Y2|J3C4Y2_9FLAO	GO:0005975	glycosyl hydrolase family 3 in *Flavobacterium*
tr|J3C4Y2|J3C4Y2_9FLAO	GO:0004553	
tr|I3SH51|I3SH51_LOTJA	GO:0005840	60s ribosomal protein l5-like in *Lotus japonicus*
tr|I3SH51|I3SH51_LOTJA	GO:0008097	
tr|I3SH51|I3SH51_LOTJA	GO:0003735	
tr|I3SH51|I3SH51_LOTJA	GO:0006412	
tr|R0GU86|R0GU86_9BRAS	GO:0005829	luminal binding protein in *Capsella rubella*
tr|R0GU86|R0GU86_9BRAS	GO:0005618	
tr|R0GU86|R0GU86_9BRAS	GO:0034976	
tr|R0GU86|R0GU86_9BRAS	GO:0006457	
tr|R0GU86|R0GU86_9BRAS	GO:0005730	
tr|R0GU86|R0GU86_9BRAS	GO:0009506	
tr|R0GU86|R0GU86_9BRAS	GO:0010197	
tr|R0GU86|R0GU86_9BRAS	GO:0009408	
tr|R0GU86|R0GU86_9BRAS	GO:0005774	
tr|R0GU86|R0GU86_9BRAS	GO:0005524	
tr|R0GU86|R0GU86_9BRAS	GO:0005788	
tr|R0GU86|R0GU86_9BRAS	GO:0016592	
tr|R0GU86|R0GU86_9BRAS	GO:0009507	
tr|R0GU86|R0GU86_9BRAS	GO:0046686	
tr|R0GU86|R0GU86_9BRAS	GO:0005794	
tr|R0GU86|R0GU86_9BRAS	GO:0005886	
tr|R0GKE5|R0GKE5_9BRAS	GO:0016132	atp citrate lyase in *Capsella rubella*
tr|R0GKE5|R0GKE5_9BRAS	GO:0006085	
tr|R0GKE5|R0GKE5_9BRAS	GO:0005829	
tr|R0GKE5|R0GKE5_9BRAS	GO:0016926	
tr|R0GKE5|R0GKE5_9BRAS	GO:0016829	

tr|R0GKE5|R0GKE5_9BRAS	GO:0003878	
tr|R0GKE5|R0GKE5_9BRAS	GO:0009346	
tr|R0GKE5|R0GKE5_9BRAS	GO:0010228	
tr|R0GKE5|R0GKE5_9BRAS	GO:0044262	
tr|R0GKE5|R0GKE5_9BRAS	GO:0004775	
tr|R0GKE5|R0GKE5_9BRAS	GO:0000166	
tr|R0GKE5|R0GKE5_9BRAS	GO:0050665	
tr|R0GKE5|R0GKE5_9BRAS	GO:0048037	
tr|R0GKE5|R0GKE5_9BRAS	GO:0005886	
tr|R0GKE5|R0GKE5_9BRAS	GO:0016126	
tr|R0GKE5|R0GKE5_9BRAS	EC:6.2.1.5	
tr|R0GKE5|R0GKE5_9BRAS	EC:2.3.3.8	
tr|R0HKB9|R0HKB9_9BRAS	GO:0005507	chaperonin cpn60- mitochondrial-like in *Capsella rubella*
tr|R0HKB9|R0HKB9_9BRAS	GO:0042026	
tr|R0HKB9|R0HKB9_9BRAS	GO:0034976	
tr|R0HKB9|R0HKB9_9BRAS	GO:0006626	
tr|R0HKB9|R0HKB9_9BRAS	GO:0009408	
tr|R0HKB9|R0HKB9_9BRAS	GO:0005774	
tr|R0HKB9|R0HKB9_9BRAS	GO:0005524	
tr|R0HKB9|R0HKB9_9BRAS	GO:0005759	
tr|R0HKB9|R0HKB9_9BRAS	GO:0009507	
tr|R0HKB9|R0HKB9_9BRAS	GO:0051131	
tr|R0HKB9|R0HKB9_9BRAS	GO:0046686	
tr|R0HKB9|R0HKB9_9BRAS	GO:0009644	
tr|R0HKB9|R0HKB9_9BRAS	GO:0042542	
tr|R0HKB9|R0HKB9_9BRAS	GO:0022626	
tr|R0HKB9|R0HKB9_9BRAS	GO:0005794	
tr|R0HKB9|R0HKB9_9BRAS	GO:0005886	
tr|R0FNU6|R0FNU6_9BRAS	GO:0009750	fructose bisphosphate aldolase in *Capsella rubella*
tr|R0FNU6|R0FNU6_9BRAS	GO:0005774	
tr|R0FNU6|R0FNU6_9BRAS	GO:0051788	
tr|R0FNU6|R0FNU6_9BRAS	GO:0009853	
tr|R0FNU6|R0FNU6_9BRAS	GO:0006098	
tr|R0FNU6|R0FNU6_9BRAS	GO:0006094	
tr|R0FNU6|R0FNU6_9BRAS	GO:0009749	
tr|R0FNU6|R0FNU6_9BRAS	GO:0048046	
tr|R0FNU6|R0FNU6_9BRAS	GO:0009744	
tr|R0FNU6|R0FNU6_9BRAS	GO:0006833	
tr|R0FNU6|R0FNU6_9BRAS	GO:0009651	
tr|R0FNU6|R0FNU6_9BRAS	GO:0080167	
tr|R0FNU6|R0FNU6_9BRAS	GO:0080129	

tr|R0FNU6|R0FNU6_9BRAS	GO:0009507	
tr|R0FNU6|R0FNU6_9BRAS	GO:0005618	
tr|R0FNU6|R0FNU6_9BRAS	GO:0005794	
tr|R0FNU6|R0FNU6_9BRAS	GO:0006972	
tr|R0FNU6|R0FNU6_9BRAS	GO:0005730	
tr|R0FNU6|R0FNU6_9BRAS	GO:0005829	
tr|R0FNU6|R0FNU6_9BRAS	GO:0006511	
tr|R0FNU6|R0FNU6_9BRAS	GO:0007030	
tr|R0FNU6|R0FNU6_9BRAS	GO:0006096	
tr|R0FNU6|R0FNU6_9BRAS	GO:0046686	
tr|R0FNU6|R0FNU6_9BRAS	GO:0005740	
tr|R0FNU6|R0FNU6_9BRAS	GO:0009506	
tr|R0FNU6|R0FNU6_9BRAS	GO:0009266	
tr|R0FNU6|R0FNU6_9BRAS	GO:0004332	
tr|R0FNU6|R0FNU6_9BRAS	GO:0005886	
tr|R0FNU6|R0FNU6_9BRAS	GO:0005507	
tr|R0FNU6|R0FNU6_9BRAS	GO:0006085	
tr|R0FNU6|R0FNU6_9BRAS	EC:4.1.2.13	
tr|Q9XHC7|Q9XHC7_LOTCO	GO:0005829	phosphoenolpyruvate carboxylase in *Lotus corniculatus*
tr|Q9XHC7|Q9XHC7_LOTCO	GO:0006099	
tr|Q9XHC7|Q9XHC7_LOTCO	GO:0008964	
tr|Q9XHC7|Q9XHC7_LOTCO	GO:0015979	
tr|Q9XHC7|Q9XHC7_LOTCO	GO:0015977	
tr|Q9XHC7|Q9XHC7_LOTCO	EC:4.1.1.31	
tr|R0FV42|R0FV42_9BRAS	GO:0005774	heat shock protein 60-2 in *Capsella rubella*
tr|R0FV42|R0FV42_9BRAS	GO:0007010	
tr|R0FV42|R0FV42_9BRAS	GO:0006094	
tr|R0FV42|R0FV42_9BRAS	GO:0009941	
tr|R0FV42|R0FV42_9BRAS	GO:0042542	
tr|R0FV42|R0FV42_9BRAS	GO:0009220	
tr|R0FV42|R0FV42_9BRAS	GO:0022626	
tr|R0FV42|R0FV42_9BRAS	GO:0005759	
tr|R0FV42|R0FV42_9BRAS	GO:0010498	
tr|R0FV42|R0FV42_9BRAS	GO:0051131	
tr|R0FV42|R0FV42_9BRAS	GO:0005794	
tr|R0FV42|R0FV42_9BRAS	GO:0034976	
tr|R0FV42|R0FV42_9BRAS	GO:0009570	
tr|R0FV42|R0FV42_9BRAS	GO:0009644	
tr|R0FV42|R0FV42_9BRAS	GO:0006954	
tr|R0FV42|R0FV42_9BRAS	GO:0046686	
tr|R0FV42|R0FV42_9BRAS	GO:0009408	

tr|R0FV42|R0FV42_9BRAS	GO:0042026	
tr|R0FV42|R0FV42_9BRAS	GO:0005886	
tr|R0FV42|R0FV42_9BRAS	GO:0005507	
tr|R0FV42|R0FV42_9BRAS	GO:0005524	
tr|R0FV42|R0FV42_9BRAS	GO:0006626	
tr|R0GRF1|R0GRF1_9BRAS	GO:0001510	60s ribosomal protein l10 in *Capsella rubella*
tr|R0GRF1|R0GRF1_9BRAS	GO:0006412	
tr|R0GRF1|R0GRF1_9BRAS	GO:0005730	
tr|R0GRF1|R0GRF1_9BRAS	GO:0003735	
tr|R0GRF1|R0GRF1_9BRAS	GO:0009941	
tr|R0GRF1|R0GRF1_9BRAS	GO:0071493	
tr|R0GRF1|R0GRF1_9BRAS	GO:0005774	
tr|R0GRF1|R0GRF1_9BRAS	GO:0005515	
tr|R0GRF1|R0GRF1_9BRAS	GO:0032502	
tr|R0GRF1|R0GRF1_9BRAS	GO:0022625	
tr|R0GRF1|R0GRF1_9BRAS	GO:0005794	
tr|R0GRF1|R0GRF1_9BRAS	GO:0005886	
tr|R0FX04|R0FX04_9BRAS	GO:0004747	pfkb-type carbohydrate kinase family protein in *Capsella rubella*
tr|R0FX04|R0FX04_9BRAS	GO:0006014	
tr|R0FX04|R0FX04_9BRAS	GO:0009744	
tr|R0FX04|R0FX04_9BRAS	GO:0005794	
tr|R0FX04|R0FX04_9BRAS	GO:0009506	
tr|R0FX04|R0FX04_9BRAS	GO:0005886	
tr|R0FX04|R0FX04_9BRAS	GO:0016310	
tr|R0FX04|R0FX04_9BRAS	GO:0009749	
tr|R0FX04|R0FX04_9BRAS	GO:0009750	
tr|R0FX04|R0FX04_9BRAS	GO:0005829	
tr|R0FX04|R0FX04_9BRAS	GO:0000041	
tr|R0FX04|R0FX04_9BRAS	EC:2.7.1.15	
tr|E3Q1S2|E3Q1S2_9ROSI	GO:0010413	isocitrate dehydrogenase in *Quercus pubescens*
tr|E3Q1S2|E3Q1S2_9ROSI	GO:0051287	
tr|E3Q1S2|E3Q1S2_9ROSI	GO:0005777	
tr|E3Q1S2|E3Q1S2_9ROSI	GO:0006099	
tr|E3Q1S2|E3Q1S2_9ROSI	GO:0004450	
tr|E3Q1S2|E3Q1S2_9ROSI	GO:0000287	
tr|E3Q1S2|E3Q1S2_9ROSI	GO:0045492	
tr|E3Q1S2|E3Q1S2_9ROSI	GO:0006102	
tr|E3Q1S2|E3Q1S2_9ROSI	GO:0009507	
tr|E3Q1S2|E3Q1S2_9ROSI	GO:0005886	
tr|E3Q1S2|E3Q1S2_9ROSI	EC:1.1.1.42	
tr|R0FHR7|R0FHR7_9BRAS	GO:0009086	ras-like gtp-binding protein in *Capsella rubella*

tr|R0FHR7|R0FHR7_9BRAS	GO:0007264	
tr|R0FHR7|R0FHR7_9BRAS	GO:0005515	
tr|R0FHR7|R0FHR7_9BRAS	GO:0000394	
tr|R0FHR7|R0FHR7_9BRAS	GO:0005773	
tr|R0FHR7|R0FHR7_9BRAS	GO:0005525	
tr|R0FHR7|R0FHR7_9BRAS	GO:0005886	
tr|R0FHR7|R0FHR7_9BRAS	GO:0009873	
tr|R0FHR7|R0FHR7_9BRAS	GO:0003924	
tr|R0FHR7|R0FHR7_9BRAS	GO:0006886	
tr|R0FHR7|R0FHR7_9BRAS	GO:0006913	
tr|I3T4F6|I3T4F6_LOTJA	GO:0005829	late embryogenesis abundant group 2 isoform 1 in *Lotus japonicus*
tr|I3T4F6|I3T4F6_LOTJA	GO:0016049	
tr|I3T4F6|I3T4F6_LOTJA	GO:0009651	
tr|I3T4F6|I3T4F6_LOTJA	GO:0009269	
tr|I3T4F6|I3T4F6_LOTJA	GO:0009506	
tr|I3T4F6|I3T4F6_LOTJA	GO:0000902	
tr|I3T4F6|I3T4F6_LOTJA	GO:0006096	
tr|I3T4F6|I3T4F6_LOTJA	GO:0048193	
tr|I3T4F6|I3T4F6_LOTJA	GO:0006094	
tr|I3T4F6|I3T4F6_LOTJA	GO:0019344	
tr|I3T4F6|I3T4F6_LOTJA	GO:0005794	
tr|I3T4F6|I3T4F6_LOTJA	GO:0005886	
tr|I3T4F6|I3T4F6_LOTJA	GO:0009793	
tr|I3T5V4|I3T5V4_LOTJA	GO:0033808	chalcone reductase in *Lotus japonicus*
tr|I3T5V4|I3T5V4_LOTJA	GO:0016491	
tr|I3T5V4|I3T5V4_LOTJA	GO:0055114	
tr|I3T5V4|I3T5V4_LOTJA	GO:0009813	
tr|I3T5V4|I3T5V4_LOTJA	EC:2.3.1.170	
tr|Q2PEW2|Q2PEW2_TRIPR	GO:0051539	aconitate hydratase in *Trifolium pratense*
tr|Q2PEW2|Q2PEW2_TRIPR	GO:0052633	
tr|Q2PEW2|Q2PEW2_TRIPR	GO:0008152	
tr|Q2PEW2|Q2PEW2_TRIPR	GO:0003994	
tr|Q2PEW2|Q2PEW2_TRIPR	GO:0047780	
tr|Q2PEW2|Q2PEW2_TRIPR	EC:4.2.1.4	
tr|Q2PEW2|Q2PEW2_TRIPR	EC:4.2.1.3	
tr|I3T2W0|I3T2W0_LOTJA	GO:0050625	flavoprotein wrba in *Lotus japonicus*
tr|I3T2W0|I3T2W0_LOTJA	GO:0005829	
tr|I3T2W0|I3T2W0_LOTJA	GO:0055114	
tr|I3T2W0|I3T2W0_LOTJA	GO:0009506	
tr|I3T2W0|I3T2W0_LOTJA	GO:0005774	
tr|I3T2W0|I3T2W0_LOTJA	GO:0009733	

tr|I3T2W0|I3T2W0_LOTJA	GO:0006970	
tr|I3T2W0|I3T2W0_LOTJA	GO:0005576	
tr|I3T2W0|I3T2W0_LOTJA	GO:0045892	
tr|I3T2W0|I3T2W0_LOTJA	GO:0046686	
tr|I3T2W0|I3T2W0_LOTJA	GO:0010181	
tr|I3T2W0|I3T2W0_LOTJA	GO:0005886	
tr|I3T2W0|I3T2W0_LOTJA	EC:1.6.5.7	
tr|Q3LVQ8|Q3LVQ8_TAROF	GO:0000278	mms zwei-like protein 3 in *Taraxacum officinale*
tr|Q3LVQ8|Q3LVQ8_TAROF	GO:0006007	
tr|Q3LVQ8|Q3LVQ8_TAROF	GO:0016192	
tr|Q3LVQ8|Q3LVQ8_TAROF	GO:0006301	
tr|Q3LVQ8|Q3LVQ8_TAROF	GO:0005829	
tr|Q3LVQ8|Q3LVQ8_TAROF	GO:0006396	
tr|Q3LVQ8|Q3LVQ8_TAROF	GO:0006511	
tr|Q3LVQ8|Q3LVQ8_TAROF	GO:0006623	
tr|Q3LVQ8|Q3LVQ8_TAROF	GO:0004842	
tr|Q3LVQ8|Q3LVQ8_TAROF	GO:0016567	
tr|Q3LVQ8|Q3LVQ8_TAROF	GO:0005515	
tr|Q3LVQ8|Q3LVQ8_TAROF	GO:0031372	
tr|Q3LVQ8|Q3LVQ8_TAROF	GO:0005634	
tr|Q3LVQ8|Q3LVQ8_TAROF	EC:6.3.2.19	
tr|Q6Q4Z3|Q6Q4Z3_CAPBU	GO:0005507	enolase in *Capsella bursa-pastoris*
tr|Q6Q4Z3|Q6Q4Z3_CAPBU	GO:0006098	
tr|Q6Q4Z3|Q6Q4Z3_CAPBU	GO:0009651	
tr|Q6Q4Z3|Q6Q4Z3_CAPBU	GO:0009416	
tr|Q6Q4Z3|Q6Q4Z3_CAPBU	GO:0009506	
tr|Q6Q4Z3|Q6Q4Z3_CAPBU	GO:0009409	
tr|Q6Q4Z3|Q6Q4Z3_CAPBU	GO:0000015	
tr|Q6Q4Z3|Q6Q4Z3_CAPBU	GO:0003677	
tr|Q6Q4Z3|Q6Q4Z3_CAPBU	GO:0009737	
tr|Q6Q4Z3|Q6Q4Z3_CAPBU	GO:0000287	
tr|Q6Q4Z3|Q6Q4Z3_CAPBU	GO:0006096	
tr|Q6Q4Z3|Q6Q4Z3_CAPBU	GO:0006094	
tr|Q6Q4Z3|Q6Q4Z3_CAPBU	GO:0005740	
tr|Q6Q4Z3|Q6Q4Z3_CAPBU	GO:0009853	
tr|Q6Q4Z3|Q6Q4Z3_CAPBU	GO:0009507	
tr|Q6Q4Z3|Q6Q4Z3_CAPBU	GO:0046686	
tr|Q6Q4Z3|Q6Q4Z3_CAPBU	GO:0004634	
tr|Q6Q4Z3|Q6Q4Z3_CAPBU	GO:0005634	
tr|Q6Q4Z3|Q6Q4Z3_CAPBU	GO:0005886	
tr|Q6Q4Z3|Q6Q4Z3_CAPBU	GO:0048046	

tr|Q6Q4Z3|Q6Q4Z3_CAPBU EC:4.2.1.11
tr|I3S0W6|I3S0W6_LOTJA GO:0022626 proteasome subunit alpha type-5-like in *Lotus japonicus*
tr|I3S0W6|I3S0W6_LOTJA GO:0019773
tr|I3S0W6|I3S0W6_LOTJA GO:0046686
tr|I3S0W6|I3S0W6_LOTJA GO:0005886
tr|I3S0W6|I3S0W6_LOTJA GO:0006511
tr|I3S0W6|I3S0W6_LOTJA GO:0005634
tr|I3S0W6|I3S0W6_LOTJA GO:0004298
tr|I3S0W6|I3S0W6_LOTJA EC:3.4.25
tr|R0HNM5|R0HNM5_9BRAS GO:0000085 proteasome subunit alpha type-5-a in *Capsella rubella*
tr|R0HNM5|R0HNM5_9BRAS GO:0005774
tr|R0HNM5|R0HNM5_9BRAS GO:0051788
tr|R0HNM5|R0HNM5_9BRAS GO:0004540
tr|R0HNM5|R0HNM5_9BRAS GO:0009853
tr|R0HNM5|R0HNM5_9BRAS GO:0006094
tr|R0HNM5|R0HNM5_9BRAS GO:0006635
tr|R0HNM5|R0HNM5_9BRAS GO:0043161
tr|R0HNM5|R0HNM5_9BRAS GO:0022626
tr|R0HNM5|R0HNM5_9BRAS GO:0009651
tr|R0HNM5|R0HNM5_9BRAS GO:0004298
tr|R0HNM5|R0HNM5_9BRAS GO:0010388
tr|R0HNM5|R0HNM5_9BRAS GO:0009640
tr|R0HNM5|R0HNM5_9BRAS GO:0019773
tr|R0HNM5|R0HNM5_9BRAS GO:0006096
tr|R0HNM5|R0HNM5_9BRAS GO:0046686
tr|R0HNM5|R0HNM5_9BRAS GO:0005886
tr|R0HNM5|R0HNM5_9BRAS GO:0005634
tr|R0HNM5|R0HNM5_9BRAS GO:0043248
tr|R0HNM5|R0HNM5_9BRAS EC:3.4.25
tr|R0HKA2|R0HKA2_9BRAS GO:0005773 60s ribosomal protein l8 in *Capsella rubella*
tr|R0HKA2|R0HKA2_9BRAS GO:0003735
tr|R0HKA2|R0HKA2_9BRAS GO:0005886
tr|R0HKA2|R0HKA2_9BRAS GO:0003723
tr|R0HKA2|R0HKA2_9BRAS GO:0022625
tr|R0HKA2|R0HKA2_9BRAS GO:0006412
tr|A0A075EAM3|A0A075EAM3_ASTMEGO:0005737 farnesyl pyrophosphate synthase in *Astragalus membranaceus*
tr|A0A075EAM3|A0A075EAM3_ASTMEGO:0046872
tr|A0A075EAM3|A0A075EAM3_ASTMEGO:0004337
tr|A0A075EAM3|A0A075EAM3_ASTMEGO:0033384
tr|A0A075EAM3|A0A075EAM3_ASTMEGO:0006695
tr|A0A075EAM3|A0A075EAM3_ASTMEGO:0004161

tr|A0A075EAM3|A0A075EAM3_ASTMEGO:0045337		
tr|A0A075EAM3|A0A075EAM3_ASTMEEC:2.5.1.10		
tr|A0A075EAM3|A0A075EAM3_ASTMEEC:2.5.1.1		
tr|R0I261|R0I261_9BRAS	GO:0005829	6-phosphogluconate decarboxylating 3 in *Capsella rubella*
tr|R0I261|R0I261_9BRAS	GO:0009749	
tr|R0I261|R0I261_9BRAS	GO:0009744	
tr|R0I261|R0I261_9BRAS	GO:0009750	
tr|R0I261|R0I261_9BRAS	GO:0005777	
tr|R0I261|R0I261_9BRAS	GO:0006098	
tr|R0I261|R0I261_9BRAS	GO:0009651	
tr|R0I261|R0I261_9BRAS	GO:0043090	
tr|R0I261|R0I261_9BRAS	GO:0010359	
tr|R0I261|R0I261_9BRAS	GO:0006888	
tr|R0I261|R0I261_9BRAS	GO:0009570	
tr|R0I261|R0I261_9BRAS	GO:0004616	
tr|R0I261|R0I261_9BRAS	GO:0050661	
tr|R0I261|R0I261_9BRAS	EC:1.1.1.44	
tr|D6PPS9|D6PPS9_9BRAS	GO:0005829	at3g02360-like protein in *Capsella rubella*
tr|D6PPS9|D6PPS9_9BRAS	GO:0009749	
tr|D6PPS9|D6PPS9_9BRAS	GO:0009744	
tr|D6PPS9|D6PPS9_9BRAS	GO:0009750	
tr|D6PPS9|D6PPS9_9BRAS	GO:0005777	
tr|D6PPS9|D6PPS9_9BRAS	GO:0006098	
tr|D6PPS9|D6PPS9_9BRAS	GO:0009651	
tr|D6PPS9|D6PPS9_9BRAS	GO:0043090	
tr|D6PPS9|D6PPS9_9BRAS	GO:0010359	
tr|D6PPS9|D6PPS9_9BRAS	GO:0006888	
tr|D6PPS9|D6PPS9_9BRAS	GO:0009570	
tr|D6PPS9|D6PPS9_9BRAS	GO:0004616	
tr|D6PPS9|D6PPS9_9BRAS	GO:0050661	
tr|D6PPS9|D6PPS9_9BRAS	EC:1.1.1.44	
tr|A0A059WQB8|A0A059WQB8_QUESU	GO:0003743	eukaryotic translation initiation factor 5a in *Quercus suber*
tr|A0A059WQB8|A0A059WQB8_QUESU	GO:0006413	
tr|A0A059WQB8|A0A059WQB8_QUESU	GO:0003746	
tr|A0A059WQB8|A0A059WQB8_QUESU	GO:0006452	
tr|A0A059WQB8|A0A059WQB8_QUESU	GO:0034050	
tr|A0A059WQB8|A0A059WQB8_QUESU	GO:0042742	
tr|A0A059WQB8|A0A059WQB8_QUESU	GO:0009611	
tr|A0A059WQB8|A0A059WQB8_QUESU	GO:0008612	
tr|A0A059WQB8|A0A059WQB8_QUESU	GO:0045905	
tr|A0A059WQB8|A0A059WQB8_QUESU	GO:0043022	

tr|A0A059WQB8|A0A059WQB8_QUESU	GO:0010089	
tr|A0A059WQB8|A0A059WQB8_QUESU	GO:0045901	
tr|A0A059WQB8|A0A059WQB8_QUESU	GO:0046686	
tr|A0A059WQB8|A0A059WQB8_QUESU	GO:0005634	
tr|I3S9M6|I3S9M6_LOTJA	GO:0005525	ras-related protein raba4c-like in *Lotus japonicus*
tr|I3S9M6|I3S9M6_LOTJA	GO:0016787	
tr|I3S9M6|I3S9M6_LOTJA	GO:0016192	
tr|I3S9M6|I3S9M6_LOTJA	GO:0007264	
tr|I3S9M6|I3S9M6_LOTJA	GO:0015031	
tr|U4L650|U4L650_PYROM	GO:0005737	atp-dependent molecular chaperone hsc82 in *Pyronema omphalodes*
tr|U4L650|U4L650_PYROM	GO:0006457	
tr|U4L650|U4L650_PYROM	GO:0006950	
tr|U4L650|U4L650_PYROM	GO:0005524	
tr|U4L650|U4L650_PYROM	GO:0051082	
tr|R0G9G5|R0G9G5_9BRAS	GO:0006457	heat shock protein isoform 2 in *Capsella rubella*
tr|R0G9G5|R0G9G5_9BRAS	GO:0006950	
tr|R0G9G5|R0G9G5_9BRAS	GO:0005618	
tr|R0G9G5|R0G9G5_9BRAS	GO:0005524	
tr|R0G9G5|R0G9G5_9BRAS	GO:0009570	
tr|R0G9G5|R0G9G5_9BRAS	GO:0051082	
tr|R0G9G5|R0G9G5_9BRAS	GO:0005739	
tr|I3S2A7|I3S2A7_LOTJA	GO:0005840	40s ribosomal protein s14-like in *Lotus japonicus*
tr|I3S2A7|I3S2A7_LOTJA	GO:0003735	
tr|I3S2A7|I3S2A7_LOTJA	GO:0006412	
tr|Q83XX0|Q83XX0_9MICC	GO:0003886	dna methyltransferase in *Arthrobacter*
tr|Q83XX0|Q83XX0_9MICC	GO:0003677	
tr|Q83XX0|Q83XX0_9MICC	GO:0009307	
tr|Q83XX0|Q83XX0_9MICC	GO:0090116	
tr|Q83XX0|Q83XX0_9MICC	EC:2.1.1.37	
tr|A0A024GZA8|A0A024GZA8_9MICC	GO:0055085	major facilitator transporter in *Arthrobacter siccitolerans*
tr|A0A024GZA8|A0A024GZA8_9MICC	GO:0016021	
tr|R0H2U3|R0H2U3_9BRAS	GO:0006346	probable inactive leucine-rich repeat receptor-like protein kinase at1g66830-like in *Capsella rubella*
tr|R0H2U3|R0H2U3_9BRAS	GO:0016246	
tr|R0H2U3|R0H2U3_9BRAS	GO:0031048	
tr|R0H2U3|R0H2U3_9BRAS	GO:0051567	
tr|R0H2U3|R0H2U3_9BRAS	GO:0009505	
tr|R0H2U3|R0H2U3_9BRAS	GO:0032440	
tr|R0H2U3|R0H2U3_9BRAS	GO:0048453	
tr|R0H2U3|R0H2U3_9BRAS	GO:0016021	
tr|R0H2U3|R0H2U3_9BRAS	GO:0007169	
tr|R0H2U3|R0H2U3_9BRAS	GO:0006468	

tr|R0H2U3|R0H2U3_9BRAS	GO:0004674	
tr|R0H2U3|R0H2U3_9BRAS	GO:0009570	
tr|R0H2U3|R0H2U3_9BRAS	GO:0048451	
tr|R0H2U3|R0H2U3_9BRAS	GO:0055114	
tr|R0H2U3|R0H2U3_9BRAS	GO:0005886	
tr|R0H2U3|R0H2U3_9BRAS	GO:0005524	
tr|R0H2U3|R0H2U3_9BRAS	GO:0005515	
tr|R0H2U3|R0H2U3_9BRAS	EC:2.7.11	
tr|R0H2U3|R0H2U3_9BRAS	EC:1.3.1.74	
tr|J1HF28|J1HF28_9ACTO	GO:0017001	penicillin-binding protein a in *Actinomyces georgiae*
tr|J1HF28|J1HF28_9ACTO	GO:0008658	
tr|J1HF28|J1HF28_9ACTO	GO:0008955	
tr|J1HF28|J1HF28_9ACTO	GO:0051301	
tr|J1HF28|J1HF28_9ACTO	GO:0008800	
tr|J1HF28|J1HF28_9ACTO	EC:2.4.1.129	
tr|J1HF28|J1HF28_9ACTO	EC:3.5.2.6	
tr|A0A0F8WI72|A0A0F8WI72_9EURO	GO:0045122	polyketide synthase in *Aspergillus rambellii*
tr|A0A0F8WI72|A0A0F8WI72_9EURO	GO:0016788	
tr|A0A0F8WI72|A0A0F8WI72_9EURO	GO:1900815	
tr|A0A0F8WI72|A0A0F8WI72_9EURO	GO:1900584	
tr|A0A0F8WI72|A0A0F8WI72_9EURO	GO:1900557	
tr|A0A0F8WI72|A0A0F8WI72_9EURO	GO:0045461	
tr|A0A0F8WI72|A0A0F8WI72_9EURO	GO:0016746	
tr|A0A0F8WI72|A0A0F8WI72_9EURO	GO:0031177	
tr|S6P2P9|S6P2P9_PSESF	GO:0035556	diguanylate cyclase in *Pseudomonas syringae*
tr|S6P2P9|S6P2P9_PSESF	GO:0016849	
tr|S6P2P9|S6P2P9_PSESF	GO:0009190	
tr|A0A0A1Z132|A0A0A1Z132_PSEFL	GO:0003886	dna methyltransferase in *Pseudomonas fluorescens*
tr|A0A0A1Z132|A0A0A1Z132_PSEFL	GO:0003677	
tr|A0A0A1Z132|A0A0A1Z132_PSEFL	GO:0009307	
tr|A0A0A1Z132|A0A0A1Z132_PSEFL	GO:0090116	
tr|A0A0A1Z132|A0A0A1Z132_PSEFL	EC:2.1.1.37	
tr|H0JGX1|H0JGX1_9PSED	GO:0009103	serine threonine protein kinase in *Pseudomonas psychrotolerans*
tr|H0JGX1|H0JGX1_9PSED	GO:0016773	
tr|H0JGX1|H0JGX1_9PSED	GO:0016301	
tr|H0JGX1|H0JGX1_9PSED	GO:0005524	
tr|H0JGX1|H0JGX1_9PSED	GO:0016020	
tr|J3HGL5|J3HGL5_9PSED	GO:0016301	deoxynucleotide monophosphate kinase in *Pseudomonas*
tr|J3HGL5|J3HGL5_9PSED	GO:0016310	
tr|A1C3C7|A1C3C7_MYCTH	GO:0003746	elongation factor partial in *Mycobacterium thermoresistibile*
tr|A1C3C7|A1C3C7_MYCTH	GO:0055114	

tr|A1C3C7|A1C3C7_MYCTH	GO:0016829	
tr|A1C3C7|A1C3C7_MYCTH	GO:0006414	
tr|A1C3C7|A1C3C7_MYCTH	GO:0016491	
tr|A1C3C7|A1C3C7_MYCTH	GO:0006184	
tr|A1C3C7|A1C3C7_MYCTH	GO:0003924	
tr|A1C3C7|A1C3C7_MYCTH	GO:0005737	
tr|A1C3C7|A1C3C7_MYCTH	GO:0005525	
tr|A6CUG3|A6CUG3_9BACI	GO:0005737	serine hydroxymethyltransferase in *Bacillus*
tr|A6CUG3|A6CUG3_9BACI	GO:0030170	
tr|A6CUG3|A6CUG3_9BACI	GO:0035999	
tr|A6CUG3|A6CUG3_9BACI	GO:0032259	
tr|A6CUG3|A6CUG3_9BACI	GO:0019264	
tr|A6CUG3|A6CUG3_9BACI	GO:0008168	
tr|A6CUG3|A6CUG3_9BACI	GO:0004372	
tr|A6CUG3|A6CUG3_9BACI	EC:2.1.2.1	
tr|W9D2K2|W9D2K2_9ACTN	GO:0006629	membrane protein in *Frankia*
tr|F3G1S6|F3G1S6_PSESJ	GO:0016491	xenobiotic reductase in *Pseudomonas syringae*
tr|F3G1S6|F3G1S6_PSESJ	GO:0042178	
tr|F3G1S6|F3G1S6_PSESJ	GO:0055114	
tr|F3G1S6|F3G1S6_PSESJ	GO:0010181	
tr|A0A061JS92|A0A061JS92_PSEST	GO:0018577	catechol dioxygenase in *Pseudomonas stutzeri*
tr|A0A061JS92|A0A061JS92_PSEST	GO:0019439	
tr|A0A061JS92|A0A061JS92_PSEST	GO:0008198	
tr|A0A061JS92|A0A061JS92_PSEST	GO:0055114	
tr|A0A061JS92|A0A061JS92_PSEST	EC:1.13.11.2	
tr|A0A085CIB6|A0A085CIB6_ELIME	GO:0051539	aconitate hydratase 1 in *Pseudomonas stutzer*
tr|A0A085CIB6|A0A085CIB6_ELIME	GO:0052633	
tr|A0A085CIB6|A0A085CIB6_ELIME	GO:0046872	
tr|A0A085CIB6|A0A085CIB6_ELIME	GO:0008152	
tr|A0A085CIB6|A0A085CIB6_ELIME	GO:0003994	
tr|A0A085CIB6|A0A085CIB6_ELIME	GO:0047780	
tr|A0A085CIB6|A0A085CIB6_ELIME	EC:4.2.1.4	
tr|A0A085CIB6|A0A085CIB6_ELIME	EC:4.2.1.3	
tr|A0A028VDZ0|A0A028VDZ0_PSEPS	GO:0051287	udp-n-acetyl-d-glucosamine dehydrogenase in *Pseudomonas pseudoalcaligenes*
tr|A0A028VDZ0|A0A028VDZ0_PSEPS	GO:0003979	
tr|A0A028VDZ0|A0A028VDZ0_PSEPS	GO:0055114	
tr|A0A028VDZ0|A0A028VDZ0_PSEPS	GO:0000271	
tr|A0A028VDZ0|A0A028VDZ0_PSEPS	GO:0016628	
tr|A0A028VDZ0|A0A028VDZ0_PSEPS	EC:1.1.1.22	
tr|A0A0D8BBH4|A0A0D8BBH4_9ACTN	GO:0006950	chemical-damaging agent resistance protein c in *Frankia*
tr|W9NFV5|W9NFV5_FUSOX	GO:0006810	atpase in *Fusarium oxysporum*

tr|W9NFV5|W9NFV5_FUSOX	GO:0016021	
tr|W9NFV5|W9NFV5_FUSOX	GO:0042626	
tr|W9NFV5|W9NFV5_FUSOX	GO:0006200	
tr|W9NFV5|W9NFV5_FUSOX	GO:0005524	
tr|A0A074TM82|A0A074TM82_9MICO	GO:0003676	lysyl-trna synthetase in *Microbacterium*
tr|A0A074TM82|A0A074TM82_9MICO	GO:0004824	
tr|A0A074TM82|A0A074TM82_9MICO	GO:0005524	
tr|A0A074TM82|A0A074TM82_9MICO	GO:0000287	
tr|A0A074TM82|A0A074TM82_9MICO	GO:0006430	
tr|A0A074TM82|A0A074TM82_9MICO	GO:0005737	
tr|A0A074TM82|A0A074TM82_9MICO	EC:6.1.1.6	
tr|R0HRK1|R0HRK1_9BRAS	GO:0005576	polygalacturonase in *Capsella rubella*
tr|R0HRK1|R0HRK1_9BRAS	GO:0004650	
tr|R0HRK1|R0HRK1_9BRAS	GO:0005975	
tr|R0HRK1|R0HRK1_9BRAS	GO:0047911	
tr|R0HRK1|R0HRK1_9BRAS	EC:3.2.1.67	
tr|R0HRK1|R0HRK1_9BRAS	EC:3.2.1.15	
tr|L1LRP3|L1LRP3_PSEPU	GO:0055085	secretion protein in *Pseudomonas putida*
tr|L1LRP3|L1LRP3_PSEPU	GO:0016020	
tr|I2FG13|I2FG13_9PSED	GO:0003676	integrase in *Pseudomonas*
tr|I2FG13|I2FG13_9PSED	GO:0000166	
tr|I2FG13|I2FG13_9PSED	GO:0015074	
tr|A0A099CG66|A0A099CG66_9MYCO	GO:0004519	hypothetical protein in *Mycobacterium rufum*
tr|A0A099CG66|A0A099CG66_9MYCO	GO:0006298	
tr|E8ND94|E8ND94_MICTS	GO:0005975	beta-glucosidase in *Microbacterium testaceum*
tr|E8ND94|E8ND94_MICTS	GO:0004553	
tr|G8X7L2|G8X7L2_FLACA	GO:0006457	trigger factor in *Flavobacterium columnare*
tr|G8X7L2|G8X7L2_FLACA	GO:0015031	
tr|G8X7L2|G8X7L2_FLACA	GO:0016853	
tr|A0A086WCF6|A0A086WCF6_9BURK	GO:0003700	family transcription regulator in *Massilia consociata*
tr|A0A086WCF6|A0A086WCF6_9BURK	GO:0003677	
tr|A0A086WCF6|A0A086WCF6_9BURK	GO:0008643	
tr|A0A086WCF6|A0A086WCF6_9BURK	GO:0006355	
tr|A0A0F2R7M9|A0A0F2R7M9_9PSED	GO:0000155	sensory box histidine kinase in *Pseudomonas*
tr|A0A0F2R7M9|A0A0F2R7M9_9PSED	GO:0023014	
tr|A0A0F2R7M9|A0A0F2R7M9_9PSED	GO:0000160	
tr|A0A0F2R7M9|A0A0F2R7M9_9PSED	GO:0005524	
tr|A0A0F2R7M9|A0A0F2R7M9_9PSED	GO:0016020	
tr|A0A0F2R7M9|A0A0F2R7M9_9PSED	EC:2.7.3	
tr|Q53D73|Q53D73_LOTJA	GO:0046872	superoxide dismutase in *Lotus japonicus*
tr|Q53D73|Q53D73_LOTJA	GO:0006801	

tr|Q53D73|Q53D73_LOTJA	GO:0004784	
tr|Q53D73|Q53D73_LOTJA	GO:0055114	
tr|Q53D73|Q53D73_LOTJA	EC:1.15.1.1	
tr|N0AYG0|N0AYG0_9BACI	GO:0050660	sulfite reductase subunit alpha
tr|N0AYG0|N0AYG0_9BACI	GO:0070814	
tr|N0AYG0|N0AYG0_9BACI	GO:0005506	
tr|N0AYG0|N0AYG0_9BACI	GO:0010181	
tr|N0AYG0|N0AYG0_9BACI	GO:0019344	
tr|N0AYG0|N0AYG0_9BACI	GO:0000103	
tr|N0AYG0|N0AYG0_9BACI	GO:0004783	
tr|N0AYG0|N0AYG0_9BACI	GO:0022900	
tr|N0AYG0|N0AYG0_9BACI	EC:1.8.1.2	
tr|D8PDK9|D8PDK9_9BACT	GO:0005524	dna repair protein in *Bacillus*
tr|D8PDK9|D8PDK9_9BACT	GO:0006310	
tr|D8PDK9|D8PDK9_9BACT	GO:0006281	
tr|A0A096PFI9|A0A096PFI9_9HYPO	GO:0005515	ankyrin repeat protein in *Fusarium acuminatum*
tr|A0A096PFI9|A0A096PFI9_9HYPO	GO:0009116	
tr|A0A096PFI9|A0A096PFI9_9HYPO	GO:0003824	
tr|E4TCS5|E4TCS5_RIEAD	GO:0046872	superoxide dismutase in *Riemerella anatipestifer*
tr|E4TCS5|E4TCS5_RIEAD	GO:0006801	
tr|E4TCS5|E4TCS5_RIEAD	GO:0004784	
tr|E4TCS5|E4TCS5_RIEAD	GO:0055114	
tr|E4TCS5|E4TCS5_RIEAD	EC:1.15.1.1	
tr|A0A015I5U3|A0A015I5U3_9GLOM	GO:0005730	nucleolar atpase in *Rhizophagus irregularis*
tr|A0A015I5U3|A0A015I5U3_9GLOM	GO:0030686	
tr|A0A015I5U3|A0A015I5U3_9GLOM	GO:0042274	
tr|A0A015I5U3|A0A015I5U3_9GLOM	GO:0008080	
tr|A0A0C7CQZ9|A0A0C7CQZ9_PSEAI	GO:0004176	peptidase in *Pseudomonas aeruginosa*
tr|A0A0C7CQZ9|A0A0C7CQZ9_PSEAI	GO:0006508	
tr|A0A0C7CQZ9|A0A0C7CQZ9_PSEAI	GO:0004252	
tr|A0A0C7CQZ9|A0A0C7CQZ9_PSEAI	EC:3.4.21	
tr|D2B009|D2B009_STRRD	GO:0009607	glycerol-3-phosphate responsive antiterminator in *Streptosporangium roseum*
tr|D2B009|D2B009_STRRD	GO:0006355	
tr|A0A0D1AYE2|A0A0D1AYE2_9MICC	GO:0008233	transglutaminase in *Arthrobacter*
tr|A0A0D1AYE2|A0A0D1AYE2_9MICC	GO:0006508	
tr|A0A0F8V3G8|A0A0F8V3G8_9EURO	GO:0006097	malate synthase in *Aspergillus rambellii*
tr|A0A0F8V3G8|A0A0F8V3G8_9EURO	GO:0045733	
tr|A0A0F8V3G8|A0A0F8V3G8_9EURO	GO:0009062	
tr|A0A0F8V3G8|A0A0F8V3G8_9EURO	GO:0004474	
tr|A0A0F8V3G8|A0A0F8V3G8_9EURO	GO:0006099	
tr|A0A0F8V3G8|A0A0F8V3G8_9EURO	GO:0015976	

tr|A0A0F8V3G8|A0A0F8V3G8_9EURO	GO:0005782	
tr|A0A0F8V3G8|A0A0F8V3G8_9EURO	GO:0009514	
tr|A0A0F8V3G8|A0A0F8V3G8_9EURO	GO:0047776	
tr|A0A0F8V3G8|A0A0F8V3G8_9EURO	EC:2.3.3.9	
tr|A0A0F8V3G8|A0A0F8V3G8_9EURO	EC:4.1.3.22	
tr|U7DIU2|U7DIU2_PSEFL	GO:0017111	atp-dependent clp protease
tr|U7DIU2|U7DIU2_PSEFL	GO:0005524	
tr|U7DIU2|U7DIU2_PSEFL	GO:0006508	
tr|U7DIU2|U7DIU2_PSEFL	GO:0008233	
tr|U7DIU2|U7DIU2_PSEFL	EC:3.6.1.15	
tr|G9MQ72|G9MQ72_HYPVG	GO:0045454	aif-like mitochondrial oxidoreductase in *Hypocrea virens*
tr|G9MQ72|G9MQ72_HYPVG	GO:0050660	
tr|G9MQ72|G9MQ72_HYPVG	GO:0046872	
tr|G9MQ72|G9MQ72_HYPVG	GO:0016491	
tr|G9MQ72|G9MQ72_HYPVG	GO:0051537	
tr|G9MQ72|G9MQ72_HYPVG	GO:0055114	
tr|A0A010SPQ5|A0A010SPQ5_PSEFL	GO:0016021	permease in *Pseudomonas fluorescens*
tr|A0A010SPQ5|A0A010SPQ5_PSEFL	GO:0055085	
tr|A0A010SPQ5|A0A010SPQ5_PSEFL	GO:0043190	
tr|E1VSE9|E1VSE9_ARTAR	GO:0003887	atpase aaa in *Arthrobacter arilaitensis*
tr|E1VSE9|E1VSE9_ARTAR	GO:0006260	
tr|E1VSE9|E1VSE9_ARTAR	GO:0003677	
tr|E1VSE9|E1VSE9_ARTAR	GO:0009360	
tr|E1VSE9|E1VSE9_ARTAR	GO:0005524	
tr|E1VSE9|E1VSE9_ARTAR	GO:0017111	
tr|E1VSE9|E1VSE9_ARTAR	EC:2.7.7.7	
tr|E1VSE9|E1VSE9_ARTAR	EC:3.6.1.15	
tr|A0A0F4SMF9|A0A0F4SMF9_PSEFL	GO:0005737	urocanate hydratase in *Pseudomonas fluorescens*
tr|A0A0F4SMF9|A0A0F4SMF9_PSEFL	GO:0019557	
tr|A0A0F4SMF9|A0A0F4SMF9_PSEFL	GO:0016153	
tr|A0A0F4SMF9|A0A0F4SMF9_PSEFL	GO:0019556	
tr|A0A0F4SMF9|A0A0F4SMF9_PSEFL	EC:4.2.1.49	
tr|A0A024M0C4|A0A024M0C4_9MYCO	GO:0004478	s-adenosylmethionine synthetase in *Mycobacterium farcinogenes*
tr|A0A024M0C4|A0A024M0C4_9MYCO	GO:0006556	
tr|A0A024M0C4|A0A024M0C4_9MYCO	GO:0006730	
tr|A0A024M0C4|A0A024M0C4_9MYCO	GO:0005524	
tr|A0A024M0C4|A0A024M0C4_9MYCO	GO:0000287	
tr|A0A024M0C4|A0A024M0C4_9MYCO	GO:0005737	
tr|A0A024M0C4|A0A024M0C4_9MYCO	EC:2.5.1.6	
tr|A0A0F4TJB0|A0A0F4TJB0_PSEFL	GO:0051903	s- glutathione dehydrogenase in *Pseudomonas fluorescens*
tr|A0A0F4TJB0|A0A0F4TJB0_PSEFL	GO:0008270	

tr|A0A0F4TJB0|A0A0F4TJB0_PSEFL	GO:0006069	
tr|A0A0F4TJB0|A0A0F4TJB0_PSEFL	GO:0004022	
tr|A0A0F4TJB0|A0A0F4TJB0_PSEFL	EC:1.1.1.284	
tr|A0A0F4TJB0|A0A0F4TJB0_PSEFL	EC:1.1.1.1	
tr|X8AV24|X8AV24_MYCXE	GO:0005829	s-adenosylmethionine synthetase in *Mycobacterium xenopi*
tr|X8AV24|X8AV24_MYCXE	GO:0005618	
tr|X8AV24|X8AV24_MYCXE	GO:0004478	
tr|X8AV24|X8AV24_MYCXE	GO:0040007	
tr|X8AV24|X8AV24_MYCXE	GO:0006556	
tr|X8AV24|X8AV24_MYCXE	GO:0006730	
tr|X8AV24|X8AV24_MYCXE	GO:0005524	
tr|X8AV24|X8AV24_MYCXE	GO:0000287	
tr|X8AV24|X8AV24_MYCXE	GO:0005886	
tr|X8AV24|X8AV24_MYCXE	EC:2.5.1.6	
tr|A0A0D0M7J5|A0A0D0M7J5_9FLAO	GO:0008270	carbonic anhydrase in *Flavobacterium*
tr|A0A0D0M7J5|A0A0D0M7J5_9FLAO	GO:0004089	
tr|A0A0D0M7J5|A0A0D0M7J5_9FLAO	EC:4.2.1.1	
tr|A0A097F8L6|A0A097F8L6_BEABA	GO:0005975	bacteriodes thetaiotaomicron symbiotic chitinase in *Beauveria bassiana*
tr|A0A097F8L6|A0A097F8L6_BEABA	GO:0004568	
tr|A0A097F8L6|A0A097F8L6_BEABA	GO:0006032	
tr|A0A097F8L6|A0A097F8L6_BEABA	EC:3.2.1.14	
tr|D5PB09|D5PB09_9MYCO	GO:0007059	secretion protein in *Mycobacterium parascrofulaceum*
tr|D5PB09|D5PB09_9MYCO	GO:0003677	
tr|D5PB09|D5PB09_9MYCO	GO:0007049	
tr|D5PB09|D5PB09_9MYCO	GO:0005524	
tr|D5PB09|D5PB09_9MYCO	GO:0016021	
tr|D5PB09|D5PB09_9MYCO	GO:0017111	
tr|D5PB09|D5PB09_9MYCO	GO:0051301	
tr|D5PB09|D5PB09_9MYCO	EC:3.6.1.15	
tr|R0FV13|R0FV13_9BRAS	GO:0005737	n-acetyl-l-glutamate synthase 1 in *Capsella rubella*
tr|R0FV13|R0FV13_9BRAS	GO:0006526	
tr|R0FV13|R0FV13_9BRAS	GO:0004042	
tr|R0FV13|R0FV13_9BRAS	EC:2.3.1.1	
tr|U9U829|U9U829_RHIID	GO:0009235	protein ubiquitin-60s ribosomal protein l40-like *in Rhizophagus irregularis*
tr|H9T851|H9T851_PLAOV	GO:0005730	
tr|H9T851|H9T851_PLAOV	GO:0042787	
tr|H9T851|H9T851_PLAOV	GO:0003735	
tr|H9T851|H9T851_PLAOV	GO:0022625	
tr|H9T851|H9T851_PLAOV	GO:0006412	
tr|H9T851|H9T851_PLAOV	GO:0005515	
tr|C3HDD4|C3HDD4_BACTU	GO:0019866	pts n-acetylglucosamine-specific iibc component in *Bacillus thuringiensis*

tr|C3HDD4|C3HDD4_BACTU	GO:0016301	
tr|C3HDD4|C3HDD4_BACTU	GO:0005886	
tr|C3HDD4|C3HDD4_BACTU	GO:0016310	
tr|C3HDD4|C3HDD4_BACTU	GO:0015764	
tr|C3HDD4|C3HDD4_BACTU	GO:0009401	
tr|C3HDD4|C3HDD4_BACTU	GO:0005351	
tr|C3HDD4|C3HDD4_BACTU	GO:0016021	
tr|C3HDD4|C3HDD4_BACTU	GO:0015572	
tr|C3HDD4|C3HDD4_BACTU	GO:0008982	
tr|C3HDD4|C3HDD4_BACTU	EC:2.7.1.69	
tr|R0HZ92|R0HZ92_9BRAS	GO:0005773	elongation factor ef-1 gamma subunit in *Capsella rubella*
tr|R0HZ92|R0HZ92_9BRAS	GO:0003746	
tr|R0HZ92|R0HZ92_9BRAS	GO:0005853	
tr|R0HZ92|R0HZ92_9BRAS	GO:0005507	
tr|R0HZ92|R0HZ92_9BRAS	GO:0005794	
tr|R0HZ92|R0HZ92_9BRAS	GO:0046686	
tr|R0HZ92|R0HZ92_9BRAS	GO:0009506	
tr|R0HZ92|R0HZ92_9BRAS	GO:0005886	
tr|R0HZ92|R0HZ92_9BRAS	GO:0010043	
tr|R0HZ92|R0HZ92_9BRAS	GO:0005618	
tr|R0HZ92|R0HZ92_9BRAS	GO:0005829	
tr|R0HZ92|R0HZ92_9BRAS	GO:0006414	
tr|R0HZ92|R0HZ92_9BRAS	GO:0005515	
tr|E6TXI0|E6TXI0_BACCJ	GO:0009358	polyphosphate kinase in *Bacillus cellulosilyticus*
tr|E6TXI0|E6TXI0_BACCJ	GO:0016310	
tr|E6TXI0|E6TXI0_BACCJ	GO:0005524	
tr|E6TXI0|E6TXI0_BACCJ	GO:0006799	
tr|E6TXI0|E6TXI0_BACCJ	GO:0008976	
tr|E6TXI0|E6TXI0_BACCJ	EC:2.7.4.1	
tr|K9FA32|K9FA32_PEND1	GO:0055085	mfs in *Penicillium digitatum*
tr|K9FA32|K9FA32_PEND1	GO:0016021	
tr|I3SSN9|I3SSN9_LOTJA	GO:0001510	60s ribosomal protein l26-1-like in *Lotus japonicus*
tr|I3SSN9|I3SSN9_LOTJA	GO:0006412	
tr|I3SSN9|I3SSN9_LOTJA	GO:0005730	
tr|I3SSN9|I3SSN9_LOTJA	GO:0009409	
tr|I3SSN9|I3SSN9_LOTJA	GO:0003735	
tr|I3SSN9|I3SSN9_LOTJA	GO:0005774	
tr|I3SSN9|I3SSN9_LOTJA	GO:0009507	
tr|I3SSN9|I3SSN9_LOTJA	GO:0022625	
tr|I3SSN9|I3SSN9_LOTJA	GO:0005794	
tr|I3SSN9|I3SSN9_LOTJA	GO:0005886	

tr|R0GQV3|R0GQV3_9BRAS	GO:0010286	alcohol dehydrogenase class iii in *Capsella rubella*
tr|R0GQV3|R0GQV3_9BRAS	GO:0080007	
tr|R0GQV3|R0GQV3_9BRAS	GO:0051903	
tr|R0GQV3|R0GQV3_9BRAS	GO:0008270	
tr|R0GQV3|R0GQV3_9BRAS	GO:0004022	
tr|R0GQV3|R0GQV3_9BRAS	GO:0000166	
tr|R0GQV3|R0GQV3_9BRAS	GO:0051049	
tr|R0GQV3|R0GQV3_9BRAS	GO:0005777	
tr|R0GQV3|R0GQV3_9BRAS	GO:0005829	
tr|R0GQV3|R0GQV3_9BRAS	GO:0019288	
tr|R0GQV3|R0GQV3_9BRAS	GO:0006069	
tr|R0GQV3|R0GQV3_9BRAS	GO:0046292	
tr|R0GQV3|R0GQV3_9BRAS	GO:0006569	
tr|R0GQV3|R0GQV3_9BRAS	GO:0008219	
tr|R0GQV3|R0GQV3_9BRAS	GO:0009684	
tr|R0GQV3|R0GQV3_9BRAS	GO:0048316	
tr|R0GQV3|R0GQV3_9BRAS	EC:1.1.1.284	
tr|R0GQV3|R0GQV3_9BRAS	EC:1.1.1.1	
tr|I3SU87|I3SU87_LOTJA	GO:0030554	cbs domain protein in *Lotus japonicus*
tr|I3SU87|I3SU87_LOTJA	GO:0005739	
tr|I3SU87|I3SU87_LOTJA	GO:0009651	
tr|I3SU87|I3SU87_LOTJA	GO:0050897	
tr|I3SU87|I3SU87_LOTJA	GO:0045454	
tr|I3SU87|I3SU87_LOTJA	GO:0009266	
tr|I3SU87|I3SU87_LOTJA	GO:0006972	
tr|I3SU87|I3SU87_LOTJA	GO:0006833	
tr|I3SU87|I3SU87_LOTJA	GO:0007030	
tr|I3SU87|I3SU87_LOTJA	GO:0006096	
tr|I3SU87|I3SU87_LOTJA	GO:0046686	
tr|R0IHA5|R0IHA5_9BRAS	GO:0001510	60s ribosomal protein l6 in *Capsella rubella*
tr|R0IHA5|R0IHA5_9BRAS	GO:0006412	
tr|R0IHA5|R0IHA5_9BRAS	GO:0005730	
tr|R0IHA5|R0IHA5_9BRAS	GO:0009506	
tr|R0IHA5|R0IHA5_9BRAS	GO:0003735	
tr|R0IHA5|R0IHA5_9BRAS	GO:0009507	
tr|R0IHA5|R0IHA5_9BRAS	GO:0022625	
tr|R0IHA5|R0IHA5_9BRAS	GO:0005783	
tr|R0IHA5|R0IHA5_9BRAS	GO:0005886	
tr|W8GN23|W8GN23_MYCTU	GO:0006091	enhanced green fluorescent protein in *Mycobacterium tuberculosis*
tr|W8GN23|W8GN23_MYCTU	GO:0008218	
tr|Q2PEP8|Q2PEP8_TRIPR	GO:0042026	rubisco subunit binding-protein alpha subunit in *Trifolium pratense*

tr|Q2PEP8|Q2PEP8_TRIPR	GO:0005739	
tr|Q2PEP8|Q2PEP8_TRIPR	GO:0016226	
tr|Q2PEP8|Q2PEP8_TRIPR	GO:0009941	
tr|Q2PEP8|Q2PEP8_TRIPR	GO:0009658	
tr|Q2PEP8|Q2PEP8_TRIPR	GO:0048481	
tr|Q2PEP8|Q2PEP8_TRIPR	GO:0009579	
tr|Q2PEP8|Q2PEP8_TRIPR	GO:0005524	
tr|Q2PEP8|Q2PEP8_TRIPR	GO:0009570	
tr|Q2PEP8|Q2PEP8_TRIPR	GO:0022626	
tr|Q2PEP8|Q2PEP8_TRIPR	GO:0016020	
tr|Q2PEP8|Q2PEP8_TRIPR	GO:0009793	
tr|Q2PEP8|Q2PEP8_TRIPR	GO:0048046	
tr|Q3LVN1|Q3LVN1_TAROF	GO:0006006	phosphoglucomutase phosphomannomutase family protein isoform 3 in *Taraxacum officinale*
tr|Q3LVN1|Q3LVN1_TAROF	GO:0005737	
tr|Q3LVN1|Q3LVN1_TAROF	GO:0000287	
tr|Q3LVN1|Q3LVN1_TAROF	GO:0004614	
tr|Q3LVN1|Q3LVN1_TAROF	EC:5.4.2.2	
tr|R0HEU4|R0HEU4_9BRAS	GO:0006013	glycosyl hydrolase family 38 protein in *Capsella rubella*
tr|R0HEU4|R0HEU4_9BRAS	GO:0008270	
tr|R0HEU4|R0HEU4_9BRAS	GO:0009505	
tr|R0HEU4|R0HEU4_9BRAS	GO:0005774	
tr|R0HEU4|R0HEU4_9BRAS	GO:0007020	
tr|R0HEU4|R0HEU4_9BRAS	GO:0019344	
tr|R0HEU4|R0HEU4_9BRAS	GO:0030246	
tr|R0HEU4|R0HEU4_9BRAS	GO:0004559	
tr|R0HEU4|R0HEU4_9BRAS	GO:0048046	
tr|R0HEU4|R0HEU4_9BRAS	EC:3.2.1.24	
tr|G3LKE9|G3LKE9_9BRAS	GO:0005774	at1g35720-like partial in *Capsella rubella*
tr|G3LKE9|G3LKE9_9BRAS	GO:0048046	
tr|G3LKE9|G3LKE9_9BRAS	GO:0016126	
tr|G3LKE9|G3LKE9_9BRAS	GO:0008270	
tr|G3LKE9|G3LKE9_9BRAS	GO:0030003	
tr|G3LKE9|G3LKE9_9BRAS	GO:0009651	
tr|G3LKE9|G3LKE9_9BRAS	GO:0006333	
tr|G3LKE9|G3LKE9_9BRAS	GO:0005509	
tr|G3LKE9|G3LKE9_9BRAS	GO:0005618	
tr|G3LKE9|G3LKE9_9BRAS	GO:0005829	
tr|G3LKE9|G3LKE9_9BRAS	GO:0007030	
tr|G3LKE9|G3LKE9_9BRAS	GO:0004601	
tr|G3LKE9|G3LKE9_9BRAS	GO:0009570	
tr|G3LKE9|G3LKE9_9BRAS	GO:0071435	

tr|G3LKE9|G3LKE9_9BRAS	GO:0005544	
tr|G3LKE9|G3LKE9_9BRAS	GO:0009579	
tr|G3LKE9|G3LKE9_9BRAS	GO:0046686	
tr|G3LKE9|G3LKE9_9BRAS	GO:0009409	
tr|G3LKE9|G3LKE9_9BRAS	GO:0019344	
tr|G3LKE9|G3LKE9_9BRAS	GO:0009408	
tr|G3LKE9|G3LKE9_9BRAS	GO:0009737	
tr|G3LKE9|G3LKE9_9BRAS	GO:0009506	
tr|G3LKE9|G3LKE9_9BRAS	GO:0070588	
tr|G3LKE9|G3LKE9_9BRAS	GO:0042803	
tr|G3LKE9|G3LKE9_9BRAS	GO:0005886	
tr|G3LKE9|G3LKE9_9BRAS	GO:0005507	
tr|G3LKE9|G3LKE9_9BRAS	GO:0005634	
tr|G3LKE9|G3LKE9_9BRAS	GO:0005524	
tr|G3LKE9|G3LKE9_9BRAS	GO:0006979	
tr|G3LKE9|G3LKE9_9BRAS	GO:0009269	
tr|G3LKE9|G3LKE9_9BRAS	GO:0005739	
tr|G3LKE9|G3LKE9_9BRAS	EC:1.11.1.7	
tr|H9BPH6|H9BPH6_VICFA	GO:0006952	pathogenesis-related protein pr10 in *Vicia faba*
tr|H9BPH6|H9BPH6_VICFA	GO:0009607	
tr|H9BPH6|H9BPH6_VICFA	GO:0005737	
tr|I3T7W2|I3T7W2_LOTJA	GO:0006144	uricase (nod-35) in *Lotus japonicus*
tr|I3T7W2|I3T7W2_LOTJA	GO:0019628	
tr|I3T7W2|I3T7W2_LOTJA	GO:0005777	
tr|I3T7W2|I3T7W2_LOTJA	GO:0009877	
tr|I3T7W2|I3T7W2_LOTJA	GO:0004846	
tr|I3T7W2|I3T7W2_LOTJA	GO:0055114	
tr|I3T7W2|I3T7W2_LOTJA	EC:1.7.3.3	
tr|H9T7Q2|H9T7Q2_QUESU	GO:0005730	profilin 3 family protein in *Quercus suber*
tr|H9T7Q2|H9T7Q2_QUESU	GO:0009524	
tr|H9T7Q2|H9T7Q2_QUESU	GO:0003779	
tr|H9T7Q2|H9T7Q2_QUESU	GO:0005886	
tr|H9T7Q2|H9T7Q2_QUESU	GO:0009826	
tr|H9T7Q2|H9T7Q2_QUESU	GO:0005618	
tr|H9T7Q2|H9T7Q2_QUESU	GO:0005819	
tr|H9T7Q2|H9T7Q2_QUESU	GO:0009507	
tr|H9T7Q2|H9T7Q2_QUESU	GO:0008154	
tr|R0GR76|R0GR76_9BRAS	GO:0005737	20s proteasome beta subunit e1 in *Capsella rubella*
tr|R0GR76|R0GR76_9BRAS	GO:0005839	
tr|R0GR76|R0GR76_9BRAS	GO:0046686	
tr|R0GR76|R0GR76_9BRAS	GO:0006511	

tr|R0GR76|R0GR76_9BRAS	GO:0005634	
tr|R0GR76|R0GR76_9BRAS	GO:0004298	
tr|R0GR76|R0GR76_9BRAS	EC:3.4.25	
tr|J7LJ06|J7LJ06_9MICC	GO:0004352	glutamate dehydrogenase in *Arthrobacter*
tr|J7LJ06|J7LJ06_9MICC	GO:0055114	
tr|J7LJ06|J7LJ06_9MICC	GO:0019551	
tr|J7LJ06|J7LJ06_9MICC	EC:1.4.1.2	
tr|Q0RK08|Q0RK08_FRAAA	GO:0006200	antibiotic abc transporter atp-binding protein in *Frankia alni*
tr|Q0RK08|Q0RK08_FRAAA	GO:0005524	
tr|Q0RK08|Q0RK08_FRAAA	GO:0016887	
tr|Q0RK08|Q0RK08_FRAAA	EC:3.6.1.3	
tr|Q1AVY1|Q1AVY1_RUBXD	GO:0004735	pyrroline-5-carboxylate reductase in *Rubrobacter xylanophilus*
tr|Q1AVY1|Q1AVY1_RUBXD	GO:0006561	
tr|Q1AVY1|Q1AVY1_RUBXD	GO:0055114	
tr|Q1AVY1|Q1AVY1_RUBXD	EC:1.5.1.2	
tr|Q9MB61|Q9MB61_ASTSI	GO:0070981	asparagine synthetase in *Astragalus sinicus*
tr|Q9MB61|Q9MB61_ASTSI	GO:0004066	
tr|Q9MB61|Q9MB61_ASTSI	GO:0006541	
tr|Q9MB61|Q9MB61_ASTSI	GO:0005524	
tr|Q9MB61|Q9MB61_ASTSI	EC:6.3.5.4	
tr|J1SXQ9|J1SXQ9_9DELT	GO:0016779	elongation factor tu
tr|J1SXQ9|J1SXQ9_9DELT	GO:0003746	
tr|J1SXQ9|J1SXQ9_9DELT	GO:0006414	
tr|J1SXQ9|J1SXQ9_9DELT	GO:0006184	
tr|J1SXQ9|J1SXQ9_9DELT	GO:0003924	
tr|J1SXQ9|J1SXQ9_9DELT	GO:0005737	
tr|J1SXQ9|J1SXQ9_9DELT	GO:0005525	
tr|D8PD20|D8PD20_9BACT	GO:0005737	molecular chaperone in *Nitrospira defluvii*
tr|D8PD20|D8PD20_9BACT	GO:0006457	
tr|D8PD20|D8PD20_9BACT	GO:0051087	
tr|D8PD20|D8PD20_9BACT	GO:0005524	
tr|I3SQF0|I3SQF0_LOTJA	GO:0005730	40s ribosomal protein s19-1-like in *Lotus japonicus*
tr|I3SQF0|I3SQF0_LOTJA	GO:0005773	
tr|I3SQF0|I3SQF0_LOTJA	GO:0003735	
tr|I3SQF0|I3SQF0_LOTJA	GO:0005618	
tr|I3SQF0|I3SQF0_LOTJA	GO:0022627	
tr|I3SQF0|I3SQF0_LOTJA	GO:0016020	
tr|I3SQF0|I3SQF0_LOTJA	GO:0006412	
tr|Q43781|Q43781_LOTJA	GO:0004069	aspartate aminotransferase in *Lotus japonicus*
tr|Q43781|Q43781_LOTJA	GO:0051788	
tr|Q43781|Q43781_LOTJA	GO:0006635	

tr|Q43781|Q43781_LOTJA	GO:0009536	
tr|Q43781|Q43781_LOTJA	GO:0043161	
tr|Q43781|Q43781_LOTJA	GO:0009693	
tr|Q43781|Q43781_LOTJA	GO:0009407	
tr|Q43781|Q43781_LOTJA	GO:0080129	
tr|Q43781|Q43781_LOTJA	GO:0005618	
tr|Q43781|Q43781_LOTJA	GO:0048767	
tr|Q43781|Q43781_LOTJA	GO:0005777	
tr|Q43781|Q43781_LOTJA	GO:0005829	
tr|Q43781|Q43781_LOTJA	GO:0080130	
tr|Q43781|Q43781_LOTJA	GO:0030170	
tr|Q43781|Q43781_LOTJA	GO:0006520	
tr|Q43781|Q43781_LOTJA	GO:0009735	
tr|Q43781|Q43781_LOTJA	GO:0005886	
tr|Q43781|Q43781_LOTJA	GO:0005507	
tr|Q43781|Q43781_LOTJA	EC:2.6.1.9	
tr|Q43781|Q43781_LOTJA	EC:2.6.1.57	
tr|Q43781|Q43781_LOTJA	EC:2.6.1.5	
tr|Q43781|Q43781_LOTJA	EC:2.6.1.1	
tr|W9MDE0|W9MDE0_FUSOX	GO:0016236	adp-ribosylation factor in *Fusarium oxysporum*
tr|W9MDE0|W9MDE0_FUSOX	GO:0004871	
tr|W9MDE0|W9MDE0_FUSOX	GO:0007264	
tr|W9MDE0|W9MDE0_FUSOX	GO:0006888	
tr|W9MDE0|W9MDE0_FUSOX	GO:0006893	
tr|W9MDE0|W9MDE0_FUSOX	GO:0005794	
tr|W9MDE0|W9MDE0_FUSOX	GO:0005525	
tr|W9MDE0|W9MDE0_FUSOX	GO:0006886	
tr|A0A0A8JM98|A0A0A8JM98_BACSX	GO:0006457	molecular chaperone in *Bacillus*
tr|A0A0A8JM98|A0A0A8JM98_BACSX	GO:0016491	
tr|A0A0A8JM98|A0A0A8JM98_BACSX	GO:0006950	
tr|A0A0A8JM98|A0A0A8JM98_BACSX	GO:0005524	
tr|A0A0A8JM98|A0A0A8JM98_BACSX	GO:0051082	
tr|A0A0A8JM98|A0A0A8JM98_BACSX	GO:0055114	
tr|A0A051TRF4|A0A051TRF4_MYCTX	GO:0006098	6-phosphogluconate dehydrogenase in *Mycobacterium tuberculosis*
tr|A0A051TRF4|A0A051TRF4_MYCTX	GO:0004616	
tr|A0A051TRF4|A0A051TRF4_MYCTX	GO:0051287	
tr|A0A051TRF4|A0A051TRF4_MYCTX	EC:1.1.1.44	
tr|A0A0F7BPC5|A0A0F7BPC5_MYXFU	GO:0003848	2-amino-4-hydroxy-6-hydroxymethyldihydropteridine pyrophosphokinase in *Myxococcus fulvus*
tr|A0A0F7BPC5|A0A0F7BPC5_MYXFU	GO:0009396	
tr|A0A0F7BPC5|A0A0F7BPC5_MYXFU	GO:0016301	
tr|A0A0F7BPC5|A0A0F7BPC5_MYXFU	GO:0016310	

tr|A0A0F7BPC5|A0A0F7BPC5_MYXFU	EC:2.7.6.3	
tr|I3SLT0|I3SLT0_LOTJA	GO:0006412	60s ribosomal protein l9-like in *Lotus japonicus*
tr|I3SLT0|I3SLT0_LOTJA	GO:0009955	
tr|I3SLT0|I3SLT0_LOTJA	GO:0005730	
tr|I3SLT0|I3SLT0_LOTJA	GO:0019843	
tr|I3SLT0|I3SLT0_LOTJA	GO:0003735	
tr|I3SLT0|I3SLT0_LOTJA	GO:0009941	
tr|I3SLT0|I3SLT0_LOTJA	GO:0005773	
tr|I3SLT0|I3SLT0_LOTJA	GO:0022625	
tr|I3SLT0|I3SLT0_LOTJA	GO:0005886	
tr|Q0CHN8|Q0CHN8_ASPTN	GO:0006468	serine threonine protein kinase in *Aspergillus terreus*
tr|Q0CHN8|Q0CHN8_ASPTN	GO:0005524	
tr|Q0CHN8|Q0CHN8_ASPTN	GO:0004674	
tr|Q0CHN8|Q0CHN8_ASPTN	EC:2.7.11	
tr|J3E9X9|J3E9X9_9PSED	GO:0006200	arabinose abc transporter atp-binding protein in *Pseudomonas*
tr|J3E9X9|J3E9X9_9PSED	GO:0042882	
tr|J3E9X9|J3E9X9_9PSED	GO:0005524	
tr|J3E9X9|J3E9X9_9PSED	GO:0015612	
tr|J3E9X9|J3E9X9_9PSED	GO:0005886	
tr|G9QLS3|G9QLS3_9BACI	GO:0016788	s1 rna-binding domain-containing protein in *Bacillus smithii*
tr|G9QLS3|G9QLS3_9BACI	GO:0006281	
tr|G9QLS3|G9QLS3_9BACI	GO:0003723	
tr|G9QLS3|G9QLS3_9BACI	GO:0003677	
tr|A0A0D0GRZ7|A0A0D0GRZ7_9SPHI	GO:0005975	glycoside hydrolase family 3 in *Pedobacter*
tr|A0A0D0GRZ7|A0A0D0GRZ7_9SPHI	GO:0004553	
tr|A0A0A8JM46|A0A0A8JM46_BACSX	GO:0005737	thiouridylase in *Bacillus*
tr|A0A0A8JM46|A0A0A8JM46_BACSX	GO:0006400	
tr|A0A0A8JM46|A0A0A8JM46_BACSX	GO:0005524	
tr|A0A0A8JM46|A0A0A8JM46_BACSX	GO:0032259	
tr|A0A0A8JM46|A0A0A8JM46_BACSX	GO:0016783	
tr|A0A0A8JM46|A0A0A8JM46_BACSX	GO:0008168	
tr|A0A0A8JM46|A0A0A8JM46_BACSX	GO:0000049	
tr|G9N9Q5|G9N9Q5_HYPVG	GO:0030001	hypothetical protein TRIVIDRAFT_40355 in *Hypocrea virens*
tr|G9N9Q5|G9N9Q5_HYPVG	GO:0016020	
tr|G9N9Q5|G9N9Q5_HYPVG	GO:0046873	
tr|G9N9Q5|G9N9Q5_HYPVG	GO:0055085	
tr|A0A0A8JEH9|A0A0A8JEH9_BACSX	GO:0006184	gtp-binding protein in *Bacillus*
tr|A0A0A8JEH9|A0A0A8JEH9_BACSX	GO:0003924	
tr|A0A0A8JEH9|A0A0A8JEH9_BACSX	GO:0005737	
tr|A0A0A8JEH9|A0A0A8JEH9_BACSX	GO:0005525	
tr|R0IFZ3|R0IFZ3_9BRAS	GO:0005829	dehydroascorbate reductase in *Capsella rubella*

tr|R0IFZ3|R0IFZ3_9BRAS	GO:0005507	
tr|R0IFZ3|R0IFZ3_9BRAS	GO:0009753	
tr|R0IFZ3|R0IFZ3_9BRAS	GO:0010193	
tr|R0IFZ3|R0IFZ3_9BRAS	GO:0055114	
tr|R0IFZ3|R0IFZ3_9BRAS	GO:0005777	
tr|R0IFZ3|R0IFZ3_9BRAS	GO:0005739	
tr|R0IFZ3|R0IFZ3_9BRAS	GO:0009610	
tr|R0IFZ3|R0IFZ3_9BRAS	GO:0045174	
tr|R0IFZ3|R0IFZ3_9BRAS	GO:0016740	
tr|R0IFZ3|R0IFZ3_9BRAS	GO:0009570	
tr|R0IFZ3|R0IFZ3_9BRAS	GO:0043903	
tr|R0IFZ3|R0IFZ3_9BRAS	GO:0010583	
tr|R0IFZ3|R0IFZ3_9BRAS	GO:0043295	
tr|R0IFZ3|R0IFZ3_9BRAS	GO:0010043	
tr|R0IFZ3|R0IFZ3_9BRAS	GO:0010731	
tr|R0IFZ3|R0IFZ3_9BRAS	GO:0005773	
tr|R0IFZ3|R0IFZ3_9BRAS	GO:0009407	
tr|R0IFZ3|R0IFZ3_9BRAS	GO:0005886	
tr|R0IFZ3|R0IFZ3_9BRAS	GO:0048046	
tr|R0IFZ3|R0IFZ3_9BRAS	GO:0005515	
tr|R0IFZ3|R0IFZ3_9BRAS	EC:1.8.5.1	
tr|A0A0D6SZ14|A0A0D6SZ14_9PSED	GO:0016740	methyltransferase in *Pseudomonas*
tr|A0A0D6SZ14|A0A0D6SZ14_9PSED	GO:0052699	
tr|R0EZ20|R0EZ20_9BRAS	GO:0001510	60s ribosomal protein l14 in *Capsella rubella*
tr|R0EZ20|R0EZ20_9BRAS	GO:0006412	
tr|R0EZ20|R0EZ20_9BRAS	GO:0005730	
tr|R0EZ20|R0EZ20_9BRAS	GO:0009506	
tr|R0EZ20|R0EZ20_9BRAS	GO:0003735	
tr|R0EZ20|R0EZ20_9BRAS	GO:0005774	
tr|R0EZ20|R0EZ20_9BRAS	GO:0042254	
tr|R0EZ20|R0EZ20_9BRAS	GO:0009507	
tr|R0EZ20|R0EZ20_9BRAS	GO:0022625	
tr|R0EZ20|R0EZ20_9BRAS	GO:0005794	
tr|R0EZ20|R0EZ20_9BRAS	GO:0005783	
tr|R0EZ20|R0EZ20_9BRAS	GO:0005886	
tr|A0A024M601|A0A024M601_9MYCO	GO:0006184	gtpase in *Mycobacterium farcinogenes*
tr|A0A024M601|A0A024M601_9MYCO	GO:0000287	
tr|A0A024M601|A0A024M601_9MYCO	GO:0003924	
tr|A0A024M601|A0A024M601_9MYCO	GO:0005737	
tr|A0A024M601|A0A024M601_9MYCO	GO:0005525	
tr|I3S8F0|I3S8F0_LOTJA	GO:0006301	ubiquitin-conjugating enzyme e2 36-like in *Lotus japonicus*

tr|I3S8F0|I3S8F0_LOTJA	GO:0005829	
tr|I3S8F0|I3S8F0_LOTJA	GO:0006511	
tr|I3S8F0|I3S8F0_LOTJA	GO:0010053	
tr|I3S8F0|I3S8F0_LOTJA	GO:0010039	
tr|I3S8F0|I3S8F0_LOTJA	GO:0004842	
tr|I3S8F0|I3S8F0_LOTJA	GO:0016567	
tr|I3S8F0|I3S8F0_LOTJA	GO:0005515	
tr|I3S8F0|I3S8F0_LOTJA	GO:0031372	
tr|I3S8F0|I3S8F0_LOTJA	GO:0016579	
tr|I3S8F0|I3S8F0_LOTJA	GO:0009507	
tr|I3S8F0|I3S8F0_LOTJA	GO:0046686	
tr|I3S8F0|I3S8F0_LOTJA	GO:0005634	
tr|I3S8F0|I3S8F0_LOTJA	GO:0005886	
tr|I3S8F0|I3S8F0_LOTJA	EC:6.3.2.19	
tr|A0A078MHP2|A0A078MHP2_9PSED	GO:0033014	uroporphyrinogen iii synthase hem4 in *Pseudomonas*
tr|A0A078MHP2|A0A078MHP2_9PSED	GO:0004852	
tr|I3TAM1|I3TAM1_LOTJA	GO:0003735	60s ribosomal protein l18 in *Lotus japonicus*
tr|I3TAM1|I3TAM1_LOTJA	GO:0022625	
tr|I3TAM1|I3TAM1_LOTJA	GO:0006412	
tr|R0F3J6|R0F3J6_9BRAS	GO:0001666	pyruvate decarboxylase in *Capsella rubella*
tr|R0F3J6|R0F3J6_9BRAS	GO:0005829	
tr|R0F3J6|R0F3J6_9BRAS	GO:0030976	
tr|R0F3J6|R0F3J6_9BRAS	GO:0009862	
tr|R0F3J6|R0F3J6_9BRAS	GO:0010498	
tr|R0F3J6|R0F3J6_9BRAS	GO:0007010	
tr|R0F3J6|R0F3J6_9BRAS	GO:0016740	
tr|R0F3J6|R0F3J6_9BRAS	GO:0000287	
tr|R0F3J6|R0F3J6_9BRAS	GO:0006094	
tr|R0F3J6|R0F3J6_9BRAS	GO:0009507	
tr|R0F3J6|R0F3J6_9BRAS	GO:0004737	
tr|R0F3J6|R0F3J6_9BRAS	GO:0010310	
tr|R0F3J6|R0F3J6_9BRAS	GO:0016020	
tr|R0F3J6|R0F3J6_9BRAS	EC:4.1.1.1	
tr|A0A0D9AZE0|A0A0D9AZE0_PSEFL	GO:0035556	transcriptional regulator in *Pseudomonas fluorescens*
tr|A0A0D9AZE0|A0A0D9AZE0_PSEFL	GO:0003700	
tr|A0A0D9AZE0|A0A0D9AZE0_PSEFL	GO:0000160	
tr|A0A0D9AZE0|A0A0D9AZE0_PSEFL	GO:0003677	
tr|A0A0D9AZE0|A0A0D9AZE0_PSEFL	GO:0006355	
tr|A0A0D9AZE0|A0A0D9AZE0_PSEFL	GO:0000156	
tr|A0A0F4TTX1|A0A0F4TTX1_PSEFL	GO:0097264	sugar-binding protein in *Pseudomonas fluorescens*
tr|S0DX72|S0DX72_GIBF5	GO:0005524	ubiquitin-like 1-activating enzyme e1 b in *Gibberella fujikuroi*

tr|S0DX72|S0DX72_GIBF5	GO:0016925	
tr|S0DX72|S0DX72_GIBF5	GO:0019948	
sp|P52416|GLGS1_VICFA	GO:0010155	adp-glucose pyrophosphorylase in *Vicia faba*
sp|P52416|GLGS1_VICFA	GO:0006098	
sp|P52416|GLGS1_VICFA	GO:0009853	
sp|P52416|GLGS1_VICFA	GO:0010218	
sp|P52416|GLGS1_VICFA	GO:0048046	
sp|P52416|GLGS1_VICFA	GO:0009744	
sp|P52416|GLGS1_VICFA	GO:0030931	
sp|P52416|GLGS1_VICFA	GO:0043085	
sp|P52416|GLGS1_VICFA	GO:0009793	
sp|P52416|GLGS1_VICFA	GO:0019252	
sp|P52416|GLGS1_VICFA	GO:0010114	
sp|P52416|GLGS1_VICFA	GO:0000023	
sp|P52416|GLGS1_VICFA	GO:0048573	
sp|P52416|GLGS1_VICFA	GO:0008878	
sp|P52416|GLGS1_VICFA	GO:0016226	
sp|P52416|GLGS1_VICFA	GO:0019288	
sp|P52416|GLGS1_VICFA	GO:0009570	
sp|P52416|GLGS1_VICFA	GO:0009637	
sp|P52416|GLGS1_VICFA	GO:0005978	
sp|P52416|GLGS1_VICFA	GO:0009644	
sp|P52416|GLGS1_VICFA	GO:0019761	
sp|P52416|GLGS1_VICFA	GO:0005524	
sp|P52416|GLGS1_VICFA	GO:0048481	
sp|P52416|GLGS1_VICFA	GO:0010027	
sp|P52416|GLGS1_VICFA	EC:2.7.7.27	
tr|I3DWI3|I3DWI3_BACMT	GO:0006184	dynamin family protein in *Bacillus methanolicus*
tr|I3DWI3|I3DWI3_BACMT	GO:0003924	
tr|I3DWI3|I3DWI3_BACMT	GO:0005525	
tr|R0HN35|R0HN35_9BRAS	GO:0006833	40s ribosomal protein sa (laminin receptor-like protein) in *Capsella rubella*
tr|R0HN35|R0HN35_9BRAS	GO:0006412	
tr|R0HN35|R0HN35_9BRAS	GO:0000028	
tr|R0HN35|R0HN35_9BRAS	GO:0001510	
tr|R0HN35|R0HN35_9BRAS	GO:0009664	
tr|R0HN35|R0HN35_9BRAS	GO:0000447	
tr|R0HN35|R0HN35_9BRAS	GO:0009651	
tr|R0HN35|R0HN35_9BRAS	GO:0009507	
tr|R0HN35|R0HN35_9BRAS	GO:0005794	
tr|R0HN35|R0HN35_9BRAS	GO:0006972	
tr|R0HN35|R0HN35_9BRAS	GO:0007030	

tr|R0HN35|R0HN35_9BRAS	GO:0000461	
tr|R0HN35|R0HN35_9BRAS	GO:0006096	
tr|R0HN35|R0HN35_9BRAS	GO:0042256	
tr|R0HN35|R0HN35_9BRAS	GO:0046686	
tr|R0HN35|R0HN35_9BRAS	GO:0003735	
tr|R0HN35|R0HN35_9BRAS	GO:0009506	
tr|R0HN35|R0HN35_9BRAS	GO:0009266	
tr|R0HN35|R0HN35_9BRAS	GO:0030686	
tr|R0HN35|R0HN35_9BRAS	GO:0005886	
tr|R0HN35|R0HN35_9BRAS	GO:0006606	
tr|R0HN35|R0HN35_9BRAS	GO:0005634	
tr|R0HN35|R0HN35_9BRAS	GO:0042545	
tr|R0HN35|R0HN35_9BRAS	GO:0006407	
tr|R0HN35|R0HN35_9BRAS	GO:0022627	
tr|B2CBB3|B2CBB3_9POAL	GO:0005737	phosphoglucose isomerase in *Festuca ovina*
tr|B2CBB3|B2CBB3_9POAL	GO:0006094	
tr|B2CBB3|B2CBB3_9POAL	GO:0004347	
tr|B2CBB3|B2CBB3_9POAL	GO:0006096	
tr|B2CBB3|B2CBB3_9POAL	EC:5.3.1.9	
tr|U2XBN4|U2XBN4_9MICO	GO:0004672	abc transporter in *Microbacterium*
tr|U2XBN4|U2XBN4_9MICO	GO:0006468	
tr|U2XBN4|U2XBN4_9MICO	GO:0005524	
tr|F3HN82|F3HN82_PSEYM	GO:0003333	threonine serine transporter in *Pseudomonas syringae*
tr|A0A075EAM1|A0A075EAM1_ASTME	GO:0016132	acetyl- cytosolic in *Astragalus membranaceus*
tr|A0A075EAM1|A0A075EAM1_ASTME	GO:0006085	
tr|A0A075EAM1|A0A075EAM1_ASTME	GO:0005829	
tr|A0A075EAM1|A0A075EAM1_ASTME	GO:0003985	
tr|A0A075EAM1|A0A075EAM1_ASTME	GO:0005777	
tr|A0A075EAM1|A0A075EAM1_ASTME	GO:0009860	
tr|A0A075EAM1|A0A075EAM1_ASTME	GO:0009846	
tr|A0A075EAM1|A0A075EAM1_ASTME	GO:0019745	
tr|A0A075EAM1|A0A075EAM1_ASTME	GO:0005886	
tr|A0A075EAM1|A0A075EAM1_ASTME	GO:0009793	
tr|A0A075EAM1|A0A075EAM1_ASTME	GO:0016126	
tr|A0A075EAM1|A0A075EAM1_ASTME	EC:2.3.1.9	
tr|V7K184|V7K184_MYCAV	GO:0030170	glutamate decarboxylase in *Mycobacterium avium*
tr|V7K184|V7K184_MYCAV	GO:0006536	
tr|V7K184|V7K184_MYCAV	GO:0004351	
tr|V7K184|V7K184_MYCAV	EC:4.1.1.15	
tr|R0FUG6|R0FUG6_9BRAS	GO:0043687	nodule inception protein 8 in *Capsella rubella*
tr|R0FUG6|R0FUG6_9BRAS	GO:0003700	

tr|R0FUG6|R0FUG6_9BRAS	GO:0009688	
tr|R0FUG6|R0FUG6_9BRAS	GO:0045893	
tr|R0FUG6|R0FUG6_9BRAS	GO:0005634	
tr|R0FUG6|R0FUG6_9BRAS	GO:0005515	
tr|V7JIP4|V7JIP4_MYCPC	GO:0035556	adenylyl cyclase in *Mycobacterium avium*
tr|V7JIP4|V7JIP4_MYCPC	GO:0004871	
tr|V7JIP4|V7JIP4_MYCPC	GO:0016021	
tr|V7JIP4|V7JIP4_MYCPC	GO:0004016	
tr|V7JIP4|V7JIP4_MYCPC	GO:0006200	
tr|V7JIP4|V7JIP4_MYCPC	GO:0006171	
tr|V7JIP4|V7JIP4_MYCPC	GO:0006198	
tr|V7JIP4|V7JIP4_MYCPC	EC:4.6.1.1	
tr|A0A0D9NAR3|A0A0D9NAR3_ASPFL	GO:0008199	extracellular dioxygenase in *Aspergillus flavus*
tr|A0A0D9NAR3|A0A0D9NAR3_ASPFL	GO:0006725	
tr|A0A0D9NAR3|A0A0D9NAR3_ASPFL	GO:0016702	
tr|A0A0D9NAR3|A0A0D9NAR3_ASPFL	GO:0055114	
tr|A0A0D9NAR3|A0A0D9NAR3_ASPFL	EC:1.13.11	
tr|A0A0C3E2C5|A0A0C3E2C5_9HOMO	GO:0046961	atp synthase f1 alpha subunit in *Scleroderma citrinum*
tr|A0A0C3E2C5|A0A0C3E2C5_9HOMO	GO:0046933	
tr|A0A0C3E2C5|A0A0C3E2C5_9HOMO	GO:0015991	
tr|A0A0C3E2C5|A0A0C3E2C5_9HOMO	GO:0042645	
tr|A0A0C3E2C5|A0A0C3E2C5_9HOMO	GO:0005754	
tr|A0A0C3E2C5|A0A0C3E2C5_9HOMO	GO:0015986	
tr|A0A0C3E2C5|A0A0C3E2C5_9HOMO	GO:0006200	
tr|A0A0C3E2C5|A0A0C3E2C5_9HOMO	GO:0005524	
tr|A0A0A2J8X6|A0A0A2J8X6_PENEN	GO:0009987	nonribosomal peptide in *Penicillium expansum*
tr|A0A0A2J8X6|A0A0A2J8X6_PENEN	GO:0031177	
tr|A0A0A2J8X6|A0A0A2J8X6_PENEN	GO:0019748	
tr|A0A0A2J8X6|A0A0A2J8X6_PENEN	GO:0003824	
tr|R4UL46|R4UL46_MYCAB	GO:0032259	dna methyltransferase in *Mycobacterium abscessus*
tr|R4UL46|R4UL46_MYCAB	GO:0006139	
tr|R4UL46|R4UL46_MYCAB	GO:0008168	
tr|R4UL46|R4UL46_MYCAB	GO:0003676	
tr|G9MZ07|G9MZ07_HYPVG	GO:0000981	transcriptional regulatory protein moc3 in *Hypocrea virens*
tr|G9MZ07|G9MZ07_HYPVG	GO:0006357	
tr|G9MZ07|G9MZ07_HYPVG	GO:0005634	
tr|G9MZ07|G9MZ07_HYPVG	GO:0008270	
tr|T5KKJ6|T5KKJ6_9MICO	GO:0006200	abc transporter atp-binding protein in *Microbacterium maritypicum*
tr|T5KKJ6|T5KKJ6_9MICO	GO:0005524	
tr|T5KKJ6|T5KKJ6_9MICO	GO:0016887	
tr|T5KKJ6|T5KKJ6_9MICO	EC:3.6.1.3	

tr|A0A0C1CMS5|A0A0C1CMS5_9FLAO	GO:0000160	histidine kinase in *Flavobacterium*
tr|A0A0C1CMS5|A0A0C1CMS5_9FLAO	GO:0000156	
tr|A0A0C1CMS5|A0A0C1CMS5_9FLAO	GO:0035556	
tr|A0A0C1CMS5|A0A0C1CMS5_9FLAO	GO:0006355	
tr|A0A0C1CMS5|A0A0C1CMS5_9FLAO	GO:0023014	
tr|A0A0C1CMS5|A0A0C1CMS5_9FLAO	GO:0005524	
tr|A0A0C1CMS5|A0A0C1CMS5_9FLAO	GO:0016020	
tr|A0A0C1CMS5|A0A0C1CMS5_9FLAO	GO:0000155	
tr|A0A0C1CMS5|A0A0C1CMS5_9FLAO	GO:0005515	
tr|A0A0C1CMS5|A0A0C1CMS5_9FLAO	EC:2.7.3	
tr|A0A060LYD6|A0A060LYD6_9BACI	GO:0004852	uroporphyrinogen-iii synthase in *Bacillus lehensis*
tr|A0A060LYD6|A0A060LYD6_9BACI	GO:0033014	
tr|A0A0C1ENM9|A0A0C1ENM9_9FLAO	GO:0008658	penicillin-binding protein 2 in *Flavobacterium*
tr|A0A0C1ENM9|A0A0C1ENM9_9FLAO	GO:0009252	
tr|A0A0C1ENM9|A0A0C1ENM9_9FLAO	GO:0008955	
tr|A0A0C1ENM9|A0A0C1ENM9_9FLAO	GO:0016020	
tr|A0A0C1ENM9|A0A0C1ENM9_9FLAO	EC:2.4.1.129	
tr|A0A0A1Z4D5|A0A0A1Z4D5_PSEFL	GO:0035556	family transcriptional regulator in *Pseudomonas fluorescens*
tr|A0A0A1Z4D5|A0A0A1Z4D5_PSEFL	GO:0003700	
tr|A0A0A1Z4D5|A0A0A1Z4D5_PSEFL	GO:0000160	
tr|A0A0A1Z4D5|A0A0A1Z4D5_PSEFL	GO:0003677	
tr|A0A0A1Z4D5|A0A0A1Z4D5_PSEFL	GO:0006355	
tr|A0A0A1Z4D5|A0A0A1Z4D5_PSEFL	GO:0000156	
tr|C9SAY9|C9SAY9_VERA1	GO:0030915	nuclear protein qri2 in *Verticillium alfalfae*
tr|C9SAY9|C9SAY9_VERA1	GO:0016020	
tr|C9SAY9|C9SAY9_VERA1	GO:0005634	
tr|C9SAY9|C9SAY9_VERA1	GO:0006281	
tr|A0A0F8UYB0|A0A0F8UYB0_9EURO	GO:0009411	20s cyclosome subunit (apc1) in *Aspergillus rambellii*
tr|A0A0F8UYB0|A0A0F8UYB0_9EURO	GO:0051301	
tr|A0A0F8UYB0|A0A0F8UYB0_9EURO	GO:0031145	
tr|A0A0F8UYB0|A0A0F8UYB0_9EURO	GO:0045839	
tr|A0A0F8UYB0|A0A0F8UYB0_9EURO	GO:0005680	
tr|Q9F0I1|Q9F0I1_9PSED	GO:0003840	gamma-glutamyltransferase in *Pseudomonas chlororaphis*
tr|Q9F0I1|Q9F0I1_9PSED	GO:0006749	
tr|Q9F0I1|Q9F0I1_9PSED	EC:2.3.2.2	
tr|A0A0E8P0D6|A0A0E8P0D6_MYCTX	GO:0006200	multidrug abc transporter atp-binding protein in *Mycobacterium tuberculosis*
tr|A0A0E8P0D6|A0A0E8P0D6_MYCTX	GO:0005524	
tr|A0A0E8P0D6|A0A0E8P0D6_MYCTX	GO:0016887	
tr|A0A0E8P0D6|A0A0E8P0D6_MYCTX	EC:3.6.1.3	

Supplementary Table S4: Pathways identified by KEGG analysis

Pathway	Seqs in Pathway	Enzyme	Ezyme ID	Seqs of Enzyme
Biosynthesis of antibiotics	52	dehydrogenase (2-methylpropanoyl-transferring)	ec:1.2.4.4	1
Biosynthesis of antibiotics	52	reductase	ec:1.1.1.133	1
Biosynthesis of antibiotics	52	dehydrogenase	ec:1.2.1.31	1
Biosynthesis of antibiotics	52	synthase	ec:2.5.1.47	2
Biosynthesis of antibiotics	52	penicillinase	ec:3.5.2.6	1
Biosynthesis of antibiotics	52	aldolase	ec:4.1.2.13	3
Biosynthesis of antibiotics	52	isomerase	ec:5.3.1.9	3
Biosynthesis of antibiotics	52	hydratase	ec:4.2.1.11	1
Biosynthesis of antibiotics	52	mutase	ec:5.4.2.10	1
Biosynthesis of antibiotics	52	dehydrogenase (phosphorylating)	ec:1.2.1.12	5
Biosynthesis of antibiotics	52	citrate synthase	ec:2.3.3.8	1
Biosynthesis of antibiotics	52	C-acetyltransferase	ec:2.3.1.9	2
Biosynthesis of antibiotics	52	N-acetyltransferase	ec:2.3.1.1	1
Biosynthesis of antibiotics	52	transaminase	ec:2.6.1.57	2
Biosynthesis of antibiotics	52	transaminase	ec:2.6.1.50	1
Biosynthesis of antibiotics	52	hydroxymethyltransferase	ec:2.1.2.1	1
Biosynthesis of antibiotics	52	(alpha-D-glucose-1,6-bisphosphate-dependent)	ec:5.4.2.2	1
Biosynthesis of antibiotics	52	diphosphate synthase	ec:2.5.1.10	1
Biosynthesis of antibiotics	52	oxidase	ec:1.1.3.15	1
Biosynthesis of antibiotics	52	transaminase	ec:2.6.1.9	2
Biosynthesis of antibiotics	52	transaminase	ec:2.6.1.5	2
Biosynthesis of antibiotics	52	ligase (ADP-forming)	ec:6.2.1.5	2
Biosynthesis of antibiotics	52	transaminase	ec:2.6.1.1	2
Biosynthesis of antibiotics	52	reductase	ec:1.17.1.8	1
Biosynthesis of antibiotics	52	hydratase	ec:4.2.1.3	4
Biosynthesis of antibiotics	52	dehydrogenase (NAD+)	ec:1.2.1.3	1
Biosynthesis of antibiotics	52	synthase	ec:2.2.1.6	1
Biosynthesis of antibiotics	52	kinase	ec:2.7.4.6	1
Biosynthesis of antibiotics	52	kinase	ec:2.7.2.3	1
Biosynthesis of antibiotics	52	reductase	ec:1.5.1.2	1
Biosynthesis of antibiotics	52	geranyl-diphosphate synthase	ec:2.5.1.1	1
Biosynthesis of antibiotics	52	dehydrogenase (NADP+-dependent, decarboxylating)	ec:1.1.1.44	4
Biosynthesis of antibiotics	52	dehydrogenase (NADP+)	ec:1.1.1.42	2
Biosynthesis of antibiotics	52	decarboxylase	ec:4.1.1.1	1
Biosynthesis of antibiotics	52	dehydrogenase	ec:1.1.1.37	1

Biosynthesis of antibiotics	52	dehydrogenase	ec:1.1.1.1	2
Biosynthesis of antibiotics	52	carboxylase	ec:6.4.1.2	1
Biosynthesis of antibiotics	52	phosphatase	ec:3.1.3.25	1
Purine metabolism	27	DNA polymerase	ec:2.7.7.7	2
Purine metabolism	27	RNA polymerase	ec:2.7.7.6	3
Purine metabolism	27	urate hydroxylase	ec:1.7.3.3	1
Purine metabolism	27	(alpha-D-glucose-1,6-bisphosphate-dependent)	ec:5.4.2.2	1
Purine metabolism	27	adenylpyrophosphatase	ec:3.6.1.3	11
Purine metabolism	27	cyclase	ec:4.6.1.1	1
Purine metabolism	27	dehydrogenase	ec:1.17.1.4	1
Purine metabolism	27	kinase	ec:2.7.4.6	1
Purine metabolism	27	phosphatase	ec:3.6.1.40	1
Purine metabolism	27	(asymmetrical)	ec:3.6.1.17	1
Purine metabolism	27	phosphatase	ec:3.6.1.15	5
Purine metabolism	27	metaphosphatase	ec:3.6.1.11	1
Glycolysis / Gluconeogenesis	20	aldolase	ec:4.1.2.13	3
Glycolysis / Gluconeogenesis	20	isomerase	ec:5.3.1.9	3
Glycolysis / Gluconeogenesis	20	hydratase	ec:4.2.1.11	1
Glycolysis / Gluconeogenesis	20	dehydrogenase (phosphorylating)	ec:1.2.1.12	5
Glycolysis / Gluconeogenesis	20	(alpha-D-glucose-1,6-bisphosphate-dependent)	ec:5.4.2.2	1
Glycolysis / Gluconeogenesis	20	phosphotransferase	ec:2.7.1.69	2
Glycolysis / Gluconeogenesis	20	dehydrogenase (ferredoxin)	ec:1.2.7.6	1
Glycolysis / Gluconeogenesis	20	dehydrogenase (NAD+)	ec:1.2.1.3	1
Glycolysis / Gluconeogenesis	20	kinase	ec:2.7.2.3	1
Glycolysis / Gluconeogenesis	20	decarboxylase	ec:4.1.1.1	1
Glycolysis / Gluconeogenesis	20	dehydrogenase	ec:1.1.1.1	2
Methane metabolism	17	aldolase	ec:4.1.2.13	3
Methane metabolism	17	dehydrogenase	ec:1.1.1.284	2
Methane metabolism	17	hydratase	ec:4.2.1.11	1
Methane metabolism	17	hydroxymethyltransferase	ec:2.1.2.1	1
Methane metabolism	17	dehydrogenase	ec:1.2.1.2	1
Methane metabolism	17	dehydrogenase (acceptor)	ec:1.2.99.2	1
Methane metabolism	17	carboxylase	ec:4.1.1.31	1
Methane metabolism	17	kinase	ec:2.7.1.29	1
Methane metabolism	17	dehydrogenase	ec:1.1.1.37	1
Methane metabolism	17	dehydrogenase	ec:1.2.1.46	5
Glyoxylate and dicarboxylate metabolism	16	synthase	ec:2.3.3.9	2

Glyoxylate and dicarboxylate metabolism	16	C-acetyltransferase	ec:2.3.1.9	2
Glyoxylate and dicarboxylate metabolism	16	hydroxymethyltransferase	ec:2.1.2.1	1
Glyoxylate and dicarboxylate metabolism	16	oxidase	ec:1.1.3.15	1
Glyoxylate and dicarboxylate metabolism	16	ec:3.5.1.49 formamidase	ec:3.5.1.49	3
Glyoxylate and dicarboxylate metabolism	16	hydratase	ec:4.2.1.3	4
Glyoxylate and dicarboxylate metabolism	16	dehydrogenase	ec:1.2.1.2	1
Glyoxylate and dicarboxylate metabolism	16	synthase	ec:4.1.1.47	1
Glyoxylate and dicarboxylate metabolism	16	dehydrogenase	ec:1.1.1.37	1
Pentose phosphate pathway	15	aldolase	ec:4.1.2.13	3
Pentose phosphate pathway	15	isomerase	ec:5.3.1.9	3
Pentose phosphate pathway	15	D-xylulose-5-phosphate D-glyceraldehyde-3-phosphate-lyase (phosphate-acetylating)	ec:4.1.2.9	1
Pentose phosphate pathway	15	(alpha-D-glucose-1,6-bisphosphate-dependent)	ec:5.4.2.2	1
Pentose phosphate pathway	15	phosphotransferase	ec:2.7.1.69	2
Pentose phosphate pathway	15	dehydrogenase (NADP+-dependent, decarboxylating)	ec:1.1.1.44	4
Pentose phosphate pathway	15	deoxyribokinase	ec:2.7.1.15	1
Amino sugar and nucleotide sugar metabolism	15	adenylyltransferase	ec:2.7.7.27	1
Amino sugar and nucleotide sugar metabolism	15	isomerase	ec:5.3.1.9	3
Amino sugar and nucleotide sugar metabolism	15	isomerase	ec:5.3.1.8	1
Amino sugar and nucleotide sugar metabolism	15	guanylyltransferase	ec:2.7.7.13	1
Amino sugar and nucleotide sugar metabolism	15	mutase	ec:5.4.2.10	1
Amino sugar and nucleotide sugar metabolism	15	(alpha-D-glucose-1,6-bisphosphate-dependent)	ec:5.4.2.2	1
Amino sugar and nucleotide sugar metabolism	15	deacetylase	ec:3.5.1.41	1
Amino sugar and nucleotide sugar metabolism	15	phosphotransferase	ec:2.7.1.69	2
Amino sugar and nucleotide sugar metabolism	15	end alpha-L-arabinofuranosidase	ec:3.2.1.55	1
Amino sugar and nucleotide sugar metabolism	15	2-epimerase (non-hydrolysing)	ec:5.1.3.14	1
Amino sugar and nucleotide sugar metabolism	15	4-epimerase	ec:5.1.3.2	1
Amino sugar and nucleotide sugar metabolism	15	1,4-beta-xylosidase	ec:3.2.1.37	1
Amino sugar and nucleotide sugar metabolism	15	chitodextrinase	ec:3.2.1.14	1

Amino sugar and nucleotide sugar metabolism	15	6-dehydrogenase	ec:1.1.1.22	1
Cysteine and methionine metabolism	15	synthase	ec:2.5.1.47	2
Cysteine and methionine metabolism	15	(cytosine-5-)-methyltransferase	ec:2.1.1.37	2
Cysteine and methionine metabolism	15	S-adenosylhomocysteine synthase	ec:3.3.1.1	4
Cysteine and methionine metabolism	15	transaminase	ec:2.6.1.57	2
Cysteine and methionine metabolism	15	S-methyltransferase	ec:2.1.1.14	1
Cysteine and methionine metabolism	15	synthase	ec:2.1.1.13	2
Cysteine and methionine metabolism	15	transaminase	ec:2.6.1.5	2
Cysteine and methionine metabolism	15	transaminase	ec:2.6.1.1	2
Cysteine and methionine metabolism	15	adenosyltransferase	ec:2.5.1.6	2
Cysteine and methionine metabolism	15	dehydrogenase	ec:1.1.1.37	1
Carbon fixation pathways in prokaryotes	14	citrate synthase	ec:2.3.3.8	1
Carbon fixation pathways in prokaryotes	14	C-acetyltransferase	ec:2.3.1.9	2
Carbon fixation pathways in prokaryotes	14	ligase (ADP-forming)	ec:6.2.1.5	2
Carbon fixation pathways in prokaryotes	14	hydratase	ec:4.2.1.3	4
Carbon fixation pathways in prokaryotes	14	dehydrogenase (acceptor)	ec:1.2.99.2	1
Carbon fixation pathways in prokaryotes	14	dehydrogenase (NADP+)	ec:1.1.1.42	2
Carbon fixation pathways in prokaryotes	14	carboxylase	ec:4.1.1.31	1
Carbon fixation pathways in prokaryotes	14	dehydrogenase	ec:1.1.1.37	1
Carbon fixation pathways in prokaryotes	14	carboxylase	ec:6.4.1.2	1
Carbon fixation in photosynthetic organisms	14	phosphoketolase	ec:4.1.2.22	1
Carbon fixation in photosynthetic organisms	14	aldolase	ec:4.1.2.13	3
Carbon fixation in photosynthetic organisms	14	dehydrogenase (phosphorylating)	ec:1.2.1.12	5
Carbon fixation in photosynthetic organisms	14	D-xylulose-5-phosphate D-glyceraldehyde-3-phosphate-lyase (phosphate-acetylating)	ec:4.1.2.9	1
Carbon fixation in photosynthetic organisms	14	transaminase	ec:2.6.1.1	2
Carbon fixation in photosynthetic organisms	14	kinase	ec:2.7.2.3	1
Carbon fixation in photosynthetic organisms	14	carboxylase	ec:4.1.1.31	1
Carbon fixation in photosynthetic organisms	14	dehydrogenase	ec:1.1.1.37	1
Starch and sucrose metabolism	12	adenylyltransferase	ec:2.7.7.27	1

Starch and sucrose metabolism	12	synthase	ec:2.4.1.13	1
Starch and sucrose metabolism	12	isomerase	ec:5.3.1.9	3
Starch and sucrose metabolism	12	(alpha-D-glucose-1,6-bisphosphate-dependent)	ec:5.4.2.2	1
Starch and sucrose metabolism	12	1,4-alpha-galacturonidase	ec:3.2.1.67	1
Starch and sucrose metabolism	12	phosphotransferase	ec:2.7.1.69	2
Starch and sucrose metabolism	12	1,4-beta-xylosidase	ec:3.2.1.37	1
Starch and sucrose metabolism	12	invertase	ec:3.2.1.26	1
Starch and sucrose metabolism	12	pectin depolymerase	ec:3.2.1.15	1
Starch and sucrose metabolism	12	6-dehydrogenase	ec:1.1.1.22	1
Glutathione metabolism	10	peroxidase	ec:1.11.1.11	1
Glutathione metabolism	10	transferase	ec:2.5.1.18	1
Glutathione metabolism	10	dehydrogenase (NADP+-dependent, decarboxylating)	ec:1.1.1.44	4
Glutathione metabolism	10	dehydrogenase (NADP+)	ec:1.1.1.42	2
Glutathione metabolism	10	glutamyl transpeptidase	ec:2.3.2.2	1
Glutathione metabolism	10	dehydrogenase (ascorbate)	ec:1.8.5.1	1
Pyruvate metabolism	9	reductase (NADPH)	ec:1.1.1.283	1
Pyruvate metabolism	9	synthase	ec:2.3.3.9	2
Pyruvate metabolism	9	C-acetyltransferase	ec:2.3.1.9	2
Pyruvate metabolism	9	dehydrogenase (NAD+)	ec:1.2.1.3	1
Pyruvate metabolism	9	carboxylase	ec:4.1.1.31	1
Pyruvate metabolism	9	dehydrogenase	ec:1.1.1.37	1
Pyruvate metabolism	9	carboxylase	ec:6.4.1.2	1
Citrate cycle (TCA cycle)	9	citrate synthase	ec:2.3.3.8	1
Citrate cycle (TCA cycle)	9	ligase (ADP-forming)	ec:6.2.1.5	2
Citrate cycle (TCA cycle)	9	hydratase	ec:4.2.1.3	4
Citrate cycle (TCA cycle)	9	dehydrogenase (NADP+)	ec:1.1.1.42	2
Citrate cycle (TCA cycle)	9	dehydrogenase	ec:1.1.1.37	1
Sulfur metabolism	8	dioxygenase	ec:1.14.11.17	1
Sulfur metabolism	8	synthase	ec:2.5.1.47	2
Sulfur metabolism	8	reductase (NADPH)	ec:1.8.1.2	1
Sulfur metabolism	8	sulfurtransferase	ec:2.8.1.1	4
Chloroalkane and chloroalkene degradation	8	dehydrogenase (NAD+)	ec:1.2.1.3	1
Chloroalkane and chloroalkene degradation	8	dehydrogenase	ec:1.1.1.1	2
Chloroalkane and chloroalkene degradation	8	dehydrogenase	ec:1.2.1.46	5
Alanine, aspartate and glutamate metabolism	8	synthase (glutamine-hydrolysing)	ec:6.3.5.5	1
Alanine, aspartate and glutamate	8	synthase (glutamine-hydrolysing)	ec:6.3.5.4	1

metabolism				
Alanine, aspartate and glutamate metabolism	8	transaminase	ec:2.6.1.1	2
Alanine, aspartate and glutamate metabolism	8	decarboxylase	ec:4.1.1.15	2
Alanine, aspartate and glutamate metabolism	8	dehydrogenase	ec:1.4.1.2	1
Alanine, aspartate and glutamate metabolism	8	dehydrogenase	ec:1.4.1.1	1
Pyrimidine metabolism	8	DNA polymerase	ec:2.7.7.7	2
Pyrimidine metabolism	8	RNA polymerase	ec:2.7.7.6	3
Pyrimidine metabolism	8	synthase (glutamine-hydrolysing)	ec:6.3.5.5	1
Pyrimidine metabolism	8	kinase	ec:2.7.4.6	1
Pyrimidine metabolism	8	(asymmetrical)	ec:3.6.1.17	1
Glycerolipid metabolism	7	kinase (ATP)	ec:2.7.1.107	1
Glycerolipid metabolism	7	dehydrogenase (NAD+)	ec:1.2.1.3	1
Glycerolipid metabolism	7	kinase	ec:2.7.1.30	4
Glycerolipid metabolism	7	kinase	ec:2.7.1.29	1
Fructose and mannose metabolism	7	aldolase	ec:4.1.2.13	3
Fructose and mannose metabolism	7	isomerase	ec:5.3.1.8	1
Fructose and mannose metabolism	7	guanylyltransferase	ec:2.7.7.13	1
Fructose and mannose metabolism	7	phosphofructokinase 2	ec:2.7.1.105	1
Fructose and mannose metabolism	7	phosphotransferase	ec:2.7.1.69	2
Fructose and mannose metabolism	7	2-phosphatase	ec:3.1.3.46	1
Taurine and hypotaurine metabolism	6	dioxygenase	ec:1.14.11.17	1
Taurine and hypotaurine metabolism	6	glutamyl transpeptidase	ec:2.3.2.2	1
Taurine and hypotaurine metabolism	6	decarboxylase	ec:4.1.1.15	2
Taurine and hypotaurine metabolism	6	dehydrogenase	ec:1.4.1.2	1
Taurine and hypotaurine metabolism	6	dehydrogenase	ec:1.4.1.1	1
Arginine and proline metabolism	6	agmatine ureohydrolase	ec:3.5.3.11	1
Arginine and proline metabolism	6	transaminase	ec:2.6.1.1	2
Arginine and proline metabolism	6	dehydrogenase (NAD+)	ec:1.2.1.3	1
Arginine and proline metabolism	6	acylamidase	ec:3.5.1.4	1
Arginine and proline metabolism	6	reductase	ec:1.5.1.2	1
Cyanoamino acid metabolism	6	hydroxymethyltransferase	ec:2.1.2.1	1
Cyanoamino acid metabolism	6	ec:3.5.1.49 formamidase	ec:3.5.1.49	3
Cyanoamino acid metabolism	6	glutamyl transpeptidase	ec:2.3.2.2	1
Cyanoamino acid metabolism	6	synthase	ec:4.4.1.9	1
Butanoate metabolism	6	C-acetyltransferase	ec:2.3.1.9	2
Butanoate metabolism	6	synthase	ec:2.2.1.6	1

Butanoate metabolism	6	ligase	ec:6.2.1.16	1
Butanoate metabolism	6	decarboxylase	ec:4.1.1.15	2
Propanoate metabolism	6	reductase (NADPH)	ec:1.1.1.283	1
Propanoate metabolism	6	C-acetyltransferase	ec:2.3.1.9	2
Propanoate metabolism	6	ligase (ADP-forming)	ec:6.2.1.5	2
Propanoate metabolism	6	carboxylase	ec:6.4.1.2	1
Ascorbate and aldarate metabolism	6	peroxidase	ec:1.11.1.11	1
Ascorbate and aldarate metabolism	6	phosphotransferase	ec:2.7.1.69	2
Ascorbate and aldarate metabolism	6	dehydrogenase (NAD+)	ec:1.2.1.3	1
Ascorbate and aldarate metabolism	6	dehydrogenase (ascorbate)	ec:1.8.5.1	1
Ascorbate and aldarate metabolism	6	6-dehydrogenase	ec:1.1.1.22	1
Nitrogen metabolism	5	ec:3.5.1.49 formamidase	ec:3.5.1.49	3
Nitrogen metabolism	5	dehydratase	ec:4.2.1.1	1
Nitrogen metabolism	5	dehydrogenase	ec:1.4.1.2	1
Valine, leucine and isoleucine degradation	5	dehydrogenase (2-methylpropanoyl-transferring)	ec:1.2.4.4	1
Valine, leucine and isoleucine degradation	5	C-acetyltransferase	ec:2.3.1.9	2
Valine, leucine and isoleucine degradation	5	dehydrogenase (NAD+)	ec:1.2.1.3	1
Valine, leucine and isoleucine degradation	5	ligase	ec:6.2.1.16	1
Fatty acid degradation	5	C-acetyltransferase	ec:2.3.1.9	2
Fatty acid degradation	5	dehydrogenase (NAD+)	ec:1.2.1.3	1
Fatty acid degradation	5	dehydrogenase	ec:1.1.1.1	2
Galactose metabolism	5	(alpha-D-glucose-1,6-bisphosphate-dependent)	ec:5.4.2.2	1
Galactose metabolism	5	phosphotransferase	ec:2.7.1.69	2
Galactose metabolism	5	4-epimerase	ec:5.1.3.2	1
Galactose metabolism	5	invertase	ec:3.2.1.26	1
Histidine metabolism	5	transaminase	ec:2.6.1.9	2
Histidine metabolism	5	dehydrogenase (NAD+)	ec:1.2.1.3	1
Histidine metabolism	5	hydratase	ec:4.2.1.49	2
Thiamine metabolism	5	phosphatase	ec:3.6.1.15	5
Oxidative phosphorylation	4	reductase (H+-translocating)	ec:1.6.5.3	3
Oxidative phosphorylation	4	kinase	ec:2.7.4.1	1
Streptomycin biosynthesis	4	reductase	ec:1.1.1.133	1
Streptomycin biosynthesis	4	transaminase	ec:2.6.1.50	1
Streptomycin biosynthesis	4	(alpha-D-glucose-1,6-bisphosphate-dependent)	ec:5.4.2.2	1
Streptomycin biosynthesis	4	phosphatase	ec:3.1.3.25	1
Tryptophan metabolism	4	C-acetyltransferase	ec:2.3.1.9	2

Tryptophan metabolism	4	dehydrogenase (NAD+)	ec:1.2.1.3	1
Tryptophan metabolism	4	acylamidase	ec:3.5.1.4	1
Arginine biosynthesis	4	N-acetyltransferase	ec:2.3.1.1	1
Arginine biosynthesis	4	transaminase	ec:2.6.1.1	2
Arginine biosynthesis	4	dehydrogenase	ec:1.4.1.2	1
Lysine degradation	4	dehydrogenase	ec:1.2.1.31	1
Lysine degradation	4	C-acetyltransferase	ec:2.3.1.9	2
Lysine degradation	4	dehydrogenase (NAD+)	ec:1.2.1.3	1
C5-Branched dibasic acid metabolism	4	lyase	ec:4.1.3.22	1
C5-Branched dibasic acid metabolism	4	ligase (ADP-forming)	ec:6.2.1.5	2
C5-Branched dibasic acid metabolism	4	synthase	ec:2.2.1.6	1
Lysine biosynthesis	4	dehydrogenase	ec:1.2.1.31	1
Lysine biosynthesis	4	transaminase	ec:2.6.1.57	2
Lysine biosynthesis	4	reductase	ec:1.17.1.8	1
Peptidoglycan biosynthesis	4	glycosyltransferase	ec:2.4.1.129	4
Tyrosine metabolism	4	transaminase	ec:2.6.1.57	2
Tyrosine metabolism	4	transaminase	ec:2.6.1.9	2
Tyrosine metabolism	4	transaminase	ec:2.6.1.5	2
Tyrosine metabolism	4	transaminase	ec:2.6.1.1	2
Tyrosine metabolism	4	dehydrogenase	ec:1.1.1.1	2
Drug metabolism - cytochrome P450	3	transferase	ec:2.5.1.18	1
Drug metabolism - cytochrome P450	3	dehydrogenase	ec:1.1.1.1	2
Metabolism of xenobiotics by cytochrome P450	3	transferase	ec:2.5.1.18	1
Metabolism of xenobiotics by cytochrome P450	3	dehydrogenase	ec:1.1.1.1	2
Ubiquinone and other terpenoid-quinone biosynthesis	3	transaminase	ec:2.6.1.5	2
Ubiquinone and other terpenoid-quinone biosynthesis	3	ligase	ec:6.2.1.26	1
One carbon pool by folate	3	hydroxymethyltransferase	ec:2.1.2.1	1
One carbon pool by folate	3	synthase	ec:2.1.1.13	2
beta-Alanine metabolism	3	dehydrogenase (NAD+)	ec:1.2.1.3	1
beta-Alanine metabolism	3	decarboxylase	ec:4.1.1.15	2
Terpenoid backbone biosynthesis	3	C-acetyltransferase	ec:2.3.1.9	2
Terpenoid backbone biosynthesis	3	diphosphate synthase	ec:2.5.1.10	1
Terpenoid backbone biosynthesis	3	geranyl-diphosphate synthase	ec:2.5.1.1	1
Benzoate degradation	3	2,3-dioxygenase	ec:1.13.11.2	1
Benzoate degradation	3	C-acetyltransferase	ec:2.3.1.9	2
Phenylalanine metabolism	3	transaminase	ec:2.6.1.57	2

Phenylalanine metabolism	3	transaminase	ec:2.6.1.9	2
Phenylalanine metabolism	3	transaminase	ec:2.6.1.5	2
Phenylalanine metabolism	3	transaminase	ec:2.6.1.1	2
Phenylalanine metabolism	3	acylamidase	ec:3.5.1.4	1
Glycine, serine and threonine metabolism	3	hydroxymethyltransferase	ec:2.1.2.1	1
Glycine, serine and threonine metabolism	3	dehydrogenase	ec:1.1.1.1	2
Phenylpropanoid biosynthesis	3	lactoperoxidase	ec:1.11.1.7	3
Retinol metabolism	3	15,15'-monooxygenase	ec:1.14.99.36	1
Retinol metabolism	3	dehydrogenase	ec:1.1.1.1	2
Pentose and glucuronate interconversions	3	1,4-alpha-galacturonidase	ec:3.2.1.67	1
Pentose and glucuronate interconversions	3	dehydrogenase (NAD+)	ec:1.2.1.3	1
Pentose and glucuronate interconversions	3	pectin depolymerase	ec:3.2.1.15	1
Pentose and glucuronate interconversions	3	6-dehydrogenase	ec:1.1.1.22	1
alpha-Linolenic acid metabolism	2	dehydrogenase	ec:1.1.1.1	2
Aminobenzoate degradation	2	reductase	ec:1.6.5.7	1
Aminobenzoate degradation	2	acylamidase	ec:3.5.1.4	1
Naphthalene degradation	2	dehydrogenase	ec:1.1.1.1	2
Tropane, piperidine and pyridine alkaloid biosynthesis	2	transaminase	ec:2.6.1.57	2
Tropane, piperidine and pyridine alkaloid biosynthesis	2	transaminase	ec:2.6.1.9	2
Tropane, piperidine and pyridine alkaloid biosynthesis	2	transaminase	ec:2.6.1.5	2
Tropane, piperidine and pyridine alkaloid biosynthesis	2	transaminase	ec:2.6.1.1	2
Synthesis and degradation of ketone bodies	2	C-acetyltransferase	ec:2.3.1.9	2
Novobiocin biosynthesis	2	transaminase	ec:2.6.1.57	2
Novobiocin biosynthesis	2	transaminase	ec:2.6.1.9	2
Novobiocin biosynthesis	2	transaminase	ec:2.6.1.5	2
Novobiocin biosynthesis	2	transaminase	ec:2.6.1.1	2
Phenylalanine, tyrosine and tryptophan biosynthesis	2	transaminase	ec:2.6.1.57	2
Phenylalanine, tyrosine and tryptophan biosynthesis	2	transaminase	ec:2.6.1.9	2
Phenylalanine, tyrosine and tryptophan biosynthesis	2	transaminase	ec:2.6.1.5	2
Phenylalanine, tyrosine and tryptophan biosynthesis	2	transaminase	ec:2.6.1.1	2
Isoquinoline alkaloid biosynthesis	2	transaminase	ec:2.6.1.57	2

Isoquinoline alkaloid biosynthesis	2	transaminase	ec:2.6.1.5	2
Isoquinoline alkaloid biosynthesis	2	transaminase	ec:2.6.1.1	2
Chlorocyclohexane and chlorobenzene degradation	2	2,3-dioxygenase	ec:1.13.11.2	1
Chlorocyclohexane and chlorobenzene degradation	2	reductase	ec:1.6.5.7	1
Phosphatidylinositol signaling system	2	kinase (ATP)	ec:2.7.1.107	1
Phosphatidylinositol signaling system	2	phosphatase	ec:3.1.3.25	1
Selenocompound metabolism	2	S-methyltransferase	ec:2.1.1.14	1
Selenocompound metabolism	2	synthase	ec:2.1.1.13	2
Styrene degradation	2	2,3-dioxygenase	ec:1.13.11.2	1
Styrene degradation	2	acylamidase	ec:3.5.1.4	1
Caffeine metabolism	1	urate hydroxylase	ec:1.7.3.3	1
Valine, leucine and isoleucine biosynthesis	1	synthase	ec:2.2.1.6	1
Xylene degradation	1	2,3-dioxygenase	ec:1.13.11.2	1
Polyketide sugar unit biosynthesis	1	reductase	ec:1.1.1.133	1
Aminoacyl-tRNA biosynthesis	1	ligase	ec:6.1.1.6	1
Pantothenate and CoA biosynthesis	1	synthase	ec:2.2.1.6	1
Various types of N-glycan biosynthesis	1	1,2-alpha-mannosidase	ec:3.2.1.113	1
Zeatin biosynthesis	1	dimethylallyltransferase	ec:2.5.1.75	1
Other glycan degradation	1	alpha-D-mannosidase	ec:3.2.1.24	1
N-Glycan biosynthesis	1	1,2-alpha-mannosidase	ec:3.2.1.113	1
Limonene and pinene degradation	1	dehydrogenase (NAD+)	ec:1.2.1.3	1
Penicillin and cephalosporin biosynthesis	1	penicillinase	ec:3.5.2.6	1
Porphyrin and chlorophyll metabolism	1	2,1-aminomutase	ec:5.4.3.8	1
Sphingolipid metabolism	1	sulfatase	ec:3.1.6.1	1
Glycerophospholipid metabolism	1	kinase (ATP)	ec:2.7.1.107	1
Inositol phosphate metabolism	1	phosphatase	ec:3.1.3.25	1
beta-Lactam resistance	1	penicillinase	ec:3.5.2.6	1
T cell receptor signaling pathway	1	phosphatase	ec:3.1.3.16	1
Monobactam biosynthesis	1	reductase	ec:1.17.1.8	1
Fatty acid biosynthesis	1	carboxylase	ec:6.4.1.2	1
Flavonoid biosynthesis	1	synthase	ec:2.3.1.170	1
Aflatoxin biosynthesis	1	carboxylase	ec:6.4.1.2	1
Tetracycline biosynthesis	1	carboxylase	ec:6.4.1.2	1
Nitrotoluene degradation	1	dehydrogenase (acceptor)	ec:1.2.99.2	1
Folate biosynthesis	1	diphosphokinase	ec:2.7.6.3	1
Steroid hormone biosynthesis	1	sulfatase	ec:3.1.6.1	1

Supplementary Figure S1: Organismal classification of the identified proteins inside and outside the brûlé; a: superkingdom level; b: kingdom level; c: phylum level; d: class level.

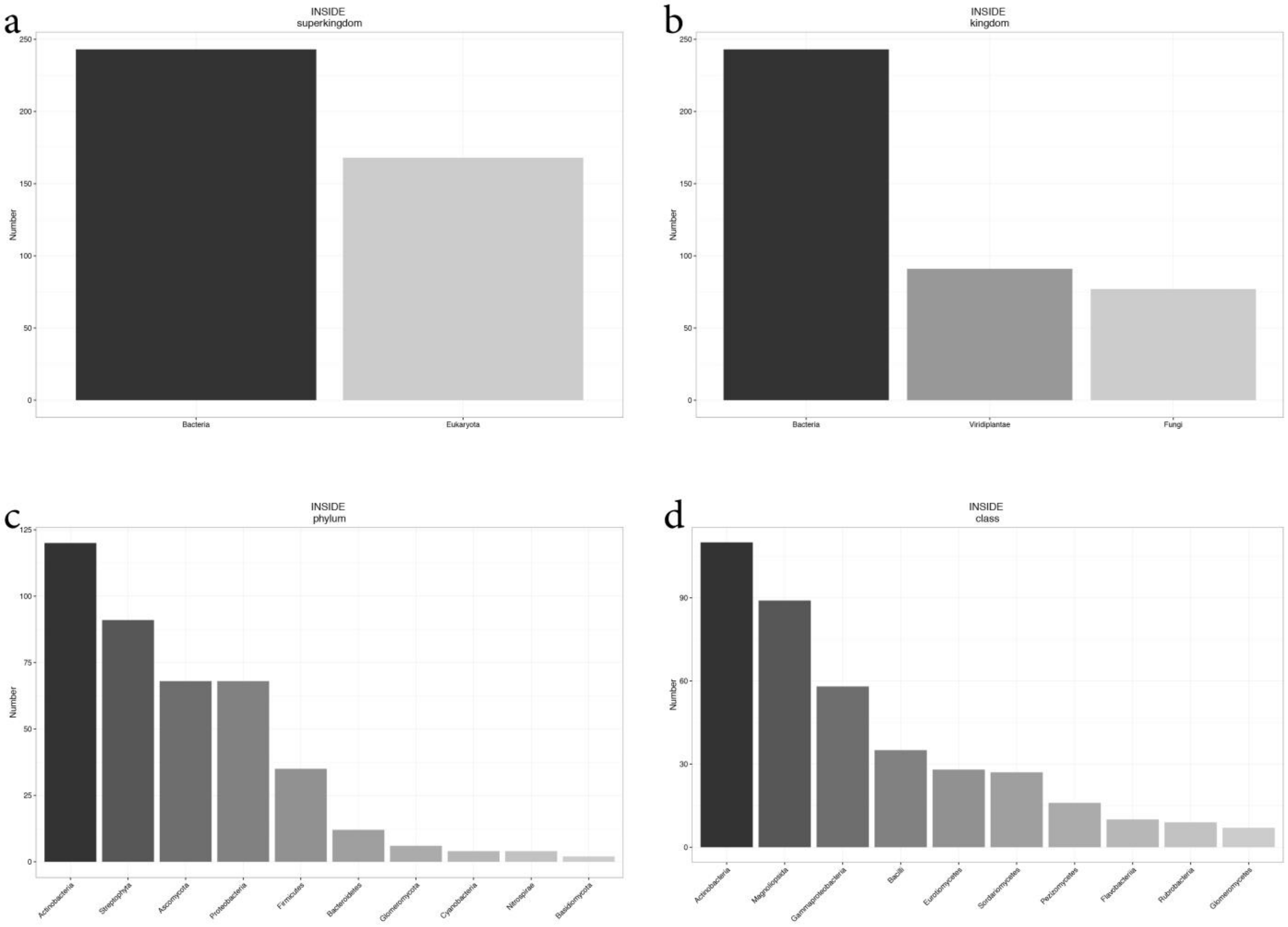

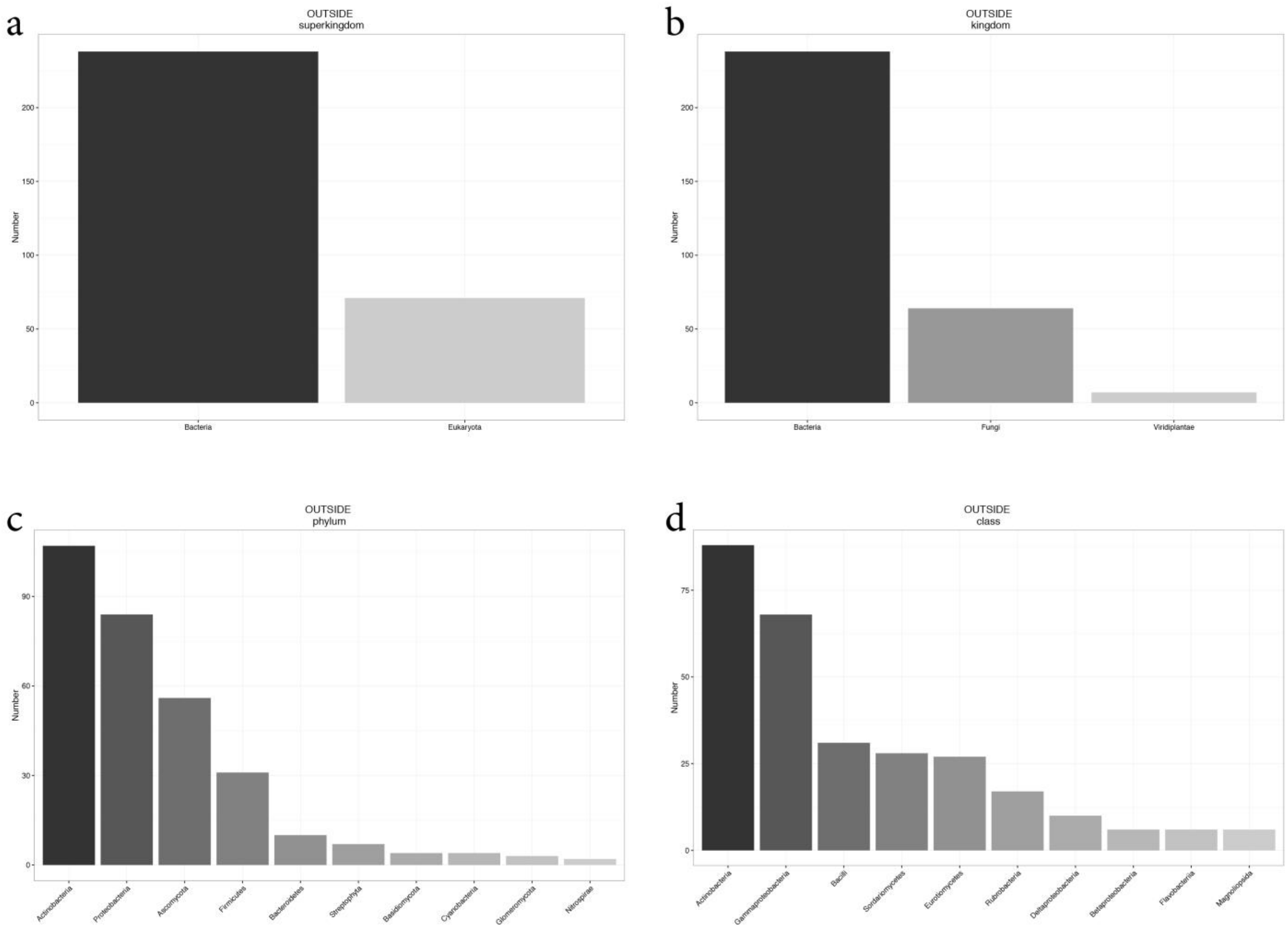
a
OUTSIDE
superkingdom
Number
0
50
100
150
200
Bacteria
Eukaryota
b
OUTSIDE
kingdom
Number
0
50
100
150
200
Bacteria
Fungi
Viridiplantae
c
OUTSIDE
phylum
Number
0
30
60
90
Actinobacteria
Proteobacteria
Ascomycota
Firmicutes
Bacteroidetes
Streptophyta
Basidiomycota
Cyanobacteria
Glomeromycota
Nitrospirae
d
OUTSIDE
class
Number
0
25
50
75
Actinobacteria
Gammaproteobacteria
Bacilli
Sordariomycetes
Eurotiomycetes
Rubrobacteria
Deltaproteobacteria
Betaproteobacteria
Flavobacteria
Magnoliopsida
